# Induction and maintenance of mucosal healing in Crohn’s disease with ustekinumab in clinical practice across all care levels in Germany (MUCUS)

**DOI:** 10.1038/s41598-024-70241-9

**Published:** 2024-09-03

**Authors:** Daniel C. Baumgart, Andreas Stallmach, Philip Grunert, Stefan Schubert, Stefanie Howaldt, Ulrike von Arnim, Thomas Ochsenkühn, Jürgen Stein, Andreas Lügering, Daniel Schmidt, Marten Schulz, Andreas Fischer

**Affiliations:** 1 Medizinische Klinik m.S. Hepatologie und Gastroenterologie, Charité-Universitätsmedizin Berlin, Berlin, Germany; 2 Klinik für Innere Medizin IV Gastroenterologie, Hepatologie, Infektiologie, Zentrale Endoskopie, Universitätsklinikum Jena, Jena, Germany; 3 Gastroenterologie am Bayerischen Platz, Gastroenterologie, Berlin, Germany; 4 Hamburgisches Forschungsinstitut für chronisch entzündliche Darmerkrankungen, Immunologie, Hamburg, Germany; 5 Universitätsklinikum Magdeburg, Universitätsklinik für Gastroenterologie, Hepatologie und Infektiologie, Magdeburg, Germany; 6 Isarklinikum, Klinik für Gastroenterologie, Hepatologie und Gastroenterologische Onkologie, München, Germany; 7 DGD Kliniken Frankfurt Sachsenhausen, Abteilung Gastroenterologie/Ernährungsmedizin, Frankfurt am Main, Germany; 8 MVZ Portal 10, Zentrum für Leber-, Magen- und Darmerkrankungen, Münster, Germany

**Keywords:** Crohn’s disease, Inflammatory bowel disease, Ustekinumab, Mucosal healing, Extraintestinal manifestations, Quality of life, Gastrointestinal diseases, Inflammatory bowel disease, Crohn's disease

## Abstract

The impact of ustekinumab (UST) on mucosal- and fistula healing and extraintestinal manifestations (EIM) in Crohn’s disease (CD) were not fully elucidated in the registration trials. In this prospective, multicenter study (EudraCT number: 2017-005151-83) we evaluated the German label real-world-effectiveness of UST to achieve the primary endpoint of combined clinical and endoscopic response at week 52 and several secondary endpoints. Of 79 screened we enrolled 52 patients (female n = 28, bionaïve n = 13, biologic n = 39). At week 52 (per protocol analysis), 52% (n = 13/25) of patients achieved the primary endpoint [50% (n = 3/6) in the bionaïve, 45.5% (n = 5/11) biologic, 62.5% (n = 5/8 ) multiple biologics cohorts, respectively with age as independent predictor [OR 95% CI 0.933 (0.873, 0.998) p = 0.043], 60% (n = 15/25) achieved endoscopic response [50% (n = 3/6) in the bionaïve, 54.5% (n = 6/11) biologic, 75% (n = 6/8) multiple biologics cohorts, respectively], 36% (n = 9/25) achieved endoscopic remission [50% (n = 3/6) in the bionaïve, 27.3% (n = 3/11) biologic, 37.5% (n = 3/8) multiple biologics cohorts, respectively], 48% (n = 12/25) achieved mucosal healing [50% (n = 3/6) in the bionaïve, 36.4% (n = 4/11) biologic, 62.5% (n = 5/8) multiple biologics cohorts, respectively]. All achieved a fistula response and 33.3% (n = 1/3) in the multiple biologics group fistula remission at week 52. EIM decreased (week 0 28.2% vs. week 52 8%). CRP, FCP, PRO-2, EQ-5D-5L improved throughout. 36 patients (69.2%) experienced ≥ 1 treatment emergent adverse event, in 8 (15.4%) cases rated as severe and in 5 (9.6%) leading to UST discontinuation, but no very severe events or deaths. The effectiveness of UST was better than in the registration trials.

## Introduction

Crohn’s disease (CD) is an idiopathic inflammatory bowel disease (IBD) that cannot be cured and challenges patients and healthcare systems worldwide^1^. CD results from an inappropriate immune response towards the commensal microbiota in genetically susceptible individuals^2^, exacerbated and promoted by environmental factors such as Western lifestyle, diet and industrialization^3^. CD requires lifelong medical therapy and progresses with repeated flare-ups to possible complete digestive failure^4^. The chronic inflammation is associated with an early, increased risk of various digestive and other cancers^5^.

Due to its increasing incidence and early age peaks in the 2nd and 3rd decade, CD impacts on patients’ personal and professional lives due to impaired activity, lost work-productivity, fatigue, as well as early disability. The complete economic burden has been estimated at and 2.1 to 16.7 billion € for CD annually in Europe^6–8^. Recent research from our group comparing actual costs and revenues indicates, that inpatient care expenditures are almost double compared with the average population and not fully recovered by German diagnosis related group (G-DRG) proceeds^9^. Biologic medications^10^ contribute the most to the deficit. Thus, evidence driven patient and treatment stratification are paramount.

The perceived quality of and satisfaction with CD care delivered varies substantially between patients^11^ and providers^12^. Patient reported outcomes (PRO)^13^ and patient relevant endpoints^14^ are not systematically collected^15^ in Germany, despite their demanded implementation by major regulatory bodies^14,16^ and their immediate impact on the approval and reimbursement of novel compounds such as ustekinumab. More than one third of all IBD patients in Germany are not in remission^17^ underscoring the need to implement and study the real-world application of new therapies.

Ustekinumab was compared to placebo in the UNITI-1 trial in CD patients who had failed anti-TNFα and in the UNITI-2 trial, carried out in CD patients who had failed conventional therapy as well as in a maintenance study with re-randomized responders (IM-UNITI)^18,19^. In endoscopic and histologic substudies of patients who participated in UNITI-1, UNITI-2 and IM-UNITI, ustekinumab induced a greater reduction in the Simplified Endoscopic Activity Score for Crohn’s Disease (SES-CD) and global histology activity score reduction at week 8 than placebo^20,21^. Ustekinumab was approved for CD based on UNITI and IM-UNITI (and later also for ulcerative colitis results based on the UNIFI prospective randomized controlled trial)^22^.

Since clinical trials rarely represent the real world patient population^23^ and ustekinumab’s impact on induction and maintenance of mucosal healing, fistula healing and extraintestinal manifestations in CD are largely unknown long-term remission patients reported outcomes and quality of life data are incomplete we aim to close these knowledge gaps.

## Materials and methods

### Study drug ustekinumab

Ustekinumab is a fully human immunoglobulin G1 kappa (IgG1k) monoclonal antibody to human IL-12/23p40 that binds with high affinity to the p40 subunit of human IL-12 and IL-23^10^. By inhibiting interaction with the cell surface IL-12Rβ1 receptor protein, ustekinumab effectively neutralizes all IL-12 (Th1) and IL-23 (Th17) mediated cellular responses. Abnormal regulation of IL-12 and IL-23 has been associated with multiple immune-mediated diseases including CD.

### Study design

In this prospective, open label, nationwide, multicenter, phase IV study we investigated ustekinumab use in adult subjects with active, moderate to severe, ileal and/or colonic CD in a real world setting in 8 (originally planned 10) German centers representing all care levels—private practice, community hospitals and academic institutions. The drug was used in accordance with its German label^24–26^.

An initial target of 100 adult male and female subjects with endoscopic evidence of active disease were planned to be enrolled, but later adjusted to 50–60 due to pandemic related issues. Patients enrolled were either naïve to biologic treatment or previously had an inadequate response, loss of response, been intolerant to, or had medical contraindications to either 1, or 2 or more biologic(s) approved for the treatment of CD. Eligibility of subjects was evaluated locally, and centrally read endoscopic assessments at screening was used for baseline evaluation. At week 0, all eligible subjects initiated IV induction treatment with ustekinumab, in line with the German label, on a weight-tiered basis at a dose of approximately 6 mg/kg IV. At week 8, all subjects received a 90 mg SC injection of ustekinumab. To ensure a balanced, unbiased real world cohort subjects were be stratified 1:1:1 at baseline according to being naïve to biologics (bionaïve), prior exposure to 1 (biologic) or 2 or more biologics (multiple biologic) for treatment of Crohn’s disease. All subjects were to be followed for 52 weeks. Consideration was given to discontinuing treatment in patients who show no evidence of therapeutic benefit by week 16 or 16 weeks after switching to the 8-weekly dose. In accordance with the German label, subjects who lost response during 12-weekly dosing may benefit from an adjustment to 8-weekly maintenance treatment. Subjects were subsequently dosed every 8 weeks or every 12 weeks according to clinical judgment. In contrast, subjects already receiving 8-weekly treatment were not able to adjust the ustekinumab dose following disease flare and left the study (early termination visit).

### Study endpoints and assessments

Assessments of vital signs, concomitant medications, procedures (i.e. surgeries and other non-medical interventions) hospitalizations, extraintestinal manifestations, clinical and biochemical disease activity (Harvey Bradshaw Index [HBI]^27^, complete blood count [CBC], C-reactive protein [CRP], albumin and fecal calprotectin [FCP]) were performed throughout the study at the times indicated in the Time and Events Schedule. For subjects with fistulizing disease, perianal disease activity index (PDAI)^28^ was assessed. Patient-reported outcomes^29^ included assessments of PRO-2^30^ and non-disease specific health related quality of life (EQ-5D-5L)^31^. Adverse event data and information on concomitant therapies according to Medical Dictionary for Regulatory Activities (MedDRA)^32^ and Common Terminology for Adverse Events (CTCAE)^33^ were collected throughout the study. Immunogenicity assessments included an inspection of the infusion and injection sites for reactions and monitoring of vital signs for systemic allergic or other immunological reactions. An ileocolonoscopic assessment of endoscopic disease activity with the Simple Endoscopic Score for Crohn’s Disease (SES-CD)^34^ and mucosal healing documentation via central reading was performed at the Week 0, voluntarily at Week 26 and mandatory at the Week 52 study visits. For patients receiving corticosteroids at baseline tapering was mandatory from Week 16 onwards. For detailed information on the definition and timing of efficacy and safety assessments throughout the study refer to Supplementary Web Appendix Part 1: Web Table 1 Time and Event Schedule and Web Table 2 Objectives and Endpoints.

### Statistical considerations

All subjects who received at least one dose of ustekinumab were included in the safety analysis set. All subjects who received at least one dose of ustekinumab and have at least one post-stratification efficacy assessment were included in the intent-to-treat (ITT) analysis set. In addition, all subjects who continued to the maintenance phase and received treatment were included in the maintenance ITT population. All ITT subjects who completed all aspects of the trial and did not have any major deviations from protocol were included in the per-protocol (PP) analysis set. PP analyses were performed on the primary efficacy variable.

In line with the Selecting Therapeutic Targets in Inflammatory Bowel Disease (STRIDE)^35,36^ consensus, *a combined endpoint of clinical response plus endoscopic disease activity was set as the primary endpoint* of this study. This was not an RCT and hence had no placebo comparator. Assuming a combined clinical and endoscopic response rate of 40%, the anticipated asymptotic 95% confidence interval (CI) about the remission rate will be ± 10% with 60 subjects; to account for attrition, 68 subjects were planned to be enrolled. Enrolled patients who stopped treatment before reaching week 52 due to any reason, or patients without endoscopic data at week 52 were analyzed as non-responders. The number and percentage of responders were summarized by stratification groups and compared using a generalized linear model with a logit link. Baseline history of CD related surgeries, baseline mono versus combination therapy status, and baseline fistulizing versus non-fistulizing CD status were included as covariates. Least squares means odds ratios (OR) and 95% confidence intervals (CI) were provided for each stratification group.

All available efficacy and safety data collected for the study were included in data listings and/or summary tables. Categorical endpoints that were assessed at end of the study imputed missing values using non-responder imputation. All other endpoints were assessed using mixed models and not imputed.

### Ethical statement

The study was approved by central ethics committee of the state government of Berlin Germany (Landesamt für Gesundheit und Soziales, Ethik Kommission des Landes Berlin 18/0263-EK13). All methods were carried out in accordance with relevant guidelines and regulations. informed consent was obtained from all subjects and/or their legal guardian(s).

### Study registration

This non-interventional study was registered with EudraCT (European Union Drug Regulating Authorities Clinical Trials Database) reference number 2017-005151-83. Details can be found here: https://www.clinicaltrialsregister.eu/ctr-search/search?query=2017-005151-83.

## Results

### Patient disposition

We screened 79 patients, of which 34% (27/79) were screening failures and 66% (52/79) were enrolled in the study. 65 percent of patients (34/52) completed the study, this attrition rate was likely impacted by the unprecedented global pandemic. A greater proportion of patients in the bionaïve group completed the study. The reasons for discontinuation were comparable among the groups. However, the highest number of discontinuations due to non-HBI response was recorded in the biologic group whereas patient discontinuing due loss to follow-up occurred only in the multiple-biologic group. Table 1,2summarizes treatment compliance. For additional details about treatment compliance and protocol adherence, refer to Supplementary Web Appendix Part 2 Web Tables 14.1.3 and 14.1.7.

**Table 1:** Patient disposition.

	Naive (N = 13)	Biologic (N = 22)	Multiple biologic (N = 17)	Total (N = 52)
Study disposition: n (%)				
Screened				79
Screen failed				27
Randomized	13	22	17	52
Discontinued from study	3 (23.1%)	8 (36.4%)	7 (41.2%)	18 (34.6%)
Completed study	10 (76.9%)	14 (63.6%)	10 (58.8%)	34 (65.4%)
Eligible for safety analysis set	13 (100.0%)	22 (100.0%)	17 (100.0%)	52 (100.0%)
Eligible for ITT analysis set	13 (100.0%)	22 (100.0%)	17 (100.0%)	52 (100.0%)
Eligible for per-protocol analysis set	6 (46.2%)	11 (50.0%)	8 (47.1%)	25 (48.1%)
Reasons for discontinuation: n (%)	3 (23.1%)	8 (36.4%)	7 (41.2%)	18 (34.6%)
Adverse event	1 (7.7%)	1 (4.5%)	1 (5.9%)	3 (5.8%)
Lost to follow-up	–	–	3 (17.6%)	3 (5.8%)
Non-HBI responder	1 (7.7%)	3 (13.6%)	1 (5.9%)	5 (9.6%)
Other	–	1 (4.5%)	1 (5.9%)	2 (3.8%)
Pregnancy	–	1 (4.5%)	–	1 (1.9%)
Subject decision	1 (7.7%)	2 (9.1%)	1 (5.9%)	4 (7.7%)

*ITT* intent-to-treat analysis set, *N* number of randomized subjects, *n* number of subjects within each category, *HBI* Harvey Bradshaw Index.

**Table 2:** Treatment compliance.

	Naive (N = 13)	Biologic (N = 22)	Multiple biologic (N = 17)
Number of injections administered			
N	13	22	17
Mean (SD)	6.8 (2.35)	5.9 (2.55)	6.1 (2.99)
Median (min, max)	7.0 (1, 9)	6.0 (1, 10)	6.0 (1, 11)
Number of subjects who received > 6 injections	10 (76.9%)	8 (36.4%)	8 (47.1%)
Number of subjects who received 6 injections	1 (7.7%)	5 (22.7%)	3 (17.6%)
Number of subjects who received < 6 injections	2 (15.4%)	9 (40.9%)	6 (35.3%)

*N* number of randomized subjects, *n* number of subjects within each category, *SD* Standard Deviation, *min* minimum, *max* maximum.

### Patient baseline characteristics

Sex distribution was for the most part equal. Most patients enrolled were in their 40s and shared the same physical status according to their respective BMI. All patients were of Caucasian descent. Patients in the biologic- and multiple biologics groups had more frequently ileocolonic and fistulizing disease and were more severe as indicated by higher baseline CRP, FCP and SES-CD score values, the most severe group being those previously exposed to multiple biologics. Table 3 For additional details about patient’s prior treatment complications, medical history, medications, concomitant medications please refer to the Supplementary Web Appendix Part 2 Web Tables 14.1.3–14.1.6.

**Table 3:** Patient baseline characteristics (safety population).

	Naive (N = 13)	Biologic (N = 22)	Multiple biologic (N = 17)	Total (N = 52)
Gender: n (%)				
Female	8 (61.5%)	14 (63.6%)	6 (35.3%)	28 (53.8%)
Male	5 (38.5%)	8 (36.4%)	11 (64.7%)	24 (46.2%)
Age: (years)				
n	13	22	17	52
Mean (SD)	39.0 (15.25)	40.3 (12.01)	39.5 (10.43)	39.7 (12.19)
Median (min, max)	39.2 (20, 64)	36.7 (21, 74)	42.4 (21, 57)	39.3 (20, 74)
Race: n (%)				
White	13 (100.0%)	22 (100.0%)	17 (100.0%)	52 (100.0%)
Prior Crohn’s surgery: n (%)				
Yes	–	3 (13.6%)	4 (23.5%)	7 (13.5%)
No	13 (100.0%)	19 (86.4%)	13 (76.5%)	45 (86.5%)
Concomitant monotherapy immunomodulators: n (%)				
Yes	–	2 (9.1%)	1 (5.9%)	3 (5.8%)
No	13 (100.0%)	20 (90.9%)	16 (94.1%)	49 (94.2%)
Concomitant combination therapy immunomodulators: n (%)				
Yes	–	–	–	–
No	13 (100.0%)	22 (100.0%)	17 (100.0%)	52 (100.0%)
Fistulizing Crohn’s: n (%)				
Yes	–	2 (9.1%)	5 (29.4%)	7 (13.5%)
No	13 (100.0%)	20 (90.9%)	12 (70.6%)	45 (86.5%)
Weight: (kg)				
n	12	20	15	47
Mean (SD)	73.0 (17.01)	72.8 (14.68)	72.3 (16.09)	72.7 (15.40)
Median (min, max)	79.5 (39, 93)	68.0 (51, 103)	72.0 (47, 106)	72.0 (39, 106)
Height: (cm)				
n	12	20	16	48
Mean (SD)	173.3 (14.89)	171.3 (10.61)	173.5 (7.80)	172.5 (10.86)
Median (min, max)	169.0 (147, 195)	169.0 (157, 192)	172.0 (161, 195)	171.0 (147, 195)
Body mass index: (kg/m^2^)				
n	12	20	15	47
Mean (SD)	24.2 (4.93)	24.7 (3.59)	23.8 (4.46)	24.3 (4.16)
Median (min, max)	23.5 (18, 34)	23.7 (19, 33)	22.4 (17, 32)	23.3 (17, 34)
Extraintestinal manifestations: n (%)				
Yes	5 (38.5%)	5 (22.7%)	4 (23.5%)	14 (26.9%)
No	8 (61.5%)	17 (77.3%)	13 (76.5%)	38 (73.1%)
Baseline HBI				
n	13	21	16	50
Mean (SD)	9.4 (4.84)	8.8 (3.34)	10.4 (5.02)	9.5 (4.30)
Median (min, max)	8.0 (5, 23)	7.0 (3, 15)	8.5 (5, 19)	8.0 (3, 23)
Baseline SES-CD				
n	12	21	16	49
Mean (SD)	9.4 (5.43)	8.5 (4.91)	14.4 (6.35)	10.7 (6.03)
Median (min, max)	9.5 (3, 21)	7.0 (3, 20)	15.0 (5, 24)	9.0 (3, 24)
Baseline C-reactive protein				
n	13	22	17	52
Mean (SD)	18.1 (16.75)	19.8 (29.56)	35.3 (49.40)	24.4 (35.36)
Median (min, max)	12.6 (1, 52)	7.4 (1, 113)	25.9 (0, 210)	10.6 (0, 210)
Baseline fecal calprotectin				
n	12	20	15	47
Mean (SD)	648.0 (791.44)	875.3 (954.21)	1170.5 (986.69)	911.5 (928.83)
Median (min, max)	247.0 (50, 2000)	584.5 (5, 4030)	800.0 (15, 3000)	504.0 (5, 4030)
Baseline PDAI				
n	13	22	16	51
Mean (SD)	0.0 (0.00)	0.9 (3.12)	2.7 (4.98)	1.2 (3.56)
Median (min, max)	0.0 (0, 0)	0.0 (0, 14)	0.0 (0, 17)	0.0 (0, 17)
Baseline EQ-5D-5L				
n	12	21	15	48
Mean (SD)	0.7 (0.30)	0.7 (0.25)	0.7 (0.39)	0.7 (0.31)
Median (min, max)	0.8 (0, 1)	0.8 (0, 1)	0.7 (− 1, 1)	0.8 (− 1, 1)
Baseline PRO-2				
n	11	22	13	46
Mean (SD)	89.3 (74.56)	113.5 (53.01)	135.6 (140.49)	113.9 (89.97)
Median (min, max)	67.0 (2, 278)	110.0 (21, 203)	76.0 (0, 520)	87.0 (0, 520)
Age at diagnosis: n (%)				
< 16 years	1 (7.7%)	3 (13.6%)	2 (11.8%)	6 (11.5%)
16–40 years	10 (76.9%)	14 (63.6%)	14 (82.4%)	38 (73.1%)
> 40 years	2 (15.4%)	5 (22.7%)	1 (5.9%)	8 (15.4%)
Location: n (%)				
Ileal	2 (15.4%)	4 (18.2%)	1 (5.9%)	7 (13.5%)
Colonic	5 (38.5%)	3 (13.6%)	7 (41.2%)	15 (28.8%)
Ileocolonic	4 (30.8%)	11 (50.0%)	9 (52.9%)	24 (46.2%)
Isolated upper disease	2 (15.4%)	4 (18.2%)	–	6 (11.5%)
Behavior: n (%)				
Nonstricturing, nonpenetrating	9 (69.2%)	12 (54.5%)	10 (58.8%)	31 (59.6%)
Stricturing	2 (15.4%)	7 (31.8%)	3 (17.6%)	12 (23.1%)
Penetrating	2 (15.4%)	3 (13.6%)	4 (23.5%)	9 (17.3%)
Perianal disease modifier: n (%)				
Yes	–	2 (9.1%)	2 (11.8%)	4 (7.7%)
No	13 (100.0%)	20 (90.9%)	15 (88.2%)	48 (92.3%)

*ITT* intent-to-treat analysis set, *N* number of randomized subjects, *n* number of subjects within each category, Harvey Bradshaw Index; *PDAI* perianal activity index, *PRO2* patient reported outcomes assessment; Immunomodulators, azathioprine, mercaptopurine or methotrexate.

### Efficacy

**Figure 1:**
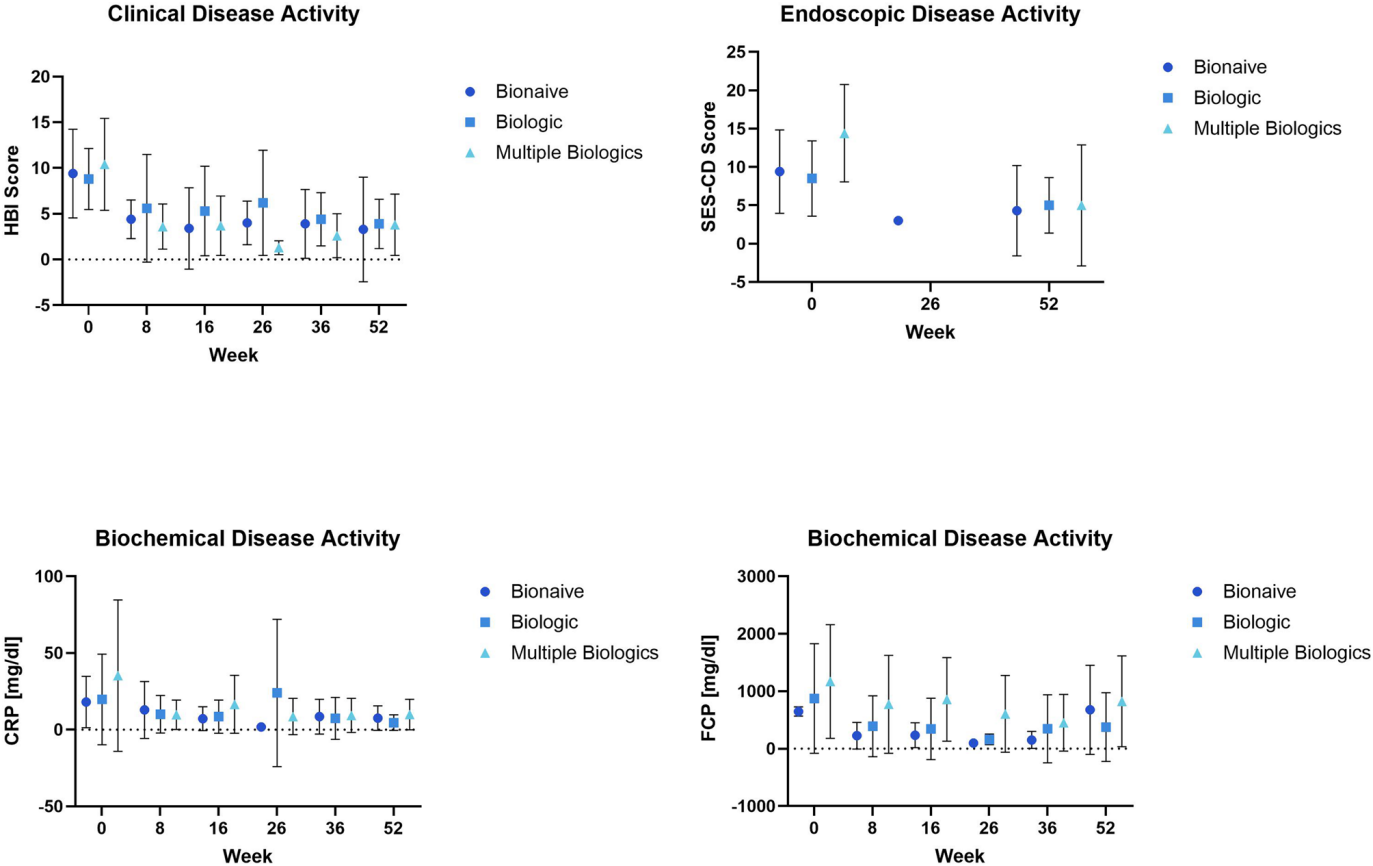
Disease activity throughout the study.

Overall, disease activity decreased throughout the course of the study among all groups. The course of key clinical, endoscopic and laboratory efficacy parameters over the course of the study is depicted in Fig. 1. For additional details please refer to the Supplementary Web Appendix Part 2 Web Tables 14.2.10–14.2.16 and 14.3.5.2.

#### Primary endpoint—combined clinical and endoscopic response

**Figure 2:**
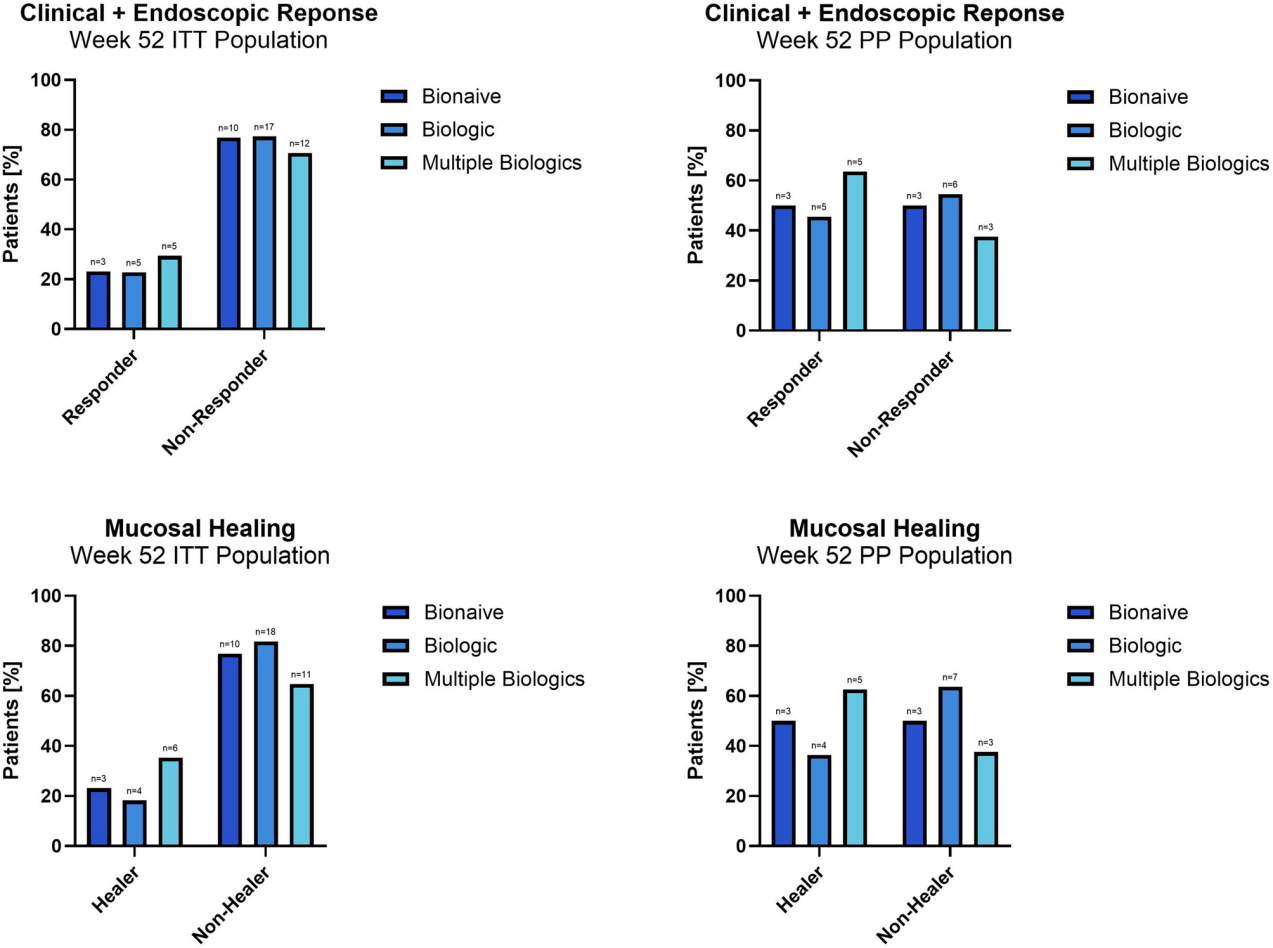
Top: Primary endpoint (combined clinical and endoscopic response at week 52—defined by an HBI score reduction ≥ 3 points from baseline and 50% SES-CD reduction from baseline) Bottom: mucosal healing (defined as the absence of mucosal ulcerations in any ileocolonic segment).

In the ITT population, 23.1%, 22.7% and 29.4% of patients in the bionaïve, biologic, and multiple biologics population achieved the primary endpoint, respectively. In the PP population, 50%, 45.5% and 62.5% of patients in the naïve, biologic, and multiple biologic population achieved the primary endpoint, respectively. There was no statistically significant difference between the ORs of all groups in both the ITT and PP analyses, respectively. Figure 2 top Web Appendix Part 2 Web Table 14.2.1.1.

#### Endoscopic response, remission and mucosal healing

**Figure 3:**
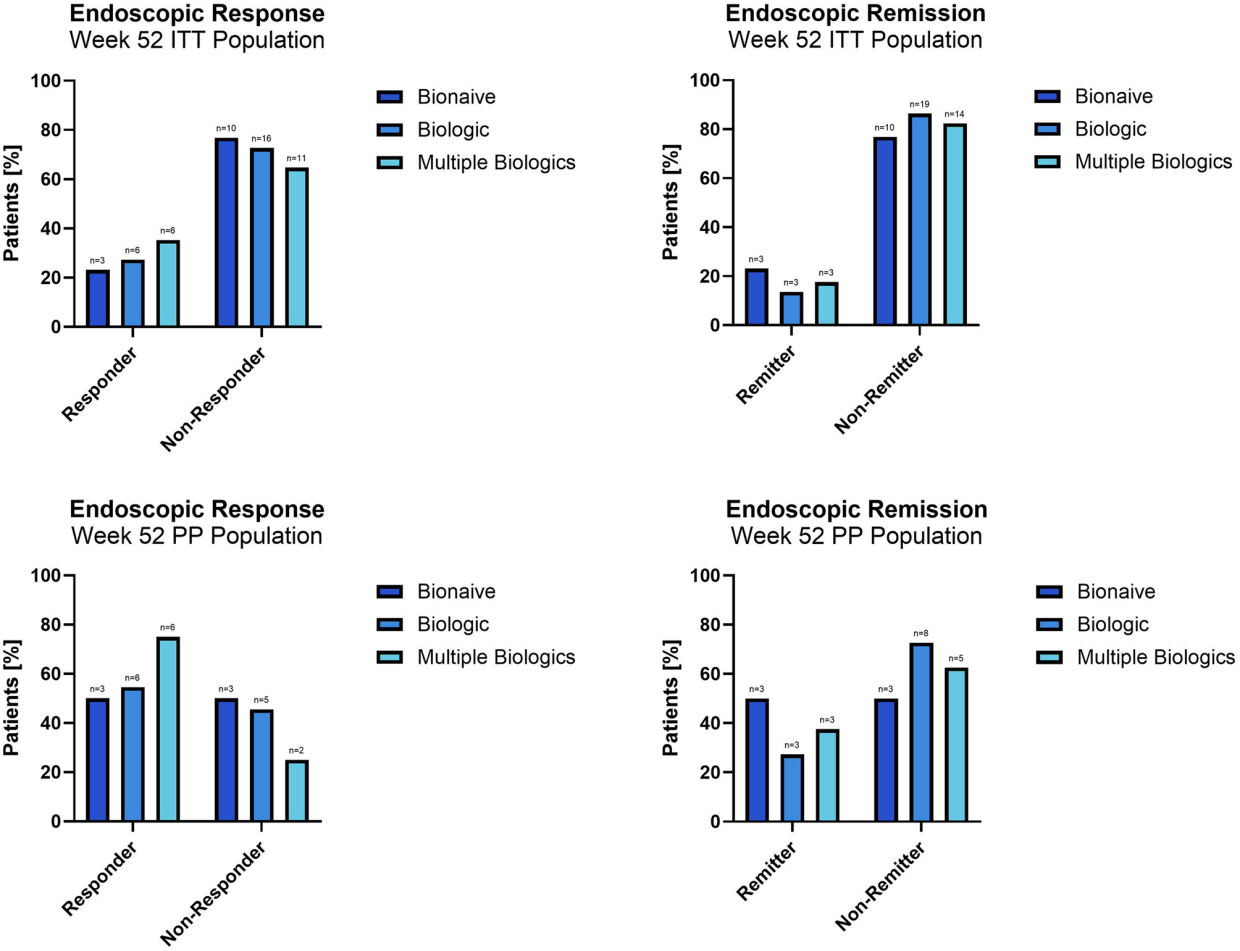
Endoscopic response (defined as 50% SES-CD reduction from baseline) and remission (defined as a SES-CD score ≤ 2) at Week 52.

In the ITT population, 23.1%, 27.3% and 35.3% of patients in the bionaïve, biologic, and multiple biologics groups achieved an endoscopic response, respectively. In the PP population, 50%, 54.5% and 75% of patients in the naïve, biologic, and multiple biologic population achieved an endoscopic response at week 52, respectively. Figure 3 Web Appendix Part 2 Web Tables 14.2.3.1 and 14.2.3.2.

In ITT population, 23.1%, 13.6% and 17.6% of patients in the bionaïve, biologic, and multiple biologics population achieved an endoscopic remission, respectively. In the PP population, 50%, 27.3% and 37.5% of patients in the naïve, biologic, and multiple biologic population achieved endoscopic remission at week 52, respectively. Figure 3 Web Appendix Part 2 Web Tables 14.2.4.1 and 14.2.4.2.

In ITT population, 23.1%, 18.2% and 35.3% of patients in the bionaïve, biologic, and multiple biologics population achieved mucosal healing, respectively. In the PP population, 50%, 36.4% and 62.5% of patients in the bionaïve, biologic, and multiple biologic population achieved mucosal healing at week 52, respectively. (Fig. 2 bottom) Web Appendix Part 2 Web Tables 14.2.5.1 and 14.2.5.2.

There was no statistically significant difference between the ORs of all groups regarding endoscopic response, endoscopic remission and mucosal healing in both the ITT and the PP analyses, respectively.

The SES-CD score decreased throughout the study. There were no statistically significant differences among the SES-CD change score LS means of all groups. Figure 1 Web Appendix Part 2 Web Table 14.2.11.

#### Clinical response, clinical remission, steroid free clinical remission and steroid sparing

**Figure 4:**
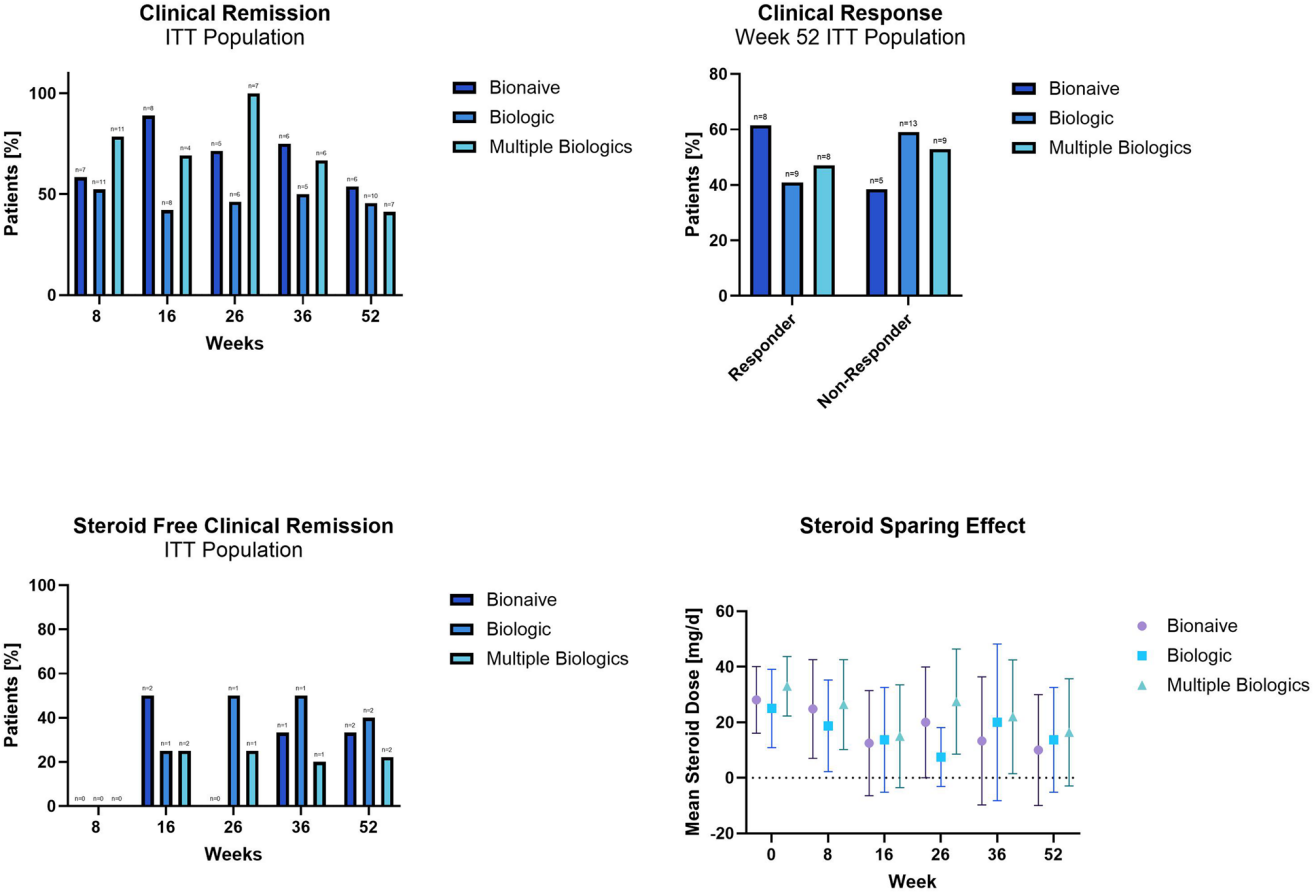
Clinical response (defined as HBI score reduction of ≥ 3 points), clinical remission (defined as HBI score of ≤ 4), steroid free clinical remission (defined as HBI of ≤ 4 and absence of concomitant steroid therapy in patients who were on it at baseline) and steroid sparing effect.

In the ITT population, 61.5%, 40.9% and 47.1% of patients in the bionaïve, biologic, and multiple biologics populations achieved a clinical response at week 52. There was no statistically significant difference between the OR of all groups, at week 52, respectively. Figure 4 Web Appendix Part 2 Web Table 14.2.2.

In the ITT population, 53.8%, 45.5% and 41.2% of patients in the bionaïve, biologic, and multiple biologics groups achieved a clinical remission at week 52. There was no statistically significant difference between the ORs of all groups, at all timepoints, respectively. Figure 4 Web Appendix Part 2 Web Table 14.2.6.

In the ITT population, 33.8%, 40% and 22.2% of patients in the bionaïve, biologic, and multiple biologics populations achieved steroid free clinical remission at week 52. At week 16 significantly more patients in the bionaïve vs. biologic or multiple biologic groups (p < 0.001) achieved steroid free clinical remission, but there was no statistically significant difference between the ORs of all groups, at all other timepoints, respectively. Overall, the steroid dose was successively reduced throughout the study. Figure 4 Web Appendix Part 2 Web Tables 14.2.7 and 14.3.1.11.

The HBI score decreased throughout the study. There were no statistically significant differences between HBI change score LS means of all groups. Figure 1 Web Appendix Part 2 Web Table 14.2.10.

#### Fistula response, remission and PDAI Score

There were no patients with fistulizing CD in the bionaïve group. In the ITT population, all patients in the biologic and multiple biologic populations achieved a fistula response at week 52. There was no statistically significant difference between the ORs of all groups, at week 52 and all timepoints, respectively.

In the ITT population, no patients in the biologic and 33.3% patients in the multiple biologics groups achieved fistula remission at week 52. A statistically significant difference (p < 0.001) between the ORs of the biologic vs. multiple biologic groups was noted at week 8, 16, and 52, respectively.

**Figure 5:**
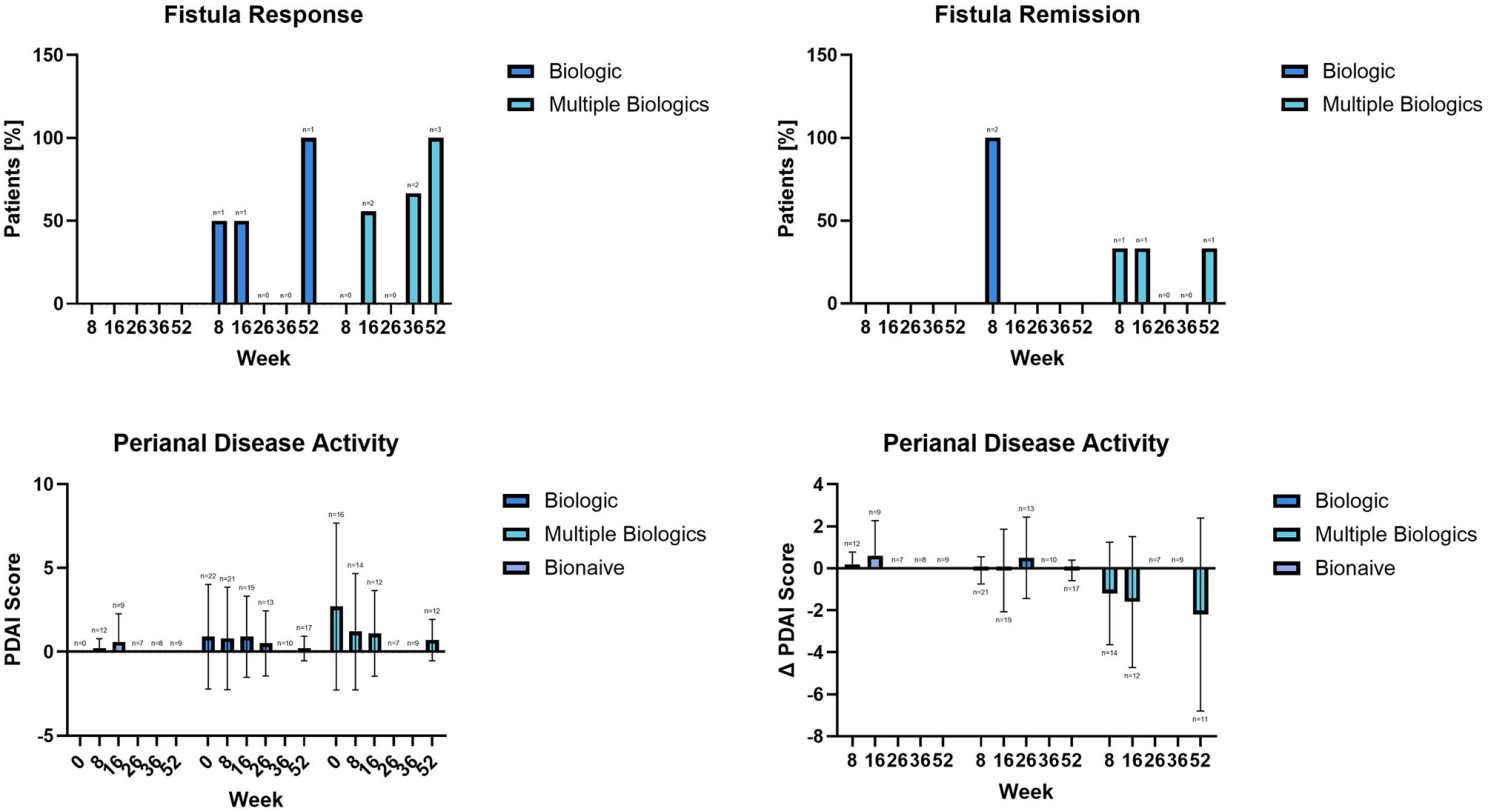
Fistula response (yes/no), fistula remission (yes/no) and perianal disease activity score (0 = remission, < 4 inactive disease not requiring therapy and ≥ 4 active disease requiring medical or surgical therapy).

The PDAI score decreased throughout the study, especially in the multiple biologic group that included most of the fistulizing CD patients at baseline. There were no statistically significant differences between PDAI change score LS means of groups. Figure 5 Web Appendix Part 2 Web Tables 14.2.8.1, 14.2.8.2, and 14.2.9.

#### Extraintestinal manifestations

**Figure 6:**
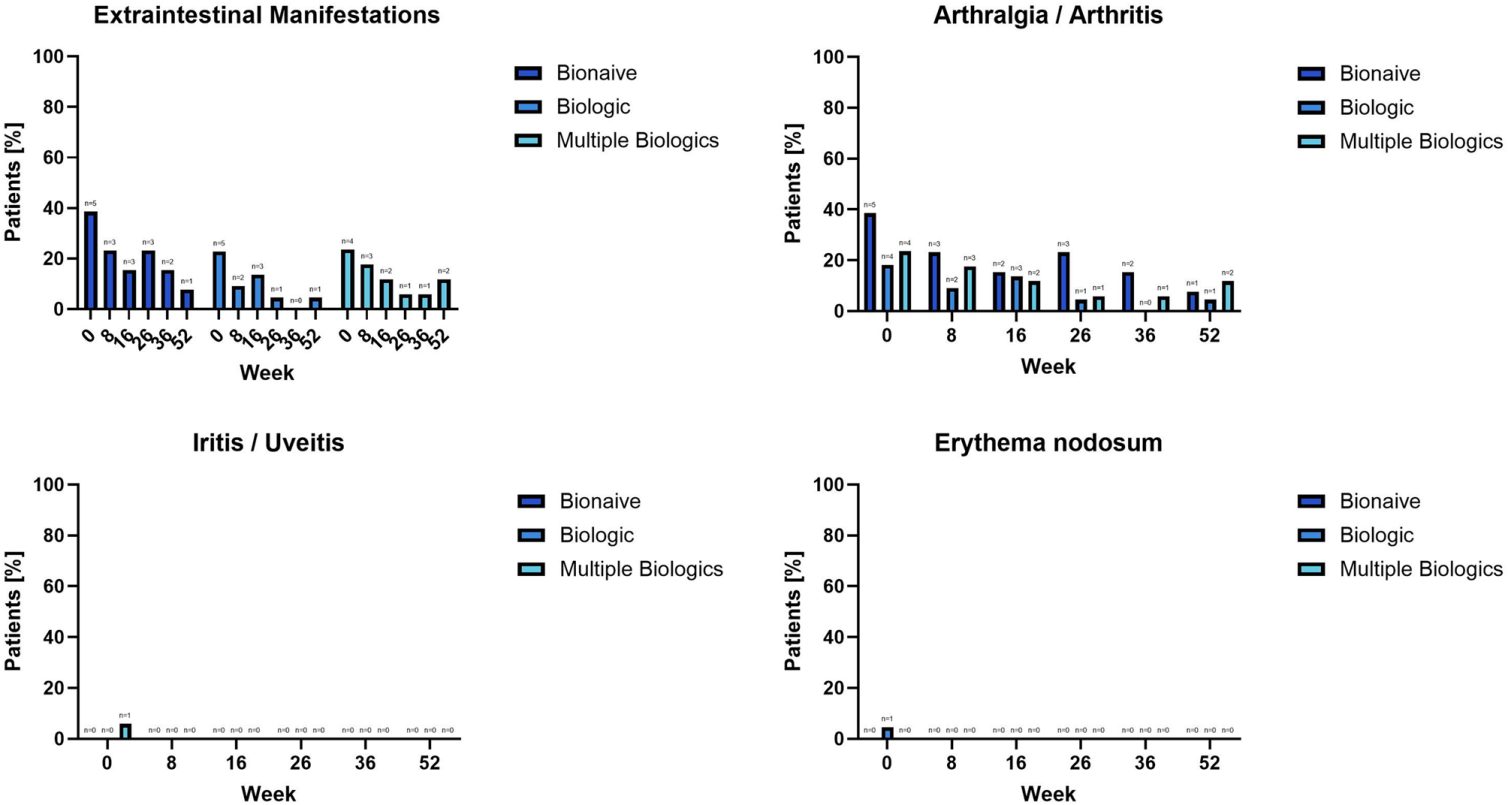
Extraintestinal manifestations Top left: cumulative data stratified by biologic exposure strata throughout the study. Top right, bottom left and right: arthralgia and arthritis, iritis/uveitis and erythema nodosum stratified by biologic exposure strata, respectively throughout the study.

Of the tracked extraintestinal manifestations (Supplementary Web Appendix Part 1: Web Table 2 Objectives and Endpoints) only arthralgia/arthritis, iritis/uveitis and erythema nodosum were present at baseline, with arthralgia/arthritis being the most common one across all strata, and no additional ones developed throughout the study. In the ITT population, 38.5%, 22.7% and 23.5% of patients in the bionaïve, biologic, and multiple biologics populations at baseline, which decreased to 7.7%, 4.5% and 11.8%, respectively at week 52. Figure 6 Web Appendix Part 2 Web Table 14.3.1.13.

#### Patient reported outcome (PRO-2)

**Figure 7:**
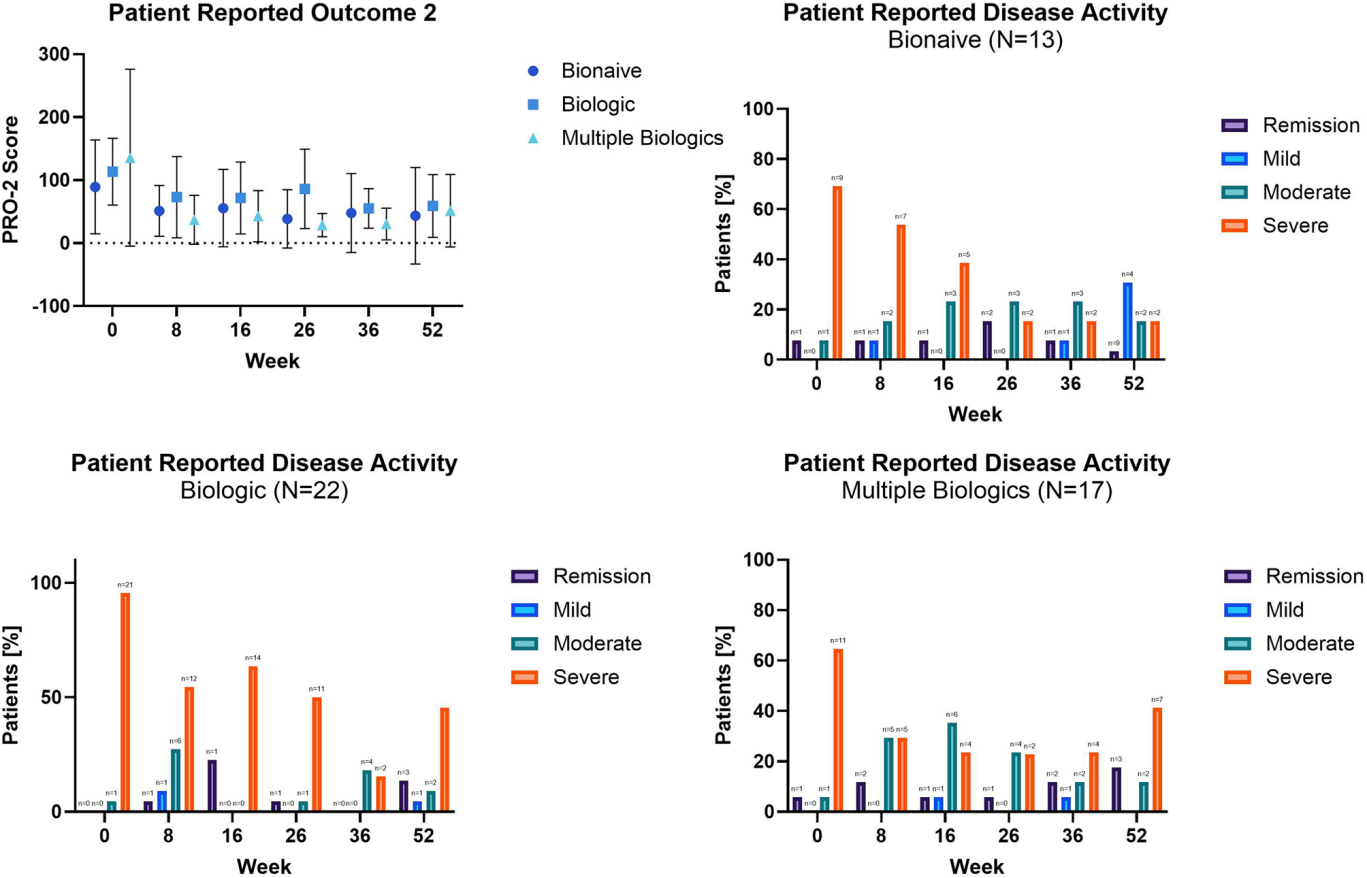
Reported outcome 2 (PRO-2) defined disease activity (PRO-2 remission 2 < 8, mild 8–13, moderate 14–34, severe ≥ 35 disease).

The PRO-2 score decreased throughout the study. Overall, fewer patients reported severe disease determined by PRO-2 activity at week 52 compared with baseline across all groups. There were no statistically significant differences between PRO-2 change score LS means of all groups. Figure 7 Web Appendix Part 2 Web Table 14.2.16.

#### Health related quality of life (EQ-5D-5L)

**Figure 8:**
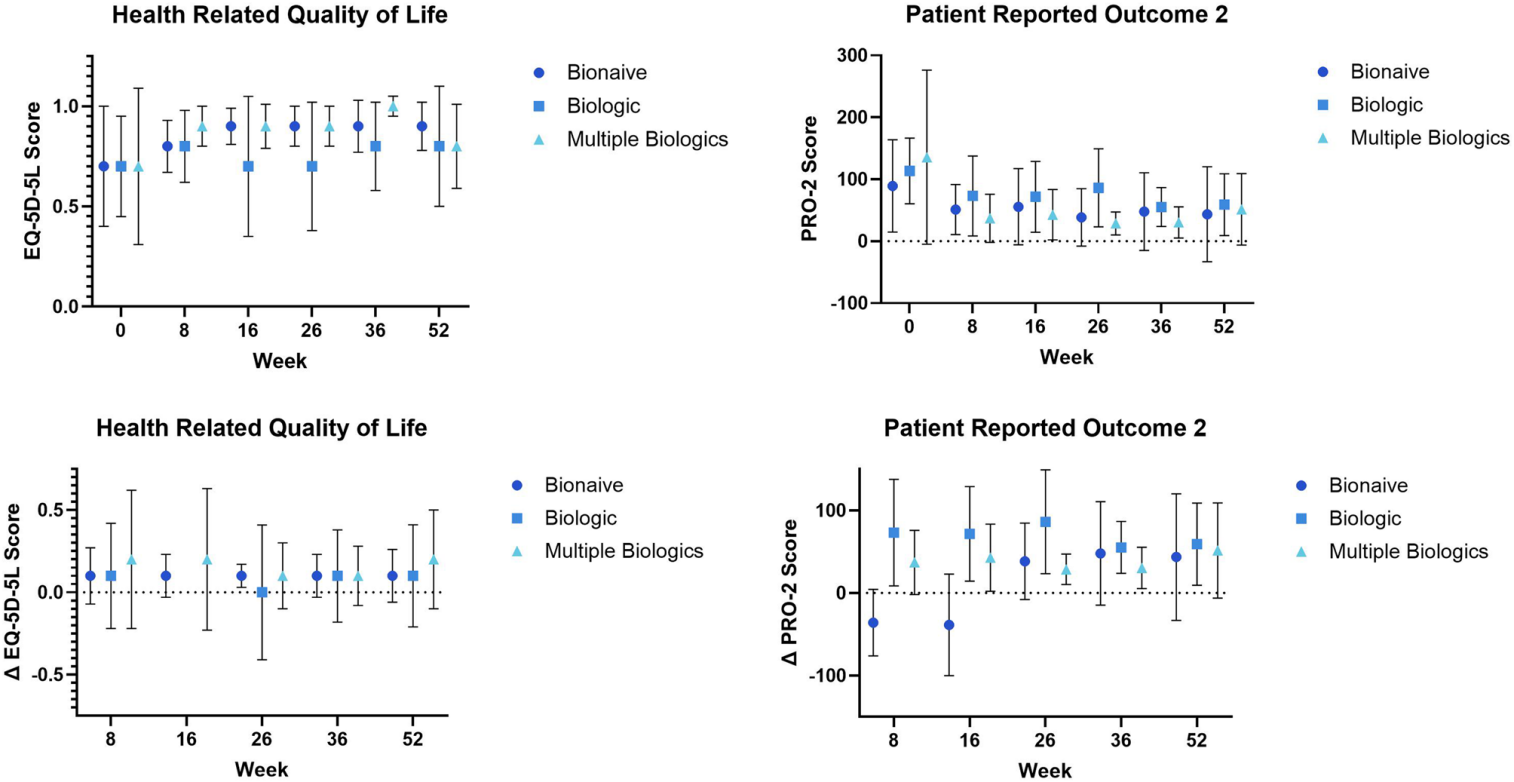
Health related quality of life EQ-5D-5L.

The EQ-5D-5L score increased throughout the study. There were no statistically significant differences between HBI change score LS means among of all groups. Figure 8 Web Appendix Part 2 Web Table 14.2.15.

#### Crohn’s disease related surgeries or hospitalizations

While 9.1% of patients in biologic group were hospitalized for CD [85.5 ± 40.3 (mean ± SD) days], none were admitted in the bionaïve or multiple biologics groups. 7.7%, 9.1% and 0% of patients in the bionaïve, biologic, and multiple biologic groups required CD related surgery after 374 ± 0 vs. 207.5 ± 212.84 (mean ± SD) days, respectively. Web Appendix Part 2 Tables 14.3.1.14, 14.3.1.15. There were no statistically significant differences between groups in the Kaplan Meier analysis. Web Appendix Part 2 Tables 14.4.1 and 14.4.2.

#### Other secondary and exploratory efficacy endpoints

Additional data on CBC, hemoglobin and albumin can be found in the Web Appendix Part 2. Table 14.3.5.2.

Only patient age remained statistically significantly [OR 95% CI 0.933 (0.873, 0.998) p = 0.043] associated with the primary outcome in the ITT population, while prior Crohn’s treatment strata, sex, age at diagnosis, disease phenotype, extraintestinal manifestations, CRP, FCP, albumin, EuroQoL-5D health questionnaire scores had no impact. Web Appendix Part 2. Table 14.4.3. No independent predictors for secondary outcomes could be identified (data not shown).

### Safety

#### Adverse events

Thirty-six patients (69.2%) across all groups (bionaïve 84.6%, biologic, 63.3%, multiple biologics 64.7%) experienced ≥ 1 treatment emergent adverse event (TEAE), in 8 (15.4%) cases rated as severe and in 5 (9.6%) across all groups (bionaïve 15.4%. biologic 9.1%, multiple biologics 5.9%) leading to discontinuation of ustekinumab, but no very severe events or deaths (Table 4).

**Table 4:** Summary of adverse events. See Supplementary Web Appendix Part 2 for additional details.

	Naive (N = 13)	Biologic (N = 22)	Multiple biologic (N = 17)
Total number of AEs	55	46	48
Total number of TEAEs	51	45	43
Subjects with ≥ 1 TEAE	11 (84.6%)	14 (63.6%)	11 (64.7%)
Subjects with an TEAE Leading to study drug discontinuation	2 (15.4%)	2 (9.1%)	1 (5.9%)
Total number of serious TEAEs	5	11	2
Subjects with ≥ 1 serious TEAE	3 (23.1%)	6 (27.3%)	2 (11.8%)
Subjects with serious TEAE leading to study drug discontinuation	2 (15.4%)	1 (4.5%)	1 (5.9%)
Number of subjects with ≥ 1 TEAE by maximum treatment relation			
Not related	5 (38.5%)	8 (36.4%)	5 (29.4%)
Unlikely	1 (7.7%)	–	1 (5.9%)
Possible	2 (15.4% )	4 (18.2%)	5 (29.4%)
Probable	1 (7.7%)	–	–
Definite	2 (15.4%)	2 (9.1%)	–
Number of subjects with ≥ 1 TEAE by maximum severity			
Mild	3 (23.1%)	2 (9.1%)	2 (11.8%)
Moderate	5 (38.5%)	9 (40.9%)	7 (41.2%)
Severe	3 (23.1%)	3 (13.6%)	2 (11.8%)
Very severe	–	–	–
Death	–	–	–

*N* number of randomized subjects, *n* number of subjects within each category, *TEAE* Treatment Emergent Adverse Events.

The TEAEs SOC and PT summary shows that gastrointestinal AEs (i.e., uncontrolled, or complicated Crohn’s disease) occurred more commonly in the bionaïve group. No very severe or deadly AEs occurred. Most TEAEs were judged as non-related. Patients in the multiple-biologic group—as expected—experienced more possibly related and more moderate TEAEs. Apart from the gastrointestinal disorders, infections were the second most frequently occurring TEAEs. Web Appendix Part 2 Tables 14.3.1.2–14.3.1.4, 14.3.1.6. Overall, we did not notice any new safety signals.

#### Infusion reactions

No infusion reaction TEAEs occurred. Web Appendix Part 2 Table 14.3.1.8.

#### Injection site reactions

Eight patients (15.5%) across all groups (bionaïve 15.4%, biologic 13.6%, multiple biologics 17.6%) experienced ≥ 1 injection site TEAE, most commonly edema followed by pyrexia and erythema. Web Appendix Part 2 Table 14.3.1.9.

#### Infections

Sixteen patients (31.7%) across all groups (bionaïve 38.5%, biologic 27.3%, multiple biologics 29.4%) experienced ≥ 1 infection TEAE, most commonly nasopharyngitis followed by urinary tract infections and COVID-19 infections. Web Appendix Part 2 Table 14.3.1.10.

## Discussion

First and foremost, it speaks to the spirit of our team and our commitment to the patients entrusted to our care, that we successfully finished a nationwide clinical research study in the middle of the COVID-19 pandemic^37^, where many non-pandemic related research projects were either cancelled or completely failed.

The strengths of our work include the national, multicentric approach, the definition of a combined clinical and endoscopic endpoint, inclusion of patients who failed multiple biologics and/or immunomodulators, recording and central (investigator independent) reading of the endoscopy videos, investigation of fistula healing, capturing the effect on important extraintestinal manifestations and the comprehensive efficacy and safety analyses usually not seen in real world studies.

Up to one third vs. two thirds (ITT vs. PP population), respectively, achieved the combined primary endpoint (combined clinical and endoscopic response) in our cohort. Interestingly, prior exposure to multiple biologics (not just anti-TNFα as in the ustekinumab randomized registration trial program^18,19^) did not a have negative impact on the primary outcome. These patients did numerically better although this was not statistically significant. This unexpected observation is likely related to sample size disparities among the different strata and does not challenge our general experience that biologic drugs generally work better when given first line.

Our biologic exposed patients achieved slightly higher clinical remission rates than re-randomized responders in the UNITI-1 randomized controlled trial (stratified for primary or secondary loss of response to anti-TNFα cohort) at week 44 (90 mg q8w) in the IM-UNITI study^18,19^. This is remarkable, since we used a classic treat-through-design, included patients who in some cases failed immunomodulators or biologics or both, compared with the UNITI program where biologic failures were limited to anti-TNFα and strictly separated from conventional treatment failures in UNITI-1 and UNITI-2 cohorts and re-randomization of responders (with the resulting tendency to overestimate the therapeutic effect size) occurred. However, this comparison should still be interpreted with caution, given other trial design differences like assessment timepoints (week 44 vs. 52) or outcome assessment instruments (HBI vs. CDAI).

Roughly one third vs. two thirds (ITT vs. PP population) of our patients achieved an endoscopic response and mucosal healing, respectively. Prior biologic exposure had no statistically significant impact on these outcomes. Both endoscopic outcomes were much better (mucosal healing two times better) than in the ustekinumab development program, where both outcomes were only assessed in pooled subgroup analyses of UNITI-1, UNITI-2 and IM-UNITI in patients with ulcerations at baseline^20^. To date, no randomized controlled trial with ustekinumab focused on mucosal healing was conducted.

All four perianal fistula patients achieved a fistula response and more than one third in the multiple biologics group achieved fistula remission. Our fistula healing data are similar to the ustekinumab development program, where it was defined as complete resolution from baseline. A pooled subgroup analysis of the CERTIFI, UNITI-1 and IM-UNITI trials in abstract format^38^ is available where there was only a numerical, but not statistically significant difference against placebo. To date, no placebo controlled randomized controlled trial with ustekinumab focused on fistula healing was conducted.

Ustekinumab also impressively improved the most common extraintestinal manifestations such as arthropathy, uveitis and erythema nodosum. Overall, the fraction of patients with extraintestinal manifestations decreased by more than one third.

Other secondary indicators of inflammation like CRP, FCP, PRO-2 and quality of life like the EQ-5D-5L improved throughout the study in line with the results of the composite primary endpoint and its components underscoring the plausibility and validity of our findings. Moreover, there were no statistically significant differences between groups in the Kaplan Meier analysis of CD related surgeries or hospitalizations among the three different prior biologic exposure strata.

Overall, we did not identify any new safety signals which is comparable to the ustekinumab development program extension study in which ustekinumab responders of the UNITI-1, UNITI-2 and IM-UNITI program were re-randomized and did not detect new safety signals over 96 weeks^39^.

Recently, the real-world effectiveness and safety of ustekinumab in the treatment of Crohn’s Disease was published by the North American SUCCESS^40^ consortium giving the opportunity to view our findings with a more appropriate cohort.

The SUCCESS cohort included 1,113 patients, almost all of which (90%) were exposed to anti-TNFα prior and achieved cumulative clinical-, steroid free- clinical and endoscopic remission rates of 40%, 32% and 39% as opposed to 45.5% and 41.2% vs. 40% and 22.2%, 13.6% / 27.3% (ITT vs. PP) and 17.6%/37.5% (ITT vs. PPP) in our biologic and multiple biologics groups, at week 52, respectively. Overall, these data confirm our findings. The discrepancy in the endoscopy data sets likely results from the high attrition rate reflected in the discordant ITT vs. PP population results of our study. Like in the SUCCESS consortium biologic-naive patients did achieve higher rates of clinical and endoscopic remission.

Our study has important limitations. The negative impact of the unprecedented COVID global pandemic most likely greatly contributed to the higher than previously experienced attrition rate of the study.

An unprecedented challenge was the conduction of a research study during a global pandemic with all the imposed restrictions and limitations including but not limited to sick study personnel, sick patients, sick patient relatives, limited pharmacy, laboratory in- and outpatient hospital services [clinic access, lab availability, endoscopy availability], mandatory adherence to institutional policies (i.e. shutdown of all research activities) and repeated government curfews. With recruitment severely impaired and an unusually high attrition rate, fewer patients than planned were enrolled and completed the study. Thus, we were unable to complete the originally planned statistical modeling to identify independent predictors of mucosal healing and had to default to mostly basic statistics and reporting.

As a non-interventional RWE study with application of medication according to its national label, it did not include a control group. Drug application according to the national label also precluded any dose escalation or reinductions, that are now known to be effective and common practice to improve outcomes and extend medication use^41,42^.

In summary, we demonstrated, when prescribed within its German label, ustekinumab effectively and safely induces mucosal- and fistula healing, alleviates many extraintestinal manifestations and improves quality of life in patients with Crohn’s disease. Ustekinumab’s overall effectiveness was better than in the original ustekinumab development program and comparable with the large SUCCESS consortium real world experience.

## Supplementary Information

Supplementary Information 1.

Supplementary Information 2.

## Data Availability

All data generated or analysed during this study are included in this published article [and its supplementary information files].
