# Supplementary material for: Induction and maintenance of mucosal healing in Crohn’s disease with ustekinumab in clinical practice across all care levels in Germany (MUCUS)
Source: Sci Rep. 2024 Sep 3;14:20502. doi: 10.1038/s41598-024-70241-9 (PMC11371836; doi:10.1038/s41598-024-70241-9)
Supplement: Supplementary file 1 — Supplementary Information 1. [file 41598_2024_70241_MOESM1_ESM.pdf]

Supplementary Web Appendix – Part 1

Web Table 1: Time and Event Schedule 2
Web Table 2: Objectives and Endpoints 6

Web Table 1: Time and Event Schedule

		Induction		Maintenance (Weeks 16 to 48):						
	Screen[a]	Week 0 ST	Week 8	Week 16[x]	Week 26[c]	Week 36	Disease Flare Visit[d]	Week 52	ET[e]	Safety FU[f]
**Study Procedure**[b]										
Informed consent (ICF)[g]	X									
Demographics, Montreal Classification and EIM	X							X		
Vitals incl. Weight (BMI)	X	X	X	X	X	X	X	X	X	X
Height	X									
Medical history and Prior Therapy	X									
Inclusion/exclusion criteria[h]	X									
Concomitant medication	X	X	X	X	X	X	X	X	X	X
QuantiFERON-TB Gold In-Tube test[i,r]	X									
Stool studies to evaluate for enteric pathogens[kj]	X									
Training on HBI, PRO-2 and EQ5D5L completion	X									
Serum pregnancy test	X									
	Ustekinumab Administration									
Administer IV ustekinumab (induction)[l,x]		X								
Stratification[y]		X								
Administer SC ustekinumab[m]			X	X[op]	X[op]	X[op]		X[op]		
Dosing frequency decision[o]				X			X			
Drug accountability		X	X	X	X	X		X	X	
	Safety Assessments									
Detailed physical examination	X	X	X	X	X	X	X	X	X	X
TB evaluation[s]/other infection assessment	X	X	X	X	X	X	X	X	X	X
CBC, CRP, albumin, fecal	X	X	X	X	X	X	X	X	X	X
Macroscopic immunogenicity assessments		X	X	X	X	X		X	X	
HIV, Hepatitis B, Hepatitis C	X									
Urine Pregnancy test[q]		X	X	X	X	X		X		
Begin corticosteroid taper[x]				X[x]						
Ustekinumab injection-site evaluation		X	X	X[o]	X	X	X	X	X	
Hospitalization?		X	X	X	X	X	X	X	X	X
Surgery or Intervention (e.g. balloon dilation)?		X	X	X	X	X	X	X	X	X

Adverse Events Documentation (MedDRA)		X	X	X	X	X	X	X	X	X
	Efficacy Assessments									
HBI and EIM assessments[t]		X[v]	X	X	X	X	X	X	X	
Quality of Life EQ-5D-5L		X	X	X	X	X	X	X	X	
PRO-2		X	X	X	X	X	X	X	X	
Stool sample (FC)[w]		X	X	X	X	X	X	X	X	
Video ileocolonoscopy and SES-CD	X				(X)			X	X	
Fistula assessment (PDAI)		X	X	X	X	X	X	X	X	

Abbreviations: ET = early termination; FU = follow-up; IV = intravenous; S = Screening ST = Stratification; HBI = Harvey Bradshaw Index; EIM = Extraintestinal Manifestations; PRO-2 = Patient Related Outcome 2; EQ5D5L = European Quality of Life Score (EuroQoL)

Footnotes:

a. The Screening period will be up to 2 weeks. A prior endoscopy may be used only if obtained within 3 months prior to baseline (Week 0), in which case the prior endoscopy must be centrally read again and SES-CD calculated based on this second, centralized read-out.

b. The visit windows will be ±10 days for all visits. At ustekinumab injection/assessment visits, all assessments are to be completed prior to ustekinumab administration, unless otherwise specified. It is recommended that patient-reported outcome (PRO) assessments be completed first.

c. Assessment visits: the week of each visit will depend on initial maintenance dosing frequency regimen and subsequent dosing adjustments during the study.

d. Disease flare assessments can be performed at any time between Week 16 and Week 52 in case of clinical worsening reported by the subject, consistent with disease flare in the investigator’s judgment. Clinical assessments in case of disease flare will be at the investigator’s discretion. Information on assessments of disease flare and on the reasons for dosing frequency changes will be documented in the eCRF.

e. Subjects who discontinue ustekinumab treatment before Week 52 will have Early Termination (ET) assessments as close as possible to the time of discontinuation unless consent is withdrawn. ET assessments should include ileocolonoscopy.

f. All subjects will have a final safety follow-up visit 16 weeks after the last administration of ustekinumab within the study. If a subject refuses to come for an onsite visit, a telephone visit can be performed to collect information on AEs, infection assessment and hospitalization.

g. Must be signed before first study-related activity.

h. Minimum criteria for the availability of documentation supporting the eligibility criteria as described, Source Documentation. Check clinical status again before first dose of study medication.

i. To be performed at a local laboratory. In sites where QuantiFERON-TB Gold test is not used, a tuberculin skin test is additionally required. The QuantiFERON-TB Gold In-Tube test is not required at screening for subjects with a history of latent TB and appropriate treatment as described in the Inclusion Criteria.

j. Stool studies for enteric pathogens will be performed at the local laboratory and must include a stool culture and *Clostridium difficile* toxin assay.

k. Induction treatment at Week 0: ustekinumab IV (weight-based dosing approximately 6 mg/kg) to be administered over a period of not less than 1 hour.

l. Subjects who do not achieve HBI response (Harvey Bradshaw index score reduction ≥ 3 points) at Week 16 will discontinue from the study.

m. Ustekinumab SC (90 mg prefilled syringe). After Week 8, subjects who have been trained how to self-inject may self-administer. Information documented in the e CRF will include date of administration, self-administration (yes/no) and whether administration was complete based on the returned syringe.

n. Only subjects initially assigned to 8-weekly maintenance treatment will receive ustekinumab at Week 16 (subjects initially assigned to 12-weekly treatment will have the next ustekinumab injection at Week 20).

o. From Week 16, subjects' ustekinumab maintenance treatment will be according to the German label for ustekinumab. After Week 16, subjects who lose response during 12-weekly SC dosing may benefit from an adjustment to 8-weekly maintenance treatment in compliance with the German label. Subjects may subsequently be dosed every 8 weeks or every 12 weeks according to clinical judgment. Subjects on an 8-weekly regimen at the time of disease flare, will not be able to dose adjust further and will leave the study if they would not benefit from continuing study treatment in the investigator's judgment.

p. Temperature, pulse/heart rate, blood pressure, height, weight, and calculated BMI. At Week 0, vital signs assessed prior to infusion, approximately every 30 minutes during infusion, and twice (approximately 30-minute intervals) after the completion of infusion.

q. Must be performed prior to each ustekinumab administration in females of childbearing potential. Additional pregnancy tests may be performed, as determined necessary by the investigator, or required by local regulation.

r. If TB is suspected at any time during the study, a chest radiograph, QuantiFERON-TB Gold, or tuberculin skin test In-Tube test should be performed.

s. Hematology, CRP, albumin, fecal calprotectin to be tested on all visits. They do not need to be repeated at Week 0 if the screening tests were done ≤2 weeks previously.

t. Ileocolonoscopic examinations and HBI assessment will be scheduled to avoid an impact on HBI data. For example, if ileocolonoscopy is performed on the day of the visit, the 7 days prior to the initiation of the colonoscopy preparation should be used to calculate the HBI score for the visit. Ileocolonoscopies will be assessed at a central facility. Ileocolonoscopy examination at week 26 is

desired but not mandatory.

v. For calculation of HBI at Week 0, the hematocrit value obtained during screening will be used.

w. Analyzed at a local laboratory. For FC testing, study site personnel will remind subjects of the need to provide stool samples.

x. For subjects receiving oral corticosteroids who had a response at Week 16 (HBI Responders) corticosteroid tapering is mandatory. Corticosteroid tapering can be initiated from Week 8 in subjects already demonstrating response to ustekinumab treatment (see 12-week prednisolone tapering calendar in Attachment 12).

To ensure a balanced, unbiased real world cohort subjects will be stratified 1:1:1 at baseline according to being naïve to biologics, prior exposure to 1 or 2 or more biologics for treatment of Crohn’s disease.

Web Table 2: Objectives and Endpoints

Objectives	Endpoints
**Primary**	
To evaluate the real-world effectiveness of ustekinumab (routine care) as combined clinical and endoscopic response in week 52.	Clinical response (Harvey Bradshaw index score reduction ≥ 3 points from baseline) AND endoscopic response (50% reduction of SES-CD from baseline)
**Objectives**	**Endpoints**
**Secondary**	
To examine the robustness of the primary endpoint analysis, sensitivity analyses of the primary endpoint will be conducted.	Clinical response (Harvey Bradshaw index score reduction ≥ 3 points from baseline) AND endoscopic response (50% reduction of SES-CD from baseline)
To evaluate the real-world effectiveness (routine care) in achieving and maintaining endoscopic remission.	Endoscopic remission defined as a SES-CD score ≤2 at Week 26 (optional) and Week 52
To evaluate the real-world effectiveness (routine care) in improving endoscopic and clinical indexes.	Changes in score from baseline for the Harvey Bradshaw index and SES-CD questionnaire at Weeks 26 and 52.

To evaluate the real-world effectiveness (routine care) in achieving and maintaining of mucosal healing.	Mucosal healing defined as the complete absence of mucosal ulcerations in any ileocolonic segment at Week 26 and Week 52
To evaluate the real-world effectiveness (routine care) in achieving and maintaining clinical remission.	Clinical remission is defined by a Harvey Bradshaw index score of ≤ 4. It will be assessed at Weeks 8, 16, 26, 36, 52.
To evaluate the real-world effectiveness (routine care) in achieving and maintaining steroid free clinical remission.	Steroid free clinical remission is defined by a Harvey Bradshaw index score of ≤ 4and the absence of concomitant steroid therapy in subjects who were on steroids at baseline. It will be assessed at Weeks 8, 16, 26, 36, 52.
To evaluate the real-world effectiveness (routine care) in achieving and maintaining clinical response.	Clinical response is defined as a Harvey Bradshaw index score reduction ≥ 3 points. It will be assessed at Weeks 8, 16, 26, 36, 52.
To evaluate the real-world effectiveness (routine care) in controlling perianal disease activity in subjects with active fistulizing Crohn's disease at baseline	The PDAI will be used. Fistula response (yes / no) and remission (yes / no) will be assessed compared with baseline reading. 0 = remission. <4 (inactive disease not requiring therapy) ≥4 (active disease requiring medical or surgical therapy) Done on Weeks 0, 8, 16, 26, 36, 52
To evaluate the real-world effectiveness (routine care) on serum CRP and albumin, as well as fecal calprotectin levels.	Serum CRP<br>Serum albumin<br>FCP on Weeks 0, 8, 16, 26, 36, 52
To evaluate the real-world effectiveness (routine care) on health- related quality of life (QoL) and patient-reported outcomes (PRO-2 soft stool frequency over past week abdominal pain over past week)	Changes from baseline for European quality of life 5 dimensions 5 level (EQ-5D-5L) on Weeks 0, 8, 16, 26, 36, 52<br>PRO-2: remission, mild, mode rate, severe (<8, 8–13, 14–34, >35) on Weeks 0, 8, 16, 26, 36, 52
To evaluate the real-world effectiveness (routine care) on extraintestinal manifestations and/or phenotype.	Phenotype according to Montreal classification will be recorded on Weeks 0, 8, 16, 26, 36, 52.<br>Presence and activity of extraintestinal manifestations will be recorded on Weeks 0, 8, 16, 26, 36, 52

To describe the safety of real world ustekinumab use at every study visit	Adverse events according to MedDRA terminology<br>Clinical laboratory data<br>Vital signs<br>Physical examination findings on Weeks 0, 8, 16, 26, 36, 52.
To assess the steroid sparing effect of ustekinumab	Record steroid tapering dose at all visits with concomitant medications
**Exploratory**	
To explore the benefit of routine care ustekinumab on Crohn's disease-related hospitalizations and surgeries.	Crohn’s disease related hospitalizations<br>Crohn’s disease related surgery
To explore the impact of various demographic and baseline characteristics on the effectiveness of routine care ustekinumab on Crohn's primary and secondary outcomes.	Statistical modeling
