# Supplementary material for: Induction and maintenance of mucosal healing in Crohn’s disease with ustekinumab in clinical practice across all care levels in Germany (MUCUS)
Source: Sci Rep. 2024 Sep 3;14:20502. doi: 10.1038/s41598-024-70241-9 (PMC11371836; doi:10.1038/s41598-024-70241-9)
Supplement: Supplementary file 2 — Supplementary Information 2. [file 41598_2024_70241_MOESM2_ESM.pdf]

Table of Contents

Item	Page Number
14.1.1 - Subject Disposition	3
14.1.2.1 - Summary of Demographic and Baseline Disease Characteristics: Safety Population	4
14.1.2.2 - Summary of Demographic and Baseline Disease Characteristics: ITT Population	7
14.1.3 - Summary of Treatment Compliances	10
14.1.4 - Summary of Medical History by SOC and PT	11
14.1.5 - Summary of Prior Medications	17
14.1.6 - Summary of Concomitant Medications	19
14.1.7 - Summary of Protocol Deviations	26
14.2.1.1 - Summary of Clinical Response and Endoscopic Response at Week 52: ITT Population	27
14.2.1.2 - Summary of Clinical Response and Endoscopic Response at Week 52: PP Population	28
14.2.1.3 - Summary of Clinical Response and Endoscopic Response at Week 52 Gender Subgroup Analysis: ITT Population	29
14.2.2 - Summary of Clinical Response at Week 52: ITT Population	30
14.2.3.1 - Summary of Endoscopic Response at Week 52: ITT Population	31
14.2.3.2 - Summary of Endoscopic Response at Week 52: Per-Protocol Analysis Set	32
14.2.4.1 - Summary of Endoscopic Remission at Week 26 and 52: ITT Population	33
14.2.4.2 - Summary of Endoscopic Remission at Week 26 and 52: Per-Protocol Analysis Set	34
14.2.5.1 - Summary of Mucosal Healing Rate at Week 26 and 52: ITT Population	35
14.2.5.2 - Summary of Mucosal Healing Rate at Week 26 and 52: Per-Protocol Analysis Set	36
14.2.6 - Summary of Clinical Remission at Week 8, 16, 26, 36, 52: ITT Population	37
14.2.7 - Summary of Steroid Free Clinical Remission at Week 8, 16, 26, 36, 52	39
14.2.8.1 - Summary of Fistula Response at Week 8, 16, 26, 36, 52	41
14.2.8.2 - Summary of Fistula Remission at Week 8, 16, 26, 36, 52	43
14.2.9 - Summary of Mean Change in PDAI Score from Baseline at Week 8, 16, 26, 36, 52	45
14.2.10 - Summary of Mean Change in HBI Index from Baseline at Week 8, 16, 26, 36, 52	48
14.2.11 - Summary of Mean Change in SES-CD Score from Baseline at Week 26 and 52	51
14.2.12 - Summary of Mean Change hs-CRP Concentration from Baseline at week 8, 16, 26, 36, 52	53
14.2.13 - Summary of Mean Change Albumin Concentration from Baseline at week 8, 16, 26, 36, 52	56
14.2.14 - Summary of Mean Change Fecal Calprotectin Concentration from Baseline at week 8, 16, 26, 36, 52	59
14.2.15 - Summary of Mean Change in EQ-5D-5L from Baseline at Weeks 8, 16, 26, 36, 52	62
14.2.16 - Summary of Mean Change in PRO-2 from Baseline at Weeks 8, 16, 26, 36, 52	65
14.2.17 - Summary of PRO-2 Remission at Weeks 0, 8, 16, 26, 36, 52	68
14.3.1.1 - Overall Summary of AEs	70
14.3.1.2 - Summary of TEAEs by SOC and PT	71
14.3.1.3 - Summary of TEAEs by SOC, PT and Max Severity	75
14.3.1.4 - Summary of Severe (CTCAE Grade >= 3) TEAEs by SOC and PT	88
14.3.1.5 - Summary of Related TEAEs by SOC and PT	89

Table of Contents

Item	Page Number
14.3.1.6 - Summary of Serious TEAEs by SOC and PT	90
14.3.1.7 - Summary of Related Serious TEAEs by SOC and PT	91
14.3.1.8 - Summary of Infusion Reaction TEAEs by SOC and PT	92
14.3.1.9 - Summary of Injection-Site Reaction TEAEs by SOC and PT	93
14.3.1.10 - Summary of Infection TEAEs by SOC and PT	94
14.3.1.11 - Summary of Steroid Tapering at Week 0, 8 ,16, 26, 36, 52	95
14.3.1.12 - Summary of Montreal Classification Phenotype at Week 0, 8 ,16, 26, 36, 52	96
14.3.1.13 - Summary of Extraintestinal Manifestations at Week 0, 8 ,16, 26, 36, 52	100
14.3.1.14 - Summary of Crohn's Related Hospitilizations	102
14.3.1.15 - Summary of Crohn's Related Surgery	103
14.3.5.1 - Summary of Vital Signs	104
14.3.5.2 - Summary of Clinical Laboratory Test Results	135
14.4.1 - Time to CD-Related Hospitalization	201
14.4.2 - Time to CD-Related Surgery	202
14.4.3 - Summary of Clinical Response and Endoscopic Response Exploratory Analysis at Week 52: ITT Population	203

Table 14.1.1: Subject Disposition

	Naive (N=13)	Biologic (N=22)	Multiple Biologic (N=17)	Total (N=52)
Study Disposition: n (%)				
Screened				79
Screen Failed				27
Randomized	13	22	17	52
Discontinued from Study	3 (23.1%)	8 (36.4%)	7 (41.2%)	18 (34.6%)
Completed Study	10 (76.9%)	14 (63.6%)	10 (58.8%)	34 (65.4%)
Eligible for Safety Analysis Set	13 (100.0%)	22 (100.0%)	17 (100.0%)	52 (100.0%)
Eligible for ITT Analysis Set	13 (100.0%)	22 (100.0%)	17 (100.0%)	52 (100.0%)
Eligible for Per-Protocol Analysis Set	6 (46.2%)	11 (50.0%)	8 (47.1%)	25 (48.1%)
Reasons for Discontinuation: n (%)	3 (23.1%)	8 (36.4%)	7 (41.2%)	18 (34.6%)
Adverse Event	1 (7.7%)	1 (4.5%)	1 (5.9%)	3 (5.8%)
Lost to Follow-Up	-	-	3 (17.6%)	3 (5.8%)
Non-HBI Responder	1 (7.7%)	3 (13.6%)	1 (5.9%)	5 (9.6%)
Other	-	1 (4.5%)	1 (5.9%)	2 (3.8%)
Pregnancy	-	1 (4.5%)	-	1 (1.9%)
Subject Decision	1 (7.7%)	2 (9.1%)	1 (5.9%)	4 (7.7%)

Abbreviations: ITT, Intent-to-Treat Analysis set; N, number of randomized subjects; n, number of subjects within each category; HBI, Harvey Bradshaw Index.

Program Name: MUCUS-Shell1-V01
Output Location: .\Output\Shared\MUCUS-TFLS-Final-27MAR24-V01.pdf

Table 14.1.2.1: Summary of Demographic and Baseline Disease Characteristics: Safety Population

	Naive (N=13)	Biologic (N=22)	Multiple Biologic (N=17)	Total (N=52)
Gender: n(%)				
Female	8 (61.5%)	14 (63.6%)	6 (35.3%)	28 (53.8%)
Male	5 (38.5%)	8 (36.4%)	11 (64.7%)	24 (46.2%)
Age: (yrs.)				
n	13	22	17	52
Mean (SD)	39.0 (15.25)	40.3 (12.01)	39.5 (10.43)	39.7 (12.19)
Median (min, max)	39.2 (20, 64)	36.7 (21, 74)	42.4 (21, 57)	39.3 (20, 74)
Race: n(%)				
White	13 (100.0%)	22 (100.0%)	17 (100.0%)	52 (100.0%)
Prior Crohn's Surgery: n(%)				
Yes	-	3 (13.6%)	4 (23.5%)	7 (13.5%)
No	13 (100.0%)	19 (86.4%)	13 (76.5%)	45 (86.5%)
Concomitant Monotherapy Immunomodulators: n(%)				
Yes	-	2 (9.1%)	1 (5.9%)	3 (5.8%)
No	13 (100.0%)	20 (90.9%)	16 (94.1%)	49 (94.2%)
Concomitant Combination Therapy Immunomodulators: n(%)				
Yes	-	-	-	-
No	13 (100.0%)	22 (100.0%)	17 (100.0%)	52 (100.0%)
Fistulizing Crohn’s: n(%)				
Yes	-	2 (9.1%)	5 (29.4%)	7 (13.5%)
No	13 (100.0%)	20 (90.9%)	12 (70.6%)	45 (86.5%)
Weight: (kg)				
n	12	20	15	47
Mean (SD)	73.0 (17.01)	72.8 (14.68)	72.3 (16.09)	72.7 (15.40)
Median (min, max)	79.5 (39, 93)	68.0 (51, 103)	72.0 (47, 106)	72.0 (39, 106)
Height: (cm)				
n	12	20	16	48
Mean (SD)	173.3 (14.89)	171.3 (10.61)	173.5 (7.80)	172.5 (10.86)
Median (min, max)	169.0 (147, 195)	169.0 (157, 192)	172.0 (161, 195)	171.0 (147, 195)
Body Mass Index: (kg/m²)				

Abbreviations: ITT, Intent-to-Treat Analysis set; N, number of randomized subjects; n, number of subjects within each category; HBI, Harvey Bradshaw Index; PDAI, perianal activity index; PRO-2, patient reported outcomes assesment; Immunomodulators, azathioprine, mercaptopurine or methotrexate.

Program Name: MUCUS-Shell2-V01
Output Location: .\Output\Shared\MUCUS-TFLS-Final-27MAR24-V01.pdf

Table 14.1.2.1: Summary of Demographic and Baseline Disease Characteristics: Safety Population

	Naive (N=13)	Biologic (N=22)	Multiple Biologic (N=17)	Total (N=52)
n	12	20	15	47
Mean (SD)	24.2 (4.93)	24.7 (3.59)	23.8 (4.46)	24.3 (4.16)
Median (min, max)	23.5 (18, 34)	23.7 (19, 33)	22.4 (17, 32)	23.3 (17, 34)
Extraintestinal Manifestations: n(%)				
Yes	5 (38.5%)	5 (22.7%)	4 (23.5%)	14 (26.9%)
No	8 (61.5%)	17 (77.3%)	13 (76.5%)	38 (73.1%)
Baseline HBI:				
n	13	21	16	50
Mean (SD)	9.4 (4.84)	8.8 (3.34)	10.4 (5.02)	9.5 (4.30)
Median (min, max)	8.0 (5, 23)	7.0 (3, 15)	8.5 (5, 19)	8.0 (3, 23)
Baseline SES-CD:				
n	12	21	16	49
Mean (SD)	9.4 (5.43)	8.5 (4.91)	14.4 (6.35)	10.7 (6.03)
Median (min, max)	9.5 (3, 21)	7.0 (3, 20)	15.0 (5, 24)	9.0 (3, 24)
Baseline C-reactive Protein:				
n	13	22	17	52
Mean (SD)	18.1 (16.75)	19.8 (29.56)	35.3 (49.40)	24.4 (35.36)
Median (min, max)	12.6 (1, 52)	7.4 (1, 113)	25.9 (0, 210)	10.6 (0, 210)
Baseline Fecal CalProtectin:				
n	12	20	15	47
Mean (SD)	648.0 (791.44)	875.3 (954.21)	1170.5 (986.69)	911.5 (928.83)
Median (min, max)	247.0 (50, 2000)	584.5 (5, 4030)	800.0 (15, 3000)	504.0 (5, 4030)
Baseline PDAI:				
n	13	22	16	51
Mean (SD)	0.0 (0.00)	0.9 (3.12)	2.7 (4.98)	1.2 (3.56)
Median (min, max)	0.0 (0, 0)	0.0 (0, 14)	0.0 (0, 17)	0.0 (0, 17)
Baseline EQ-5D-5L:				
n	12	21	15	48
Mean (SD)	0.7 (0.30)	0.7 (0.25)	0.7 (0.39)	0.7 (0.31)
Median (min, max)	0.8 (0, 1)	0.8 (0, 1)	0.7 (-1, 1)	0.8 (-1, 1)

Abbreviations: ITT, Intent-to-Treat Analysis set; N, number of randomized subjects; n, number of subjects within each category; HBI, Harvey Bradshaw Index; PDAI, perianal activity index; PRO-2, patient reported outcomes assesment; Immunomodulators, azathioprine, mercaptopurine or methotrexate.

Program Name: MUCUS-Shell2-V01
Output Location: .\Output\Shared\MUCUS-TFLS-Final-27MAR24-V01.pdf

Table 14.1.2.1: Summary of Demographic and Baseline Disease Characteristics: Safety Population

	Naive (N=13)	Biologic (N=22)	Multiple Biologic (N=17)	Total (N=52)
Baseline PRO-2:				
n	11	22	13	46
Mean (SD)	89.3 (74.56)	113.5 (53.01)	135.6 (140.49)	113.9 (89.97)
Median (min, max)	67.0 (2, 278)	110.0 (21, 203)	76.0 (0, 520)	87.0 (0, 520)
Age at Diagnosis: n(%)				
<16yrs	1 (7.7%)	3 (13.6%)	2 (11.8%)	6 (11.5%)
16-40yrs	10 (76.9%)	14 (63.6%)	14 (82.4%)	38 (73.1%)
>40yrs	2 (15.4%)	5 (22.7%)	1 (5.9%)	8 (15.4%)
Location: n(%)				
Ileal	2 (15.4%)	4 (18.2%)	1 (5.9%)	7 (13.5%)
Colonic	5 (38.5%)	3 (13.6%)	7 (41.2%)	15 (28.8%)
Ileocolonic	4 (30.8%)	11 (50.0%)	9 (52.9%)	24 (46.2%)
Isolated Upper disease	2 (15.4%)	4 (18.2%)	-	6 (11.5%)
Behaviour: n(%)				
Nonstricturing, Nonpenetrating	9 (69.2%)	12 (54.5%)	10 (58.8%)	31 (59.6%)
Stricturing	2 (15.4%)	7 (31.8%)	3 (17.6%)	12 (23.1%)
Penetrating	2 (15.4%)	3 (13.6%)	4 (23.5%)	9 (17.3%)
Perianal diasease modifer: n(%)				
Yes	-	2 (9.1%)	2 (11.8%)	4 (7.7%)
No	13 (100.0%)	20 (90.9%)	15 (88.2%)	48 (92.3%)

Abbreviations: ITT, Intent-to-Treat Analysis set; N, number of randomized subjects; n, number of subjects within each category; HBI, Harvey Bradshaw Index; PDAI, perianal activity index; PRO-2, patient reported outcomes assesment; Immunomodulators, azathioprine, mercaptopurine or methotrexate.

Program Name: MUCUS-Shell2-V01
Output Location: .\Output\Shared\MUCUS-TFLS-Final-27MAR24-V01.pdf

Table 14.1.2.2: Summary of Demographic and Baseline Disease Characteristics: ITT Population

	Naive (N=13)	Biologic (N=22)	Multiple Biologic (N=17)	Total (N=52)
Gender: n(%)				
Female	8 (61.5%)	14 (63.6%)	6 (35.3%)	28 (53.8%)
Male	5 (38.5%)	8 (36.4%)	11 (64.7%)	24 (46.2%)
Age: (yrs.)				
n	13	22	17	52
Mean (SD)	39.0 (15.25)	40.3 (12.01)	39.5 (10.43)	39.7 (12.19)
Median (min, max)	39.2 (20, 64)	36.7 (21, 74)	42.4 (21, 57)	39.3 (20, 74)
Race: n(%)				
White	13 (100.0%)	22 (100.0%)	17 (100.0%)	52 (100.0%)
Prior Crohn's Surgery: n(%)				
Yes	-	3 (13.6%)	4 (23.5%)	7 (13.5%)
No	13 (100.0%)	19 (86.4%)	13 (76.5%)	45 (86.5%)
Concomitant Monotherapy Immunomodulators: n(%)				
Yes	-	2 (9.1%)	1 (5.9%)	3 (5.8%)
No	13 (100.0%)	20 (90.9%)	16 (94.1%)	49 (94.2%)
Concomitant Combination Therapy Immunomodulators: n(%)				
Yes	-	-	-	-
No	13 (100.0%)	22 (100.0%)	17 (100.0%)	52 (100.0%)
Fistulizing Crohn’s: n(%)				
Yes	-	2 (9.1%)	5 (29.4%)	7 (13.5%)
No	13 (100.0%)	20 (90.9%)	12 (70.6%)	45 (86.5%)
Weight: (kg)				
n	12	20	15	47
Mean (SD)	73.0 (17.01)	72.8 (14.68)	72.3 (16.09)	72.7 (15.40)
Median (min, max)	79.5 (39, 93)	68.0 (51, 103)	72.0 (47, 106)	72.0 (39, 106)
Height: (cm)				
n	12	20	16	48
Mean (SD)	173.3 (14.89)	171.3 (10.61)	173.5 (7.80)	172.5 (10.86)
Median (min, max)	169.0 (147, 195)	169.0 (157, 192)	172.0 (161, 195)	171.0 (147, 195)
Body Mass Index: (kg/㎡)				

Abbreviations: N, number of randomized subjects; n, number of subjects within each category;HBI, Harvey Bradshaw Index; PDAI, perianal activity index; PRO-2, patient reported outcomes assesment; Immunomodulators, azathioprine, mercaptopurine or methotrexate.

Program Name: MUCUS-Shell2-V01
Output Location: .\Output\Shared\MUCUS-TFLS-Final-27MAR24-V01.pdf

Table 14.1.2.2: Summary of Demographic and Baseline Disease Characteristics: ITT Population

	Naive (N=13)	Biologic (N=22)	Multiple Biologic (N=17)	Total (N=52)
n	12	20	15	47
Mean (SD)	24.2 (4.93)	24.7 (3.59)	23.8 (4.46)	24.3 (4.16)
Median (min, max)	23.5 (18, 34)	23.7 (19, 33)	22.4 (17, 32)	23.3 (17, 34)
Extraintestinal Manifestations: n(%)				
Yes	5 (38.5%)	5 (22.7%)	4 (23.5%)	14 (26.9%)
No	8 (61.5%)	17 (77.3%)	13 (76.5%)	38 (73.1%)
Baseline HBI:				
n	13	21	16	50
Mean (SD)	9.4 (4.84)	8.8 (3.34)	10.4 (5.02)	9.5 (4.30)
Median (min, max)	8.0 (5, 23)	7.0 (3, 15)	8.5 (5, 19)	8.0 (3, 23)
Baseline SES-CD:				
n	12	21	16	49
Mean (SD)	9.4 (5.43)	8.5 (4.91)	14.4 (6.35)	10.7 (6.03)
Median (min, max)	9.5 (3, 21)	7.0 (3, 20)	15.0 (5, 24)	9.0 (3, 24)
Baseline C-reactive Protein:				
n	13	22	17	52
Mean (SD)	18.1 (16.75)	19.8 (29.56)	35.3 (49.40)	24.4 (35.36)
Median (min, max)	12.6 (1, 52)	7.4 (1, 113)	25.9 (0, 210)	10.6 (0, 210)
Baseline Fecal CalProtectin:				
n	12	20	15	47
Mean (SD)	648.0 (791.44)	875.3 (954.21)	1170.5 (986.69)	911.5 (928.83)
Median (min, max)	247.0 (50, 2000)	584.5 (5, 4030)	800.0 (15, 3000)	504.0 (5, 4030)
Baseline PDAI:				
n	13	22	16	51
Mean (SD)	0.0 (0.00)	0.9 (3.12)	2.7 (4.98)	1.2 (3.56)
Median (min, max)	0.0 (0, 0)	0.0 (0, 14)	0.0 (0, 17)	0.0 (0, 17)
Baseline EQ-5D-5L:				
n	12	21	15	48
Mean (SD)	0.7 (0.30)	0.7 (0.25)	0.7 (0.39)	0.7 (0.31)
Median (min, max)	0.8 (0, 1)	0.8 (0, 1)	0.7 (-1, 1)	0.8 (-1, 1)

Abbreviations: N, number of randomized subjects; n, number of subjects within each category;HBI, Harvey Bradshaw Index; PDAI, perianal activity index; PRO-2, patient reported outcomes assesment; Immunomodulators, azathioprine, mercaptopurine or methotrexate.

Program Name: MUCUS-Shell2-V01
Output Location: .\Output\Shared\MUCUS-TFLS-Final-27MAR24-V01.pdf

Table 14.1.2.2: Summary of Demographic and Baseline Disease Characteristics: ITT Population

	Naive (N=13)	Biologic (N=22)	Multiple Biologic (N=17)	Total (N=52)
Baseline PRO-2:				
n	11	22	13	46
Mean (SD)	89.3 (74.56)	113.5 (53.01)	135.6 (140.49)	113.9 (89.97)
Median (min, max)	67.0 (2, 278)	110.0 (21, 203)	76.0 (0, 520)	87.0 (0, 520)
Age at Diagnosis: n(%)				
<16yrs	1 (7.7%)	3 (13.6%)	2 (11.8%)	6 (11.5%)
16-40yrs	10 (76.9%)	14 (63.6%)	14 (82.4%)	38 (73.1%)
>40yrs	2 (15.4%)	5 (22.7%)	1 (5.9%)	8 (15.4%)
Location: n(%)				
Ileal	2 (15.4%)	4 (18.2%)	1 (5.9%)	7 (13.5%)
Colonic	5 (38.5%)	3 (13.6%)	7 (41.2%)	15 (28.8%)
Ileocolonic	4 (30.8%)	11 (50.0%)	9 (52.9%)	24 (46.2%)
Isolated Upper disease	2 (15.4%)	4 (18.2%)	-	6 (11.5%)
Behaviour: n(%)				
Nonstricturing, Nonpenetrating	9 (69.2%)	12 (54.5%)	10 (58.8%)	31 (59.6%)
Stricturing	2 (15.4%)	7 (31.8%)	3 (17.6%)	12 (23.1%)
Penetrating	2 (15.4%)	3 (13.6%)	4 (23.5%)	9 (17.3%)
Perianal diasease modifer: n(%)				
Yes	-	2 (9.1%)	2 (11.8%)	4 (7.7%)
No	13 (100.0%)	20 (90.9%)	15 (88.2%)	48 (92.3%)

Abbreviations: N, number of randomized subjects; n, number of subjects within each category;HBI, Harvey Bradshaw Index; PDAI, perianal activity index; PRO-2, patient reported outcomes assesment; Immunomodulators, azathioprine, mercaptopurine or methotrexate.

Program Name: MUCUS-Shell2-V01
Output Location: .\Output\Shared\MUCUS-TFLS-Final-27MAR24-V01.pdf

Table 14.1.3: Summary of Treatment Compliances

	Naive (N=13)	Biologic (N=22)	Multiple Biologic (N=17)
Number of Injections Administered:			
N	13	22	17
Mean (SD)	6.8 (2.35)	5.9 (2.55)	6.1 (2.99)
Median (Min, Max)	7.0 (1, 9)	6.0 (1, 10)	6.0 (1, 11)
Number of Subjects who received > 6 injections	10 (76.9%)	8 (36.4%)	8 (47.1%)
Number of Subjects who received 6 injections	1 (7.7%)	5 (22.7%)	3 (17.6%)
Number of Subjects who received < 6 injections	2 (15.4%)	9 (40.9%)	6 (35.3%)

Abbreviations: N, number of randomized subjects; n, number of subjects within each category; SD, Standard Deviation; min, minimum; max, maximum.

Program Name: MUCUS-Shell3-V01
Output Location: .\Output\Shared\MUCUS-TFLS-Final-27MAR24-V01.pdf

Table 14.1.4: Summary of Medical History by SOC and PT

	Naive<br>(N=13)	Biologic<br>(N=22)	Multiple Biologic<br>(N=17)
Subjects with >= 1 Medical History Event	13(100.0%)	22(100.0%)	17(100.0%)
Blood and lymphatic system disorders	3(23.1%)	3(13.6%)	2(11.8%)
Abdominal lymphadenopathy	-	-	1(5.9%)
Anaemia	1(7.7%)	-	-
Iron deficiency anaemia	2(15.4%)	2(9.1%)	-
Lymphadenopathy	-	-	1(5.9%)
Spherocytic anaemia	-	1(4.5%)	-
Cardiac disorders	2(15.4%)	3(13.6%)	1(5.9%)
Acute myocardial infarction	-	-	1(5.9%)
Arrhythmia	1(7.7%)	-	-
Coronary artery disease	-	-	1(5.9%)
Myocardial infarction	1(7.7%)	-	-
Sinus tachycardia	-	2(9.1%)	-
Tachyarrhythmia	-	1(4.5%)	-
Congenital, familial and genetic disorders	1(7.7%)	1(4.5%)	-
Antithrombin III deficiency	-	1(4.5%)	-
Fructose-1,6-bisphosphatase deficiency	1(7.7%)	-	-
Ear and labyrinth disorders	-	-	1(5.9%)
Vertigo	-	-	1(5.9%)
Endocrine disorders	2(15.4%)	1(4.5%)	-
Basedow's disease	1(7.7%)	-	-
Hypothyroidism	1(7.7%)	1(4.5%)	-
Gastrointestinal disorders	13(100.0%)	22(100.0%)	17(100.0%)
Anal fissure	1(7.7%)	1(4.5%)	-
Anal fistula	-	1(4.5%)	1(5.9%)
Chronic gastritis	-	-	1(5.9%)
Crohn's disease	13(100.0%)	22(100.0%)	17(100.0%)
Diarrhoea	-	1(4.5%)	-
Diverticulum intestinal	-	1(4.5%)	-
Duodenal perforation	-	1(4.5%)	-

Abbreviations: N, number of randomized subjects; n, number of subjects within each category; SOC, System Organ Class; PT, Preferred Term.

Program Name: MUCUS-Shell10a-V01
Output Location: .\Output\Shared\MUCUS-TFLS-Final-27MAR24-V01.pdf

Table 14.1.4: Summary of Medical History by SOC and PT

	Naive (N=13)	Biologic (N=22)	Multiple Biologic (N=17)
Duodenal ulcer	-	-	1(5.9%)
Dyspepsia	-	-	1(5.9%)
Enterocutaneous fistula	-	1(4.5%)	-
Flatulence	1(7.7%)	-	-
Gastric ulcer	-	1(4.5%)	-
Gastritis	-	2(9.1%)	-
Gastritis erosive	1(7.7%)	-	-
Gastrooesophageal reflux disease	2(15.4%)	-	1(5.9%)
Haemorrhoids	-	1(4.5%)	-
Hyperchlorhydria	-	-	1(5.9%)
Ileal stenosis	1(7.7%)	-	-
Inguinal hernia	-	1(4.5%)	-
Intestinal dilatation	-	1(4.5%)	-
Intestinal stenosis	-	1(4.5%)	-
Large intestine perforation	-	1(4.5%)	-
Pancreatitis	-	-	2(11.8%)
Rectal stenosis	-	-	1(5.9%)
Short-bowel syndrome	-	1(4.5%)	-
Terminal ileitis	-	1(4.5%)	-
Umbilical hernia	-	1(4.5%)	-
General disorders and administration site conditions	-	2(9.1%)	1(5.9%)
Hernia	-	1(4.5%)	-
Pain	-	-	1(5.9%)
Stenosis	-	1(4.5%)	-
Hepatobiliary disorders	1(7.7%)	2(9.1%)	1(5.9%)
Cholelithiasis	1(7.7%)	1(4.5%)	1(5.9%)
Cholestasis	-	1(4.5%)	-
Hepatic steatosis	1(7.7%)	-	-
Immune system disorders	-	3(13.6%)	3(17.6%)
Allergy to animal	-	-	1(5.9%)

Abbreviations: N, number of randomized subjects; n, number of subjects within each category; SOC, System Organ Class; PT, Preferred Term.

Program Name: MUCUS-Shell10a-V01
Output Location: .\Output\Shared\MUCUS-TFLS-Final-27MAR24-V01.pdf

Table 14.1.4: Summary of Medical History by SOC and PT

	Naive (N=13)	Biologic (N=22)	Multiple Biologic (N=17)
Allergy to metals	-	1(4.5%)	1(5.9%)
Drug hypersensitivity	-	-	1(5.9%)
Food allergy	-	1(4.5%)	-
Mite allergy	-	-	1(5.9%)
Seasonal allergy	-	2(9.1%)	2(11.8%)
Infections and infestations	1(7.7%)	3(13.6%)	3(17.6%)
Herpes zoster	-	1(4.5%)	1(5.9%)
Latent tuberculosis	-	1(4.5%)	-
Oesophageal candidiasis	-	1(4.5%)	1(5.9%)
Pilonidal disease	-	-	1(5.9%)
Staphylococcal infection	-	1(4.5%)	-
Urinary tract infection	1(7.7%)	-	-
Injury, poisoning and procedural complications	1(7.7%)	1(4.5%)	2(11.8%)
Anastomotic stenosis	-	1(4.5%)	-
Clavicle fracture	-	-	1(5.9%)
Incisional hernia	1(7.7%)	-	-
Joint dislocation	-	-	1(5.9%)
Investigations	-	1(4.5%)	1(5.9%)
Aldolase abnormal	-	-	1(5.9%)
Interferon gamma release assay positive	-	1(4.5%)	-
Metabolism and nutrition disorders	7(53.8%)	3(13.6%)	6(35.3%)
Cachexia	-	-	1(5.9%)
Folate deficiency	-	-	1(5.9%)
Fructose intolerance	1(7.7%)	-	-
Histamine intolerance	-	-	1(5.9%)
Hypercholesterolaemia	1(7.7%)	1(4.5%)	-
Hyperlipidaemia	-	1(4.5%)	1(5.9%)
Iron deficiency	1(7.7%)	-	2(11.8%)
Lactose intolerance	3(23.1%)	-	1(5.9%)
Obesity	1(7.7%)	-	-

Abbreviations: N, number of randomized subjects; n, number of subjects within each category; SOC, System Organ Class; PT, Preferred Term.

Program Name: MUCUS-Shell10a-V01
Output Location: .\Output\Shared\MUCUS-TFLS-Final-27MAR24-V01.pdf

Table 14.1.4: Summary of Medical History by SOC and PT

	Naive (N=13)	Biologic (N=22)	Multiple Biologic (N=17)
Protein deficiency	-	-	1(5.9%)
Type 2 diabetes mellitus	-	-	1(5.9%)
Vitamin B12 deficiency	3(23.1%)	1(4.5%)	1(5.9%)
Vitamin D deficiency	2(15.4%)	-	3(17.6%)
Zinc deficiency	1(7.7%)	-	-
Musculoskeletal and connective tissue disorders	1(7.7%)	4(18.2%)	1(5.9%)
Arthralgia	-	1(4.5%)	-
Axial spondyloarthritis	1(7.7%)	-	-
Intervertebral disc protrusion	-	1(4.5%)	-
Osteopenia	-	1(4.5%)	-
Sacroiliitis	-	1(4.5%)	-
Spinal pain	-	-	1(5.9%)
Spondylolisthesis	1(7.7%)	-	-
Neoplasms benign, malignant and unspecified (incl cysts and polyps)	1(7.7%)	3(13.6%)	1(5.9%)
Basal cell carcinoma	1(7.7%)	-	-
Colon cancer	-	-	1(5.9%)
Fibroma	-	1(4.5%)	-
Infected neoplasm	-	1(4.5%)	-
Neoplasm	-	1(4.5%)	-
Nervous system disorders	2(15.4%)	1(4.5%)	-
Complex regional pain syndrome	-	1(4.5%)	-
Headache	2(15.4%)	-	-
Migraine	-	1(4.5%)	-
Psychiatric disorders	2(15.4%)	1(4.5%)	2(11.8%)
Depression	2(15.4%)	1(4.5%)	2(11.8%)
Post-traumatic stress disorder	-	1(4.5%)	-
Renal and urinary disorders	1(7.7%)	1(4.5%)	2(11.8%)
Acute kidney injury	1(7.7%)	-	-
Nephrolithiasis	-	1(4.5%)	1(5.9%)
Renal colic	-	-	1(5.9%)

Abbreviations: N, number of randomized subjects; n, number of subjects within each category; SOC, System Organ Class; PT, Preferred Term.

Program Name: MUCUS-Shell10a-V01
Output Location: .\Output\Shared\MUCUS-TFLS-Final-27MAR24-V01.pdf

Table 14.1.4: Summary of Medical History by SOC and PT

	Naive (N=13)	Biologic (N=22)	Multiple Biologic (N=17)
Renal cyst	-	-	1(5.9%)
Reproductive system and breast disorders	2(15.4%)	-	1(5.9%)
Balanoposthitis	-	-	1(5.9%)
Female genital tract fistula	1(7.7%)	-	-
Rectocele	1(7.7%)	-	-
Respiratory, thoracic and mediastinal disorders	2(15.4%)	1(4.5%)	2(11.8%)
Asthma	1(7.7%)	-	1(5.9%)
Chronic obstructive pulmonary disease	-	-	1(5.9%)
Nasal polyps	-	-	1(5.9%)
Pulmonary embolism	-	1(4.5%)	-
Rhinitis allergic	1(7.7%)	-	-
Skin and subcutaneous tissue disorders	2(15.4%)	5(22.7%)	3(17.6%)
Alopecia	-	1(4.5%)	-
Hidradenitis	-	-	1(5.9%)
Neurodermatitis	-	-	1(5.9%)
Psoriasis	1(7.7%)	4(18.2%)	1(5.9%)
Pyoderma gangrenosum	1(7.7%)	-	1(5.9%)
Solar urticaria	-	1(4.5%)	-
Surgical and medical procedures	3(23.1%)	12(54.5%)	8(47.1%)
Abscess drainage	-	-	1(5.9%)
Anal fissure excision	1(7.7%)	1(4.5%)	-
Anal skin tag excision	-	1(4.5%)	-
Appendicectomy	-	4(18.2%)	2(11.8%)
Caesarean section	-	-	1(5.9%)
Catheter management	-	-	1(5.9%)
Cervical conisation	-	1(4.5%)	-
Cholecystectomy	1(7.7%)	1(4.5%)	1(5.9%)
Colectomy	-	2(9.1%)	1(5.9%)
Colon operation	-	-	1(5.9%)
Colorectostomy	-	1(4.5%)	-

Abbreviations: N, number of randomized subjects; n, number of subjects within each category; SOC, System Organ Class; PT, Preferred Term.

Program Name: MUCUS-Shell10a-V01
Output Location: .\Output\Shared\MUCUS-TFLS-Final-27MAR24-V01.pdf

Table 14.1.4: Summary of Medical History by SOC and PT

	Naive (N=13)	Biologic (N=22)	Multiple Biologic (N=17)
Coronary arterial stent insertion	-	-	1(5.9%)
Fistula repair	-	1(4.5%)	-
Gastroenterostomy	-	1(4.5%)	-
Haemorrhoid operation	1(7.7%)	1(4.5%)	1(5.9%)
Hysterectomy	1(7.7%)	-	1(5.9%)
Ileocaecal resection	1(7.7%)	5(22.7%)	2(11.8%)
Ileostomy	-	-	1(5.9%)
Ovarian cystectomy	-	1(4.5%)	-
Parenteral nutrition	-	1(4.5%)	-
Sinus operation	-	-	1(5.9%)
Thyroidectomy	-	-	1(5.9%)
Toe amputation	-	-	1(5.9%)
Tonsillectomy	-	1(4.5%)	-
Tympanoplasty	-	1(4.5%)	-
Umbilical hernia repair	-	1(4.5%)	-
Vaginal fistula repair	1(7.7%)	-	-
Vascular disorders	3(23.1%)	5(22.7%)	3(17.6%)
Behcet's syndrome	-	1(4.5%)	-
Deep vein thrombosis	-	1(4.5%)	1(5.9%)
Hypertension	3(23.1%)	2(9.1%)	1(5.9%)
Hypotension	-	1(4.5%)	-
Thrombosis	-	-	1(5.9%)

Abbreviations: N, number of randomized subjects; n, number of subjects within each category; SOC, System Organ Class; PT, Preferred Term.

Program Name: MUCUS-Shell10a-V01
Output Location: .\Output\Shared\MUCUS-TFLS-Final-27MAR24-V01.pdf

Table 14.1.5: Summary of Prior Medications

	Naive (N=13)	Biologic (N=22)	Multiple Biologic (N=17)
Subjects with >= 1 Prior Medications	9 (69.2%)	22 (100.0%)	17 (100.0%)
Anesthetics	-	3 (13.6%)	3 (17.6%)
Propofol	-	3 (13.6%)	3 (17.6%)
Antianemic Preparations	-	2 (9.1%)	1 (5.9%)
Ferric Carboxymaltose	-	1 (4.5%)	-
Ferric Maltol	-	1 (4.5%)	1 (5.9%)
Antidiarrheals, Intestinal Antiinflammatory/Antiinfective Agents	8 (61.5%)	11 (50.0%)	13 (76.5%)
Bifidobacterium Longum;fibre, Dietary;lactobacillus Rhamnosus	-	1 (4.5%)	-
Budesonide	4 (30.8%)	6 (27.3%)	7 (41.2%)
Hydrocortisone Acetate	-	-	2 (11.8%)
Mesalazine	1 (7.7%)	4 (18.2%)	8 (47.1%)
Prednisolone	6 (46.2%)	8 (36.4%)	7 (41.2%)
Sulfasalazine	-	-	2 (11.8%)
Antineoplastic Agents	-	-	1 (5.9%)
Mercaptopurine	-	-	1 (5.9%)
Beta Blocking Agents	-	1 (4.5%)	-
Bisoprolol	-	1 (4.5%)	-
Bile And Liver Therapy	-	1 (4.5%)	-
Ursodeoxycholic Acid	-	1 (4.5%)	-
Corticosteroids For Systemic Use	-	1 (4.5%)	-
Steroids	-	1 (4.5%)	-
Drugs For Constipation	-	3 (13.6%)	-
Citric Acid;magnesium Oxide;sodium Picosulfate	-	2 (9.1%)	-
Macrogol 3350;potassium Chloride;sodium Bicarbonate;sodium Chloride;sodium Sulfate Anhydrous	-	1 (4.5%)	-
Drugs For Functional Gastrointestinal Disorders	-	1 (4.5%)	-
Mebeverine	-	1 (4.5%)	-
Immunosuppressants	4 (30.8%)	22 (100.0%)	17 (100.0%)
Adalimumab	-	11 (50.0%)	14 (82.4%)
Azathioprine	4 (30.8%)	10 (45.5%)	10 (58.8%)
Golimumab	-	-	1 (5.9%)

Abbreviations: N, number of randomized subjects; n, number of subjects within each category.

Program Name: MUCUS-Shell4-V01
Output Location: .\Output\Shared\MUCUS-TFLS-Final-27MAR24-V01.pdf

Table 14.1.5: Summary of Prior Medications

	Naive (N=13)	Biologic (N=22)	Multiple Biologic (N=17)
Infliximab	-	11 (50.0%)	14 (82.4%)
Methotrexate	-	1 (4.5%)	1 (5.9%)
Vedolizumab	-	1 (4.5%)	8 (47.1%)
Investigational Drug	-	-	1 (5.9%)
Mongersen	-	-	1 (5.9%)
Other Alimentary Tract And Metabolism Products	1 (7.7%)	-	-
Escherichia Coli	1 (7.7%)	-	-
Psycholeptics	-	2 (9.1%)	1 (5.9%)
Midazolam	-	2 (9.1%)	1 (5.9%)
Vasoprotectives	-	1 (4.5%)	1 (5.9%)
Betamethasone Sodium Phosphate	-	-	1 (5.9%)
Corticosteroids	-	1 (4.5%)	-
Vitamins	-	1 (4.5%)	-
Colecalciferol	-	1 (4.5%)	-

Abbreviations: N, number of randomized subjects; n, number of subjects within each category.

Program Name: MUCUS-Shell4-V01
Output Location: .\Output\Shared\MUCUS-TFLS-Final-27MAR24-V01.pdf

Table 14.1.6: Summary of Concomitant Medications

	Naive (N=13)	Biologic (N=22)	Multiple Biologic (N=17)
Subjects with >= 1 Concomitant Medications	12 (92.3%)	21 (95.5%)	17 (100.0%)
Agents Acting On The Renin-Angiotensin System	3 (23.1%)	2 (9.1%)	2 (11.8%)
Candesartan	2 (15.4%)	1 (4.5%)	-
Indapamide;perindopril Arginine	-	-	1 (5.9%)
Olmesartan	-	1 (4.5%)	-
Ramipril	1 (7.7%)	-	2 (11.8%)
Telmisartan	-	-	1 (5.9%)
All Other Therapeutic Products	-	-	2 (11.8%)
Ascorbic Acid	-	-	1 (5.9%)
Sodium Polystyrene Sulfonate	-	-	1 (5.9%)
Analgesics	7 (53.8%)	11 (50.0%)	5 (29.4%)
Caffeine;paracetamol;propyphenazone	1 (7.7%)	1 (4.5%)	3 (17.6%)
Dihydrocodeine	-	1 (4.5%)	1 (5.9%)
Metamizole	2 (15.4%)	2 (9.1%)	-
Metamizole Sodium	3 (23.1%)	6 (27.3%)	1 (5.9%)
Naloxone Hydrochloride;tilidine Hydrochloride	-	1 (4.5%)	-
Paracetamol	2 (15.4%)	1 (4.5%)	-
Pregabalin	-	1 (4.5%)	-
Sumatriptan	-	1 (4.5%)	-
Tramadol	-	1 (4.5%)	-
Tramadol Hydrochloride	-	1 (4.5%)	-
Anesthetics	1 (7.7%)	3 (13.6%)	2 (11.8%)
Propofol	1 (7.7%)	3 (13.6%)	2 (11.8%)
Anti-Parkinson Drugs	-	1 (4.5%)	-
Levodopa	-	1 (4.5%)	-
Antianemic Preparations	8 (61.5%)	5 (22.7%)	8 (47.1%)
Cyanocobalamin	3 (23.1%)	1 (4.5%)	3 (17.6%)
Ferric Carboxymaltose	5 (38.5%)	2 (9.1%)	3 (17.6%)
Ferric Maltol	1 (7.7%)	2 (9.1%)	1 (5.9%)
Ferrous Glycine Sulfate	1 (7.7%)	-	1 (5.9%)

Abbreviations: N, number of randomized subjects; n, number of subjects within each category.

Program Name: MUCUS-Shell4-V01
Output Location: .\Output\Shared\MUCUS-TFLS-Final-27MAR24-V01.pdf

Table 14.1.6: Summary of Concomitant Medications

	Naive (N=13)	Biologic (N=22)	Multiple Biologic (N=17)
Folic Acid	4 (30.8%)	3 (13.6%)	4 (23.5%)
Iron Isomaltoside 1000	-	-	1 (5.9%)
Saccharated Iron Oxide	1 (7.7%)	-	-
Vitamin B12 Nos	2 (15.4%)	1 (4.5%)	1 (5.9%)
Antibacterials For Systemic Use	1 (7.7%)	1 (4.5%)	4 (23.5%)
Antibiotics	1 (7.7%)	-	-
Cefuroxime	-	-	1 (5.9%)
Ciprofloxacin	-	1 (4.5%)	1 (5.9%)
Metronidazole	-	-	1 (5.9%)
Sulfamethoxazole;trimethoprim	-	-	1 (5.9%)
Antibiotics And Chemotherapeutics For Dermatological Use	1 (7.7%)	1 (4.5%)	-
Aciclovir	1 (7.7%)	1 (4.5%)	-
Antidiarrheals, Intestinal Antiinflammatory/Antiinfective Agents	10 (76.9%)	12 (54.5%)	11 (64.7%)
Betamethasone Valerate	-	1 (4.5%)	-
Bifidobacterium Animalis;enterococcus Faecium;lactobacillus Acidophilus ;lactobacillus Lactis;lactobacillus Salivarius;streptococcus Lactis	-	-	1 (5.9%)
Budesonide	4 (30.8%)	2 (9.1%)	3 (17.6%)
Colestyramine	1 (7.7%)	2 (9.1%)	1 (5.9%)
Loperamide	1 (7.7%)	-	-
Loperamide Hydrochloride	-	2 (9.1%)	1 (5.9%)
Malus Spp.	-	1 (4.5%)	-
Mesalazine	3 (23.1%)	1 (4.5%)	4 (23.5%)
Papaver Somniferum Tincture	-	1 (4.5%)	-
Prednisolone	4 (30.8%)	7 (31.8%)	6 (35.3%)
Vancomycin	-	-	1 (5.9%)
Antifungals For Dermatological Use	1 (7.7%)	-	-
Amorolfine	1 (7.7%)	-	-
Antihistamines For Systemic Use	1 (7.7%)	4 (18.2%)	-
Antihistamines	-	1 (4.5%)	-
Cetirizine Hydrochloride	-	2 (9.1%)	-
Desloratadine	1 (7.7%)	-	-

Abbreviations: N, number of randomized subjects; n, number of subjects within each category.

Program Name: MUCUS-Shell4-V01
Output Location: .\Output\Shared\MUCUS-TFLS-Final-27MAR24-V01.pdf

Table 14.1.6: Summary of Concomitant Medications

	Naive (N=13)	Biologic (N=22)	Multiple Biologic (N=17)
Dimetindene Maleate	-	1 (4.5%)	-
Ebastine	-	1 (4.5%)	-
Fexofenadine Hydrochloride	-	1 (4.5%)	-
Antiinflammatory And Antirheumatic Products	1 (7.7%)	-	1 (5.9%)
Celecoxib	1 (7.7%)	-	-
Curcuma Longa	-	-	1 (5.9%)
Ibuprofen	1 (7.7%)	-	-
Antimycobacterials	-	2 (9.1%)	-
Isoniazid	-	2 (9.1%)	-
Antithrombotic Agents	1 (7.7%)	3 (13.6%)	2 (11.8%)
Acetylsalicylic Acid	-	1 (4.5%)	1 (5.9%)
Apixaban	-	1 (4.5%)	-
Certoparin Sodium	-	1 (4.5%)	-
Dalteparin Sodium	1 (7.7%)	-	-
Rivaroxaban	-	1 (4.5%)	1 (5.9%)
Ticagrelor	-	-	1 (5.9%)
Tinzaparin Sodium	-	1 (4.5%)	-
Beta Blocking Agents	1 (7.7%)	4 (18.2%)	1 (5.9%)
Bisoprolol	-	3 (13.6%)	-
Bisoprolol Fumarate	1 (7.7%)	-	-
Metoprolol	-	-	1 (5.9%)
Metoprolol Succinate	-	1 (4.5%)	-
Bile And Liver Therapy	-	1 (4.5%)	-
Ursodeoxycholic Acid	-	1 (4.5%)	-
Blood Substitutes And Perfusion Solutions	-	1 (4.5%)	1 (5.9%)
Alanine;arginine;aspartic Acid;calcium Chloride;glucose Monohydrate;glutamic Acid;glycine;glycine Max Oil;histidine Hydrochloride;isoleucine;leucine;lysine Hydrochloride;magnesium Acetate Tetrahydrate;medium-Chain Triglycerides;methionine;phenylalanine;potassium Acetate;proline;serine;sodium Acetate;sodium Chloride;sodium Hydroxide;sodium Phosphate Monobasic (Dihydrate);threonine;tryptophan, L-;valine;zinc Acetate Dihydrate	-	1 (4.5%)	-
Calcium Chloride Dihydrate;potassium Chloride;sodium Lactate	-	-	1 (5.9%)
Cardiac Therapy	-	1 (4.5%)	-
Glyceryl Trinitrate	-	1 (4.5%)	-
Corticosteroids For Systemic Use	1 (7.7%)	-	1 (5.9%)

Abbreviations: N, number of randomized subjects; n, number of subjects within each category.

Program Name: MUCUS-Shell4-V01
Output Location: .\Output\Shared\MUCUS-TFLS-Final-27MAR24-V01.pdf

Table 14.1.6: Summary of Concomitant Medications

	Naive (N=13)	Biologic (N=22)	Multiple Biologic (N=17)
Methylprednisolone	-	-	1 (5.9%)
Prednisone	1 (7.7%)	-	-
Corticosteroids, Dermatological Preparations	-	1 (4.5%)	-
Mometasone Furoate	-	1 (4.5%)	-
Diuretics	1 (7.7%)	-	2 (11.8%)
Hydrochlorothiazide	1 (7.7%)	-	-
Torasemide	-	-	2 (11.8%)
Drugs For Acid Related Disorders	6 (46.2%)	6 (27.3%)	7 (41.2%)
Calcium Carbonate	1 (7.7%)	-	-
Esomeprazole	1 (7.7%)	-	1 (5.9%)
Pantoprazole	5 (38.5%)	5 (22.7%)	5 (29.4%)
Pantoprazole Sodium Sesquihydrate	-	1 (4.5%)	1 (5.9%)
Drugs For Constipation	2 (15.4%)	3 (13.6%)	1 (5.9%)
Citric Acid;magnesium Oxide;sodium Picosulfate	-	1 (4.5%)	-
Macrogol	-	1 (4.5%)	-
Macrogol 3350;potassium Chloride;sodium Bicarbonate;sodium Chloride;sodium Sulfate Anhydrous	1 (7.7%)	-	1 (5.9%)
Plantago Ovata	1 (7.7%)	-	-
Plantago Ovata Seed Husk	-	1 (4.5%)	-
Drugs For Functional Gastrointestinal Disorders	2 (15.4%)	2 (9.1%)	1 (5.9%)
Hyoscine Butylbromide	-	2 (9.1%)	-
Hyoscine Butylbromide;paracetamol	-	-	1 (5.9%)
Mebeverine Embonate	1 (7.7%)	-	-
Simeticone	1 (7.7%)	-	-
Drugs For Obstructive Airway Diseases	-	1 (4.5%)	2 (11.8%)
Budesonide;formoterol Fumarate	-	-	1 (5.9%)
Fluticasone Furoate;umeclidinium Bromide;vilanterol Trifenatate	-	-	1 (5.9%)
Fluticasone Furoate;vilanterol Trifenatate	-	-	1 (5.9%)
Salbutamol	-	-	1 (5.9%)
Tiotropium Bromide Monohydrate	-	1 (4.5%)	-
Drugs Used In Diabetes	-	-	1 (5.9%)

Abbreviations: N, number of randomized subjects; n, number of subjects within each category.

Program Name: MUCUS-Shell4-V01
Output Location: .\Output\Shared\MUCUS-TFLS-Final-27MAR24-V01.pdf

Table 14.1.6: Summary of Concomitant Medications

	Naive (N=13)	Biologic (N=22)	Multiple Biologic (N=17)
Insulin	-	-	1 (5.9%)
General Nutrients	1 (7.7%)	1 (4.5%)	1 (5.9%)
Carbohydrates Nos;fats Nos;minerals Nos;proteins Nos;vitamins Nos	-	1 (4.5%)	-
Carbohydrates Nos;fatty Acids Nos;minerals Nos;proteins Nos;vitamins No s	-	-	1 (5.9%)
Nutrients Nos	1 (7.7%)	-	-
Immunosuppressants	1 (7.7%)	5 (22.7%)	2 (11.8%)
Adalimumab	1 (7.7%)	1 (4.5%)	-
Azathioprine	-	2 (9.1%)	1 (5.9%)
Infliximab	-	1 (4.5%)	-
Methotrexate	-	-	1 (5.9%)
Ustekinumab	-	2 (9.1%)	-
Lipid Modifying Agents	2 (15.4%)	3 (13.6%)	3 (17.6%)
Atorvastatin	-	-	1 (5.9%)
Colesevelam Hydrochloride	-	1 (4.5%)	1 (5.9%)
Colestyramine	1 (7.7%)	1 (4.5%)	-
Ezetimibe	-	-	2 (11.8%)
Rosuvastatin	-	1 (4.5%)	1 (5.9%)
Simvastatin	1 (7.7%)	-	-
Mineral Supplements	3 (23.1%)	3 (13.6%)	2 (11.8%)
Calcium Carbonate;colecalciferol	1 (7.7%)	1 (4.5%)	-
Calcium Carbonate;colecalciferol;menaquinone	-	-	1 (5.9%)
Magnesium	-	1 (4.5%)	1 (5.9%)
Potassium Chloride	-	1 (4.5%)	-
Zinc Orotate	2 (15.4%)	-	-
Zinc Oxide	-	1 (4.5%)	-
Nasal Preparations	-	-	1 (5.9%)
Mometasone Furoate	-	-	1 (5.9%)
Other Alimentary Tract And Metabolism Products	1 (7.7%)	-	-
Zinc	1 (7.7%)	-	-
Otologicals	1 (7.7%)	-	-

Abbreviations: N, number of randomized subjects; n, number of subjects within each category.

Program Name: MUCUS-Shell4-V01
Output Location: .\Output\Shared\MUCUS-TFLS-Final-27MAR24-V01.pdf

Table 14.1.6: Summary of Concomitant Medications

	Naive (N=13)	Biologic (N=22)	Multiple Biologic (N=17)
Ofloxacin	1 (7.7%)	-	-
Psychoanaleptics	2 (15.4%)	-	-
Escitalopram	1 (7.7%)	-	-
Sertraline	1 (7.7%)	-	-
Trimipramine	1 (7.7%)	-	-
Psycholeptics	1 (7.7%)	1 (4.5%)	1 (5.9%)
Midazolam	-	1 (4.5%)	1 (5.9%)
Pipamperone Hydrochloride	1 (7.7%)	-	-
Temazepam	1 (7.7%)	-	-
Sex Hormones And Modulators Of The Genital System	1 (7.7%)	2 (9.1%)	-
Chlormadinone Acetate;ethinylestradiol	-	1 (4.5%)	-
Ethinylestradiol;levonorgestrel	1 (7.7%)	1 (4.5%)	-
Stomatological Preparations	-	-	2 (11.8%)
Hydrocortisone	-	-	1 (5.9%)
Zinc	-	-	1 (5.9%)
Thyroid Therapy	3 (23.1%)	1 (4.5%)	1 (5.9%)
Levothyroxine	3 (23.1%)	1 (4.5%)	-
Levothyroxine Sodium	-	-	1 (5.9%)
Vaccines	2 (15.4%)	3 (13.6%)	1 (5.9%)
Covid-19 Vaccine Nrvv Ad (Chadox1 Ncov-19)	1 (7.7%)	-	-
Elasomeran	1 (7.7%)	-	-
Influenza Vaccine Inact Sag 4v	1 (7.7%)	1 (4.5%)	-
Tozinameran	1 (7.7%)	2 (9.1%)	1 (5.9%)
Vasoprotectives	-	1 (4.5%)	-
Lidocaine	-	1 (4.5%)	-
Lidocaine Hydrochloride	-	1 (4.5%)	-
Vitamins	7 (53.8%)	5 (22.7%)	8 (47.1%)
Colecalciferol	6 (46.2%)	5 (22.7%)	8 (47.1%)
Nicotinamide;pyridoxine Hydrochloride;riboflavin;thiamine Hydrochloride	-	-	1 (5.9%)
Pyridoxine Hydrochloride	-	1 (4.5%)	-

Abbreviations: N, number of randomized subjects; n, number of subjects within each category.

Program Name: MUCUS-Shell4-V01
Output Location: .\Output\Shared\MUCUS-TFLS-Final-27MAR24-V01.pdf

Table 14.1.6: Summary of Concomitant Medications

	Naive (N=13)	Biologic (N=22)	Multiple Biologic (N=17)
Vitamin D Nos	1 (7.7%)	-	-

Abbreviations: N, number of randomized subjects; n, number of subjects within each category.

Program Name: MUCUS-Shell4-V01
Output Location: .\Output\Shared\MUCUS-TFLS-Final-27MAR24-V01.pdf

Table 14.1.7: Summary of Protocol Deviations

	Naive (N=13)	Biologic (N=22)	Multiple Biologic (N=17)
Major: n(%)	5 (38.5%)	16 (72.7%)	9 (52.9%)
Inclusion/Exclusion Criteria - Subject	4 (30.8%)	3 (13.6%)	7 (41.2%)
Informed Consent - Subject	-	3 (13.6%)	-
Investigational Product - Subject	1 (7.7%)	2 (9.1%)	-
Laboratory - Subject	-	3 (13.6%)	-
Procedures/Tests/Assessments - Site	-	1 (4.5%)	-
Procedures/Tests/Assessments - Subject	-	6 (27.3%)	3 (17.6%)
Randomization - Site	-	1 (4.5%)	-
Visit Schedule - Subject	-	3 (13.6%)	-
Minor: n(%)	10 (76.9%)	20 (90.9%)	12 (70.6%)
Inclusion/Exclusion Criteria - Subject	2 (15.4%)	1 (4.5%)	1 (5.9%)
Informed Consent - Subject	1 (7.7%)	-	2 (11.8%)
Investigational Product - Site	-	2 (9.1%)	-
Laboratory - Subject	-	1 (4.5%)	-
Procedures/Tests/Assessments - Site	1 (7.7%)	5 (22.7%)	1 (5.9%)
Procedures/Tests/Assessments - Subject	5 (38.5%)	13 (59.1%)	6 (35.3%)
Visit Schedule - Subject	7 (53.8%)	13 (59.1%)	7 (41.2%)

Abbreviations: N, number of randomized subjects; n, number of subjects within each category.

Program Name: MUCUS-Shell5-V01
Output Location: .\Output\Shared\MUCUS-TFLS-Final-27MAR24-V01.pdf

Table 14.2.1.1: Summary of Clinical Response and Endoscopic Response at Week 52: ITT Population

	Naive (N=13)	Biologic (N=22)	Multiple Biologic (N=17)
Clinical Response + Endoscopic Response			
Week 52			
N	13	22	17
[Responder] n(%)	3 (23.1%)	5 (22.7%)	5 (29.4%)
[Non-Responder] n(%)	10 (76.9%)	17 (77.3%)	12 (70.6%)
[Responder] Odds Ratio Strata 1 vs Strata 2 or 3		1.020	0.720
Odds Ratio 95% CI		(0.200, 5.209)	(0.137, 3.784)
Odds Ratio P-value		0.981	0.698
[Responder] Odds Ratio Strata 2 vs Strata 3			0.706
Odds Ratio 95% CI			(0.167, 2.989)
Odds Ratio P-value			0.636

Abbreviations: ITT, Intent-to-Treat Analysis set; N, number of randomized subjects; n, number of subjects within each category; Outcome definition, Reduction from baseline in SES-CD score >= 50% and Harvey Bradshaw Index decrease >= 3 points; Proc Logistic used to generate Odds Ratios and P-values.

Program Name: MUCUS-Shell6-V01-NC
Output Location: .\Output\Shared\MUCUS-TFLS-Final-27MAR24-V01.pdf

Table 14.2.1.2: Summary of Clinical Response and Endoscopic Response at Week 52: PP Population

	Naive (N=6)	Biologic (N=11)	Multiple Biologic (N=8)
Clinical Response + Endoscopic Response			
Week 52			
N	6	11	8
[Responder] n(%)	3 (50.0%)	5 (45.5%)	5 (62.5%)
[Non-Responder] n(%)	3 (50.0%)	6 (54.5%)	3 (37.5%)
[Responder] Odds Ratio Strata 1 vs Strata 2 or 3		1.200	0.600
Odds Ratio 95% CI		(0.164, 8.799)	(0.070, 5.136)
Odds Ratio P-value		0.858	0.641
[Responder] Odds Ratio Strata 2 vs Strata 3			0.500
Odds Ratio 95% CI			(0.078, 3.210)
Odds Ratio P-value			0.465

Abbreviations: N, number of randomized subjects; n, number of subjects within each category; Outcome definition, Reduction from baseline in SES-CD score >= 50% and Harvey Bradshaw Index decrease >= 3 points; Proc Logistic used to generate Odds Ratios and P-values.

Program Name: MUCUS-Shell6-V01-NC
Output Location: .\Output\Shared\MUCUS-TFLS-Final-27MAR24-V01.pdf

Table 14.2.1.3: Summary of Clinical Response and Endoscopic Response at Week 52 Gender Subgroup Analysis: ITT Population

	Naive (N=13)	Biologic (N=22)	Multiple Biologic (N=17)
Clinical Response + Endoscopic Response			
Week 52			
Women			
N	8	14	6
[Responder] n(%)	2 (25.0%)	4 (28.6%)	1 (16.7%)
[Non-Responder] n(%)	6 (75.0%)	10 (71.4%)	5 (83.3%)
[Responder] Odds Ratio Strata 1 vs Strata 2 or 3		0.833	1.667
Odds Ratio 95% CI		(0.115, 6.013)	(0.115, 24.256)
Odds Ratio P-value		0.857	0.708
[Responder] Odds Ratio Strata 2 vs Strata 3			2.000
Odds Ratio 95% CI			(0.174, 22.949)
Odds Ratio P-value			0.578
Men			
N	5	8	11
[Responder] n(%)	1 (20.0%)	1 (12.5%)	4 (36.4%)
[Non-Responder] n(%)	4 (80.0%)	7 (87.5%)	7 (63.6%)
[Responder] Odds Ratio Strata 1 vs Strata 2 or 3		1.750	0.437
Odds Ratio 95% CI		(0.084, 36.284)	(0.035, 5.395)
Odds Ratio P-value		0.718	0.519
[Responder] Odds Ratio Strata 2 vs Strata 3			0.250
Odds Ratio 95% CI			(0.022, 2.837)
Odds Ratio P-value			0.263
Interaction P-value strata 1 vs Strata 3			0.519
Interaction P-value strata 2 vs Strata 3			0.263

Abbreviations: ITT, Intent-to-Treat Analysis set; N, number of randomized subjects; n, number of subjects within each category; Outcome definition, Reduction from baseline in SES-CD score >= 50% and Harvey Bradshaw Index decrease >= 3 points; Proc Logistic used to generate Odds Ratios and P-values.

Program Name: MUCUS-Shell6-V01-NC
Output Location: .\Output\Shared\MUCUS-TFLS-Final-27MAR24-V01.pdf

Table 14.2.2: Summary of Clinical Response at Week 52: ITT Population

	Naive (N=13)	Biologic (N=22)	Multiple Biologic (N=17)
Clinical Response			
Week 52			
N	13	22	17
[Responder] n(%)	8 (61.5%)	9 (40.9%)	8 (47.1%)
[Non-Responder] n(%)	5 (38.5%)	13 (59.1%)	9 (52.9%)
[Responder] Odds Ratio Strata 1 vs Strata 2 or 3		2.311	1.800
Odds Ratio 95% CI		(0.568, 9.408)	(0.415, 7.814)
Odds Ratio P-value		0.242	0.433
[Responder] Odds Ratio Strata 2 vs Strata 3			0.779
Odds Ratio 95% CI			(0.217, 2.791)
Odds Ratio P-value			0.701

Abbreviations: ITT, Intent-to-Treat Analysis set; N, number of randomized subjects; n, number of subjects within each category; Outcome definition, Reduction from baseline in Harvey Bradshaw Index >= 3 points; Proc Logistic used to generate Odds Ratios and P-values.

Program Name: MUCUS-Shell6-V01-NC
Output Location: .\Output\Shared\MUCUS-TFLS-Final-27MAR24-V01.pdf

Table 14.2.3.1: Summary of Endoscopic Response at Week 52: ITT Population

	Naive (N=13)	Biologic (N=22)	Multiple Biologic (N=17)
Endoscopic Response			
Week 52			
N	13	22	17
[Responder] n(%)	3 (23.1%)	6 (27.3%)	6 (35.3%)
[Non-Responder] n(%)	10 (76.9%)	16 (72.7%)	11 (64.7%)
[Responder] Odds Ratio Strata 1 vs Strata 2 or 3		0.800	0.550
Odds Ratio 95% CI		(0.162, 3.944)	(0.108, 2.805)
Odds Ratio P-value		0.784	0.472
[Responder] Odds Ratio Strata 2 vs Strata 3			0.687
Odds Ratio 95% CI			(0.175, 2.698)
Odds Ratio P-value			0.591

Abbreviations: ITT, Intent-to-Treat Analysis set; N, number of randomized subjects; n, number of subjects within each category; Outcome definition, Reduction from baseline in SES-CD score >= 50%; Proc Logistic used to generate Odds Ratios and P-values.

Program Name: MUCUS-Shell6-V01-NC
Output Location: .\Output\Shared\MUCUS-TFLS-Final-27MAR24-V01.pdf

Table 14.2.3.2: Summary of Endoscopic Response at Week 52: Per-Protocol Analysis Set

	Naive (N=6)	Biologic (N=11)	Multiple Biologic (N=8)
Endoscopic Response			
Week 52			
N	6	11	8
[Responder] n(%)	3 (50.0%)	6 (54.5%)	6 (75.0%)
[Non-Responder] n(%)	3 (50.0%)	5 (45.5%)	2 (25.0%)
[Responder] Odds Ratio Strata 1 vs Strata 2 or 3		0.833	0.333
Odds Ratio 95% CI		(0.114, 6.111)	(0.035, 3.205)
Odds Ratio P-value		0.858	0.341
[Responder] Odds Ratio Strata 2 vs Strata 3			0.400
Odds Ratio 95% CI			(0.055, 2.933)
Odds Ratio P-value			0.367

Abbreviations: N, number of randomized subjects; n, number of subjects within each category; Outcome definition, Reduction from baseline in SES-CD score >= 50%; Proc Logistic used to generate Odds Ratios and P-values.

Program Name: MUCUS-Shell6-V01-NC
Output Location: .\Output\Shared\MUCUS-TFLS-Final-27MAR24-V01.pdf

Table 14.2.4.1: Summary of Endoscopic Remission at Week 26 and 52: ITT Population

	Naive (N=14)	Biologic (N=22)	Multiple Biologic (N=17)
Endoscopic Remission			
Week 26			
N	1	-	-
[Remitter] n(%)	-	-	-
[Non-Remitter] n(%)	1 (100.0%)	-	-
Week 52			
N	13	22	17
[Remitter] n(%)	3 (23.1%)	3 (13.6%)	3 (17.6%)
[Non-Remitter] n(%)	10 (76.9%)	19 (86.4%)	14 (82.4%)
[Remitter] Odds Ratio Strata 1 vs Strata 2 or 3		1.900	1.400
Odds Ratio 95% CI		(0.322, 11.200)	(0.233, 8.421)
Odds Ratio P-value		0.478	0.713
[Remitter] Odds Ratio Strata 2 vs Strata 3			0.737
Odds Ratio 95% CI			(0.129, 4.210)
Odds Ratio P-value			0.731

Abbreviations: ITT, Intent-to-Treat Analysis set; N, number of randomized subjects; n, number of subjects within each category; Outcome definition, SES-CD score <=2; Proc Logistic used to generate Odds Ratios and P-values.

Program Name: MUCUS-Shell6-V01-NC
Output Location: .\Output\Shared\MUCUS-TFLS-Final-27MAR24-V01.pdf

Table 14.2.4.2: Summary of Endoscopic Remission at Week 26 and 52: Per-Protocol Analysis Set

	Naive (N=7)	Biologic (N=11)	Multiple Biologic (N=8)
Endoscopic Remission			
Week 26			
N	1	-	-
[Remitter] n(%)	-	-	-
[Non-Remitter] n(%)	1 (100.0%)	-	-
Week 52			
N	6	11	8
[Remitter] n(%)	3 (50.0%)	3 (27.3%)	3 (37.5%)
[Non-Remitter] n(%)	3 (50.0%)	8 (72.7%)	5 (62.5%)
[Remitter] Odds Ratio Strata 1 vs Strata 2 or 3		2.666	1.667
Odds Ratio 95% CI		(0.333, 21.317)	(0.195, 14.266)
Odds Ratio P-value		0.355	0.641
[Remitter] Odds Ratio Strata 2 vs Strata 3			0.625
Odds Ratio 95% CI			(0.089, 4.401)
Odds Ratio P-value			0.637

Abbreviations: N, number of randomized subjects; n, number of subjects within each category; Outcome definition, SES-CD score <=2; Proc Logistic used to generate Odds Ratios and P-values.

Program Name: MUCUS-Shell6-V01-NC
Output Location: .\Output\Shared\MUCUS-TFLS-Final-27MAR24-V01.pdf

Table 14.2.5.1: Summary of Mucosal Healing Rate at Week 26 and 52: ITT Population

	Naive (N=14)	Biologic (N=22)	Multiple Biologic (N=17)
Mucosal Healing			
Week 26			
N	1	-	-
[Responder] n(%)	1 (100.0%)	-	-
[Non-Responder] n(%)	-	-	-
Week 52			
N	13	22	17
[Responder] n(%)	3 (23.1%)	4 (18.2%)	6 (35.3%)
[Non-Responder] n(%)	10 (76.9%)	18 (81.8%)	11 (64.7%)
[Responder] Odds Ratio Strata 1 vs Strata 2 or 3		1.350	0.550
Odds Ratio 95% CI		(0.250, 7.278)	(0.108, 2.805)
Odds Ratio P-value		0.727	0.472
[Responder] Odds Ratio Strata 2 vs Strata 3			0.407
Odds Ratio 95% CI			(0.094, 1.773)
Odds Ratio P-value			0.231

Abbreviations: ITT, Intent-to-Treat Analysis set; N, number of randomized subjects; n, number of subjects within each category; Outcome definition, Complete abscense of mucosal ulcerations; Proc Logistic used to generate Odds Ratios and P-values;

Program Name: MUCUS-Shell6-V01-NC
Output Location: .\Output\Shared\MUCUS-TFLS-Final-27MAR24-V01.pdf

Table 14.2.5.2: Summary of Mucosal Healing Rate at Week 26 and 52: Per-Protocol Analysis Set

	Naive (N=7)	Biologic (N=11)	Multiple Biologic (N=8)
Mucosal Healing			
Week 26			
N	1	-	-
[Responder] n(%)	1 (100.0%)	-	-
[Non-Responder] n(%)	-	-	-
Week 52			
N	6	11	8
[Responder] n(%)	3 (50.0%)	4 (36.4%)	5 (62.5%)
[Non-Responder] n(%)	3 (50.0%)	7 (63.6%)	3 (37.5%)
[Responder] Odds Ratio Strata 1 vs Strata 2 or 3		1.750	0.600
Odds Ratio 95% CI		(0.233, 13.158)	(0.070, 5.136)
Odds Ratio P-value		0.587	0.641
[Responder] Odds Ratio Strata 2 vs Strata 3			0.343
Odds Ratio 95% CI			(0.052, 2.261)
Odds Ratio P-value			0.266

Abbreviations: N, number of randomized subjects; n, number of subjects within each category; Outcome definition, Complete abscense of mucosal ulcerations; Proc Logistic used to generate Odds Ratios and P-values.

Program Name: MUCUS-Shell6-V01-NC
Output Location: .\Output\Shared\MUCUS-TFLS-Final-27MAR24-V01.pdf

Table 14.2.6: Summary of Clinical Remission at Week 8, 16, 26, 36, 52: ITT Population

	Naive (N=13)	Biologic (N=22)	Multiple Biologic (N=17)
Clinical Remission			
Week 8			
N	12	21	14
[Remitter] n(%)	7 (58.3%)	11 (52.4%)	11 (78.6%)
[Non-Remitter] n(%)	5 (41.7%)	10 (47.6%)	3 (21.4%)
[Remitter] Odds Ratio Strata 1 vs Strata 2 or 3		1.280	0.227
Odds Ratio 95% CI		(0.116, 14.164)	(0.014, 3.566)
Odds Ratio P-value		0.839	0.289
[Remitter] Odds Ratio Strata 2 vs Strata 3			0.177
Odds Ratio 95% CI			(0.015, 2.061)
Odds Ratio P-value			0.165
Week 16			
N	9	19	13
[Remitter] n(%)	8 (88.9%)	8 (42.1%)	9 (69.2%)
[Non-Remitter] n(%)	1 (11.1%)	11 (57.9%)	4 (30.8%)
[Remitter] Odds Ratio Strata 1 vs Strata 2 or 3		40.237	7.517
Odds Ratio 95% CI		(1.219, 1328.3)	(0.204, 276.47)
Odds Ratio P-value		0.039	0.270
[Remitter] Odds Ratio Strata 2 vs Strata 3			0.187
Odds Ratio 95% CI			(0.017, 2.047)
Odds Ratio P-value			0.168
Week 26			
N	7	13	7
[Remitter] n(%)	5 (71.4%)	6 (46.2%)	7 (100.0%)
[Non-Remitter] n(%)	2 (28.6%)	7 (53.8%)	-
[Remitter] Odds Ratio Strata 1 vs Strata 2 or 3		7.360	<0.001

Abbreviations: ITT, Intent-to-Treat Analysis set; N, number of randomized subjects; n, number of subjects within each category; Outcome definition, Harvey Bradshaw Index <=4; Proc GLIMMIX used to generate Odds Ratios and P-values.

Program Name: MUCUS-Shell6-V01-NC
Output Location: .\Output\Shared\MUCUS-TFLS-Final-27MAR24-V01.pdf

Table 14.2.6: Summary of Clinical Remission at Week 8, 16, 26, 36, 52: ITT Population

	Naive (N=13)	Biologic (N=22)	Multiple Biologic (N=17)
Odds Ratio 95% CI		(0.320, 169.29)	(<0.001, NE)
Odds Ratio P-value		0.210	0.992
[Remitter] Odds Ratio Strata 2 vs Strata 3			<0.001
Odds Ratio 95% CI			(<0.001, NE)
Odds Ratio P-value			0.991
Week 36			
N	8	10	9
[Remitter] n(%)	6 (75.0%)	5 (50.0%)	6 (66.7%)
[Non-Remitter] n(%)	2 (25.0%)	5 (50.0%)	3 (33.3%)
[Remitter] Odds Ratio Strata 1 vs Strata 2 or 3		11.165	2.733
Odds Ratio 95% CI		(0.393, 317.28)	(0.096, 77.528)
Odds Ratio P-value		0.156	0.553
[Remitter] Odds Ratio Strata 2 vs Strata 3			0.245
Odds Ratio 95% CI			(0.014, 4.233)
Odds Ratio P-value			0.330
Week 52			
N	13	22	17
[Remitter] n(%)	7 (53.8%)	10 (45.5%)	7 (41.2%)
[Non-Remitter] n(%)	6 (46.2%)	12 (54.5%)	10 (58.8%)
[Remitter] Odds Ratio Strata 1 vs Strata 2 or 3		1.620	2.210
Odds Ratio 95% CI		(0.157, 16.670)	(0.190, 25.711)
Odds Ratio P-value		0.683	0.524
[Remitter] Odds Ratio Strata 2 vs Strata 3			1.364
Odds Ratio 95% CI			(0.159, 11.716)
Odds Ratio P-value			0.776

Abbreviations: ITT, Intent-to-Treat Analysis set; N, number of randomized subjects; n, number of subjects within each category; Outcome definition, Harvey Bradshaw Index <=4; Proc GLIMMIX used to generate Odds Ratios and P-values.

Program Name: MUCUS-Shell6-V01-NC
Output Location: .\Output\Shared\MUCUS-TFLS-Final-27MAR24-V01.pdf

Table 14.2.7: Summary of Steroid Free Clinical Remission at Week 8, 16, 26, 36, 52

	Naive (N=13)	Biologic (N=22)	Multiple Biologic (N=17)
Steroid Free Clinical Remission			
Week 8			
N	6	4	9
[Remitter] n(%)	-	-	-
[Non-Remitter] n(%)	6 (100.0%)	4 (100.0%)	9 (100.0%)
[Remitter] Odds Ratio Strata 1 vs Strata 2 or 3		1722.2	<0.001
Odds Ratio 95% CI		(0.018, 1.65E8)	(<0.001, 3.542)
Odds Ratio P-value		0.197	0.078
[Remitter] Odds Ratio Strata 2 vs Strata 3			<0.001
Odds Ratio 95% CI			(<0.001, <0.001)
Odds Ratio P-value			<.001
Week 16			
N	4	4	8
[Remitter] n(%)	2 (50.0%)	1 (25.0%)	2 (25.0%)
[Non-Remitter] n(%)	2 (50.0%)	3 (75.0%)	6 (75.0%)
[Remitter] Odds Ratio Strata 1 vs Strata 2 or 3		3137.3	7.325
Odds Ratio 95% CI		(0.009, 1.07E9)	(<0.001, 832000)
Odds Ratio P-value		0.209	0.732
[Remitter] Odds Ratio Strata 2 vs Strata 3			0.002
Odds Ratio 95% CI			(<0.001, 1490.5)
Odds Ratio P-value			0.365
Week 26			
N	3	2	4
[Remitter] n(%)	-	1 (50.0%)	1 (25.0%)
[Non-Remitter] n(%)	3 (100.0%)	1 (50.0%)	3 (75.0%)
[Remitter] Odds Ratio Strata 1 vs Strata 2 or 3		<0.001	<0.001

Abbreviations: ITT, Intent-to-Treat Analysis set; N, number of randomized subjects; n, number of subjects within each category; Outcome definition,Harvey Bradshaw index score of <= 4 and the absence of concomitant steroid therapy in subjects who were on steroids at baseline; Proc GLIMMIX used to generate Odds Ratios and P-values.

Program Name: MUCUS-Shell6-V01-NC
Output Location: .\Output\Shared\MUCUS-TFLS-Final-27MAR24-V01.pdf

Table 14.2.7: Summary of Steroid Free Clinical Remission at Week 8, 16, 26, 36, 52

	Naive (N=13)	Biologic (N=22)	Multiple Biologic (N=17)
Odds Ratio 95% CI		(<0.001, 16937)	(<0.001, <0.001)
Odds Ratio P-value		0.145	<.001
[Remitter] Odds Ratio Strata 2 vs Strata 3			0.302
Odds Ratio 95% CI			(<0.001, 784E12)
Odds Ratio P-value			0.946
Week 36			
N	3	2	5
[Remitter] n(%)	1 (33.3%)	1 (50.0%)	1 (20.0%)
[Non-Remitter] n(%)	2 (66.7%)	1 (50.0%)	4 (80.0%)
[Remitter] Odds Ratio Strata 1 vs Strata 2 or 3		72.704	25.196
Odds Ratio 95% CI		(<0.001, 657E14)	(<0.001, 8.51E6)
Odds Ratio P-value		0.803	0.612
[Remitter] Odds Ratio Strata 2 vs Strata 3			0.347
Odds Ratio 95% CI			(<0.001, 784E12)
Odds Ratio P-value			0.952
Week 52			
N	6	5	9
[Remitter] n(%)	2 (33.3%)	2 (40.0%)	2 (22.2%)
[Non-Remitter] n(%)	4 (66.7%)	3 (60.0%)	7 (77.8%)
[Remitter] Odds Ratio Strata 1 vs Strata 2 or 3		0.091	0.671
Odds Ratio 95% CI		(<0.001, 8755.5)	(<0.001, 122002)
Odds Ratio P-value		0.676	0.947
[Remitter] Odds Ratio Strata 2 vs Strata 3			7.342
Odds Ratio 95% CI			(<0.001, 77994)
Odds Ratio P-value			0.667

Abbreviations: ITT, Intent-to-Treat Analysis set; N, number of randomized subjects; n, number of subjects within each category; Outcome definition,Harvey Bradshaw index score of <= 4 and the absence of concomitant steroid therapy in subjects who were on steroids at baseline; Proc GLIMMIX used to generate Odds Ratios and P-values.

Program Name: MUCUS-Shell6-V01-NC
Output Location: .\Output\Shared\MUCUS-TFLS-Final-27MAR24-V01.pdf

Table 14.2.8.1: Summary of Fistula Response at Week 8, 16, 26, 36, 52

	Naive (N=13)	Biologic (N=22)	Multiple Biologic (N=17)
Fistulizing Crohns Response			
Week 8			
N	-	2	3
[Responder] n(%)	-	1 (50.0%)	2 (66.7%)
[Non-Responder] n(%)	-	1 (50.0%)	1 (33.3%)
[Responder] Odds Ratio Strata 1 vs Strata 2 or 3		-	-
Odds Ratio 95% CI		-	-
Odds Ratio P-value		-	-
[Responder] Odds Ratio Strata 2 vs Strata 3			0.431
Odds Ratio 95% CI			(<0.001, 118E9)
Odds Ratio P-value			0.938
Week 16			
N	-	2	3
[Responder] n(%)	-	1 (50.0%)	2 (66.7%)
[Non-Responder] n(%)	-	1 (50.0%)	1 (33.3%)
[Responder] Odds Ratio Strata 1 vs Strata 2 or 3		-	-
Odds Ratio 95% CI		-	-
Odds Ratio P-value		-	-
[Responder] Odds Ratio Strata 2 vs Strata 3			0.432
Odds Ratio 95% CI			(<0.001, 118E9)
Odds Ratio P-value			0.938
Week 26			
N	-	-	-
[Responder] n(%)	-	-	-
[Non-Responder] n(%)	-	-	-
[Responder] Odds Ratio Strata 1 vs Strata 2 or 3		-	-

Abbreviations: ITT, Intent-to-Treat Analysis set; N, number of randomized subjects; n, number of subjects within each category; Outcome definition,For patients with baseline Perianal Disease Activity Index (PDAI) score >=4 follow up PDAI=0 indicates remmission; Proc Glimmix used to generate Odds Ratios and P-values.

Program Name: MUCUS-Shell6-V01-NC
Output Location: .\Output\Shared\MUCUS-TFLS-Final-27MAR24-V01.pdf

Table 14.2.8.1: Summary of Fistula Response at Week 8, 16, 26, 36, 52

	Naive (N=13)	Biologic (N=22)	Multiple Biologic (N=17)
Odds Ratio 95% CI		-	-
Odds Ratio P-value		-	-
[Responder] Odds Ratio Strata 2 vs Strata 3			-
Odds Ratio 95% CI			-
Odds Ratio P-value			-
Week 36			
N	-	-	-
[Responder] n(%)	-	-	-
[Non-Responder] n(%)	-	-	-
[Responder] Odds Ratio Strata 1 vs Strata 2 or 3		-	-
Odds Ratio 95% CI		-	-
Odds Ratio P-value		-	-
[Responder] Odds Ratio Strata 2 vs Strata 3			-
Odds Ratio 95% CI			-
Odds Ratio P-value			-
Week 52			
N	-	1	3
[Responder] n(%)	-	1 (100.0%)	3 (100.0%)
[Non-Responder] n(%)	-	-	-
[Responder] Odds Ratio Strata 1 vs Strata 2 or 3		-	-
Odds Ratio 95% CI		-	-
Odds Ratio P-value		-	-
[Responder] Odds Ratio Strata 2 vs Strata 3			19.042
Odds Ratio 95% CI			(<0.001, 5189E9)
Odds Ratio P-value			0.785

Abbreviations: ITT, Intent-to-Treat Analysis set; N, number of randomized subjects; n, number of subjects within each category; Outcome definition,For patients with baseline Perianal Disease Activity Index (PDAI) score >=4 follow up PDAI=0 indicates remmission; Proc Glimmix used to generate Odds Ratios and P-values.

Program Name: MUCUS-Shell6-V01-NC
Output Location: .\Output\Shared\MUCUS-TFLS-Final-27MAR24-V01.pdf

Table 14.2.8.2: Summary of Fistula Remission at Week 8, 16, 26, 36, 52

	Naive (N=13)	Biologic (N=22)	Multiple Biologic (N=17)
Fistulizing Crohns Remission			
Week 8			
N	-	2	3
[Remitter] n(%)	-	-	1 (33.3%)
[Non-Remitter] n(%)	-	2 (100.0%)	2 (66.7%)
[Remitter] Odds Ratio Strata 1 vs Strata 2 or 3		-	-
Odds Ratio 95% CI		-	-
Odds Ratio P-value		-	-
[Remitter] Odds Ratio Strata 2 vs Strata 3			<0.001
Odds Ratio 95% CI			(NE, NE)
Odds Ratio P-value			<.001
Week 16			
N	-	2	3
[Remitter] n(%)	-	-	1 (33.3%)
[Non-Remitter] n(%)	-	2 (100.0%)	2 (66.7%)
[Remitter] Odds Ratio Strata 1 vs Strata 2 or 3		-	-
Odds Ratio 95% CI		-	-
Odds Ratio P-value		-	-
[Remitter] Odds Ratio Strata 2 vs Strata 3			<0.001
Odds Ratio 95% CI			(NE, NE)
Odds Ratio P-value			<.001
Week 26			
N	-	-	-
[Remitter] n(%)	-	-	-
[Non-Remitter] n(%)	-	-	-
[Remitter] Odds Ratio Strata 1 vs Strata 2 or 3		-	-

Abbreviations: ITT, Intent-to-Treat Analysis set; N, number of randomized subjects; n, number of subjects within each category; Outcome definition,For patients with baseline Perianal Disease Activity Index (PDAI) score >=4 a follow up 0<PDAI<4 indicates response ; Proc Glimmix used to generate Odds Ratios and P-values.

Program Name: MUCUS-Shell6-V01-NC
Output Location: .\Output\Shared\MUCUS-TFLS-Final-27MAR24-V01.pdf

Table 14.2.8.2: Summary of Fistula Remission at Week 8, 16, 26, 36, 52

	Naive (N=13)	Biologic (N=22)	Multiple Biologic (N=17)
Odds Ratio 95% CI		-	-
Odds Ratio P-value		-	-
[Remitter] Odds Ratio Strata 2 vs Strata 3			-
Odds Ratio 95% CI			-
Odds Ratio P-value			-
Week 36			
N	-	-	-
[Remitter] n(%)	-	-	-
[Non-Remitter] n(%)	-	-	-
[Remitter] Odds Ratio Strata 1 vs Strata 2 or 3		-	-
Odds Ratio 95% CI		-	-
Odds Ratio P-value		-	-
[Remitter] Odds Ratio Strata 2 vs Strata 3			-
Odds Ratio 95% CI			-
Odds Ratio P-value			-
Week 52			
N	-	1	3
[Remitter] n(%)	-	-	1 (33.3%)
[Non-Remitter] n(%)	-	1 (100.0%)	2 (66.7%)
[Remitter] Odds Ratio Strata 1 vs Strata 2 or 3		-	-
Odds Ratio 95% CI		-	-
Odds Ratio P-value		-	-
[Remitter] Odds Ratio Strata 2 vs Strata 3			<0.001
Odds Ratio 95% CI			(NE, NE)
Odds Ratio P-value			<.001

Abbreviations: ITT, Intent-to-Treat Analysis set; N, number of randomized subjects; n, number of subjects within each category; Outcome definition,For patients with baseline Perianal Disease Activity Index (PDAI) score >=4 a follow up 0<PDAI<4 indicates response ; Proc Glimmix used to generate Odds Ratios and P-values.

Program Name: MUCUS-Shell6-V01-NC
Output Location: .\Output\Shared\MUCUS-TFLS-Final-27MAR24-V01.pdf

Table 14.2.9: Summary of Mean Change in PDAI Score from Baseline at Week 8, 16, 26, 36, 52

	Naive (N=13)	Biologic (N=22)	Multiple Biologic (N=17)
Perianal Disease Activity Index			
Baseline: OBS			
N	13	22	16
Missing	0	0	1
Mean (SD)	0.0 (0.00)	0.9 (3.12)	2.7 (4.98)
Median (min, max)	0.0 (0, 0)	0.0 (0, 14)	0.0 (0, 17)
Week 8: OBS			
N	12	21	14
Missing	1	1	3
Mean (SD)	0.2 (0.58)	0.8 (3.06)	1.2 (3.47)
Median (min, max)	0.0 (0, 2)	0.0 (0, 14)	0.0 (0, 13)
Week 8: CHG			
N	12	21	13
Missing	1	1	4
Mean (SD)	0.2 (0.58)	-0.1 (0.65)	-1.2 (2.44)
Median (min, max)	0.0 (0, 2)	0.0 (-3, 0)	0.0 (-8, 0)
LS Means (SE)	0.2 (0.57)	-0.1 (0.43)	-1.2 (0.55)
LS Means Strata 1 vs (2 or 3) (95% CI) P-Value		0.3 (-1.1, 1.7) 0.668	1.3 (-0.3, 2.9) 0.101
LS Means Strata 2 vs 3 (95% CI) P-Value			1.0 (-0.4, 2.4) 0.153
Week 16: OBS			
N	9	19	13
Missing	4	3	4
Mean (SD)	0.6 (1.67)	0.9 (2.42)	1.1 (2.56)
Median (min, max)	0.0 (0, 5)	0.0 (0, 9)	0.0 (0, 9)
Week 16: CHG			
N	9	19	12
Missing	4	3	5
Mean (SD)	0.6 (1.67)	-0.1 (1.97)	-1.6 (3.12)
Median (min, max)	0.0 (0, 5)	0.0 (-5, 6)	0.0 (-8, 0)
LS Means (SE)	0.6 (0.60)	-0.1 (0.44)	-1.5 (0.56)

Abbreviations: ITT, Intent-to-Treat Analysis set; N, number of randomized subjects; n, number of subjects within each category; SD, Standard Deviation; min, minimum; max, maximum; SE, Standard Error; PDAI, Perianal Disease Activity Index; Proc Mixed used to generate LS Means and P-values.

Program Name: MUCUS-Shell7-V01-NC
Output Location: .\Output\Shared\MUCUS-TFLS-Final-27MAR24-V01.pdf

Table 14.2.9: Summary of Mean Change in PDAI Score from Baseline at Week 8, 16, 26, 36, 52

	Naive (N=13)	Biologic (N=22)	Multiple Biologic (N=17)
LS Means Strata 1 vs (2 or 3) (95% CI) P-Value		0.7 (-0.8, 2.2) 0.371	2.0 (0.4, 3.7) 0.015
LS Means Strata 2 vs 3 (95% CI) P-Value			1.4 (-0.0, 2.8) 0.058
Week 26: OBS			
N	7	13	7
Missing	6	9	10
Mean (SD)	0.0 (0.00)	0.5 (1.94)	0.0 (0.00)
Median (min, max)	0.0 (0, 0)	0.0 (0, 7)	0.0 (0, 0)
Week 26: CHG			
N	7	13	7
Missing	6	9	10
Mean (SD)	0.0 (0.00)	0.5 (1.94)	0.0 (0.00)
Median (min, max)	0.0 (0, 0)	0.0 (0, 7)	0.0 (0, 0)
LS Means (SE)	0.2 (0.64)	0.3 (0.48)	-1.3 (0.63)
LS Means Strata 1 vs (2 or 3) (95% CI) P-Value		-0.1 (-1.6, 1.5) 0.946	1.5 (-0.3, 3.3) 0.092
LS Means Strata 2 vs 3 (95% CI) P-Value			1.6 (0.0, 3.1) 0.048
Week 36: OBS			
N	8	10	9
Missing	5	12	8
Mean (SD)	0.0 (0.00)	0.0 (0.00)	0.0 (0.00)
Median (min, max)	0.0 (0, 0)	0.0 (0, 0)	0.0 (0, 0)
Week 36: CHG			
N	8	10	9
Missing	5	12	8
Mean (SD)	0.0 (0.00)	0.0 (0.00)	0.0 (0.00)
Median (min, max)	0.0 (0, 0)	0.0 (0, 0)	0.0 (0, 0)
LS Means (SE)	0.1 (0.62)	-0.4 (0.51)	-1.3 (0.59)
LS Means Strata 1 vs (2 or 3) (95% CI) P-Value		0.5 (-1.1, 2.1) 0.546	1.4 (-0.3, 3.2) 0.095
LS Means Strata 2 vs 3 (95% CI) P-Value			1.0 (-0.6, 2.5) 0.221
Week 52: OBS			
N	9	17	12

Abbreviations: ITT, Intent-to-Treat Analysis set; N, number of randomized subjects; n, number of subjects within each category; SD, Standard Deviation; min, minimum; max, maximum; SE, Standard Error; PDAI, Perianal Disease Activity Index; Proc Mixed used to generate LS Means and P-values.

Table 14.2.9: Summary of Mean Change in PDAI Score from Baseline at Week 8, 16, 26, 36, 52

	Naive (N=13)	Biologic (N=22)	Multiple Biologic (N=17)
Missing	4	5	5
Mean (SD)	0.0 (0.00)	0.2 (0.73)	0.7 (1.23)
Median (min, max)	0.0 (0, 0)	0.0 (0, 3)	0.0 (0, 3)
Week 52: CHG			
N	9	17	11
Missing	4	5	6
Mean (SD)	0.0 (0.00)	-0.1 (0.49)	-2.2 (4.60)
Median (min, max)	0.0 (0, 0)	0.0 (-2, 0)	0.0 (-14, 0)
LS Means (SE)	0.1 (0.61)	-0.2 (0.45)	-2.0 (0.57)
LS Means Strata 1 vs (2 or 3) (95% CI) P-Value		0.4 (-1.1, 1.9) 0.627	2.1 (0.4, 3.8) 0.013
LS Means Strata 2 vs 3 (95% CI) P-Value			1.7 (0.3, 3.2) 0.019

Abbreviations: ITT, Intent-to-Treat Analysis set; N, number of randomized subjects; n, number of subjects within each category; SD, Standard Deviation; min, minimum; max, maximum; SE, Standard Error; PDAI, Perianal Disease Activity Index; Proc Mixed used to generate LS Means and P-values.

Program Name: MUCUS-Shell7-V01-NC
Output Location: .\Output\Shared\MUCUS-TFLS-Final-27MAR24-V01.pdf

Table 14.2.10: Summary of Mean Change in HBI Index from Baseline at Week 8, 16, 26, 36, 52

	Naive (N=13)	Biologic (N=22)	Multiple Biologic (N=17)
Harvey Bradshaw Index			
Baseline: OBS			
N	13	21	16
Missing	0	1	1
Mean (SD)	9.4 (4.84)	8.8 (3.34)	10.4 (5.02)
Median (min, max)	8.0 (5, 23)	7.0 (3, 15)	8.5 (5, 19)
Week 8: OBS			
N	12	21	14
Missing	1	1	3
Mean (SD)	4.4 (2.11)	5.6 (5.89)	3.6 (2.47)
Median (min, max)	4.0 (2, 8)	4.0 (1, 25)	3.5 (1, 9)
Week 8: CHG			
N	12	20	13
Missing	1	2	4
Mean (SD)	-4.8 (4.26)	-3.3 (5.79)	-5.9 (5.24)
Median (min, max)	-2.5 (-16, -2)	-4.5 (-13, 10)	-4.0 (-17, -1)
LS Means (SE)	-4.8 (1.44)	-3.3 (1.12)	-5.9 (1.38)
LS Means Strata 1 vs (2 or 3) (95% CI) P-Value		-1.5 (-5.2, 2.1) 0.403	1.1 (-2.9, 5.1) 0.587
LS Means Strata 2 vs 3 (95% CI) P-Value			2.6 (-0.9, 6.2) 0.145
Week 16: OBS			
N	9	19	13
Missing	4	3	4
Mean (SD)	3.4 (4.45)	5.3 (4.89)	3.7 (3.25)
Median (min, max)	2.0 (1, 15)	5.0 (0, 19)	3.0 (0, 10)
Week 16: CHG			
N	9	18	12
Missing	4	4	5
Mean (SD)	-5.7 (1.94)	-3.7 (5.20)	-5.8 (5.94)
Median (min, max)	-5.0 (-8, -3)	-4.5 (-14, 5)	-4.5 (-17, 2)
LS Means (SE)	-5.4 (1.54)	-3.1 (1.15)	-5.4 (1.41)

Abbreviations: ITT, Intent-to-Treat Analysis set; N, number of randomized subjects; n, number of subjects within each category; SD, Standard Deviation; min, minimum; max, maximum; SE, Standard Error; HBI, Harvey Bradshaw Index ; Proc Mixed used to generate LS Means and P-values.

Program Name: MUCUS-Shell7-V01-NC
Output Location: .\Output\Shared\MUCUS-TFLS-Final-27MAR24-V01.pdf

Table 14.2.10: Summary of Mean Change in HBI Index from Baseline at Week 8, 16, 26, 36, 52

	Naive (N=13)	Biologic (N=22)	Multiple Biologic (N=17)
LS Means Strata 1 vs (2 or 3) (95% CI) P-Value		-2.3 (-6.1, 1.5) 0.232	-0.0 (-4.2, 4.1) 0.987
LS Means Strata 2 vs 3 (95% CI) P-Value			2.3 (-1.3, 5.9) 0.214
Week 26: OBS			
N	7	13	7
Missing	6	9	10
Mean (SD)	4.0 (2.38)	6.2 (5.75)	1.3 (0.76)
Median (min, max)	4.0 (1, 8)	5.0 (0, 22)	1.0 (0, 2)
Week 26: CHG			
N	7	12	7
Missing	6	10	10
Mean (SD)	-6.0 (4.32)	-2.7 (5.90)	-7.4 (4.86)
Median (min, max)	-5.0 (-15, -2)	-3.5 (-13, 11)	-5.0 (-17, -4)
LS Means (SE)	-5.6 (1.65)	-2.1 (1.27)	-6.7 (1.62)
LS Means Strata 1 vs (2 or 3) (95% CI) P-Value		-3.5 (-7.6, 0.6) 0.097	1.1 (-3.5, 5.7) 0.627
LS Means Strata 2 vs 3 (95% CI) P-Value			4.6 (0.5, 8.7) 0.027
Week 36: OBS			
N	8	10	9
Missing	5	12	8
Mean (SD)	3.9 (3.76)	4.4 (2.91)	2.6 (2.40)
Median (min, max)	3.5 (0, 12)	4.0 (0, 9)	3.0 (0, 6)
Week 36: CHG			
N	8	10	9
Missing	5	12	8
Mean (SD)	-5.3 (2.92)	-4.7 (5.36)	-5.7 (5.48)
Median (min, max)	-4.5 (-11, -2)	-3.5 (-14, 2)	-5.0 (-18, -1)
LS Means (SE)	-5.9 (1.59)	-4.5 (1.33)	-6.0 (1.51)
LS Means Strata 1 vs (2 or 3) (95% CI) P-Value		-1.4 (-5.5, 2.7) 0.504	0.1 (-4.2, 4.5) 0.952
LS Means Strata 2 vs 3 (95% CI) P-Value			1.5 (-2.5, 5.5) 0.451
Week 52: OBS			
N	9	16	12

Abbreviations: ITT, Intent-to-Treat Analysis set; N, number of randomized subjects; n, number of subjects within each category; SD, Standard Deviation; min, minimum; max, maximum; SE, Standard Error; HBI, Harvey Bradshaw Index ; Proc Mixed used to generate LS Means and P-values.

Program Name: MUCUS-Shell7-V01-NC
Output Location: .\Output\Shared\MUCUS-TFLS-Final-27MAR24-V01.pdf

Table 14.2.10: Summary of Mean Change in HBI Index from Baseline at Week 8, 16, 26, 36, 52

	Naive (N=13)	Biologic (N=22)	Multiple Biologic (N=17)
Missing	4	6	5
Mean (SD)	3.3 (5.72)	3.9 (2.70)	3.8 (3.35)
Median (min, max)	1.0 (0, 16)	3.0 (0, 10)	2.5 (0, 11)
Week 52: CHG			
N	9	16	11
Missing	4	6	6
Mean (SD)	-6.9 (3.55)	-4.8 (4.11)	-5.6 (5.16)
Median (min, max)	-7.0 (-13, 0)	-4.5 (-11, 0)	-4.0 (-17, 0)
LS Means (SE)	-6.6 (1.54)	-4.8 (1.18)	-5.9 (1.43)
LS Means Strata 1 vs (2 or 3) (95% CI) P-Value		-1.8 (-5.7, 2.0) 0.352	-0.7 (-4.9, 3.5) 0.739
LS Means Strata 2 vs 3 (95% CI) P-Value			1.1 (-2.6, 4.8) 0.550

Abbreviations: ITT, Intent-to-Treat Analysis set; N, number of randomized subjects; n, number of subjects within each category; SD, Standard Deviation; min, minimum; max, maximum; SE, Standard Error; HBI, Harvey Bradshaw Index ; Proc Mixed used to generate LS Means and P-values.

Program Name: MUCUS-Shell7-V01-NC
Output Location: .\Output\Shared\MUCUS-TFLS-Final-27MAR24-V01.pdf

Table 14.2.11: Summary of Mean Change in SES-CD Score from Baseline at Week 26 and 52

	Naive (N=13)	Biologic (N=22)	Multiple Biologic (N=17)
Simple Endoscopic Score for Crohns Disease			
Baseline: OBS			
N	12	21	16
Missing	1	1	1
Mean (SD)	9.4 (5.43)	8.5 (4.91)	14.4 (6.35)
Median (min, max)	9.5 (3, 21)	7.0 (3, 20)	15.0 (5, 24)
Week 26: OBS			
N	1	-	-
Missing	12	-	-
Mean (SD)	3.0 (-)	-	-
Median (min, max)	3.0 (3, 3)	-	-
Week 26: CHG			
N	1	-	-
Missing	12	-	-
Mean (SD)	-8.0 (-)	-	-
Median (min, max)	-8.0 (-8, -8)	-	-
LS Means (SE)	-0.7 (3.07)	-	-
LS Means Strata 1 vs (2 or 3) (95% CI) P-Value		-	-
LS Means Strata 2 vs 3 (95% CI) P-Value			-
Week 52: OBS			
N	6	11	9
Missing	7	11	8
Mean (SD)	4.3 (5.89)	5.0 (3.61)	6.0 (7.89)
Median (min, max)	1.5 (0, 14)	6.0 (0, 9)	3.0 (0, 24)
Week 52: CHG			
N	6	11	9
Missing	7	11	8
Mean (SD)	-3.5 (5.86)	-4.6 (5.73)	-10.2 (8.51)
Median (min, max)	-3.0 (-11, 5)	-3.0 (-14, 3)	-11.0 (-23, 1)
LS Means (SE)	-3.5 (2.80)	-4.6 (2.07)	-10.2 (2.28)

Abbreviations: ITT, Intent-to-Treat Analysis set; N, number of randomized subjects; n, number of subjects within each category; SD, Standard Deviation; min, minimum; max, maximum; SE, Standard Error; SESCD, Simple Endoscopic Score for Crohns Disease; Proc Mixed used to generate LS Means and P-values.

Program Name: MUCUS-Shell7-V01-NC
Output Location: .\Output\Shared\MUCUS-TFLS-Final-27MAR24-V01.pdf

Table 14.2.11: Summary of Mean Change in SES-CD Score from Baseline at Week 26 and 52

	Naive (N=13)	Biologic (N=22)	Multiple Biologic (N=17)
LS Means Strata 1 vs (2 or 3) (95% CI) P-Value		1.1 (-6.1, 8.3) 0.747	6.7 (-0.7, 14.2) 0.075
LS Means Strata 2 vs 3 (95% CI) P-Value			5.6 (-0.8, 12.0) 0.083

Abbreviations: ITT, Intent-to-Treat Analysis set; N, number of randomized subjects; n, number of subjects within each category; SD, Standard Deviation; min, minimum; max, maximum; SE, Standard Error; SESCD, Simple Endoscopic Score for Crohns Disease; Proc Mixed used to generate LS Means and P-values.

Program Name: MUCUS-Shell7-V01-NC
Output Location: .\Output\Shared\MUCUS-TFLS-Final-27MAR24-V01.pdf

Table 14.2.12: Summary of Mean Change hs-CRP Concentration from Baseline at week 8, 16, 26, 36, 52

	Naive (N=13)	Biologic (N=22)	Multiple Biologic (N=17)
C-Reactive Protein			
Baseline: OBS			
N	13	22	17
Missing	0	0	0
Mean (SD)	18.1 (16.75)	19.8 (29.56)	35.3 (49.40)
Median (min, max)	12.6 (1, 52)	7.4 (1, 113)	25.9 (0, 210)
Week 8: OBS			
N	12	20	14
Missing	1	2	3
Mean (SD)	12.9 (18.55)	10.1 (12.24)	9.8 (9.56)
Median (min, max)	6.6 (1, 67)	5.8 (1, 48)	4.6 (1, 31)
Week 8: CHG			
N	12	20	14
Missing	1	2	3
Mean (SD)	-5.6 (19.74)	-11.2 (24.47)	-28.9 (46.50)
Median (min, max)	-1.5 (-47, 33)	-1.4 (-76, 17)	-15.7 (-180, 1)
LS Means (SE)	-5.6 (8.97)	-10.4 (6.82)	-28.9 (8.30)
LS Means Strata 1 vs (2 or 3) (95% CI) P-Value		4.8 (-17.8, 27.4) 0.674	23.2 (-1.3, 47.8) 0.063
LS Means Strata 2 vs 3 (95% CI) P-Value			18.5 (-3.1, 40.0) 0.092
Week 16: OBS			
N	8	18	12
Missing	5	4	5
Mean (SD)	7.2 (7.74)	8.5 (10.81)	16.6 (18.87)
Median (min, max)	5.0 (1, 21)	4.0 (1, 39)	6.6 (1, 58)
Week 16: CHG			
N	8	18	12
Missing	5	4	5
Mean (SD)	-4.7 (11.18)	-14.6 (26.86)	-25.6 (48.60)
Median (min, max)	-1.0 (-26, 8)	-2.6 (-81, 12)	-7.2 (-165, 15)
LS Means (SE)	-7.5 (9.37)	-12.5 (6.91)	-23.3 (8.43)

Abbreviations: ITT, Intent-to-Treat Analysis set; N, number of randomized subjects; n, number of subjects within each category; SD, Standard Deviation; min, minimum; max, maximum; SE, Standard Error; hs-CRP, C-Reactive Protein ; Proc Mixed used to generate LS Means and P-values.

Program Name: MUCUS-Shell7-V01-NC
Output Location: .\Output\Shared\MUCUS-TFLS-Final-27MAR24-V01.pdf

Table 14.2.12: Summary of Mean Change hs-CRP Concentration from Baseline at week 8, 16, 26, 36, 52

	Naive (N=13)	Biologic (N=22)	Multiple Biologic (N=17)
LS Means Strata 1 vs (2 or 3) (95% CI) P-Value		4.9 (-18.4, 28.2) 0.673	15.8 (-9.4, 41.1) 0.214
LS Means Strata 2 vs 3 (95% CI) P-Value			10.9 (-11.0, 32.7) 0.322
Week 26: OBS			
N	6	10	6
Missing	7	12	11
Mean (SD)	1.8 (0.92)	24.0 (48.08)	8.6 (11.84)
Median (min, max)	1.9 (1, 3)	4.4 (1, 156)	4.5 (1, 33)
Week 26: CHG			
N	6	10	6
Missing	7	12	11
Mean (SD)	-10.4 (19.22)	1.6 (18.13)	-16.6 (23.54)
Median (min, max)	-1.7 (-49, 1)	-0.9 (-31, 43)	-5.4 (-50, 7)
LS Means (SE)	-9.4 (9.76)	0.3 (7.46)	-26.3 (9.26)
LS Means Strata 1 vs (2 or 3) (95% CI) P-Value		-9.8 (-34.3, 14.7) 0.429	16.9 (-9.9, 43.7) 0.213
LS Means Strata 2 vs 3 (95% CI) P-Value			26.7 (3.0, 50.4) 0.028
Week 36: OBS			
N	8	9	9
Missing	5	13	8
Mean (SD)	8.5 (11.28)	7.5 (13.66)	9.3 (11.09)
Median (min, max)	3.1 (1, 31)	4.0 (1, 44)	4.0 (1, 34)
Week 36: CHG			
N	8	9	9
Missing	5	13	8
Mean (SD)	-5.2 (6.45)	-12.7 (23.94)	-12.4 (19.92)
Median (min, max)	-3.3 (-19, 1)	-3.0 (-69, 3)	-5.6 (-50, 8)
LS Means (SE)	-9.2 (9.39)	-12.8 (7.58)	-26.5 (8.73)
LS Means Strata 1 vs (2 or 3) (95% CI) P-Value		3.6 (-20.5, 27.7) 0.765	17.3 (-8.3, 42.9) 0.182
LS Means Strata 2 vs 3 (95% CI) P-Value			13.7 (-9.4, 36.7) 0.241
Week 52: OBS			
N	9	13	10

Abbreviations: ITT, Intent-to-Treat Analysis set; N, number of randomized subjects; n, number of subjects within each category; SD, Standard Deviation; min, minimum; max, maximum; SE, Standard Error; hs-CRP, C-Reactive Protein ; Proc Mixed used to generate LS Means and P-values.

Program Name: MUCUS-Shell7-V01-NC
Output Location: .\Output\Shared\MUCUS-TFLS-Final-27MAR24-V01.pdf

Table 14.2.12: Summary of Mean Change hs-CRP Concentration from Baseline at week 8, 16, 26, 36, 52

	Naive (N=13)	Biologic (N=22)	Multiple Biologic (N=17)
Missing	4	9	7
Mean (SD)	7.6 (7.98)	4.6 (5.04)	9.9 (9.96)
Median (min, max)	5.4 (1, 21)	3.3 (1, 19)	4.1 (1, 28)
Week 52: CHG			
N	9	13	10
Missing	4	9	7
Mean (SD)	-14.9 (15.91)	-21.5 (33.52)	-21.5 (18.97)
Median (min, max)	-6.1 (-42, -1)	-3.0 (-94, 2)	-22.7 (-50, 3)
LS Means (SE)	-11.3 (9.25)	-16.0 (7.18)	-28.0 (8.60)
LS Means Strata 1 vs (2 or 3) (95% CI) P-Value		4.7 (-18.7, 28.1) 0.689	16.7 (-8.6, 42.0) 0.191
LS Means Strata 2 vs 3 (95% CI) P-Value			12.0 (-10.4, 34.4) 0.288

Abbreviations: ITT, Intent-to-Treat Analysis set; N, number of randomized subjects; n, number of subjects within each category; SD, Standard Deviation; min, minimum; max, maximum; SE, Standard Error; hs-CRP, C-Reactive Protein ; Proc Mixed used to generate LS Means and P-values.

Program Name: MUCUS-Shell7-V01-NC
Output Location: .\Output\Shared\MUCUS-TFLS-Final-27MAR24-V01.pdf

Table 14.2.13: Summary of Mean Change Albumin Concentration from Baseline at week 8, 16, 26, 36, 52

	Naive (N=13)	Biologic (N=22)	Multiple Biologic (N=17)
Albumin			
Baseline: OBS			
N	11	22	15
Missing	2	0	2
Mean (SD)	39.0 (7.01)	34.9 (10.12)	35.1 (5.60)
Median (min, max)	39.4 (23, 47)	37.9 (4, 49)	36.6 (21, 43)
Week 8: OBS			
N	12	17	13
Missing	1	5	4
Mean (SD)	40.1 (7.07)	39.4 (5.85)	39.2 (3.57)
Median (min, max)	43.4 (24, 47)	39.6 (29, 51)	39.5 (31, 44)
Week 8: CHG			
N	10	17	12
Missing	3	5	5
Mean (SD)	1.7 (3.52)	5.9 (9.73)	4.4 (5.29)
Median (min, max)	1.3 (-2, 9)	2.6 (-3, 39)	5.0 (-4, 17)
LS Means (SE)	1.7 (2.28)	5.0 (1.60)	4.3 (2.01)
LS Means Strata 1 vs (2 or 3) (95% CI) P-Value		-3.2 (-8.8, 2.4) 0.254	-2.5 (-8.7, 3.6) 0.409
LS Means Strata 2 vs 3 (95% CI) P-Value			0.7 (-4.5, 5.9) 0.790
Week 16: OBS			
N	8	16	11
Missing	5	6	6
Mean (SD)	39.3 (9.85)	40.2 (4.64)	37.8 (3.66)
Median (min, max)	44.0 (21, 46)	41.2 (28, 45)	38.1 (31, 43)
Week 16: CHG			
N	7	16	10
Missing	6	6	7
Mean (SD)	0.3 (2.21)	4.6 (6.37)	3.7 (5.23)
Median (min, max)	-0.5 (-2, 3)	3.6 (-5, 20)	1.5 (-2, 14)
LS Means (SE)	1.9 (2.34)	5.5 (1.61)	3.8 (2.04)

Abbreviations: ITT, Intent-to-Treat Analysis set; N, number of randomized subjects; n, number of subjects within each category; SD, Standard Deviation; min, minimum; max, maximum; SE, Standard Error; Proc Mixed used to generate LS Means and P-values.

Program Name: MUCUS-Shell7-V01-NC
Output Location: .\Output\Shared\MUCUS-TFLS-Final-27MAR24-V01.pdf

Table 14.2.13: Summary of Mean Change Albumin Concentration from Baseline at week 8, 16, 26, 36, 52

	Naive (N=13)	Biologic (N=22)	Multiple Biologic (N=17)
LS Means Strata 1 vs (2 or 3) (95% CI) P-Value		-3.6 (-9.3, 2.1) 0.208	-1.9 (-8.1, 4.4) 0.546
LS Means Strata 2 vs 3 (95% CI) P-Value			1.7 (-3.5, 7.0) 0.507
Week 26: OBS			
N	6	9	6
Missing	7	13	11
Mean (SD)	44.4 (3.22)	39.2 (7.33)	39.8 (6.08)
Median (min, max)	44.7 (40, 49)	41.3 (23, 48)	39.5 (32, 49)
Week 26: CHG			
N	5	9	5
Missing	8	13	12
Mean (SD)	2.1 (3.23)	1.9 (3.69)	7.1 (5.26)
Median (min, max)	1.0 (-1, 8)	1.1 (-4, 9)	5.4 (2, 16)
LS Means (SE)	3.0 (2.42)	4.2 (1.71)	6.0 (2.22)
LS Means Strata 1 vs (2 or 3) (95% CI) P-Value		-1.2 (-7.1, 4.8) 0.691	-2.9 (-9.5, 3.7) 0.378
LS Means Strata 2 vs 3 (95% CI) P-Value			-1.7 (-7.3, 3.9) 0.539
Week 36: OBS			
N	6	9	7
Missing	7	13	10
Mean (SD)	42.9 (4.38)	41.2 (6.74)	40.5 (3.80)
Median (min, max)	43.8 (37, 47)	43.5 (27, 48)	39.3 (36, 46)
Week 36: CHG			
N	6	9	6
Missing	7	13	11
Mean (SD)	1.2 (1.73)	3.8 (4.20)	6.9 (3.59)
Median (min, max)	1.0 (-1, 4)	3.4 (-1, 12)	6.0 (3, 13)
LS Means (SE)	1.2 (2.38)	5.9 (1.71)	6.5 (2.16)
LS Means Strata 1 vs (2 or 3) (95% CI) P-Value		-4.7 (-10.6, 1.1) 0.111	-5.3 (-11.7, 1.2) 0.106
LS Means Strata 2 vs 3 (95% CI) P-Value			-0.5 (-6.1, 5.0) 0.845
Week 52: OBS			
N	9	12	7

Abbreviations: ITT, Intent-to-Treat Analysis set; N, number of randomized subjects; n, number of subjects within each category; SD, Standard Deviation; min, minimum; max, maximum; SE, Standard Error; Proc Mixed used to generate LS Means and P-values.

Table 14.2.13: Summary of Mean Change Albumin Concentration from Baseline at week 8, 16, 26, 36, 52

	Naive (N=13)	Biologic (N=22)	Multiple Biologic (N=17)
Missing	4	10	10
Mean (SD)	45.1 (2.97)	42.6 (4.68)	41.8 (5.83)
Median (min, max)	45.0 (39, 50)	43.4 (33, 49)	40.7 (33, 51)
Week 52: CHG			
N	7	12	6
Missing	6	10	11
Mean (SD)	4.9 (4.42)	9.7 (11.10)	7.1 (5.85)
Median (min, max)	3.0 (0, 12)	5.5 (-0, 39)	6.0 (2, 17)
LS Means (SE)	3.9 (2.35)	7.3 (1.66)	6.8 (2.15)
LS Means Strata 1 vs (2 or 3) (95% CI) P-Value		-3.4 (-9.2, 2.3) 0.236	-2.9 (-9.3, 3.5) 0.365
LS Means Strata 2 vs 3 (95% CI) P-Value			0.5 (-4.9, 6.0) 0.843

Abbreviations: ITT, Intent-to-Treat Analysis set; N, number of randomized subjects; n, number of subjects within each category; SD, Standard Deviation; min, minimum; max, maximum; SE, Standard Error; Proc Mixed used to generate LS Means and P-values.

Program Name: MUCUS-Shell7-V01-NC
Output Location: .\Output\Shared\MUCUS-TFLS-Final-27MAR24-V01.pdf

Table 14.2.14: Summary of Mean Change Fecal Calprotectin Concentration from Baseline at week 8, 16, 26, 36, 52

	Naive (N=13)	Biologic (N=22)	Multiple Biologic (N=17)
Fecal Calprotectin			
Baseline: OBS			
N	12	20	15
Missing	1	2	2
Mean (SD)	648.0 (791.44)	875.3 (954.21)	1170.5 (986.69)
Median (min, max)	247.0 (50, 2000)	584.5 (5, 4030)	800.0 (15, 3000)
Week 8: OBS			
N	10	13	12
Missing	3	9	5
Mean (SD)	226.8 (231.68)	382.9 (529.73)	773.4 (851.40)
Median (min, max)	177.5 (35, 804)	154.0 (50, 2000)	406.0 (28, 2100)
Week 8: CHG			
N	10	13	11
Missing	3	9	6
Mean (SD)	-343.1 (654.65)	-457.8 (1163.07)	-288.3 (493.39)
Median (min, max)	-75.3 (-1783, 305)	-127.0 (-3797, 562)	-22.0 (-1440, 286)
LS Means (SE)	-340.7 (257.78)	-503.8 (213.14)	-299.3 (246.75)
LS Means Strata 1 vs (2 or 3) (95% CI) P-Value		163.1 (-509.2, 835.4) 0.628	-41.4 (-759.3, 676.6) 0.908
LS Means Strata 2 vs 3 (95% CI) P-Value			-204.5 (-859.8, 450.9) 0.534
Week 16: OBS			
N	7	13	10
Missing	6	9	7
Mean (SD)	235.3 (216.03)	345.1 (533.37)	858.7 (727.58)
Median (min, max)	162.0 (74, 650)	206.0 (50, 2000)	723.7 (50, 2000)
Week 16: CHG			
N	7	13	8
Missing	6	9	9
Mean (SD)	12.8 (177.21)	-540.0 (1273.02)	-403.1 (587.14)
Median (min, max)	-7.0 (-182, 355)	-225.0 (-3824, 1795)	-50.0 (-1264, 119)
LS Means (SE)	-181.8 (271.06)	-516.9 (213.91)	-557.7 (258.00)

Abbreviations: ITT, Intent-to-Treat Analysis set; N, number of randomized subjects; n, number of subjects within each category; SD, Standard Deviation; min, minimum; max, maximum; SE, Standard Error; Proc Mixed used to generate LS Means and P-values.

Program Name: MUCUS-Shell7-V01-NC
Output Location: .\Output\Shared\MUCUS-TFLS-Final-27MAR24-V01.pdf

Table 14.2.14: Summary of Mean Change Fecal Calprotectin Concentration from Baseline at week 8, 16, 26, 36, 52

	Naive (N=13)	Biologic (N=22)	Multiple Biologic (N=17)
LS Means Strata 1 vs (2 or 3) (95% CI) P-Value		335.1 (-357.2, 1027.4) 0.336	376.0 (-374.0, 1125.9) 0.319
LS Means Strata 2 vs 3 (95% CI) P-Value			40.8 (-631.3, 713.0) 0.903
Week 26: OBS			
N	5	7	6
Missing	8	15	11
Mean (SD)	97.8 (69.53)	163.1 (90.43)	605.8 (667.57)
Median (min, max)	80.9 (20, 186)	185.0 (63, 298)	489.5 (50, 1800)
Week 26: CHG			
N	5	7	5
Missing	8	15	12
Mean (SD)	-116.0 (113.73)	-513.8 (585.30)	-164.2 (313.89)
Median (min, max)	-125.0 (-269, 50)	-459.0 (-1398, 293)	-44.0 (-724, 0)
LS Means (SE)	-240.7 (287.47)	-538.6 (238.99)	-264.5 (280.95)
LS Means Strata 1 vs (2 or 3) (95% CI) P-Value		297.9 (-448.1, 1043.9) 0.428	23.8 (-778.3, 826.0) 0.953
LS Means Strata 2 vs 3 (95% CI) P-Value			-274.1 (-1009.7, 461.5) 0.460
Week 36: OBS			
N	6	8	6
Missing	7	14	11
Mean (SD)	153.0 (147.79)	347.4 (592.43)	451.9 (493.80)
Median (min, max)	67.0 (50, 367)	166.0 (50, 1800)	268.0 (50, 1292)
Week 36: CHG			
N	6	8	5
Missing	7	14	12
Mean (SD)	-321.0 (689.78)	-690.0 (1366.23)	-459.6 (617.16)
Median (min, max)	-156.0 (-1663, 317)	-412.0 (-3760, 810)	-507.8 (-1317, 251)
LS Means (SE)	-375.6 (277.83)	-501.9 (231.82)	-387.2 (280.38)
LS Means Strata 1 vs (2 or 3) (95% CI) P-Value		126.3 (-596.9, 849.6) 0.728	11.6 (-776.8, 800.0) 0.977
LS Means Strata 2 vs 3 (95% CI) P-Value			-114.7 (-840.7, 611.3) 0.753
Week 52: OBS			
N	6	6	8

Abbreviations: ITT, Intent-to-Treat Analysis set; N, number of randomized subjects; n, number of subjects within each category; SD, Standard Deviation; min, minimum; max, maximum; SE, Standard Error; Proc Mixed used to generate LS Means and P-values.

Program Name: MUCUS-Shell7-V01-NC
Output Location: .\Output\Shared\MUCUS-TFLS-Final-27MAR24-V01.pdf

Table 14.2.14: Summary of Mean Change Fecal Calprotectin Concentration from Baseline at week 8, 16, 26, 36, 52

	Naive (N=13)	Biologic (N=22)	Multiple Biologic (N=17)
Missing	7	16	9
Mean (SD)	676.7 (773.45)	375.5 (598.72)	826.3 (789.84)
Median (min, max)	369.5 (50, 2000)	159.5 (5, 1584)	654.3 (14, 2000)
Week 52: CHG			
N	6	5	7
Missing	7	17	10
Mean (SD)	-194.3 (463.97)	-1278.2 (1510.93)	-308.1 (930.44)
Median (min, max)	-172.0 (-829, 521)	-674.0 (-3837, 77)	-100.0 (-1724, 917)
LS Means (SE)	-16.3 (277.99)	-866.0 (255.08)	-250.3 (263.71)
LS Means Strata 1 vs (2 or 3) (95% CI) P-Value		849.7 (97.2, 1602.2) 0.027	234.1 (-532.6, 1000.7) 0.544
LS Means Strata 2 vs 3 (95% CI) P-Value			-615.6 (-1347.5, 116.2) 0.098

Abbreviations: ITT, Intent-to-Treat Analysis set; N, number of randomized subjects; n, number of subjects within each category; SD, Standard Deviation; min, minimum; max, maximum; SE, Standard Error; Proc Mixed used to generate LS Means and P-values.

Program Name: MUCUS-Shell7-V01-NC
Output Location: .\Output\Shared\MUCUS-TFLS-Final-27MAR24-V01.pdf

Table 14.2.15: Summary of Mean Change in EQ-5D-5L from Baseline at Weeks 8, 16, 26, 36, 52

	Naive (N=13)	Biologic (N=22)	Multiple Biologic (N=17)
EuroQoL-5D-5L Health Questionnaire			
Baseline: OBS			
N	12	21	15
Missing	1	1	2
Mean (SD)	0.7 (0.30)	0.7 (0.25)	0.7 (0.39)
Median (min, max)	0.8 (0, 1)	0.8 (0, 1)	0.7 (-1, 1)
Week 8: OBS			
N	12	21	12
Missing	1	1	5
Mean (SD)	0.8 (0.13)	0.8 (0.18)	0.9 (0.10)
Median (min, max)	0.8 (1, 1)	0.8 (0, 1)	0.9 (1, 1)
Week 8: CHG			
N	11	20	11
Missing	2	2	6
Mean (SD)	0.1 (0.17)	0.1 (0.32)	0.2 (0.42)
Median (min, max)	0.1 (-0, 0)	0.0 (-1, 1)	0.1 (-0, 1)
LS Means (SE)	0.1 (0.09)	0.1 (0.07)	0.2 (0.09)
LS Means Strata 1 vs (2 or 3) (95% CI) P-Value		-0.1 (-0.3, 0.2) 0.632	-0.1 (-0.4, 0.2) 0.405
LS Means Strata 2 vs 3 (95% CI) P-Value			-0.1 (-0.3, 0.2) 0.644
Week 16: OBS			
N	9	19	12
Missing	4	3	5
Mean (SD)	0.9 (0.09)	0.7 (0.35)	0.9 (0.11)
Median (min, max)	0.9 (1, 1)	0.8 (-0, 1)	0.9 (1, 1)
Week 16: CHG			
N	9	18	11
Missing	4	4	6
Mean (SD)	0.1 (0.13)	0.0 (0.40)	0.2 (0.43)
Median (min, max)	0.1 (-0, 0)	0.0 (-1, 1)	0.1 (-0, 1)
LS Means (SE)	0.1 (0.10)	0.0 (0.07)	0.2 (0.09)

Abbreviations: ITT, Intent-to-Treat Analysis set; N, number of randomized subjects; n, number of subjects within each category; SD, Standard Deviation; min, minimum; max, maximum; SE, Standard Error; EQ-5D-5L, EuroQoL-5D-5L Health Questionnaire; Proc Mixed used to generate LS Means and P-values.

Program Name: MUCUS-Shell7-V01-NC
Output Location: .\Output\Shared\MUCUS-TFLS-Final-27MAR24-V01.pdf

Table 14.2.15: Summary of Mean Change in EQ-5D-5L from Baseline at Weeks 8, 16, 26, 36, 52

	Naive (N=13)	Biologic (N=22)	Multiple Biologic (N=17)
LS Means Strata 1 vs (2 or 3) (95% CI) P-Value		0.1 (-0.1, 0.3) 0.407	-0.1 (-0.3, 0.2) 0.594
LS Means Strata 2 vs 3 (95% CI) P-Value			-0.2 (-0.4, 0.1) 0.139
Week 26: OBS			
N	7	13	6
Missing	6	9	11
Mean (SD)	0.9 (0.10)	0.7 (0.32)	0.9 (0.10)
Median (min, max)	0.9 (1, 1)	0.8 (-0, 1)	1.0 (1, 1)
Week 26: CHG			
N	6	12	6
Missing	7	10	11
Mean (SD)	0.1 (0.07)	-0.0 (0.41)	0.1 (0.20)
Median (min, max)	0.1 (-0, 0)	-0.0 (-1, 1)	0.1 (0, 1)
LS Means (SE)	0.1 (0.10)	0.0 (0.08)	0.1 (0.10)
LS Means Strata 1 vs (2 or 3) (95% CI) P-Value		0.1 (-0.1, 0.4) 0.383	-0.0 (-0.3, 0.3) 0.811
LS Means Strata 2 vs 3 (95% CI) P-Value			-0.1 (-0.4, 0.1) 0.242
Week 36: OBS			
N	7	10	9
Missing	6	12	8
Mean (SD)	0.9 (0.13)	0.8 (0.22)	1.0 (0.05)
Median (min, max)	0.9 (1, 1)	0.8 (0, 1)	1.0 (1, 1)
Week 36: CHG			
N	7	10	9
Missing	6	12	8
Mean (SD)	0.1 (0.13)	0.1 (0.28)	0.1 (0.18)
Median (min, max)	0.1 (-0, 0)	0.1 (-0, 1)	0.1 (-0, 1)
LS Means (SE)	0.1 (0.10)	0.1 (0.08)	0.2 (0.09)
LS Means Strata 1 vs (2 or 3) (95% CI) P-Value		0.0 (-0.2, 0.3) 0.941	-0.1 (-0.3, 0.2) 0.689
LS Means Strata 2 vs 3 (95% CI) P-Value			-0.1 (-0.3, 0.2) 0.596
Week 52: OBS			
N	9	16	12

Abbreviations: ITT, Intent-to-Treat Analysis set; N, number of randomized subjects; n, number of subjects within each category; SD, Standard Deviation; min, minimum; max, maximum; SE, Standard Error; EQ-5D-5L, EuroQoL-5D-5L Health Questionnaire; Proc Mixed used to generate LS Means and P-values.

Program Name: MUCUS-Shell7-V01-NC
Output Location: .\Output\Shared\MUCUS-TFLS-Final-27MAR24-V01.pdf

Table 14.2.15: Summary of Mean Change in EQ-5D-5L from Baseline at Weeks 8, 16, 26, 36, 52

	Naive (N=13)	Biologic (N=22)	Multiple Biologic (N=17)
Missing	4	6	5
Mean (SD)	0.9 (0.12)	0.8 (0.30)	0.8 (0.21)
Median (min, max)	1.0 (1, 1)	0.8 (-0, 1)	0.9 (0, 1)
Week 52: CHG			
N	8	16	11
Missing	5	6	6
Mean (SD)	0.1 (0.16)	0.1 (0.31)	0.2 (0.30)
Median (min, max)	0.1 (-0, 0)	0.0 (-0, 1)	0.1 (-0, 1)
LS Means (SE)	0.1 (0.10)	0.1 (0.07)	0.2 (0.09)
LS Means Strata 1 vs (2 or 3) (95% CI) P-Value		0.1 (-0.2, 0.3) 0.509	-0.0 (-0.3, 0.2) 0.805
LS Means Strata 2 vs 3 (95% CI) P-Value			-0.1 (-0.3, 0.1) 0.321

Abbreviations: ITT, Intent-to-Treat Analysis set; N, number of randomized subjects; n, number of subjects within each category; SD, Standard Deviation; min, minimum; max, maximum; SE, Standard Error; EQ-5D-5L, EuroQoL-5D-5L Health Questionnaire; Proc Mixed used to generate LS Means and P-values.

Program Name: MUCUS-Shell7-V01-NC
Output Location: .\Output\Shared\MUCUS-TFLS-Final-27MAR24-V01.pdf

Table 14.2.16: Summary of Mean Change in PRO-2 from Baseline at Weeks 8, 16, 26, 36, 52

	Naive (N=13)	Biologic (N=22)	Multiple Biologic (N=17)
Patient Reported Outcomes-2			
Baseline: OBS			
N	11	22	13
Missing	2	0	4
Mean (SD)	89.3 (74.56)	113.5 (53.01)	135.6 (140.49)
Median (min, max)	67.0 (2, 278)	110.0 (21, 203)	76.0 (0, 520)
Week 8: OBS			
N	11	21	12
Missing	2	1	5
Mean (SD)	51.1 (40.27)	73.1 (64.48)	37.0 (38.97)
Median (min, max)	43.0 (0, 141)	56.0 (4, 202)	17.5 (2, 142)
Week 8: CHG			
N	10	21	10
Missing	3	1	7
Mean (SD)	-35.8 (40.38)	-42.4 (71.15)	-90.8 (160.38)
Median (min, max)	-24.0 (-137, -2)	-33.0 (-187, 130)	-34.5 (-505, 14)
LS Means (SE)	-35.2 (29.20)	-42.4 (20.99)	-82.3 (29.25)
LS Means Strata 1 vs (2 or 3) (95% CI) P-Value		7.2 (-65.2, 79.6) 0.842	47.1 (-36.0, 130.3) 0.260
LS Means Strata 2 vs 3 (95% CI) P-Value			39.9 (-32.6, 112.3) 0.274
Week 16: OBS			
N	9	19	12
Missing	4	3	5
Mean (SD)	55.6 (61.53)	71.7 (57.26)	42.8 (40.56)
Median (min, max)	35.0 (0, 206)	70.0 (0, 173)	27.0 (4, 148)
Week 16: CHG			
N	9	19	10
Missing	4	3	7
Mean (SD)	-38.6 (29.36)	-47.9 (79.18)	-85.9 (163.44)
Median (min, max)	-34.0 (-72, 11)	-59.0 (-199, 85)	-8.0 (-495, 17)
LS Means (SE)	-33.7 (29.43)	-36.1 (21.15)	-77.4 (29.25)

Abbreviations: ITT, Intent-to-Treat Analysis set; N, number of randomized subjects; n, number of subjects within each category; SD, Standard Deviation; min, minimum; max, maximum; SE, Standard Error; PRO-2, Patient Reported Outcomes-2; Proc Mixed used to generate LS Means and P-values.

Program Name: MUCUS-Shell7-V01-NC
Output Location: .\Output\Shared\MUCUS-TFLS-Final-27MAR24-V01.pdf

Table 14.2.16: Summary of Mean Change in PRO-2 from Baseline at Weeks 8, 16, 26, 36, 52

	Naive (N=13)	Biologic (N=22)	Multiple Biologic (N=17)
LS Means Strata 1 vs (2 or 3) (95% CI) P-Value		2.3 (-70.5, 75.2) 0.949	43.7 (-39.8, 127.1) 0.298
LS Means Strata 2 vs 3 (95% CI) P-Value			41.3 (-31.3, 113.9) 0.258
Week 26: OBS			
N	7	13	7
Missing	6	9	10
Mean (SD)	38.4 (46.28)	86.2 (62.92)	28.7 (18.38)
Median (min, max)	18.0 (4, 135)	84.0 (0, 204)	28.0 (0, 55)
Week 26: CHG			
N	6	13	6
Missing	7	9	11
Mean (SD)	-58.8 (51.45)	-38.5 (77.71)	-93.8 (183.10)
Median (min, max)	-49.5 (-143, -6)	-43.0 (-167, 70)	-24.5 (-465, 2)
LS Means (SE)	-56.9 (30.46)	-19.3 (21.78)	-76.2 (30.45)
LS Means Strata 1 vs (2 or 3) (95% CI) P-Value		-37.5 (-112.6, 37.5) 0.321	19.4 (-67.0, 105.7) 0.655
LS Means Strata 2 vs 3 (95% CI) P-Value			56.9 (-18.2, 132.0) 0.134
Week 36: OBS			
N	7	10	9
Missing	6	12	8
Mean (SD)	47.9 (62.65)	55.2 (31.44)	30.4 (25.08)
Median (min, max)	25.0 (6, 184)	47.0 (25, 119)	34.0 (0, 69)
Week 36: CHG			
N	7	10	8
Missing	6	12	9
Mean (SD)	-59.2 (30.81)	-68.0 (67.32)	-76.4 (175.30)
Median (min, max)	-65.0 (-96, -18)	-71.0 (-172, 10)	-13.5 (-505, 19)
LS Means (SE)	-58.5 (30.06)	-52.0 (22.33)	-82.8 (29.72)
LS Means Strata 1 vs (2 or 3) (95% CI) P-Value		-6.4 (-81.5, 68.7) 0.865	24.3 (-60.6, 109.2) 0.568
LS Means Strata 2 vs 3 (95% CI) P-Value			30.7 (-43.9, 105.3) 0.412
Week 52: OBS			
N	9	16	12

Abbreviations: ITT, Intent-to-Treat Analysis set; N, number of randomized subjects; n, number of subjects within each category; SD, Standard Deviation; min, minimum; max, maximum; SE, Standard Error; PRO-2, Patient Reported Outcomes-2; Proc Mixed used to generate LS Means and P-values.

Table 14.2.16: Summary of Mean Change in PRO-2 from Baseline at Weeks 8, 16, 26, 36, 52

	Naive (N=13)	Biologic (N=22)	Multiple Biologic (N=17)
Missing	4	6	5
Mean (SD)	43.5 (76.58)	59.1 (49.78)	51.6 (57.61)
Median (min, max)	10.0 (0, 225)	49.5 (0, 173)	36.5 (0, 176)
Week 52: CHG			
N	8	16	10
Missing	5	6	7
Mean (SD)	-52.8 (36.28)	-55.2 (67.40)	-81.6 (161.95)
Median (min, max)	-49.0 (-103, -2)	-28.5 (-203, 27)	-18.0 (-493, 19)
LS Means (SE)	-49.1 (29.69)	-49.9 (21.41)	-79.7 (29.17)
LS Means Strata 1 vs (2 or 3) (95% CI) P-Value		0.8 (-72.7, 74.3) 0.983	30.6 (-53.1, 114.2) 0.466
LS Means Strata 2 vs 3 (95% CI) P-Value			29.8 (-43.0, 102.5) 0.415

Abbreviations: ITT, Intent-to-Treat Analysis set; N, number of randomized subjects; n, number of subjects within each category; SD, Standard Deviation; min, minimum; max, maximum; SE, Standard Error; PRO-2, Patient Reported Outcomes-2; Proc Mixed used to generate LS Means and P-values.

Program Name: MUCUS-Shell7-V01-NC
Output Location: .\Output\Shared\MUCUS-TFLS-Final-27MAR24-V01.pdf

Table 14.2.17: Summary of PRO-2 Remission at Weeks 0, 8, 16, 26, 36, 52

	Naive (N=13)	Biologic (N=22)	Multiple Biologic (N=17)
PRO-2			
Baseline: OBS			
Remission (<8)	1 (7.7%)	-	1 (5.9%)
Mild (8-13)	-	-	-
Moderate (14-34)	1 (7.7%)	1 (4.5%)	1 (5.9%)
Severe (>=35)	9 (69.2%)	21 (95.5%)	11 (64.7%)
Week 8: OBS			
Remission (<8)	1 (7.7%)	1 (4.5%)	2 (11.8%)
Mild (8-13)	1 (7.7%)	2 (9.1%)	-
Moderate (14-34)	2 (15.4%)	6 (27.3%)	5 (29.4%)
Severe (>=35)	7 (53.8%)	12 (54.5%)	5 (29.4%)
Week 16: OBS			
Remission (<8)	1 (7.7%)	5 (22.7%)	1 (5.9%)
Mild (8-13)	-	-	1 (5.9%)
Moderate (14-34)	3 (23.1%)	-	6 (35.3%)
Severe (>=35)	5 (38.5%)	14 (63.6%)	4 (23.5%)
Week 26: OBS			
Remission (<8)	2 (15.4%)	1 (4.5%)	1 (5.9%)
Mild (8-13)	-	-	-
Moderate (14-34)	3 (23.1%)	1 (4.5%)	4 (23.5%)
Severe (>=35)	2 (15.4%)	11 (50.0%)	2 (11.8%)
Week 36: OBS			
Remission (<8)	1 (7.7%)	-	2 (11.8%)
Mild (8-13)	1 (7.7%)	-	1 (5.9%)
Moderate (14-34)	3 (23.1%)	4 (18.2%)	2 (11.8%)
Severe (>=35)	2 (15.4%)	6 (27.3%)	4 (23.5%)

Abbreviations: ITT, Intent-to-Treat Analysis set; N, number of randomized subjects; n, number of subjects within each category; SD, Standard Deviation; min, minimum; max, maximum; SE, Standard Error; PRO-2, Patient Reported Outcomes-2; Proc Mixed used to generate LS Means and P-values.

Program Name: MUCUS-Shell8-V01
Output Location: .\Output\Shared\MUCUS-TFLS-Final-27MAR24-V01.pdf

Table 14.2.17: Summary of PRO-2 Remission at Weeks 0, 8, 16, 26, 36, 52

	Naive (N=13)	Biologic (N=22)	Multiple Biologic (N=17)
Week 52: OBS			
Remission (<8)	4 (30.8%)	3 (13.6%)	3 (17.6%)
Mild (8-13)	1 (7.7%)	1 (4.5%)	-
Moderate (14-34)	2 (15.4%)	2 (9.1%)	2 (11.8%)
Severe (>=35)	2 (15.4%)	10 (45.5%)	7 (41.2%)

Abbreviations: ITT, Intent-to-Treat Analysis set; N, number of randomized subjects; n, number of subjects within each category; SD, Standard Deviation; min, minimum; max, maximum; SE, Standard Error; PRO-2, Patient Reported Outcomes-2; Proc Mixed used to generate LS Means and P-values.

Program Name: MUCUS-Shell8-V01
Output Location: .\Output\Shared\MUCUS-TFLS-Final-27MAR24-V01.pdf

Listing 14.3.1.1: Overall Summary of AEs

	Naive (N=13)	Biologic (N=22)	Multiple Biologic (N=17)
Total Number of AEs	55	46	48
Total Number of TEAEs	51	45	43
Subjects with >= 1 TEAE	11(84.6%)	14(63.6%)	11(64.7%)
Subjects with an TEAE Leading to Study Drug Discontinuation	2(15.4%)	2(9.1%)	1(5.9%)
Total Number of Serious TEAEs	5	11	2
Subjects with >= 1 Serious TEAE	3(23.1%)	6(27.3%)	2(11.8%)
Subjects with Serious TEAE Leading to Study Drug Discontinuation	2(15.4%)	1(4.5%)	1(5.9%)
Number of Subjects with >=1 TEAE by Maximum Treatment Relation			
Not Related	5(38.5%)	8(36.4%)	5(29.4%)
Unlikely	1(7.7%)	-	1(5.9%)
Possible	2(15.4%)	4(18.2%)	5(29.4%)
Probable	1(7.7%)	-	-
Definite	2(15.4%)	2(9.1%)	-
Number of Subjects with >=1 TEAE by Maximum Severity			
Mild	3(23.1%)	2(9.1%)	2(11.8%)
Moderate	5(38.5%)	9(40.9%)	7(41.2%)
Severe	3(23.1%)	3(13.6%)	2(11.8%)
Very Severe	-	-	-
Death	-	-	-

Abbreviations: N, number of randomized subjects; n, number of subjects within each category; TEAE, Treatment Emergent Adverse Events.

Program Name: MUCUS-Shell10-V01
Output Location: .\Output\Shared\MUCUS-TFLS-Final-27MAR24-V01.pdf

Table 14.3.1.2: Summary of TEAEs by SOC and PT

	Naive (N=13)	Biologic (N=22)	Multiple Biologic (N=17)
Subjects with >= 1 TEAE	11(84.6%)	14(63.6%)	11(64.7%)
Blood and lymphatic system disorders	-	1(4.5%)	1(5.9%)
Anaemia	-	1(4.5%)	-
Iron deficiency anaemia	-	1(4.5%)	1(5.9%)
Endocrine disorders	-	-	1(5.9%)
Adrenal insufficiency	-	-	1(5.9%)
Gastrointestinal disorders	8(61.5%)	7(31.8%)	5(29.4%)
Abdominal pain upper	1(7.7%)	-	-
Anal fissure	2(15.4%)	-	-
Anal fistula	-	-	1(5.9%)
Bile acid malabsorption	-	1(4.5%)	1(5.9%)
Colitis ulcerative	-	1(4.5%)	-
Constipation	2(15.4%)	-	-
Crohn's disease	1(7.7%)	5(22.7%)	3(17.6%)
Diarrhoea	1(7.7%)	-	-
Diarrhoea haemorrhagic	-	1(4.5%)	-
Enteritis	-	1(4.5%)	-
Gastrointestinal pain	-	1(4.5%)	-
Ileal stenosis	1(7.7%)	-	-
Inguinal hernia	-	1(4.5%)	-
Nausea	-	-	1(5.9%)
Pancreatitis	1(7.7%)	-	-
Subileus	1(7.7%)	-	-
Swollen tongue	-	-	1(5.9%)
Toothache	-	-	1(5.9%)
General disorders and administration site conditions	2(15.4%)	3(13.6%)	3(17.6%)
Drug ineffective	1(7.7%)	-	-
Injection site erythema	-	1(4.5%)	-
Oedema	-	-	1(5.9%)
Oedema peripheral	-	-	1(5.9%)

Abbreviations: N, number of randomized subjects; n, number of subjects within each category; TEAE, Treatment Emergent Adverse Events; SOC, System Organ Class; PT, Preferred Term.

Program Name: MUCUS-Shell10b-V01
Output Location: .\Output\Shared\MUCUS-TFLS-Final-27MAR24-V01.pdf

Table 14.3.1.2: Summary of TEAEs by SOC and PT

	Naive (N=13)	Biologic (N=22)	Multiple Biologic (N=17)
Peripheral swelling	-	-	1(5.9%)
Pyrexia	1(7.7%)	1(4.5%)	2(11.8%)
Sensation of foreign body	-	1(4.5%)	-
Hepatobiliary disorders	1(7.7%)	1(4.5%)	-
Cholangitis	1(7.7%)	-	-
Hepatic steatosis	-	1(4.5%)	-
Immune system disorders	-	1(4.5%)	-
Anaphylactic reaction	-	1(4.5%)	-
Infections and infestations	5(38.5%)	6(27.3%)	5(29.4%)
Abscess	-	1(4.5%)	-
COVID-19	1(7.7%)	2(9.1%)	-
Campylobacter infection	-	1(4.5%)	-
Clostridium difficile infection	-	-	1(5.9%)
Conjunctivitis	1(7.7%)	-	-
Gastrointestinal infection	-	-	1(5.9%)
Genitourinary chlamydia infection	1(7.7%)	-	-
Lyme disease	-	-	1(5.9%)
Nasopharyngitis	2(15.4%)	1(4.5%)	1(5.9%)
Norovirus infection	1(7.7%)	-	-
Oral herpes	1(7.7%)	1(4.5%)	-
Respiratory tract infection	1(7.7%)	-	-
Upper respiratory tract infection	-	1(4.5%)	-
Urinary tract infection	1(7.7%)	-	2(11.8%)
Injury, poisoning and procedural complications	1(7.7%)	2(9.1%)	-
Procedural pain	-	2(9.1%)	-
Seroma	1(7.7%)	-	-
Investigations	-	2(9.1%)	1(5.9%)
Blood prolactin increased	-	1(4.5%)	-
Haemoglobin decreased	-	-	1(5.9%)
Weight decreased	-	1(4.5%)	-

Abbreviations: N, number of randomized subjects; n, number of subjects within each category; TEAE, Treatment Emergent Adverse Events; SOC, System Organ Class; PT, Preferred Term.

Program Name: MUCUS-Shell10b-V01
Output Location: .\Output\Shared\MUCUS-TFLS-Final-27MAR24-V01.pdf

Table 14.3.1.2: Summary of TEAEs by SOC and PT

	Naive (N=13)	Biologic (N=22)	Multiple Biologic (N=17)
Metabolism and nutrition disorders	5(38.5%)	1(4.5%)	4(23.5%)
Folate deficiency	1(7.7%)	-	-
Hyperlipidaemia	-	-	1(5.9%)
Iron deficiency	1(7.7%)	-	3(17.6%)
Vitamin B12 deficiency	2(15.4%)	-	2(11.8%)
Vitamin D deficiency	2(15.4%)	1(4.5%)	1(5.9%)
Zinc deficiency	1(7.7%)	-	-
Musculoskeletal and connective tissue disorders	4(30.8%)	2(9.1%)	1(5.9%)
Arthralgia	2(15.4%)	2(9.1%)	-
Arthropathy	1(7.7%)	-	-
Fistula	1(7.7%)	-	-
Myalgia	1(7.7%)	-	-
Pain in extremity	-	-	1(5.9%)
Nervous system disorders	4(30.8%)	1(4.5%)	1(5.9%)
Ataxia	-	1(4.5%)	-
Dizziness	-	-	1(5.9%)
Headache	2(15.4%)	-	-
Migraine	1(7.7%)	-	-
Paraesthesia	1(7.7%)	-	-
Pregnancy, puerperium and perinatal conditions	1(7.7%)	1(4.5%)	-
Abortion missed	-	1(4.5%)	-
Pregnancy	1(7.7%)	1(4.5%)	-
Reproductive system and breast disorders	1(7.7%)	3(13.6%)	-
Cervical dysplasia	-	2(9.1%)	-
Dysmenorrhoea	1(7.7%)	-	-
Pruritus genital	-	1(4.5%)	-
Respiratory, thoracic and mediastinal disorders	-	-	1(5.9%)
Asthma	-	-	1(5.9%)
Chronic obstructive pulmonary disease	-	-	1(5.9%)
Skin and subcutaneous tissue disorders	4(30.8%)	3(13.6%)	4(23.5%)

Abbreviations: N, number of randomized subjects; n, number of subjects within each category; TEAE, Treatment Emergent Adverse Events; SOC, System Organ Class; PT, Preferred Term.

Program Name: MUCUS-Shell10b-V01
Output Location: .\Output\Shared\MUCUS-TFLS-Final-27MAR24-V01.pdf

Table 14.3.1.2: Summary of TEAEs by SOC and PT

	Naive (N=13)	Biologic (N=22)	Multiple Biologic (N=17)
Alopecia	1(7.7%)	-	1(5.9%)
Cold urticaria	-	1(4.5%)	-
Eczema	1(7.7%)	-	-
Hyperhidrosis	-	-	1(5.9%)
Perioral dermatitis	1(7.7%)	-	-
Photosensitivity reaction	1(7.7%)	-	-
Pruritus	-	-	1(5.9%)
Rash	1(7.7%)	1(4.5%)	1(5.9%)
Urticaria	-	1(4.5%)	1(5.9%)
Surgical and medical procedures	1(7.7%)	1(4.5%)	-
Anal fissure excision	-	1(4.5%)	-
Tooth extraction	1(7.7%)	-	-
Vascular disorders	1(7.7%)	1(4.5%)	-
Hypertension	-	1(4.5%)	-
Hypotension	1(7.7%)	-	-

Abbreviations: N, number of randomized subjects; n, number of subjects within each category; TEAE, Treatment Emergent Adverse Events; SOC, System Organ Class; PT, Preferred Term.

Program Name: MUCUS-Shell10b-V01
Output Location: .\Output\Shared\MUCUS-TFLS-Final-27MAR24-V01.pdf

Table 14.3.1.3: Summary of TEAEs by SOC, PT and Max Severity

	Naive (N=13)	Biologic (N=22)	Multiple Biologic (N=17)
Subjects with >= 1 TEAE	11(84.6%)	14(63.6%)	11(64.7%)
Blood and lymphatic system disorders	-	1(4.5%)	1(5.9%)
Anaemia	-	1(4.5%)	-
Mild	-	-	-
Moderate	-	-	-
Severe	-	1(4.5%)	-
Iron deficiency anaemia	-	1(4.5%)	1(5.9%)
Mild	-	-	1(5.9%)
Moderate	-	1(4.5%)	-
Severe	-	-	-
Endocrine disorders	-	-	1(5.9%)
Adrenal insufficiency	-	-	1(5.9%)
Mild	-	-	1(5.9%)
Moderate	-	-	-
Severe	-	-	-
Gastrointestinal disorders	8(61.5%)	7(31.8%)	5(29.4%)
Abdominal pain upper	1(7.7%)	-	-
Mild	1(7.7%)	-	-
Moderate	-	-	-
Severe	-	-	-
Anal fissure	2(15.4%)	-	-
Mild	1(7.7%)	-	-
Moderate	1(7.7%)	-	-
Severe	-	-	-
Anal fistula	-	-	1(5.9%)
Mild	-	-	1(5.9%)
Moderate	-	-	-
Severe	-	-	-
Bile acid malabsorption	-	1(4.5%)	1(5.9%)
Mild	-	-	-

Abbreviations: N, number of randomized subjects; n, number of subjects within each category; TEAE, Treatment Emergent Adverse Events; SOC, System Organ Class; PT, Preferred Term.

Program Name: MUCUS-Shell10c-V01
Output Location: .\Output\Shared\MUCUS-TFLS-Final-27MAR24-V01.pdf

Table 14.3.1.3: Summary of TEAEs by SOC, PT and Max Severity

	Naive (N=13)	Biologic (N=22)	Multiple Biologic (N=17)
Moderate	-	1(4.5%)	1(5.9%)
Severe	-	-	-
Colitis ulcerative	-	1(4.5%)	-
Mild	-	-	-
Moderate	-	1(4.5%)	-
Severe	-	-	-
Constipation	2(15.4%)	-	-
Mild	1(7.7%)	-	-
Moderate	1(7.7%)	-	-
Severe	-	-	-
Crohn's disease	1(7.7%)	5(22.7%)	3(17.6%)
Mild	-	1(4.5%)	-
Moderate	1(7.7%)	3(13.6%)	2(11.8%)
Severe	-	1(4.5%)	1(5.9%)
Diarrhoea	1(7.7%)	-	-
Mild	-	-	-
Moderate	1(7.7%)	-	-
Severe	-	-	-
Diarrhoea haemorrhagic	-	1(4.5%)	-
Mild	-	-	-
Moderate	-	1(4.5%)	-
Severe	-	-	-
Enteritis	-	1(4.5%)	-
Mild	-	1(4.5%)	-
Moderate	-	-	-
Severe	-	-	-
Gastrointestinal pain	-	1(4.5%)	-
Mild	-	-	-
Moderate	-	1(4.5%)	-
Severe	-	-	-

Abbreviations: N, number of randomized subjects; n, number of subjects within each category; TEAE, Treatment Emergent Adverse Events; SOC, System Organ Class; PT, Preferred Term.

Program Name: MUCUS-Shell10c-V01
Output Location: .\Output\Shared\MUCUS-TFLS-Final-27MAR24-V01.pdf

Table 14.3.1.3: Summary of TEAEs by SOC, PT and Max Severity

	Naive (N=13)	Biologic (N=22)	Multiple Biologic (N=17)
Ileal stenosis	1(7.7%)	-	-
Mild	-	-	-
Moderate	-	-	-
Severe	1(7.7%)	-	-
Inguinal hernia	-	1(4.5%)	-
Mild	-	-	-
Moderate	-	1(4.5%)	-
Severe	-	-	-
Nausea	-	-	1(5.9%)
Mild	-	-	1(5.9%)
Moderate	-	-	-
Severe	-	-	-
Pancreatitis	1(7.7%)	-	-
Mild	-	-	-
Moderate	1(7.7%)	-	-
Severe	-	-	-
Subileus	1(7.7%)	-	-
Mild	-	-	-
Moderate	-	-	-
Severe	1(7.7%)	-	-
Swollen tongue	-	-	1(5.9%)
Mild	-	-	1(5.9%)
Moderate	-	-	-
Severe	-	-	-
Toothache	-	-	1(5.9%)
Mild	-	-	1(5.9%)
Moderate	-	-	-
Severe	-	-	-
General disorders and administration site conditions	2(15.4%)	3(13.6%)	3(17.6%)
Drug ineffective	1(7.7%)	-	-

Abbreviations: N, number of randomized subjects; n, number of subjects within each category; TEAE, Treatment Emergent Adverse Events; SOC, System Organ Class; PT, Preferred Term.

Program Name: MUCUS-Shell10c-V01
Output Location: .\Output\Shared\MUCUS-TFLS-Final-27MAR24-V01.pdf

Table 14.3.1.3: Summary of TEAEs by SOC, PT and Max Severity

	Naive (N=13)	Biologic (N=22)	Multiple Biologic (N=17)
Mild	-	-	-
Moderate	-	-	-
Severe	1(7.7%)	-	-
Injection site erythema	-	1(4.5%)	-
Mild	-	1(4.5%)	-
Moderate	-	-	-
Severe	-	-	-
Oedema	-	-	1(5.9%)
Mild	-	-	1(5.9%)
Moderate	-	-	-
Severe	-	-	-
Oedema peripheral	-	-	1(5.9%)
Mild	-	-	-
Moderate	-	-	1(5.9%)
Severe	-	-	-
Peripheral swelling	-	-	1(5.9%)
Mild	-	-	1(5.9%)
Moderate	-	-	-
Severe	-	-	-
Pyrexia	1(7.7%)	1(4.5%)	2(11.8%)
Mild	-	-	1(5.9%)
Moderate	1(7.7%)	1(4.5%)	-
Severe	-	-	1(5.9%)
Sensation of foreign body	-	1(4.5%)	-
Mild	-	1(4.5%)	-
Moderate	-	-	-
Severe	-	-	-
Hepatobiliary disorders	1(7.7%)	1(4.5%)	-
Cholangitis	1(7.7%)	-	-
Mild	-	-	-

Abbreviations: N, number of randomized subjects; n, number of subjects within each category; TEAE, Treatment Emergent Adverse Events; SOC, System Organ Class; PT, Preferred Term.

Table 14.3.1.3: Summary of TEAEs by SOC, PT and Max Severity

	Naive (N=13)	Biologic (N=22)	Multiple Biologic (N=17)
Moderate	-	-	-
Severe	1(7.7%)	-	-
Hepatic steatosis	-	1(4.5%)	-
Mild	-	1(4.5%)	-
Moderate	-	-	-
Severe	-	-	-
Immune system disorders	-	1(4.5%)	-
Anaphylactic reaction	-	1(4.5%)	-
Mild	-	-	-
Moderate	-	1(4.5%)	-
Severe	-	-	-
Infections and infestations	5(38.5%)	6(27.3%)	5(29.4%)
Abscess	-	1(4.5%)	-
Mild	-	1(4.5%)	-
Moderate	-	-	-
Severe	-	-	-
COVID-19	1(7.7%)	2(9.1%)	-
Mild	1(7.7%)	2(9.1%)	-
Moderate	-	-	-
Severe	-	-	-
Campylobacter infection	-	1(4.5%)	-
Mild	-	1(4.5%)	-
Moderate	-	-	-
Severe	-	-	-
Clostridium difficile infection	-	-	1(5.9%)
Mild	-	-	1(5.9%)
Moderate	-	-	-
Severe	-	-	-
Conjunctivitis	1(7.7%)	-	-
Mild	1(7.7%)	-	-

Abbreviations: N, number of randomized subjects; n, number of subjects within each category; TEAE, Treatment Emergent Adverse Events; SOC, System Organ Class; PT, Preferred Term.

Program Name: MUCUS-Shell10c-V01
Output Location: .\Output\Shared\MUCUS-TFLS-Final-27MAR24-V01.pdf

Table 14.3.1.3: Summary of TEAEs by SOC, PT and Max Severity

	Naive (N=13)	Biologic (N=22)	Multiple Biologic (N=17)
Moderate	-	-	-
Severe	-	-	-
Gastrointestinal infection	-	-	1(5.9%)
Mild	-	-	-
Moderate	-	-	1(5.9%)
Severe	-	-	-
Genitourinary chlamydia infection	1(7.7%)	-	-
Mild	1(7.7%)	-	-
Moderate	-	-	-
Severe	-	-	-
Lyme disease	-	-	1(5.9%)
Mild	-	-	-
Moderate	-	-	1(5.9%)
Severe	-	-	-
Nasopharyngitis	2(15.4%)	1(4.5%)	1(5.9%)
Mild	1(7.7%)	1(4.5%)	1(5.9%)
Moderate	1(7.7%)	-	-
Severe	-	-	-
Norovirus infection	1(7.7%)	-	-
Mild	-	-	-
Moderate	-	-	-
Severe	1(7.7%)	-	-
Oral herpes	1(7.7%)	1(4.5%)	-
Mild	-	-	-
Moderate	1(7.7%)	1(4.5%)	-
Severe	-	-	-
Respiratory tract infection	1(7.7%)	-	-
Mild	-	-	-
Moderate	1(7.7%)	-	-
Severe	-	-	-

Abbreviations: N, number of randomized subjects; n, number of subjects within each category; TEAE, Treatment Emergent Adverse Events; SOC, System Organ Class; PT, Preferred Term.

Program Name: MUCUS-Shell10c-V01
Output Location: .\Output\Shared\MUCUS-TFLS-Final-27MAR24-V01.pdf

Table 14.3.1.3: Summary of TEAEs by SOC, PT and Max Severity

	Naive (N=13)	Biologic (N=22)	Multiple Biologic (N=17)
Upper respiratory tract infection	-	1(4.5%)	-
Mild	-	1(4.5%)	-
Moderate	-	-	-
Severe	-	-	-
Urinary tract infection	1(7.7%)	-	2(11.8%)
Mild	-	-	1(5.9%)
Moderate	1(7.7%)	-	1(5.9%)
Severe	-	-	-
Injury, poisoning and procedural complications	1(7.7%)	2(9.1%)	-
Procedural pain	-	2(9.1%)	-
Mild	-	1(4.5%)	-
Moderate	-	1(4.5%)	-
Severe	-	-	-
Seroma	1(7.7%)	-	-
Mild	-	-	-
Moderate	1(7.7%)	-	-
Severe	-	-	-
Investigations	-	2(9.1%)	1(5.9%)
Blood prolactin increased	-	1(4.5%)	-
Mild	-	1(4.5%)	-
Moderate	-	-	-
Severe	-	-	-
Haemoglobin decreased	-	-	1(5.9%)
Mild	-	-	-
Moderate	-	-	1(5.9%)
Severe	-	-	-
Weight decreased	-	1(4.5%)	-
Mild	-	1(4.5%)	-
Moderate	-	-	-
Severe	-	-	-

Abbreviations: N, number of randomized subjects; n, number of subjects within each category; TEAE, Treatment Emergent Adverse Events; SOC, System Organ Class; PT, Preferred Term.

Program Name: MUCUS-Shell10c-V01
Output Location: .\Output\Shared\MUCUS-TFLS-Final-27MAR24-V01.pdf

Table 14.3.1.3: Summary of TEAEs by SOC, PT and Max Severity

	Naive (N=13)	Biologic (N=22)	Multiple Biologic (N=17)
Metabolism and nutrition disorders	5(38.5%)	1(4.5%)	4(23.5%)
Folate deficiency	1(7.7%)	-	-
Mild	1(7.7%)	-	-
Moderate	-	-	-
Severe	-	-	-
Hyperlipidaemia	-	-	1(5.9%)
Mild	-	-	-
Moderate	-	-	1(5.9%)
Severe	-	-	-
Iron deficiency	1(7.7%)	-	3(17.6%)
Mild	-	-	1(5.9%)
Moderate	1(7.7%)	-	2(11.8%)
Severe	-	-	-
Vitamin B12 deficiency	2(15.4%)	-	2(11.8%)
Mild	2(15.4%)	-	1(5.9%)
Moderate	-	-	1(5.9%)
Severe	-	-	-
Vitamin D deficiency	2(15.4%)	1(4.5%)	1(5.9%)
Mild	1(7.7%)	1(4.5%)	1(5.9%)
Moderate	1(7.7%)	-	-
Severe	-	-	-
Zinc deficiency	1(7.7%)	-	-
Mild	1(7.7%)	-	-
Moderate	-	-	-
Severe	-	-	-
Musculoskeletal and connective tissue disorders	4(30.8%)	2(9.1%)	1(5.9%)
Arthralgia	2(15.4%)	2(9.1%)	-
Mild	1(7.7%)	1(4.5%)	-
Moderate	1(7.7%)	1(4.5%)	-
Severe	-	-	-

Abbreviations: N, number of randomized subjects; n, number of subjects within each category; TEAE, Treatment Emergent Adverse Events; SOC, System Organ Class; PT, Preferred Term.

Program Name: MUCUS-Shell10c-V01
Output Location: .\Output\Shared\MUCUS-TFLS-Final-27MAR24-V01.pdf

Table 14.3.1.3: Summary of TEAEs by SOC, PT and Max Severity

	Naive (N=13)	Biologic (N=22)	Multiple Biologic (N=17)
Arthropathy	1(7.7%)	-	-
Mild	1(7.7%)	-	-
Moderate	-	-	-
Severe	-	-	-
Fistula	1(7.7%)	-	-
Mild	-	-	-
Moderate	1(7.7%)	-	-
Severe	-	-	-
Myalgia	1(7.7%)	-	-
Mild	1(7.7%)	-	-
Moderate	-	-	-
Severe	-	-	-
Pain in extremity	-	-	1(5.9%)
Mild	-	-	1(5.9%)
Moderate	-	-	-
Severe	-	-	-
Nervous system disorders	4(30.8%)	1(4.5%)	1(5.9%)
Ataxia	-	1(4.5%)	-
Mild	-	-	-
Moderate	-	1(4.5%)	-
Severe	-	-	-
Dizziness	-	-	1(5.9%)
Mild	-	-	1(5.9%)
Moderate	-	-	-
Severe	-	-	-
Headache	2(15.4%)	-	-
Mild	-	-	-
Moderate	2(15.4%)	-	-
Severe	-	-	-
Migraine	1(7.7%)	-	-

Abbreviations: N, number of randomized subjects; n, number of subjects within each category; TEAE, Treatment Emergent Adverse Events; SOC, System Organ Class; PT, Preferred Term.

Program Name: MUCUS-Shell10c-V01
Output Location: .\Output\Shared\MUCUS-TFLS-Final-27MAR24-V01.pdf

Table 14.3.1.3: Summary of TEAEs by SOC, PT and Max Severity

	Naive (N=13)	Biologic (N=22)	Multiple Biologic (N=17)
Mild	-	-	-
Moderate	1(7.7%)	-	-
Severe	-	-	-
Paraesthesia	1(7.7%)	-	-
Mild	-	-	-
Moderate	1(7.7%)	-	-
Severe	-	-	-
Pregnancy, puerperium and perinatal conditions	1(7.7%)	1(4.5%)	-
Abortion missed	-	1(4.5%)	-
Mild	-	-	-
Moderate	-	-	-
Severe	-	1(4.5%)	-
Pregnancy	1(7.7%)	1(4.5%)	-
Mild	1(7.7%)	-	-
Moderate	-	-	-
Severe	-	1(4.5%)	-
Reproductive system and breast disorders	1(7.7%)	3(13.6%)	-
Cervical dysplasia	-	2(9.1%)	-
Mild	-	2(9.1%)	-
Moderate	-	-	-
Severe	-	-	-
Dysmenorrhoea	1(7.7%)	-	-
Mild	1(7.7%)	-	-
Moderate	-	-	-
Severe	-	-	-
Pruritus genital	-	1(4.5%)	-
Mild	-	-	-
Moderate	-	1(4.5%)	-
Severe	-	-	-
Respiratory, thoracic and mediastinal disorders	-	-	1(5.9%)

Abbreviations: N, number of randomized subjects; n, number of subjects within each category; TEAE, Treatment Emergent Adverse Events; SOC, System Organ Class; PT, Preferred Term.

Program Name: MUCUS-Shell10c-V01
Output Location: .\Output\Shared\MUCUS-TFLS-Final-27MAR24-V01.pdf

Table 14.3.1.3: Summary of TEAEs by SOC, PT and Max Severity

	Naive (N=13)	Biologic (N=22)	Multiple Biologic (N=17)
Asthma	-	-	1(5.9%)
Mild	-	-	-
Moderate	-	-	1(5.9%)
Severe	-	-	-
Chronic obstructive pulmonary disease	-	-	1(5.9%)
Mild	-	-	-
Moderate	-	-	1(5.9%)
Severe	-	-	-
Skin and subcutaneous tissue disorders	4(30.8%)	3(13.6%)	4(23.5%)
Alopecia	1(7.7%)	-	1(5.9%)
Mild	1(7.7%)	-	-
Moderate	-	-	1(5.9%)
Severe	-	-	-
Cold urticaria	-	1(4.5%)	-
Mild	-	-	-
Moderate	-	1(4.5%)	-
Severe	-	-	-
Eczema	1(7.7%)	-	-
Mild	1(7.7%)	-	-
Moderate	-	-	-
Severe	-	-	-
Hyperhidrosis	-	-	1(5.9%)
Mild	-	-	1(5.9%)
Moderate	-	-	-
Severe	-	-	-
Perioral dermatitis	1(7.7%)	-	-
Mild	-	-	-
Moderate	1(7.7%)	-	-
Severe	-	-	-
Photosensitivity reaction	1(7.7%)	-	-

Abbreviations: N, number of randomized subjects; n, number of subjects within each category; TEAE, Treatment Emergent Adverse Events; SOC, System Organ Class; PT, Preferred Term.

Program Name: MUCUS-Shell10c-V01
Output Location: .\Output\Shared\MUCUS-TFLS-Final-27MAR24-V01.pdf

Table 14.3.1.3: Summary of TEAEs by SOC, PT and Max Severity

	Naive (N=13)	Biologic (N=22)	Multiple Biologic (N=17)
Mild	1(7.7%)	-	-
Moderate	-	-	-
Severe	-	-	-
Pruritus	-	-	1(5.9%)
Mild	-	-	1(5.9%)
Moderate	-	-	-
Severe	-	-	-
Rash	1(7.7%)	1(4.5%)	1(5.9%)
Mild	1(7.7%)	1(4.5%)	1(5.9%)
Moderate	-	-	-
Severe	-	-	-
Urticaria	-	1(4.5%)	1(5.9%)
Mild	-	-	1(5.9%)
Moderate	-	1(4.5%)	-
Severe	-	-	-
Surgical and medical procedures	1(7.7%)	1(4.5%)	-
Anal fissure excision	-	1(4.5%)	-
Mild	-	1(4.5%)	-
Moderate	-	-	-
Severe	-	-	-
Tooth extraction	1(7.7%)	-	-
Mild	1(7.7%)	-	-
Moderate	-	-	-
Severe	-	-	-
Vascular disorders	1(7.7%)	1(4.5%)	-
Hypertension	-	1(4.5%)	-
Mild	-	1(4.5%)	-
Moderate	-	-	-
Severe	-	-	-
Hypotension	1(7.7%)	-	-

Abbreviations: N, number of randomized subjects; n, number of subjects within each category; TEAE, Treatment Emergent Adverse Events; SOC, System Organ Class; PT, Preferred Term.

Program Name: MUCUS-Shell10c-V01
Output Location: .\Output\Shared\MUCUS-TFLS-Final-27MAR24-V01.pdf

Table 14.3.1.3: Summary of TEAEs by SOC, PT and Max Severity

	Naive (N=13)	Biologic (N=22)	Multiple Biologic (N=17)
Mild	1(7.7%)	-	-
Moderate	-	-	-
Severe	-	-	-

Abbreviations: N, number of randomized subjects; n, number of subjects within each category; TEAE, Treatment Emergent Adverse Events; SOC, System Organ Class; PT, Preferred Term.

Program Name: MUCUS-Shell10c-V01
Output Location: .\Output\Shared\MUCUS-TFLS-Final-27MAR24-V01.pdf

Table 14.3.1.4: Summary of Severe (CTCAE Grade >= 3) TEAEs by SOC and PT

	Naive (N=13)	Biologic (N=22)	Multiple Biologic (N=17)
Subjects with >= 1 TEAE	3(23.1%)	3(13.6%)	2(11.8%)
Blood and lymphatic system disorders	-	1(4.5%)	-
Anaemia	-	1(4.5%)	-
Gastrointestinal disorders	1(7.7%)	1(4.5%)	1(5.9%)
Crohn's disease	-	1(4.5%)	1(5.9%)
Ileal stenosis	1(7.7%)	-	-
Subileus	1(7.7%)	-	-
General disorders and administration site conditions	1(7.7%)	-	1(5.9%)
Drug ineffective	1(7.7%)	-	-
Pyrexia	-	-	1(5.9%)
Hepatobiliary disorders	1(7.7%)	-	-
Cholangitis	1(7.7%)	-	-
Infections and infestations	1(7.7%)	-	-
Norovirus infection	1(7.7%)	-	-
Pregnancy, puerperium and perinatal conditions	-	1(4.5%)	-
Abortion missed	-	1(4.5%)	-
Pregnancy	-	1(4.5%)	-

Abbreviations: N, number of randomized subjects; n, number of subjects within each category; CTCAE, Common Terminology for Adverse Events; TEAE, Treatment Emergent Adverse Event; SOC, System Organ Class; PT, Preferred Term.

Program Name: MUCUS-Shell10b-V01
Output Location: .\Output\Shared\MUCUS-TFLS-Final-27MAR24-V01.pdf

Table 14.3.1.5: Summary of Related TEAEs by SOC and PT

	Naive (N=13)	Biologic (N=22)	Multiple Biologic (N=17)
Subjects with >= 1 TEAE	5(38.5%)	6(27.3%)	5(29.4%)
General disorders and administration site conditions	1(7.7%)	1(4.5%)	-
Drug ineffective	1(7.7%)	-	-
Injection site erythema	-	1(4.5%)	-
Infections and infestations	2(15.4%)	-	3(17.6%)
Gastrointestinal infection	-	-	1(5.9%)
Nasopharyngitis	-	-	1(5.9%)
Oral herpes	1(7.7%)	-	-
Respiratory tract infection	1(7.7%)	-	-
Urinary tract infection	-	-	1(5.9%)
Musculoskeletal and connective tissue disorders	-	1(4.5%)	-
Arthralgia	-	1(4.5%)	-
Pregnancy, puerperium and perinatal conditions	-	1(4.5%)	-
Abortion missed	-	1(4.5%)	-
Reproductive system and breast disorders	-	1(4.5%)	-
Pruritus genital	-	1(4.5%)	-
Skin and subcutaneous tissue disorders	3(23.1%)	1(4.5%)	2(11.8%)
Alopecia	1(7.7%)	-	-
Cold urticaria	-	1(4.5%)	-
Eczema	1(7.7%)	-	-
Photosensitivity reaction	1(7.7%)	-	-
Pruritus	-	-	1(5.9%)
Rash	1(7.7%)	-	1(5.9%)
Vascular disorders	-	1(4.5%)	-
Hypertension	-	1(4.5%)	-

Abbreviations: N, number of randomized subjects; n, number of subjects within each category; TEAE, Treatment Emergent Adverse Event; SOC, System Organ Class; PT, Preferred Term.

Program Name: MUCUS-Shell10b-V01
Output Location: .\Output\Shared\MUCUS-TFLS-Final-27MAR24-V01.pdf

Table 14.3.1.6: Summary of Serious TEAEs by SOC and PT

	Naive (N=13)	Biologic (N=22)	Multiple Biologic (N=17)
Subjects with >= 1 TEAE	3(23.1%)	6(27.3%)	2(11.8%)
Blood and lymphatic system disorders	-	1(4.5%)	-
Anaemia	-	1(4.5%)	-
Gastrointestinal disorders	1(7.7%)	3(13.6%)	1(5.9%)
Crohn's disease	-	3(13.6%)	1(5.9%)
Ileal stenosis	1(7.7%)	-	-
Subileus	1(7.7%)	-	-
General disorders and administration site conditions	1(7.7%)	1(4.5%)	1(5.9%)
Drug ineffective	1(7.7%)	-	-
Pyrexia	-	1(4.5%)	1(5.9%)
Hepatobiliary disorders	1(7.7%)	-	-
Cholangitis	1(7.7%)	-	-
Infections and infestations	1(7.7%)	1(4.5%)	-
Abscess	-	1(4.5%)	-
COVID-19	-	1(4.5%)	-
Norovirus infection	1(7.7%)	-	-
Injury, poisoning and procedural complications	-	1(4.5%)	-
Procedural pain	-	1(4.5%)	-
Nervous system disorders	-	1(4.5%)	-
Ataxia	-	1(4.5%)	-
Pregnancy, puerperium and perinatal conditions	-	1(4.5%)	-
Abortion missed	-	1(4.5%)	-
Pregnancy	-	1(4.5%)	-

Abbreviations: N, number of randomized subjects; n, number of subjects within each category; TEAE, Treatment Emergent Adverse Event; SOC, System Organ Class; PT, Preferred Term.

Program Name: MUCUS-Shell10b-V01
Output Location: .\Output\Shared\MUCUS-TFLS-Final-27MAR24-V01.pdf

Table 14.3.1.7: Summary of Related Serious TEAEs by SOC and PT

	Naive (N=13)	Biologic (N=22)	Multiple Biologic (N=17)
Subjects with >= 1 TEAE	1(7.7%)	1(4.5%)	-
General disorders and administration site conditions	1(7.7%)	-	-
Drug ineffective	1(7.7%)	-	-
Pregnancy, puerperium and perinatal conditions	-	1(4.5%)	-
Abortion missed	-	1(4.5%)	-

Abbreviations: N, number of randomized subjects; n, number of subjects within each category; TEAE, Treatment Emergent Adverse Event; SOC, System Organ Class; PT, Preferred Term.

Program Name: MUCUS-Shell10b-V01
Output Location: .\Output\Shared\MUCUS-TFLS-Final-27MAR24-V01.pdf

Table 14.3.1.8: Summary of Infusion Reaction TEAEs by SOC and PT

	Naive (N=13)	Biologic (N=22)	Multiple Biologic (N=17)
Subjects with >= 1 TEAE	-	-	-

Abbreviations: N, number of randomized subjects; n, number of subjects within each category; TEAE, Treatment Emergent Adverse Event; SOC, System Organ Class; PT, Preferred Term.

Program Name: MUCUS-Shell10b-V01
Output Location: .\Output\Shared\MUCUS-TFLS-Final-27MAR24-V01.pdf

Table 14.3.1.9: Summary of Injection-Site Reaction TEAEs by SOC and PT

	Naive (N=13)	Biologic (N=22)	Multiple Biologic (N=17)
Subjects with >= 1 TEAE	2(15.4%)	3(13.6%)	3(17.6%)
General disorders and administration site conditions	2(15.4%)	3(13.6%)	3(17.6%)
Drug ineffective	1(7.7%)	-	-
Injection site erythema	-	1(4.5%)	-
Oedema	-	-	1(5.9%)
Oedema peripheral	-	-	1(5.9%)
Peripheral swelling	-	-	1(5.9%)
Pyrexia	1(7.7%)	1(4.5%)	2(11.8%)
Sensation of foreign body	-	1(4.5%)	-

Abbreviations: N, number of randomized subjects; n, number of subjects within each category; TEAE, Treatment Emergent Adverse Event; SOC, System Organ Class; PT, Preferred Term.

Program Name: MUCUS-Shell10b-V01
Output Location: .\Output\Shared\MUCUS-TFLS-Final-27MAR24-V01.pdf

Table 14.3.1.10: Summary of Infection TEAEs by SOC and PT

	Naive (N=13)	Biologic (N=22)	Multiple Biologic (N=17)
Subjects with >= 1 TEAE	5(38.5%)	6(27.3%)	5(29.4%)
Infections and infestations	5(38.5%)	6(27.3%)	5(29.4%)
Abscess	-	1(4.5%)	-
COVID-19	1(7.7%)	2(9.1%)	-
Campylobacter infection	-	1(4.5%)	-
Clostridium difficile infection	-	-	1(5.9%)
Conjunctivitis	1(7.7%)	-	-
Gastrointestinal infection	-	-	1(5.9%)
Genitourinary chlamydia infection	1(7.7%)	-	-
Lyme disease	-	-	1(5.9%)
Nasopharyngitis	2(15.4%)	1(4.5%)	1(5.9%)
Norovirus infection	1(7.7%)	-	-
Oral herpes	1(7.7%)	1(4.5%)	-
Respiratory tract infection	1(7.7%)	-	-
Upper respiratory tract infection	-	1(4.5%)	-
Urinary tract infection	1(7.7%)	-	2(11.8%)

Abbreviations: N, number of randomized subjects; n, number of subjects within each category; TEAE, Treatment Emergent Adverse Event; SOC, System Organ Class; PT, Preferred Term.

Program Name: MUCUS-Shell10b-V01
Output Location: .\Output\Shared\MUCUS-TFLS-Final-27MAR24-V01.pdf

Table 14.3.1.11: Summary of Steroid Tapering at Week 0, 8 ,16, 26, 36, 52

	Naive (N=13)	Biologic (N=22)	Multiple Biologic (N=17)
Steroid (mg)			
Baseline: OBS			
N	6	5	9
Missing	7	17	8
Mean (SD)	28.1 (12.03)	25.0 (14.13)	33.0 (10.73)
Median (Min, Max)	30.0 (9, 40)	20.0 (10, 40)	40.0 (8, 40)
Week 8: OBS			
N	6	4	9
Missing	7	18	8
Mean (SD)	24.8 (17.79)	18.7 (16.50)	26.4 (16.15)
Median (Min, Max)	30.0 (0, 40)	17.5 (0, 40)	30.0 (0, 40)
Week 16: OBS			
N	4	4	8
Missing	9	18	9
Mean (SD)	12.5 (18.91)	13.7 (18.86)	15.0 (18.51)
Median (Min, Max)	5.0 (0, 40)	7.5 (0, 40)	5.0 (0, 40)
Week 26: OBS			
N	3	2	4
Missing	10	20	13
Mean (SD)	20.0 (19.98)	7.5 (10.61)	27.5 (18.92)
Median (Min, Max)	20.0 (0, 40)	7.5 (0, 15)	35.0 (0, 40)
Week 36: OBS			
N	3	2	5
Missing	10	20	12
Mean (SD)	13.3 (23.07)	20.0 (28.26)	22.0 (20.49)
Median (Min, Max)	0.0 (0, 40)	20.0 (0, 40)	30.0 (0, 40)
Week 52: OBS			
N	4	4	7
Missing	9	18	10
Mean (SD)	10.0 (19.98)	13.7 (18.86)	16.4 (19.30)
Median (Min, Max)	0.0 (0, 40)	7.5 (0, 40)	5.0 (0, 40)

Abbreviations: N, number of randomized subjects; n, number of subjects within each category.

Program Name: MUCUS-Shell11a-V01
Output Location: .\Output\Shared\MUCUS-TFLS-Final-27MAR24-V01.pdf

Table 14.3.1.12: Summary of Montreal Classification Phenotype at Week 0, 8 ,16, 26, 36, 52

	Naive (N=13)	Biologic (N=22)	Multiple Biologic (N=17)
Baseline			
Age at Diagnosis: (years)			
Below 16y	1 (7.7%)	3 (13.6%)	2 (11.8%)
Between 17 and 40y	10 (76.9%)	14 (63.6%)	14 (82.4%)
Above 40y	2 (15.4%)	5 (22.7%)	1 (5.9%)
Location:			
Ileal	2 (15.4%)	4 (18.2%)	1 (5.9%)
Colonic	5 (38.5%)	3 (13.6%)	7 (41.2%)
Ileocolonic	4 (30.8%)	11 (50.0%)	9 (52.9%)
Isolated Upper Disease	2 (15.4%)	4 (18.2%)	-
Behavior:			
Non-stricturing, non-penetrating	9 (69.2%)	12 (54.5%)	10 (58.8%)
Stricturing	2 (15.4%)	7 (31.8%)	3 (17.6%)
Penetrating	2 (15.4%)	3 (13.6%)	4 (23.5%)
Week 8			
Age at Diagnosis: (years)			
Below 16y	1 (7.7%)	3 (13.6%)	1 (5.9%)
Between 17 and 40y	10 (76.9%)	13 (59.1%)	12 (70.6%)
Above 40y	1 (7.7%)	5 (22.7%)	1 (5.9%)
Location:			
Ileal	2 (15.4%)	3 (13.6%)	1 (5.9%)
Colonic	4 (30.8%)	3 (13.6%)	5 (29.4%)
Ileocolonic	4 (30.8%)	11 (50.0%)	8 (47.1%)
Isolated Upper Disease	2 (15.4%)	4 (18.2%)	-
Behavior:			

Abbreviations: ITT, Intent-to-Treat Analysis set; N, number of randomized subjects; n, number of subjects within each category.

Program Name: MUCUS-Shell16-V01
Output Location: .\Output\Shared\MUCUS-TFLS-Final-27MAR24-V01.pdf

Table 14.3.1.12: Summary of Montreal Classification Phenotype at Week 0, 8 ,16, 26, 36, 52

	Naive (N=13)	Biologic (N=22)	Multiple Biologic (N=17)
Non-stricturing, non-penetrating	8 (61.5%)	11 (50.0%)	8 (47.1%)
Stricturing	1 (7.7%)	7 (31.8%)	4 (23.5%)
Penetrating	3 (23.1%)	3 (13.6%)	2 (11.8%)
Week 16			
Age at Diagnosis: (years)			
Below 16y	1 (7.7%)	3 (13.6%)	1 (5.9%)
Between 17 and 40y	8 (61.5%)	12 (54.5%)	10 (58.8%)
Above 40y	-	4 (18.2%)	2 (11.8%)
Location:			
Ileal	2 (15.4%)	2 (9.1%)	1 (5.9%)
Colonic	3 (23.1%)	2 (9.1%)	4 (23.5%)
Ileocolonic	3 (23.1%)	11 (50.0%)	7 (41.2%)
Isolated Upper Disease	1 (7.7%)	4 (18.2%)	1 (5.9%)
Behavior:			
Non-stricturing, non-penetrating	6 (46.2%)	11 (50.0%)	8 (47.1%)
Stricturing	1 (7.7%)	5 (22.7%)	2 (11.8%)
Penetrating	2 (15.4%)	3 (13.6%)	3 (17.6%)
Week 26			
Age at Diagnosis: (years)			
Below 16y	1 (7.7%)	2 (9.1%)	-
Between 17 and 40y	6 (46.2%)	8 (36.4%)	7 (41.2%)
Above 40y	-	3 (13.6%)	-
Location:			
Ileal	2 (15.4%)	2 (9.1%)	-
Colonic	2 (15.4%)	2 (9.1%)	3 (17.6%)

Abbreviations: ITT, Intent-to-Treat Analysis set; N, number of randomized subjects; n, number of subjects within each category.

Table 14.3.1.12: Summary of Montreal Classification Phenotype at Week 0, 8 ,16, 26, 36, 52

	Naive (N=13)	Biologic (N=22)	Multiple Biologic (N=17)
Ileocolonic	3 (23.1%)	8 (36.4%)	4 (23.5%)
Isolated Upper Disease	-	1 (4.5%)	-
Behavior:			
Non-stricturing, non-penetrating	5 (38.5%)	8 (36.4%)	5 (29.4%)
Stricturing	1 (7.7%)	5 (22.7%)	2 (11.8%)
Penetrating	1 (7.7%)	-	-
Week 36			
Age at Diagnosis: (years)			
Below 16y	1 (7.7%)	2 (9.1%)	-
Between 17 and 40y	6 (46.2%)	8 (36.4%)	9 (52.9%)
Above 40y	1 (7.7%)	-	-
Location:			
Ileal	2 (15.4%)	1 (4.5%)	1 (5.9%)
Colonic	3 (23.1%)	2 (9.1%)	4 (23.5%)
Ileocolonic	2 (15.4%)	7 (31.8%)	4 (23.5%)
Isolated Upper Disease	1 (7.7%)	-	-
Behavior:			
Non-stricturing, non-penetrating	5 (38.5%)	7 (31.8%)	7 (41.2%)
Stricturing	1 (7.7%)	3 (13.6%)	2 (11.8%)
Penetrating	2 (15.4%)	-	-
Week 52			
Age at Diagnosis: (years)			
Below 16y	1 (7.7%)	3 (13.6%)	1 (5.9%)
Between 17 and 40y	7 (53.8%)	11 (50.0%)	9 (52.9%)
Above 40y	2 (15.4%)	3 (13.6%)	2 (11.8%)

Abbreviations: ITT, Intent-to-Treat Analysis set; N, number of randomized subjects; n, number of subjects within each category.

Program Name: MUCUS-Shell16-V01
Output Location: .\Output\Shared\MUCUS-TFLS-Final-27MAR24-V01.pdf

Table 14.3.1.12: Summary of Montreal Classification Phenotype at Week 0, 8 ,16, 26, 36, 52

	Naive (N=13)	Biologic (N=22)	Multiple Biologic (N=17)
Location:			
Ileal	1 (7.7%)	3 (13.6%)	1 (5.9%)
Colonic	3 (23.1%)	1 (4.5%)	5 (29.4%)
Ileocolonic	5 (38.5%)	11 (50.0%)	5 (29.4%)
Isolated Upper Disease	1 (7.7%)	2 (9.1%)	1 (5.9%)
Behavior:			
Non-stricturing, non-penetrating	8 (61.5%)	9 (40.9%)	7 (41.2%)
Stricturing	1 (7.7%)	6 (27.3%)	2 (11.8%)
Penetrating	1 (7.7%)	2 (9.1%)	3 (17.6%)

Abbreviations: ITT, Intent-to-Treat Analysis set; N, number of randomized subjects; n, number of subjects within each category.

Program Name: MUCUS-Shell16-V01
Output Location: .\Output\Shared\MUCUS-TFLS-Final-27MAR24-V01.pdf

Table 14.3.1.13: Summary of Extraintestinal Manifestations at Week 0, 8 ,16, 26, 36, 52

	Naive (N=13)	Biologic (N=22)	Multiple Biologic (N=17)
Baseline			
Extraintestinal Manifestations	5 (38.5%)	5 (22.7%)	4 (23.5%)
Arthritis/ Arthralgia	5 (38.5%)	4 (18.2%)	4 (23.5%)
Iritis/ Uveitis	0 (0.0%)	0 (0.0%)	1 (5.9%)
Erythema nodosum	0 (0.0%)	1 (4.5%)	0 (0.0%)
Pyoderma gangrenosum	0 (0.0%)	0 (0.0%)	0 (0.0%)
Aphthous	0 (0.0%)	0 (0.0%)	0 (0.0%)
Week 8			
Extraintestinal Manifestations	3 (23.1%)	2 (9.1%)	3 (17.6%)
Arthritis/ Arthralgia	3 (23.1%)	2 (9.1%)	3 (17.6%)
Iritis/ Uveitis	0 (0.0%)	0 (0.0%)	0 (0.0%)
Erythema nodosum	0 (0.0%)	0 (0.0%)	0 (0.0%)
Pyoderma gangrenosum	0 (0.0%)	0 (0.0%)	0 (0.0%)
Aphthous	0 (0.0%)	0 (0.0%)	0 (0.0%)
Week 16			
Extraintestinal Manifestations	2 (15.4%)	3 (13.6%)	2 (11.8%)
Arthritis/ Arthralgia	2 (15.4%)	3 (13.6%)	2 (11.8%)
Iritis/ Uveitis	0 (0.0%)	0 (0.0%)	0 (0.0%)
Erythema nodosum	0 (0.0%)	0 (0.0%)	0 (0.0%)
Pyoderma gangrenosum	0 (0.0%)	0 (0.0%)	0 (0.0%)
Aphthous	0 (0.0%)	0 (0.0%)	0 (0.0%)
Week 26			
Extraintestinal Manifestations	3 (23.1%)	1 (4.5%)	1 (5.9%)
Arthritis/ Arthralgia	3 (23.1%)	1 (4.5%)	1 (5.9%)
Iritis/ Uveitis	0 (0.0%)	0 (0.0%)	0 (0.0%)
Erythema nodosum	0 (0.0%)	0 (0.0%)	0 (0.0%)
Pyoderma gangrenosum	0 (0.0%)	0 (0.0%)	0 (0.0%)

Abbreviations: ITT, Intent-to-Treat Analysis set; N, number of randomized subjects; n, number of subjects within each category.

Table 14.3.1.13: Summary of Extraintestinal Manifestations at Week 0, 8 ,16, 26, 36, 52

	Naive (N=13)	Biologic (N=22)	Multiple Biologic (N=17)
Aphthous	0 (0.0%)	0 (0.0%)	0 (0.0%)
Week 36			
Extraintestinal Manifestations	2 (15.4%)	0 (0.0%)	1 (5.9%)
Arthritis/ Arthralgia	2 (15.4%)	0 (0.0%)	1 (5.9%)
Iritis/ Uveitis	0 (0.0%)	0 (0.0%)	0 (0.0%)
Erythema nodosum	0 (0.0%)	0 (0.0%)	0 (0.0%)
Pyoderma gangrenosum	0 (0.0%)	0 (0.0%)	0 (0.0%)
Aphthous	0 (0.0%)	0 (0.0%)	0 (0.0%)
Week 52			
Extraintestinal Manifestations	1 (7.7%)	1 (4.5%)	2 (11.8%)
Arthritis/ Arthralgia	1 (7.7%)	1 (4.5%)	2 (11.8%)
Iritis/ Uveitis	0 (0.0%)	0 (0.0%)	0 (0.0%)
Erythema nodosum	0 (0.0%)	0 (0.0%)	0 (0.0%)
Pyoderma gangrenosum	0 (0.0%)	0 (0.0%)	0 (0.0%)
Aphthous	0 (0.0%)	0 (0.0%)	0 (0.0%)

Abbreviations: ITT, Intent-to-Treat Analysis set; N, number of randomized subjects; n, number of subjects within each category.

Program Name: MUCUS-Shell17-V01
Output Location: .\Output\Shared\MUCUS-TFLS-Final-27MAR24-V01.pdf

Table 14.3.1.14: Summary of Crohn's Related Hospitilizations

	Naive (N=13)	Biologic (N=22)	Multiple Biologic (N=17)
Hospitalization			
Total Number of subjects with Event	-	2	-
Total Number of Events	-	3	-
Event Rate (1st event)	-	2 (9.1%)	-
Mean time to 1st event (SD)	-	85.5 (40.31)	-
Median time to 1st event (Min, Max)	-	85.5 (57, 114)	-

Abbreviations: N, number of randomized subjects; n, number of subjects within each category.

Program Name: MUCUS-Shell9-V01
Output Location: .\Output\Shared\MUCUS-TFLS-Final-27MAR24-V01.pdf

Table 14.3.1.15: Summary of Crohn's Related Surgery

	Naive<br>(N=13)	Biologic<br>(N=22)	Multiple Biologic<br>(N=17)
Surgery			
Total Number of subjects with Event	1	2	-
Total Number of Events	1	3	-
Event Rate (1st event)	1 (7.7%)	2 (9.1%)	-
Mean time to 1st event (SD)	374.0 (-)	207.5 (212.84)	-
Median time to 1st event (Min, Max)	374.0 (374, 374)	207.5 (57, 358)	-

Abbreviations: N, number of randomized subjects; n, number of subjects within each category.

Program Name: MUCUS-Shell9-V01
Output Location: .\Output\Shared\MUCUS-TFLS-Final-27MAR24-V01.pdf

Table 14.3.5.1: Summary of Vital Signs

	Naive (N=13)	Biologic (N=22)	Multiple Biologic (N=17)
Body Mass Index (kg/M^2)			
Baseline: OBS			
N	8	15	10
Missing	5	7	7
Mean (SD)	23.9 (4.21)	24.5 (3.57)	24.3 (4.50)
Median (Min, Max)	23.4 (18, 30)	23.7 (18, 32)	24.3 (17, 31)
Week 8: OBS			
N	11	18	14
Missing	2	4	3
Mean (SD)	23.7 (3.87)	24.6 (3.87)	24.9 (4.22)
Median (Min, Max)	23.2 (19, 29)	23.2 (20, 33)	23.8 (18, 32)
Week 8: CHG			
N	8	13	10
Missing	5	9	7
Mean (SD)	0.3 (0.66)	0.2 (0.75)	0.4 (1.24)
Median (Min, Max)	0.1 (-0, 1)	0.0 (-1, 2)	0.8 (-3, 2)
Week 8: PCHG			
N	8	13	10
Missing	5	9	7
Mean (SD)	1.7 (2.76)	1.1 (3.59)	2.1 (5.15)
Median (Min, Max)	0.6 (-1, 5)	0.0 (-4, 10)	3.6 (-10, 8)
Week 16: OBS			
N	8	17	13
Missing	5	5	4
Mean (SD)	24.0 (3.88)	24.9 (3.60)	24.5 (4.12)
Median (Min, Max)	23.8 (18, 29)	24.7 (20, 32)	24.2 (19, 32)

Abbreviations: N, number of randomized subjects; n, number of subjects within each category; SD, Standard Deviation; Min, Minimum; Max, Maximum.

Program Name: MUCUS-Shell11-V01
Output Location: .\Output\Shared\MUCUS-TFLS-Final-27MAR24-V01.pdf

Table 14.3.5.1: Summary of Vital Signs

	Naive (N=13)	Biologic (N=22)	Multiple Biologic (N=17)
Week 16: CHG			
N	7	13	9
Missing	6	9	8
Mean (SD)	0.1 (0.57)	0.6 (1.39)	0.3 (1.78)
Median (Min, Max)	0.3 (-1, 1)	0.0 (-2, 4)	1.0 (-3, 2)
Week 16: PCHG			
N	7	13	9
Missing	6	9	8
Mean (SD)	0.7 (2.41)	2.8 (5.82)	2.0 (7.78)
Median (Min, Max)	1.2 (-2, 4)	0.0 (-5, 15)	4.7 (-13, 11)
Week 26: OBS			
N	7	11	6
Missing	6	11	11
Mean (SD)	24.3 (3.66)	25.8 (3.80)	24.8 (2.94)
Median (Min, Max)	22.1 (21, 30)	24.7 (22, 32)	24.3 (22, 30)
Week 26: CHG			
N	6	8	5
Missing	7	14	12
Mean (SD)	0.4 (0.83)	0.7 (1.12)	1.3 (1.26)
Median (Min, Max)	0.6 (-1, 1)	0.3 (-0, 3)	0.7 (0, 3)
Week 26: PCHG			
N	6	8	5
Missing	7	14	12
Mean (SD)	1.9 (3.67)	3.1 (4.52)	6.4 (6.26)
Median (Min, Max)	2.5 (-4, 6)	1.2 (-1, 12)	3.1 (0, 13)

Abbreviations: N, number of randomized subjects; n, number of subjects within each category; SD, Standard Deviation; Min, Minimum; Max, Maximum.

Program Name: MUCUS-Shell11-V01
Output Location: .\Output\Shared\MUCUS-TFLS-Final-27MAR24-V01.pdf

Table 14.3.5.1: Summary of Vital Signs

	Naive (N=13)	Biologic (N=22)	Multiple Biologic (N=17)
Week 36: OBS			
N	7	9	9
Missing	6	13	8
Mean (SD)	24.5 (3.93)	26.0 (3.41)	25.2 (3.88)
Median (Min, Max)	23.0 (21, 32)	24.7 (21, 32)	24.9 (20, 32)
Week 36: CHG			
N	7	8	7
Missing	6	14	10
Mean (SD)	1.1 (1.24)	0.8 (1.49)	1.3 (1.65)
Median (Min, Max)	1.2 (-1, 3)	0.1 (-0, 4)	1.4 (-2, 3)
Week 36: PCHG			
N	7	8	7
Missing	6	14	10
Mean (SD)	5.1 (5.92)	3.4 (6.11)	5.6 (7.16)
Median (Min, Max)	5.9 (-4, 15)	0.5 (-1, 17)	5.6 (-8, 16)
Week 52: OBS			
N	8	11	10
Missing	5	11	7
Mean (SD)	24.1 (3.70)	24.2 (2.95)	25.3 (4.15)
Median (Min, Max)	22.0 (20, 29)	23.4 (20, 29)	25.0 (20, 34)
Week 52: CHG			
N	6	9	9
Missing	7	13	8
Mean (SD)	0.5 (1.33)	0.6 (1.91)	1.6 (2.07)
Median (Min, Max)	0.4 (-1, 2)	0.4 (-3, 4)	0.7 (-1, 5)

Abbreviations: N, number of randomized subjects; n, number of subjects within each category; SD, Standard Deviation; Min, Minimum; Max, Maximum.

Program Name: MUCUS-Shell11-V01
Output Location: .\Output\Shared\MUCUS-TFLS-Final-27MAR24-V01.pdf

Table 14.3.5.1: Summary of Vital Signs

	Naive (N=13)	Biologic (N=22)	Multiple Biologic (N=17)
Week 52: PCHG			
N	6	9	9
Missing	7	13	8
Mean (SD)	3.1 (6.66)	2.5 (7.84)	7.8 (11.01)
Median (Min, Max)	1.9 (-5, 13)	1.8 (-13, 17)	3.1 (-2, 32)
Early Termination: OBS			
N	0	2	2
Missing	-	-	-
Mean (SD)	-	24.7 (2.61)	24.9 (6.86)
Median (Min, Max)	-	24.7 (23, 27)	24.9 (20, 30)
Early Termination: CHG			
N	0	2	0
Missing	-	-	-
Mean (SD)	-	0.0 (0.00)	-
Median (Min, Max)	-	0.0 (0, 0)	-
Early Termination: PCHG			
N	0	2	0
Missing	-	-	-
Mean (SD)	-	0.0 (0.00)	-
Median (Min, Max)	-	0.0 (0, 0)	-
Disease Flare: OBS			
N	0	0	0
Missing	-	-	-
Mean (SD)	-	-	-
Median (Min, Max)	-	-	-

Abbreviations: N, number of randomized subjects; n, number of subjects within each category; SD, Standard Deviation; Min, Minimum; Max, Maximum.

Program Name: MUCUS-Shell11-V01
Output Location: .\Output\Shared\MUCUS-TFLS-Final-27MAR24-V01.pdf

Table 14.3.5.1: Summary of Vital Signs

	Naive (N=13)	Biologic (N=22)	Multiple Biologic (N=17)
Disease Flare: CHG			
N	0	0	0
Missing	-	-	-
Mean (SD)	-	-	-
Median (Min, Max)	-	-	-
Disease Flare: PCHG			
N	0	0	0
Missing	-	-	-
Mean (SD)	-	-	-
Median (Min, Max)	-	-	-
Safety Follow-Up: OBS			
N	6	8	9
Missing	7	14	8
Mean (SD)	23.2 (2.95)	23.4 (2.77)	27.8 (3.37)
Median (Min, Max)	21.9 (20, 27)	22.7 (21, 29)	27.3 (24, 35)
Safety Follow-Up: CHG			
N	5	6	6
Missing	8	16	11
Mean (SD)	0.5 (1.41)	-0.2 (1.55)	2.5 (2.71)
Median (Min, Max)	0.8 (-1, 2)	0.2 (-3, 1)	1.5 (0, 8)
Safety Follow-Up: PCHG			
N	5	6	6
Missing	8	16	11
Mean (SD)	3.3 (6.78)	-0.8 (6.18)	11.8 (16.54)
Median (Min, Max)	3.8 (-5, 13)	0.7 (-13, 5)	6.1 (0, 45)

Abbreviations: N, number of randomized subjects; n, number of subjects within each category; SD, Standard Deviation; Min, Minimum; Max, Maximum.

Program Name: MUCUS-Shell11-V01
Output Location: .\Output\Shared\MUCUS-TFLS-Final-27MAR24-V01.pdf

Table 14.3.5.1: Summary of Vital Signs

	Naive (N=13)	Biologic (N=22)	Multiple Biologic (N=17)
Diastolic Blood Pressure (mmHg)			
Baseline: OBS			
N	9	17	11
Missing	4	5	6
Mean (SD)	73.6 (12.37)	83.6 (14.09)	77.9 (7.62)
Median (Min, Max)	70.0 (58, 94)	83.0 (60, 114)	76.0 (68, 92)
Week 8: OBS			
N	12	19	13
Missing	1	3	4
Mean (SD)	81.1 (11.31)	80.7 (8.60)	78.2 (8.08)
Median (Min, Max)	79.0 (67, 105)	83.0 (69, 99)	78.0 (67, 91)
Week 8: CHG			
N	9	15	9
Missing	4	7	8
Mean (SD)	5.6 (6.88)	0.4 (10.21)	2.6 (9.06)
Median (Min, Max)	9.0 (-5, 16)	1.0 (-14, 22)	0.0 (-10, 19)
Week 8: PCHG			
N	9	15	9
Missing	4	7	8
Mean (SD)	8.6 (10.41)	2.0 (13.87)	3.7 (12.31)
Median (Min, Max)	13.3 (-6, 26)	1.1 (-14, 35)	0.0 (-12, 28)
Week 16: OBS			
N	9	18	13
Missing	4	4	4
Mean (SD)	81.8 (10.66)	81.3 (6.89)	78.9 (8.78)

Abbreviations: N, number of randomized subjects; n, number of subjects within each category; SD, Standard Deviation; Min, Minimum; Max, Maximum.

Table 14.3.5.1: Summary of Vital Signs

	Naive (N=13)	Biologic (N=22)	Multiple Biologic (N=17)
Median (Min, Max)	80.0 (70, 105)	81.0 (70, 95)	79.0 (63, 93)
Week 16: CHG			
N	8	14	9
Missing	5	8	8
Mean (SD)	6.1 (6.66)	-1.2 (10.67)	1.3 (7.94)
Median (Min, Max)	5.0 (0, 20)	-3.0 (-16, 15)	0.0 (-11, 16)
Week 16: PCHG			
N	8	14	9
Missing	5	8	8
Mean (SD)	8.7 (10.38)	0.2 (13.42)	2.0 (10.09)
Median (Min, Max)	7.2 (0, 32)	-3.6 (-16, 20)	0.0 (-13, 21)
Week 26: OBS			
N	7	13	6
Missing	6	9	11
Mean (SD)	74.9 (8.69)	84.8 (15.25)	74.0 (2.97)
Median (Min, Max)	70.0 (67, 90)	80.0 (72, 130)	75.0 (70, 78)
Week 26: CHG			
N	6	10	5
Missing	7	12	12
Mean (SD)	1.5 (2.74)	2.4 (17.48)	-4.2 (5.72)
Median (Min, Max)	0.0 (-1, 5)	0.5 (-21, 35)	-5.0 (-11, 3)
Week 26: PCHG			
N	6	10	5
Missing	7	12	12
Mean (SD)	2.1 (3.95)	4.4 (19.92)	-4.9 (6.89)

Abbreviations: N, number of randomized subjects; n, number of subjects within each category; SD, Standard Deviation; Min, Minimum; Max, Maximum.

Table 14.3.5.1: Summary of Vital Signs

	Naive (N=13)	Biologic (N=22)	Multiple Biologic (N=17)
Median (Min, Max)	0.0 (-1, 8)	1.9 (-22, 37)	-6.3 (-13, 4)
Week 36: OBS			
N	7	9	8
Missing	6	13	9
Mean (SD)	69.1 (7.95)	83.4 (6.13)	78.8 (6.94)
Median (Min, Max)	69.0 (57, 80)	84.0 (73, 93)	80.0 (70, 90)
Week 36: CHG			
N	7	8	6
Missing	6	14	11
Mean (SD)	-3.1 (7.99)	3.0 (14.54)	1.0 (8.29)
Median (Min, Max)	-1.0 (-15, 10)	6.0 (-28, 18)	0.0 (-8, 15)
Week 36: PCHG			
N	7	8	6
Missing	6	14	11
Mean (SD)	-3.6 (10.42)	6.2 (17.72)	1.7 (10.67)
Median (Min, Max)	-1.7 (-19, 15)	7.4 (-28, 28)	0.0 (-10, 20)
Week 52: OBS			
N	9	13	10
Missing	4	9	7
Mean (SD)	74.3 (11.97)	85.6 (22.92)	79.7 (10.93)
Median (Min, Max)	71.0 (60, 100)	81.0 (69, 158)	80.0 (61, 96)
Week 52: CHG			
N	7	11	9
Missing	6	11	8
Mean (SD)	3.9 (9.55)	5.5 (23.79)	1.3 (11.94)

Abbreviations: N, number of randomized subjects; n, number of subjects within each category; SD, Standard Deviation; Min, Minimum; Max, Maximum.

Program Name: MUCUS-Shell11-V01
Output Location: .\Output\Shared\MUCUS-TFLS-Final-27MAR24-V01.pdf

Table 14.3.5.1: Summary of Vital Signs

	Naive (N=13)	Biologic (N=22)	Multiple Biologic (N=17)
Median (Min, Max)	2.0 (-10, 21)	3.0 (-26, 63)	2.0 (-19, 20)
Week 52: PCHG			
N	7	11	9
Missing	6	11	8
Mean (SD)	6.5 (14.44)	8.1 (26.04)	2.3 (15.57)
Median (Min, Max)	3.1 (-13, 34)	3.8 (-26, 66)	2.2 (-24, 26)
Early Termination: OBS			
N	0	2	2
Missing	-	-	-
Mean (SD)	-	84.5 (3.54)	85.0 (0.00)
Median (Min, Max)	-	84.5 (82, 87)	85.0 (85, 85)
Early Termination: CHG			
N	0	2	0
Missing	-	-	-
Mean (SD)	-	1.0 (8.49)	-
Median (Min, Max)	-	1.0 (-5, 7)	-
Early Termination: PCHG			
N	0	2	0
Missing	-	-	-
Mean (SD)	-	1.5 (10.25)	-
Median (Min, Max)	-	1.5 (-6, 9)	-
Disease Flare: OBS			
N	0	0	0
Missing	-	-	-
Mean (SD)	-	-	-

Abbreviations: N, number of randomized subjects; n, number of subjects within each category; SD, Standard Deviation; Min, Minimum; Max, Maximum.

Program Name: MUCUS-Shell11-V01
Output Location: .\Output\Shared\MUCUS-TFLS-Final-27MAR24-V01.pdf

Table 14.3.5.1: Summary of Vital Signs

	Naive (N=13)	Biologic (N=22)	Multiple Biologic (N=17)
Median (Min, Max)	-	-	-
Disease Flare: CHG			
N	0	0	0
Missing	-	-	-
Mean (SD)	-	-	-
Median (Min, Max)	-	-	-
Disease Flare: PCHG			
N	0	0	0
Missing	-	-	-
Mean (SD)	-	-	-
Median (Min, Max)	-	-	-
Safety Follow-Up: OBS			
N	7	8	9
Missing	6	14	8
Mean (SD)	77.0 (16.83)	82.5 (10.81)	80.4 (8.65)
Median (Min, Max)	75.0 (54, 107)	84.0 (66, 96)	80.0 (67, 91)
Safety Follow-Up: CHG			
N	6	6	6
Missing	7	16	11
Mean (SD)	6.0 (8.76)	2.2 (11.89)	1.2 (13.26)
Median (Min, Max)	5.0 (-4, 19)	2.0 (-18, 15)	-6.0 (-10, 21)
Safety Follow-Up: PCHG			
N	6	6	6
Missing	7	16	11
Mean (SD)	8.2 (13.33)	4.0 (15.47)	2.6 (17.88)

Abbreviations: N, number of randomized subjects; n, number of subjects within each category; SD, Standard Deviation; Min, Minimum; Max, Maximum.

Table 14.3.5.1: Summary of Vital Signs

	Naive (N=13)	Biologic (N=22)	Multiple Biologic (N=17)
Median (Min, Max)	7.4 (-7, 31)	2.2 (-20, 25)	-6.9 (-13, 30)
Height (cm)			
Baseline: OBS			
N	12	20	16
Missing	1	2	1
Mean (SD)	173.3 (14.89)	171.3 (10.61)	173.5 (7.80)
Median (Min, Max)	169.0 (147, 195)	169.0 (157, 192)	172.0 (161, 195)
Pulse Rate (beats/min)			
Baseline: OBS			
N	9	17	11
Missing	4	5	6
Mean (SD)	79.4 (18.04)	78.4 (9.38)	80.4 (9.46)
Median (Min, Max)	75.0 (62, 111)	78.0 (62, 97)	80.0 (67, 96)
Week 8: OBS			
N	12	19	13
Missing	1	3	4
Mean (SD)	79.6 (11.67)	79.2 (15.48)	78.5 (9.58)
Median (Min, Max)	80.0 (62, 98)	77.0 (56, 120)	77.0 (68, 107)
Week 8: CHG			
N	9	15	9
Missing	4	7	8
Mean (SD)	-2.9 (17.40)	4.9 (16.94)	-0.8 (12.70)
Median (Min, Max)	4.0 (-30, 16)	0.0 (-16, 37)	0.0 (-24, 23)
Week 8: PCHG			
N	9	15	9

Abbreviations: N, number of randomized subjects; n, number of subjects within each category; SD, Standard Deviation; Min, Minimum; Max, Maximum.

Program Name: MUCUS-Shell11-V01
Output Location: .\Output\Shared\MUCUS-TFLS-Final-27MAR24-V01.pdf

Table 14.3.5.1: Summary of Vital Signs

	Naive (N=13)	Biologic (N=22)	Multiple Biologic (N=17)
Missing	4	7	8
Mean (SD)	-0.3 (19.94)	7.3 (22.52)	-0.1 (14.30)
Median (Min, Max)	6.3 (-27, 25)	0.0 (-19, 50)	0.0 (-25, 27)
Week 16: OBS			
N	9	18	13
Missing	4	4	4
Mean (SD)	71.7 (12.56)	78.8 (10.43)	75.9 (6.42)
Median (Min, Max)	68.0 (61, 102)	79.0 (55, 101)	76.0 (67, 86)
Week 16: CHG			
N	8	14	9
Missing	5	8	8
Mean (SD)	-4.5 (21.79)	-1.4 (11.63)	-2.7 (11.26)
Median (Min, Max)	-4.5 (-44, 27)	-3.0 (-17, 24)	0.0 (-28, 11)
Week 16: PCHG			
N	8	14	9
Missing	5	8	8
Mean (SD)	-2.7 (25.06)	-1.1 (14.94)	-2.1 (12.77)
Median (Min, Max)	-6.4 (-40, 36)	-3.9 (-18, 31)	0.0 (-29, 16)
Week 26: OBS			
N	7	13	6
Missing	6	9	11
Mean (SD)	68.1 (10.62)	76.2 (7.88)	75.8 (3.25)
Median (Min, Max)	68.0 (55, 86)	76.0 (63, 91)	76.5 (72, 80)
Week 26: CHG			
N	6	10	5

Abbreviations: N, number of randomized subjects; n, number of subjects within each category; SD, Standard Deviation; Min, Minimum; Max, Maximum.

Program Name: MUCUS-Shell11-V01
Output Location: .\Output\Shared\MUCUS-TFLS-Final-27MAR24-V01.pdf

Table 14.3.5.1: Summary of Vital Signs

	Naive (N=13)	Biologic (N=22)	Multiple Biologic (N=17)
Missing	7	12	12
Mean (SD)	-1.0 (14.00)	-2.5 (9.10)	-5.6 (8.17)
Median (Min, Max)	2.0 (-22, 14)	0.5 (-19, 8)	-2.0 (-16, 4)
Week 26: PCHG			
N	6	10	5
Missing	7	12	12
Mean (SD)	0.1 (18.79)	-2.7 (10.85)	-6.1 (9.22)
Median (Min, Max)	3.1 (-28, 23)	0.6 (-20, 12)	-2.5 (-17, 6)
Week 36: OBS			
N	7	9	8
Missing	6	13	9
Mean (SD)	67.4 (11.37)	77.9 (8.57)	79.1 (7.85)
Median (Min, Max)	66.0 (54, 88)	80.0 (65, 89)	78.5 (68, 96)
Week 36: CHG			
N	7	8	6
Missing	6	14	11
Mean (SD)	-8.9 (16.39)	4.5 (9.50)	1.7 (14.56)
Median (Min, Max)	-9.0 (-37, 13)	3.5 (-12, 21)	-1.0 (-16, 28)
Week 36: PCHG			
N	7	8	6
Missing	6	14	11
Mean (SD)	-9.5 (18.75)	6.9 (14.27)	3.8 (19.73)
Median (Min, Max)	-12.0 (-35, 21)	4.6 (-16, 34)	-1.3 (-17, 41)
Week 52: OBS			
N	9	13	10

Abbreviations: N, number of randomized subjects; n, number of subjects within each category; SD, Standard Deviation; Min, Minimum; Max, Maximum.

Table 14.3.5.1: Summary of Vital Signs

	Naive (N=13)	Biologic (N=22)	Multiple Biologic (N=17)
Missing	4	9	7
Mean (SD)	68.7 (10.44)	74.8 (13.38)	77.2 (10.52)
Median (Min, Max)	67.0 (56, 88)	74.0 (45, 101)	76.0 (63, 94)
Week 52: CHG			
N	7	11	9
Missing	6	11	8
Mean (SD)	-7.1 (15.76)	-1.5 (11.52)	-2.3 (16.69)
Median (Min, Max)	-3.0 (-39, 12)	-3.0 (-23, 21)	0.0 (-24, 27)
Week 52: PCHG			
N	7	11	9
Missing	6	11	8
Mean (SD)	-7.1 (16.84)	-1.4 (13.61)	-1.0 (21.40)
Median (Min, Max)	-4.8 (-37, 19)	-3.5 (-24, 26)	0.0 (-25, 40)
Early Termination: OBS			
N	0	2	2
Missing	-	-	-
Mean (SD)	-	80.0 (14.14)	84.5 (12.02)
Median (Min, Max)	-	80.0 (70, 90)	84.5 (76, 93)
Early Termination: CHG			
N	0	2	0
Missing	-	-	-
Mean (SD)	-	-3.0 (4.24)	-
Median (Min, Max)	-	-3.0 (-6, 0)	-
Early Termination: PCHG			
N	0	2	0

Abbreviations: N, number of randomized subjects; n, number of subjects within each category; SD, Standard Deviation; Min, Minimum; Max, Maximum.

Table 14.3.5.1: Summary of Vital Signs

	Naive (N=13)	Biologic (N=22)	Multiple Biologic (N=17)
Missing	-	-	-
Mean (SD)	-	-3.9 (5.58)	-
Median (Min, Max)	-	-3.9 (-8, 0)	-
Disease Flare: OBS			
N	0	0	0
Missing	-	-	-
Mean (SD)	-	-	-
Median (Min, Max)	-	-	-
Disease Flare: CHG			
N	0	0	0
Missing	-	-	-
Mean (SD)	-	-	-
Median (Min, Max)	-	-	-
Disease Flare: PCHG			
N	0	0	0
Missing	-	-	-
Mean (SD)	-	-	-
Median (Min, Max)	-	-	-
Safety Follow-Up: OBS			
N	7	8	9
Missing	6	14	8
Mean (SD)	70.4 (6.90)	76.3 (3.92)	76.0 (7.40)
Median (Min, Max)	73.0 (60, 78)	76.5 (71, 83)	76.0 (66, 90)
Safety Follow-Up: CHG			
N	6	6	6

Abbreviations: N, number of randomized subjects; n, number of subjects within each category; SD, Standard Deviation; Min, Minimum; Max, Maximum.

Table 14.3.5.1: Summary of Vital Signs

	Naive (N=13)	Biologic (N=22)	Multiple Biologic (N=17)
Missing	7	16	11
Mean (SD)	-14.8 (22.85)	-1.7 (8.45)	-7.7 (12.44)
Median (Min, Max)	-5.0 (-46, 5)	-1.0 (-12, 12)	-12.0 (-20, 15)
Safety Follow-Up: PCHG			
N	6	6	6
Missing	7	16	11
Mean (SD)	-13.5 (21.72)	-1.2 (11.87)	-7.8 (15.80)
Median (Min, Max)	-6.0 (-43, 7)	-1.3 (-14, 19)	-13.8 (-21, 22)
Systolic Blood Pressure (mmHg)			
Baseline: OBS			
N	9	17	11
Missing	4	5	6
Mean (SD)	114.9 (20.15)	126.7 (17.64)	124.2 (20.18)
Median (Min, Max)	120.0 (76, 147)	121.0 (105, 168)	120.0 (106, 182)
Week 8: OBS			
N	12	19	13
Missing	1	3	4
Mean (SD)	129.1 (17.52)	122.5 (14.34)	121.8 (8.24)
Median (Min, Max)	129.0 (100, 156)	125.0 (99, 155)	122.0 (105, 136)
Week 8: CHG			
N	9	15	9
Missing	4	7	8
Mean (SD)	11.6 (10.26)	1.3 (7.64)	3.0 (11.12)
Median (Min, Max)	9.0 (-2, 27)	0.0 (-11, 22)	0.0 (-5, 30)
Week 8: PCHG			

Abbreviations: N, number of randomized subjects; n, number of subjects within each category; SD, Standard Deviation; Min, Minimum; Max, Maximum.

Table 14.3.5.1: Summary of Vital Signs

	Naive (N=13)	Biologic (N=22)	Multiple Biologic (N=17)
N	9	15	9
Missing	4	7	8
Mean (SD)	11.5 (11.66)	1.3 (6.74)	2.8 (10.27)
Median (Min, Max)	7.4 (-2, 32)	0.0 (-10, 20)	0.0 (-5, 28)
Week 16: OBS			
N	9	18	13
Missing	4	4	4
Mean (SD)	125.7 (17.39)	127.2 (12.95)	123.6 (11.40)
Median (Min, Max)	121.0 (112, 166)	126.0 (112, 162)	123.0 (103, 146)
Week 16: CHG			
N	8	14	9
Missing	5	8	8
Mean (SD)	6.5 (13.75)	1.2 (11.25)	-2.6 (21.01)
Median (Min, Max)	0.0 (-9, 28)	3.0 (-23, 17)	0.0 (-54, 20)
Week 16: PCHG			
N	8	14	9
Missing	5	8	8
Mean (SD)	5.9 (12.83)	1.7 (8.56)	-0.4 (12.99)
Median (Min, Max)	0.0 (-7, 30)	2.6 (-15, 16)	0.0 (-30, 16)
Week 26: OBS			
N	7	13	6
Missing	6	9	11
Mean (SD)	122.0 (9.93)	129.4 (21.76)	114.5 (6.25)
Median (Min, Max)	120.0 (110, 140)	125.0 (110, 192)	115.0 (105, 121)
Week 26: CHG			

Abbreviations: N, number of randomized subjects; n, number of subjects within each category; SD, Standard Deviation; Min, Minimum; Max, Maximum.

Program Name: MUCUS-Shell11-V01
Output Location: .\Output\Shared\MUCUS-TFLS-Final-27MAR24-V01.pdf

Table 14.3.5.1: Summary of Vital Signs

	Naive (N=13)	Biologic (N=22)	Multiple Biologic (N=17)
N	6	10	5
Missing	7	12	12
Mean (SD)	1.0 (10.58)	3.4 (16.56)	-8.0 (5.10)
Median (Min, Max)	-1.0 (-11, 20)	2.5 (-27, 39)	-7.0 (-15, -2)
Week 26: PCHG			
N	6	10	5
Missing	7	12	12
Mean (SD)	0.9 (8.80)	2.7 (11.36)	-6.6 (4.18)
Median (Min, Max)	-0.8 (-9, 17)	2.1 (-18, 25)	-5.7 (-13, -2)
Week 36: OBS			
N	7	9	8
Missing	6	13	9
Mean (SD)	116.3 (15.29)	127.8 (14.56)	122.1 (10.58)
Median (Min, Max)	120.0 (87, 135)	129.0 (105, 159)	122.5 (105, 135)
Week 36: CHG			
N	7	8	6
Missing	6	14	11
Mean (SD)	2.9 (7.45)	3.9 (13.02)	0.7 (14.77)
Median (Min, Max)	0.0 (-6, 15)	7.0 (-19, 19)	-2.5 (-16, 25)
Week 36: PCHG			
N	7	8	6
Missing	6	14	11
Mean (SD)	3.1 (7.40)	3.6 (10.17)	0.9 (12.86)
Median (Min, Max)	0.0 (-5, 14)	5.7 (-13, 14)	-2.0 (-13, 23)
Week 52: OBS			

Abbreviations: N, number of randomized subjects; n, number of subjects within each category; SD, Standard Deviation; Min, Minimum; Max, Maximum.

Program Name: MUCUS-Shell11-V01
Output Location: .\Output\Shared\MUCUS-TFLS-Final-27MAR24-V01.pdf

Table 14.3.5.1: Summary of Vital Signs

	Naive (N=13)	Biologic (N=22)	Multiple Biologic (N=17)
N	9	13	10
Missing	4	9	7
Mean (SD)	124.4 (16.43)	130.3 (16.80)	127.5 (15.25)
Median (Min, Max)	126.0 (105, 159)	125.0 (112, 175)	127.0 (100, 148)
Week 52: CHG			
N	7	11	9
Missing	6	11	8
Mean (SD)	8.9 (18.59)	8.2 (14.26)	1.1 (19.80)
Median (Min, Max)	7.0 (-16, 34)	9.0 (-24, 31)	1.0 (-34, 28)
Week 52: PCHG			
N	7	11	9
Missing	6	11	8
Mean (SD)	10.8 (21.20)	7.1 (11.35)	2.2 (15.12)
Median (Min, Max)	5.6 (-13, 45)	7.0 (-16, 30)	0.8 (-19, 26)
Early Termination: OBS			
N	0	2	2
Missing	-	-	-
Mean (SD)	-	135.5 (12.02)	129.0 (4.24)
Median (Min, Max)	-	135.5 (127, 144)	129.0 (126, 132)
Early Termination: CHG			
N	0	2	0
Missing	-	-	-
Mean (SD)	-	5.5 (2.12)	-
Median (Min, Max)	-	5.5 (4, 7)	-
Early Termination: PCHG			

Abbreviations: N, number of randomized subjects; n, number of subjects within each category; SD, Standard Deviation; Min, Minimum; Max, Maximum.

Program Name: MUCUS-Shell11-V01
Output Location: .\Output\Shared\MUCUS-TFLS-Final-27MAR24-V01.pdf

Table 14.3.5.1: Summary of Vital Signs

	Naive (N=13)	Biologic (N=22)	Multiple Biologic (N=17)
N	0	2	0
Missing	-	-	-
Mean (SD)	-	4.3 (2.10)	-
Median (Min, Max)	-	4.3 (3, 6)	-
Disease Flare: OBS			
N	0	0	0
Missing	-	-	-
Mean (SD)	-	-	-
Median (Min, Max)	-	-	-
Disease Flare: CHG			
N	0	0	0
Missing	-	-	-
Mean (SD)	-	-	-
Median (Min, Max)	-	-	-
Disease Flare: PCHG			
N	0	0	0
Missing	-	-	-
Mean (SD)	-	-	-
Median (Min, Max)	-	-	-
Safety Follow-Up: OBS			
N	7	8	9
Missing	6	14	8
Mean (SD)	120.0 (20.02)	130.0 (23.29)	129.4 (9.36)
Median (Min, Max)	110.0 (103, 159)	128.5 (93, 172)	128.0 (120, 149)
Safety Follow-Up: CHG			

Abbreviations: N, number of randomized subjects; n, number of subjects within each category; SD, Standard Deviation; Min, Minimum; Max, Maximum.

Table 14.3.5.1: Summary of Vital Signs

	Naive (N=13)	Biologic (N=22)	Multiple Biologic (N=17)
N	6	6	6
Missing	7	16	11
Mean (SD)	6.2 (17.47)	11.2 (15.79)	1.3 (28.30)
Median (Min, Max)	3.0 (-11, 27)	7.0 (-3, 42)	5.0 (-49, 30)
Safety Follow-Up: PCHG			
N	6	6	6
Missing	7	16	11
Mean (SD)	8.0 (19.24)	8.9 (12.09)	3.9 (19.47)
Median (Min, Max)	1.4 (-9, 36)	5.7 (-2, 32)	4.2 (-27, 28)
Temperature (C)			
Baseline: OBS			
N	9	17	11
Missing	4	5	6
Mean (SD)	36.4 (0.42)	36.4 (0.37)	36.3 (0.57)
Median (Min, Max)	36.3 (36, 37)	36.5 (36, 37)	36.4 (35, 37)
Week 8: OBS			
N	12	20	14
Missing	1	2	3
Mean (SD)	36.3 (0.34)	36.5 (0.51)	36.3 (0.42)
Median (Min, Max)	36.3 (36, 37)	36.5 (36, 38)	36.3 (36, 37)
Week 8: CHG			
N	9	15	10
Missing	4	7	7
Mean (SD)	-0.0 (0.22)	0.0 (0.56)	0.1 (0.44)
Median (Min, Max)	0.0 (-0, 0)	0.1 (-1, 1)	-0.0 (-0, 1)

Abbreviations: N, number of randomized subjects; n, number of subjects within each category; SD, Standard Deviation; Min, Minimum; Max, Maximum.

Program Name: MUCUS-Shell11-V01
Output Location: .\Output\Shared\MUCUS-TFLS-Final-27MAR24-V01.pdf

Table 14.3.5.1: Summary of Vital Signs

	Naive (N=13)	Biologic (N=22)	Multiple Biologic (N=17)
Week 8: PCHG			
N	9	15	10
Missing	4	7	7
Mean (SD)	-0.1 (0.60)	0.1 (1.52)	0.2 (1.25)
Median (Min, Max)	0.0 (-1, 1)	0.3 (-3, 3)	-0.1 (-1, 3)
Week 16: OBS			
N	9	18	13
Missing	4	4	4
Mean (SD)	36.3 (0.36)	36.5 (0.44)	36.3 (0.34)
Median (Min, Max)	36.2 (36, 37)	36.6 (36, 38)	36.3 (36, 37)
Week 16: CHG			
N	8	14	9
Missing	5	8	8
Mean (SD)	-0.0 (0.27)	0.1 (0.57)	0.1 (0.68)
Median (Min, Max)	-0.0 (-1, 0)	-0.1 (-1, 1)	-0.1 (-0, 2)
Week 16: PCHG			
N	8	14	9
Missing	5	8	8
Mean (SD)	-0.1 (0.73)	0.2 (1.58)	0.2 (1.93)
Median (Min, Max)	-0.0 (-1, 1)	-0.1 (-2, 4)	-0.3 (-1, 5)
Week 26: OBS			
N	7	12	6
Missing	6	10	11
Mean (SD)	36.3 (0.32)	40.3 (12.53)	36.4 (0.34)
Median (Min, Max)	36.1 (36, 37)	36.8 (36, 80)	36.3 (36, 37)

Abbreviations: N, number of randomized subjects; n, number of subjects within each category; SD, Standard Deviation; Min, Minimum; Max, Maximum.

Program Name: MUCUS-Shell11-V01
Output Location: .\Output\Shared\MUCUS-TFLS-Final-27MAR24-V01.pdf

Table 14.3.5.1: Summary of Vital Signs

	Naive (N=13)	Biologic (N=22)	Multiple Biologic (N=17)
Week 26: CHG			
N	6	9	5
Missing	7	13	12
Mean (SD)	-0.0 (0.26)	5.1 (14.33)	0.1 (0.35)
Median (Min, Max)	-0.1 (-0, 0)	0.3 (-0, 43)	-0.1 (-0, 1)
Week 26: PCHG			
N	6	9	5
Missing	7	13	12
Mean (SD)	-0.0 (0.73)	13.9 (39.05)	0.2 (0.96)
Median (Min, Max)	-0.1 (-1, 1)	0.8 (-1, 118)	-0.3 (-0, 2)
Week 36: OBS			
N	7	10	9
Missing	6	12	8
Mean (SD)	36.2 (0.35)	36.3 (0.58)	36.3 (0.26)
Median (Min, Max)	36.2 (36, 37)	36.3 (36, 38)	36.4 (36, 37)
Week 36: CHG			
N	7	9	7
Missing	6	13	10
Mean (SD)	-0.0 (0.39)	-0.0 (0.54)	0.2 (0.48)
Median (Min, Max)	-0.1 (-1, 1)	-0.1 (-1, 1)	0.2 (-0, 1)
Week 36: PCHG			
N	7	9	7
Missing	6	13	10
Mean (SD)	-0.1 (1.08)	-0.1 (1.47)	0.6 (1.35)
Median (Min, Max)	-0.3 (-2, 1)	-0.3 (-2, 3)	0.6 (-1, 3)

Abbreviations: N, number of randomized subjects; n, number of subjects within each category; SD, Standard Deviation; Min, Minimum; Max, Maximum.

Program Name: MUCUS-Shell11-V01
Output Location: .\Output\Shared\MUCUS-TFLS-Final-27MAR24-V01.pdf

Table 14.3.5.1: Summary of Vital Signs

	Naive (N=13)	Biologic (N=22)	Multiple Biologic (N=17)
Week 52: OBS			
N	9	12	10
Missing	4	10	7
Mean (SD)	36.4 (0.37)	36.4 (0.28)	36.3 (0.34)
Median (Min, Max)	36.4 (36, 37)	36.5 (36, 37)	36.3 (36, 37)
Week 52: CHG			
N	7	10	9
Missing	6	12	8
Mean (SD)	-0.1 (0.42)	-0.1 (0.25)	-0.1 (0.78)
Median (Min, Max)	0.1 (-1, 0)	-0.2 (-0, 0)	-0.2 (-1, 1)
Week 52: PCHG			
N	7	10	9
Missing	6	12	8
Mean (SD)	-0.2 (1.12)	-0.2 (0.69)	-0.2 (2.16)
Median (Min, Max)	0.3 (-2, 1)	-0.4 (-1, 1)	-0.6 (-3, 4)
Early Termination: OBS			
N	0	2	2
Missing	-	-	-
Mean (SD)	-	36.4 (0.49)	36.5 (0.07)
Median (Min, Max)	-	36.4 (36, 37)	36.5 (36, 37)
Early Termination: CHG			
N	0	2	0
Missing	-	-	-
Mean (SD)	-	0.1 (0.42)	-
Median (Min, Max)	-	0.1 (-0, 0)	-

Abbreviations: N, number of randomized subjects; n, number of subjects within each category; SD, Standard Deviation; Min, Minimum; Max, Maximum.

Program Name: MUCUS-Shell11-V01
Output Location: .\Output\Shared\MUCUS-TFLS-Final-27MAR24-V01.pdf

Table 14.3.5.1: Summary of Vital Signs

	Naive (N=13)	Biologic (N=22)	Multiple Biologic (N=17)
Early Termination: PCHG			
N	0	2	0
Missing	-	-	-
Mean (SD)	-	0.3 (1.17)	-
Median (Min, Max)	-	0.3 (-1, 1)	-
Disease Flare: OBS			
N	0	0	0
Missing	-	-	-
Mean (SD)	-	-	-
Median (Min, Max)	-	-	-
Disease Flare: CHG			
N	0	0	0
Missing	-	-	-
Mean (SD)	-	-	-
Median (Min, Max)	-	-	-
Disease Flare: PCHG			
N	0	0	0
Missing	-	-	-
Mean (SD)	-	-	-
Median (Min, Max)	-	-	-
Safety Follow-Up: OBS			
N	7	8	9
Missing	6	14	8
Mean (SD)	36.3 (0.79)	36.4 (0.23)	36.5 (0.47)
Median (Min, Max)	36.2 (35, 38)	36.4 (36, 37)	36.5 (36, 37)

Abbreviations: N, number of randomized subjects; n, number of subjects within each category; SD, Standard Deviation; Min, Minimum; Max, Maximum.

Program Name: MUCUS-Shell11-V01
Output Location: .\Output\Shared\MUCUS-TFLS-Final-27MAR24-V01.pdf

Table 14.3.5.1: Summary of Vital Signs

	Naive (N=13)	Biologic (N=22)	Multiple Biologic (N=17)
Safety Follow-Up: CHG			
N	6	6	6
Missing	7	16	11
Mean (SD)	-0.2 (1.06)	-0.2 (0.22)	0.1 (0.85)
Median (Min, Max)	-0.3 (-2, 2)	-0.2 (-0, 0)	-0.0 (-1, 2)
Safety Follow-Up: PCHG			
N	6	6	6
Missing	7	16	11
Mean (SD)	-0.6 (2.90)	-0.4 (0.59)	0.2 (2.39)
Median (Min, Max)	-1.0 (-4, 4)	-0.5 (-1, 1)	-0.1 (-2, 5)
Weight (kg)			
Baseline: OBS			
N	9	17	11
Missing	4	5	6
Mean (SD)	69.8 (15.97)	72.8 (15.00)	73.1 (15.51)
Median (Min, Max)	78.0 (39, 84)	67.0 (49, 102)	72.0 (47, 105)
Week 8: OBS			
N	12	20	14
Missing	1	2	3
Mean (SD)	71.5 (16.22)	71.7 (14.25)	75.6 (15.85)
Median (Min, Max)	78.0 (41, 100)	68.0 (54, 102)	74.5 (49, 108)
Week 8: CHG			
N	9	15	10
Missing	4	7	7
Mean (SD)	1.0 (1.66)	0.6 (1.92)	1.3 (3.77)
Median (Min, Max)	1.0 (-1, 4)	0.0 (-2, 5)	2.5 (-8, 5)

Abbreviations: N, number of randomized subjects; n, number of subjects within each category; SD, Standard Deviation; Min, Minimum; Max, Maximum.

Table 14.3.5.1: Summary of Vital Signs

	Naive (N=13)	Biologic (N=22)	Multiple Biologic (N=17)
Week 8: PCHG			
N	9	15	10
Missing	4	7	7
Mean (SD)	1.8 (2.65)	1.0 (3.34)	2.1 (5.15)
Median (Min, Max)	1.2 (-1, 5)	0.0 (-4, 10)	3.6 (-10, 8)
Week 16: OBS			
N	9	18	13
Missing	4	4	4
Mean (SD)	72.6 (11.57)	71.8 (13.38)	73.6 (14.27)
Median (Min, Max)	79.0 (53, 83)	69.5 (54, 102)	72.0 (51, 94)
Week 16: CHG			
N	8	14	9
Missing	5	8	8
Mean (SD)	0.8 (1.83)	1.6 (3.59)	0.9 (5.46)
Median (Min, Max)	1.5 (-2, 3)	0.5 (-4, 10)	3.0 (-10, 6)
Week 16: PCHG			
N	8	14	9
Missing	5	8	8
Mean (SD)	1.3 (2.69)	2.7 (5.59)	2.0 (7.78)
Median (Min, Max)	1.9 (-2, 5)	0.5 (-5, 15)	4.7 (-13, 11)
Week 26: OBS			
N	7	13	6
Missing	6	9	11
Mean (SD)	74.6 (12.19)	75.4 (14.56)	75.0 (10.49)
Median (Min, Max)	82.0 (54, 83)	73.0 (56, 102)	71.0 (66, 94)

Abbreviations: N, number of randomized subjects; n, number of subjects within each category; SD, Standard Deviation; Min, Minimum; Max, Maximum.

Program Name: MUCUS-Shell11-V01
Output Location: .\Output\Shared\MUCUS-TFLS-Final-27MAR24-V01.pdf

Table 14.3.5.1: Summary of Vital Signs

	Naive (N=13)	Biologic (N=22)	Multiple Biologic (N=17)
Week 26: CHG			
N	6	10	5
Missing	7	12	12
Mean (SD)	1.2 (2.64)	1.9 (2.77)	4.0 (3.74)
Median (Min, Max)	2.0 (-3, 4)	1.0 (-1, 8)	2.0 (0, 8)
Week 26: PCHG			
N	6	10	5
Missing	7	12	12
Mean (SD)	1.9 (3.67)	2.8 (4.11)	6.4 (6.26)
Median (Min, Max)	2.5 (-4, 6)	1.2 (-1, 12)	3.1 (0, 13)
Week 36: OBS			
N	7	10	9
Missing	6	12	8
Mean (SD)	73.4 (16.60)	78.2 (14.58)	75.2 (13.61)
Median (Min, Max)	82.0 (45, 85)	77.5 (60, 102)	74.0 (52, 95)
Week 36: CHG			
N	7	9	7
Missing	6	13	10
Mean (SD)	2.9 (3.24)	2.2 (3.73)	3.7 (4.92)
Median (Min, Max)	4.0 (-3, 6)	1.0 (-1, 11)	4.0 (-6, 10)
Week 36: PCHG			
N	7	9	7
Missing	6	13	10
Mean (SD)	5.1 (5.92)	3.4 (5.71)	5.6 (7.16)
Median (Min, Max)	5.9 (-4, 15)	1.0 (-1, 17)	5.6 (-8, 16)

Abbreviations: N, number of randomized subjects; n, number of subjects within each category; SD, Standard Deviation; Min, Minimum; Max, Maximum.

Program Name: MUCUS-Shell11-V01
Output Location: .\Output\Shared\MUCUS-TFLS-Final-27MAR24-V01.pdf

Table 14.3.5.1: Summary of Vital Signs

	Naive (N=13)	Biologic (N=22)	Multiple Biologic (N=17)
Week 52: OBS			
N	9	13	10
Missing	4	9	7
Mean (SD)	72.2 (19.16)	72.5 (12.95)	78.2 (15.63)
Median (Min, Max)	78.0 (44, 107)	70.0 (56, 102)	77.5 (61, 115)
Week 52: CHG			
N	7	11	9
Missing	6	11	8
Mean (SD)	1.1 (3.34)	1.5 (5.03)	4.8 (6.00)
Median (Min, Max)	1.0 (-4, 5)	1.0 (-10, 11)	2.0 (-2, 15)
Week 52: PCHG			
N	7	11	9
Missing	6	11	8
Mean (SD)	2.9 (6.10)	2.7 (7.19)	7.8 (11.01)
Median (Min, Max)	1.7 (-5, 13)	1.8 (-13, 17)	3.1 (-2, 32)
Early Termination: OBS			
N	0	2	2
Missing	-	-	-
Mean (SD)	-	74.0 (8.49)	67.5 (21.92)
Median (Min, Max)	-	74.0 (68, 80)	67.5 (52, 83)
Early Termination: CHG			
N	0	2	0
Missing	-	-	-
Mean (SD)	-	0.0 (0.00)	-
Median (Min, Max)	-	0.0 (0, 0)	-

Abbreviations: N, number of randomized subjects; n, number of subjects within each category; SD, Standard Deviation; Min, Minimum; Max, Maximum.

Program Name: MUCUS-Shell11-V01
Output Location: .\Output\Shared\MUCUS-TFLS-Final-27MAR24-V01.pdf

Table 14.3.5.1: Summary of Vital Signs

	Naive (N=13)	Biologic (N=22)	Multiple Biologic (N=17)
Early Termination: PCHG			
N	0	2	0
Missing	-	-	-
Mean (SD)	-	0.0 (0.00)	-
Median (Min, Max)	-	0.0 (0, 0)	-
Disease Flare: OBS			
N	0	0	0
Missing	-	-	-
Mean (SD)	-	-	-
Median (Min, Max)	-	-	-
Disease Flare: CHG			
N	0	0	0
Missing	-	-	-
Mean (SD)	-	-	-
Median (Min, Max)	-	-	-
Disease Flare: PCHG			
N	0	0	0
Missing	-	-	-
Mean (SD)	-	-	-
Median (Min, Max)	-	-	-
Safety Follow-Up: OBS			
N	7	8	9
Missing	6	14	8
Mean (SD)	65.1 (14.19)	70.8 (7.87)	85.0 (14.05)
Median (Min, Max)	62.0 (44, 83)	69.5 (59, 84)	81.0 (68, 116)

Abbreviations: N, number of randomized subjects; n, number of subjects within each category; SD, Standard Deviation; Min, Minimum; Max, Maximum.

Program Name: MUCUS-Shell11-V01
Output Location: .\Output\Shared\MUCUS-TFLS-Final-27MAR24-V01.pdf

Table 14.3.5.1: Summary of Vital Signs

	Naive (N=13)	Biologic (N=22)	Multiple Biologic (N=17)
Safety Follow-Up: CHG			
N	6	6	6
Missing	7	16	11
Mean (SD)	1.3 (3.27)	-0.7 (4.89)	7.3 (7.66)
Median (Min, Max)	2.5 (-4, 5)	0.5 (-10, 4)	4.5 (0, 21)
Safety Follow-Up: PCHG			
N	6	6	6
Missing	7	16	11
Mean (SD)	3.3 (6.07)	-0.8 (6.18)	11.8 (16.54)
Median (Min, Max)	3.5 (-5, 13)	0.7 (-13, 5)	6.1 (0, 45)

Abbreviations: N, number of randomized subjects; n, number of subjects within each category; SD, Standard Deviation; Min, Minimum; Max, Maximum.

Program Name: MUCUS-Shell11-V01
Output Location: .\Output\Shared\MUCUS-TFLS-Final-27MAR24-V01.pdf

Table 14.3.5.2: Summary of Clinical Laboratory Test Results

	Naive (N=13)	Biologic (N=22)	Multiple Biologic (N=17)
Albumin			
Baseline: OBS			
N	11	22	15
Missing	2	0	2
Mean (SD)	39.0 (7.01)	34.9 (10.12)	35.1 (5.60)
Median (Min, Max)	39.4 (23, 47)	37.9 (4, 49)	36.6 (21, 43)
Week 8: OBS			
N	12	17	13
Missing	1	5	4
Mean (SD)	40.1 (7.07)	39.4 (5.85)	39.2 (3.57)
Median (Min, Max)	43.4 (24, 47)	39.6 (29, 51)	39.5 (31, 44)
Week 8: CHG			
N	10	17	12
Missing	3	5	5
Mean (SD)	1.7 (3.52)	5.9 (9.73)	4.4 (5.29)
Median (Min, Max)	1.3 (-2, 9)	2.6 (-3, 39)	5.0 (-4, 17)
Week 8: PCHG			
N	10	17	12
Missing	3	5	5
Mean (SD)	5.1 (9.90)	68.0 (221.82)	15.8 (23.02)
Median (Min, Max)	3.7 (-4, 27)	8.2 (-8, 924)	14.0 (-10, 81)
Week 16: OBS			
N	8	16	11
Missing	5	6	6
Mean (SD)	39.3 (9.85)	40.2 (4.64)	37.8 (3.66)
Median (Min, Max)	44.0 (21, 46)	41.2 (28, 45)	38.1 (31, 43)

Abbreviations: N, number of randomized subjects; n, number of subjects within each category; SD, Standard Deviation; Min, Minimum; Max, Maximum.

Program Name: MUCUS-Shell11-V01
Output Location: .\Output\Shared\MUCUS-TFLS-Final-27MAR24-V01.pdf

Table 14.3.5.2: Summary of Clinical Laboratory Test Results

	Naive (N=13)	Biologic (N=22)	Multiple Biologic (N=17)
Week 16: CHG			
N	7	16	10
Missing	6	6	7
Mean (SD)	0.3 (2.21)	4.6 (6.37)	3.7 (5.23)
Median (Min, Max)	-0.5 (-2, 3)	3.6 (-5, 20)	1.5 (-2, 14)
Week 16: PCHG			
N	7	16	10
Missing	6	6	7
Mean (SD)	0.1 (6.18)	17.9 (27.85)	13.8 (22.04)
Median (Min, Max)	-1.1 (-10, 7)	9.0 (-10, 105)	4.1 (-5, 67)
Week 26: OBS			
N	6	9	6
Missing	7	13	11
Mean (SD)	44.4 (3.22)	39.2 (7.33)	39.8 (6.08)
Median (Min, Max)	44.7 (40, 49)	41.3 (23, 48)	39.5 (32, 49)
Week 26: CHG			
N	5	9	5
Missing	8	13	12
Mean (SD)	2.1 (3.23)	1.9 (3.69)	7.1 (5.26)
Median (Min, Max)	1.0 (-1, 8)	1.1 (-4, 9)	5.4 (2, 16)
Week 26: PCHG			
N	5	9	5
Missing	8	13	12
Mean (SD)	5.4 (8.52)	5.9 (10.35)	21.3 (16.08)
Median (Min, Max)	2.2 (-2, 20)	4.1 (-9, 27)	14.8 (7, 46)

Abbreviations: N, number of randomized subjects; n, number of subjects within each category; SD, Standard Deviation; Min, Minimum; Max, Maximum.

Program Name: MUCUS-Shell11-V01
Output Location: .\Output\Shared\MUCUS-TFLS-Final-27MAR24-V01.pdf

Table 14.3.5.2: Summary of Clinical Laboratory Test Results

	Naive (N=13)	Biologic (N=22)	Multiple Biologic (N=17)
Week 36: OBS			
N	6	9	7
Missing	7	13	10
Mean (SD)	42.9 (4.38)	41.2 (6.74)	40.5 (3.80)
Median (Min, Max)	43.8 (37, 47)	43.5 (27, 48)	39.3 (36, 46)
Week 36: CHG			
N	6	9	6
Missing	7	13	11
Mean (SD)	1.2 (1.73)	3.8 (4.20)	6.9 (3.59)
Median (Min, Max)	1.0 (-1, 4)	3.4 (-1, 12)	6.0 (3, 13)
Week 36: PCHG			
N	6	9	6
Missing	7	13	11
Mean (SD)	2.9 (4.14)	11.6 (13.06)	20.8 (11.84)
Median (Min, Max)	2.3 (-3, 9)	8.1 (-2, 37)	17.8 (7, 37)
Week 52: OBS			
N	9	12	7
Missing	4	10	10
Mean (SD)	45.1 (2.97)	42.6 (4.68)	41.8 (5.83)
Median (Min, Max)	45.0 (39, 50)	43.4 (33, 49)	40.7 (33, 51)
Week 52: CHG			
N	7	12	6
Missing	6	10	11
Mean (SD)	4.9 (4.42)	9.7 (11.10)	7.1 (5.85)
Median (Min, Max)	3.0 (0, 12)	5.5 (-0, 39)	6.0 (2, 17)

Abbreviations: N, number of randomized subjects; n, number of subjects within each category; SD, Standard Deviation; Min, Minimum; Max, Maximum.

Program Name: MUCUS-Shell11-V01
Output Location: .\Output\Shared\MUCUS-TFLS-Final-27MAR24-V01.pdf

Table 14.3.5.2: Summary of Clinical Laboratory Test Results

	Naive (N=13)	Biologic (N=22)	Multiple Biologic (N=17)
Week 52: PCHG			
N	7	12	6
Missing	6	10	11
Mean (SD)	13.5 (13.47)	101.3 (260.83)	21.4 (17.88)
Median (Min, Max)	7.0 (0, 36)	14.9 (-0, 924)	18.8 (4, 49)
Early Termination: OBS			
N	0	2	2
Missing	-	-	-
Mean (SD)	-	40.7 (0.21)	38.2 (1.63)
Median (Min, Max)	-	40.7 (41, 41)	38.2 (37, 39)
Early Termination: CHG			
N	0	2	2
Missing	-	-	-
Mean (SD)	-	2.0 (4.03)	9.4 (9.40)
Median (Min, Max)	-	2.0 (-1, 5)	9.4 (3, 16)
Early Termination: PCHG			
N	0	2	2
Missing	-	-	-
Mean (SD)	-	5.9 (11.09)	41.8 (48.66)
Median (Min, Max)	-	5.9 (-2, 14)	41.8 (7, 76)
Disease Flare: OBS			
N	0	0	1
Missing	-	-	-
Mean (SD)	-	-	38.9 (-)
Median (Min, Max)	-	-	38.9 (39, 39)

Abbreviations: N, number of randomized subjects; n, number of subjects within each category; SD, Standard Deviation; Min, Minimum; Max, Maximum.

Program Name: MUCUS-Shell11-V01
Output Location: .\Output\Shared\MUCUS-TFLS-Final-27MAR24-V01.pdf

Table 14.3.5.2: Summary of Clinical Laboratory Test Results

	Naive (N=13)	Biologic (N=22)	Multiple Biologic (N=17)
Disease Flare: CHG			
N	0	0	1
Missing	-	-	-
Mean (SD)	-	-	0.2 (-)
Median (Min, Max)	-	-	0.2 (0, 0)
Disease Flare: PCHG			
N	0	0	1
Missing	-	-	-
Mean (SD)	-	-	0.5 (-)
Median (Min, Max)	-	-	0.5 (1, 1)
Safety Follow-Up: OBS			
N	6	5	9
Missing	7	17	8
Mean (SD)	42.0 (3.16)	36.9 (4.97)	40.1 (4.48)
Median (Min, Max)	43.5 (38, 45)	38.0 (29, 42)	40.6 (33, 46)
Safety Follow-Up: CHG			
N	4	5	9
Missing	9	17	8
Mean (SD)	0.3 (5.56)	5.4 (7.50)	5.7 (6.20)
Median (Min, Max)	-1.0 (-5, 8)	1.3 (0, 18)	7.0 (-4, 15)
Safety Follow-Up: PCHG			
N	4	5	9
Missing	9	17	8
Mean (SD)	1.7 (14.89)	26.0 (40.23)	20.4 (24.85)
Median (Min, Max)	-2.2 (-12, 23)	3.4 (0, 95)	17.9 (-10, 71)

Abbreviations: N, number of randomized subjects; n, number of subjects within each category; SD, Standard Deviation; Min, Minimum; Max, Maximum.

Table 14.3.5.2: Summary of Clinical Laboratory Test Results

	Naive (N=13)	Biologic (N=22)	Multiple Biologic (N=17)
C-Reactive Protein			
Baseline: OBS			
N	13	22	17
Missing	0	0	0
Mean (SD)	18.1 (16.75)	19.8 (29.56)	35.3 (49.40)
Median (Min, Max)	12.6 (1, 52)	7.4 (1, 113)	25.9 (0, 210)
Week 8: OBS			
N	12	20	14
Missing	1	2	3
Mean (SD)	12.9 (18.55)	10.1 (12.24)	9.8 (9.56)
Median (Min, Max)	6.6 (1, 67)	5.8 (1, 48)	4.6 (1, 31)
Week 8: CHG			
N	12	20	14
Missing	1	2	3
Mean (SD)	-5.6 (19.74)	-11.2 (24.47)	-28.9 (46.50)
Median (Min, Max)	-1.5 (-47, 33)	-1.4 (-76, 17)	-15.7 (-180, 1)
Week 8: PCHG			
N	12	20	14
Missing	1	2	3
Mean (SD)	-7.0 (75.77)	6.0 (102.16)	-27.2 (126.52)
Median (Min, Max)	-43.8 (-91, 110)	-21.7 (-91, 253)	-65.4 (-93, 400)
Week 16: OBS			
N	8	18	12
Missing	5	4	5
Mean (SD)	7.2 (7.74)	8.5 (10.81)	16.6 (18.87)

Abbreviations: N, number of randomized subjects; n, number of subjects within each category; SD, Standard Deviation; Min, Minimum; Max, Maximum.

Program Name: MUCUS-Shell11-V01
Output Location: .\Output\Shared\MUCUS-TFLS-Final-27MAR24-V01.pdf

Table 14.3.5.2: Summary of Clinical Laboratory Test Results

	Naive (N=13)	Biologic (N=22)	Multiple Biologic (N=17)
Median (Min, Max)	5.0 (1, 21)	4.0 (1, 39)	6.6 (1, 58)
Week 16: CHG			
N	8	18	12
Missing	5	4	5
Mean (SD)	-4.7 (11.18)	-14.6 (26.86)	-25.6 (48.60)
Median (Min, Max)	-1.0 (-26, 8)	-2.6 (-81, 12)	-7.2 (-165, 15)
Week 16: PCHG			
N	8	18	12
Missing	5	4	5
Mean (SD)	-20.3 (57.43)	-16.9 (91.63)	55.3 (256.93)
Median (Min, Max)	-40.3 (-85, 66)	-36.8 (-93, 294)	-58.8 (-93, 667)
Week 26: OBS			
N	6	10	6
Missing	7	12	11
Mean (SD)	1.8 (0.92)	24.0 (48.08)	8.6 (11.84)
Median (Min, Max)	1.9 (1, 3)	4.4 (1, 156)	4.5 (1, 33)
Week 26: CHG			
N	6	10	6
Missing	7	12	11
Mean (SD)	-10.4 (19.22)	1.6 (18.13)	-16.6 (23.54)
Median (Min, Max)	-1.7 (-49, 1)	-0.9 (-31, 43)	-5.4 (-50, 7)
Week 26: PCHG			
N	6	10	6
Missing	7	12	11
Mean (SD)	-38.5 (65.06)	-8.4 (70.47)	-55.7 (45.07)

Abbreviations: N, number of randomized subjects; n, number of subjects within each category; SD, Standard Deviation; Min, Minimum; Max, Maximum.

Program Name: MUCUS-Shell11-V01
Output Location: .\Output\Shared\MUCUS-TFLS-Final-27MAR24-V01.pdf

Table 14.3.5.2: Summary of Clinical Laboratory Test Results

	Naive (N=13)	Biologic (N=22)	Multiple Biologic (N=17)
Median (Min, Max)	-53.3 (-94, 80)	-15.7 (-84, 141)	-68.5 (-93, 26)
Week 36: OBS			
N	8	9	9
Missing	5	13	8
Mean (SD)	8.5 (11.28)	7.5 (13.66)	9.3 (11.09)
Median (Min, Max)	3.1 (1, 31)	4.0 (1, 44)	4.0 (1, 34)
Week 36: CHG			
N	8	9	9
Missing	5	13	8
Mean (SD)	-5.2 (6.45)	-12.7 (23.94)	-12.4 (19.92)
Median (Min, Max)	-3.3 (-19, 1)	-3.0 (-69, 3)	-5.6 (-50, 8)
Week 36: PCHG			
N	8	9	9
Missing	5	13	8
Mean (SD)	-29.0 (55.68)	-28.4 (80.91)	61.8 (246.87)
Median (Min, Max)	-41.5 (-79, 90)	-50.0 (-93, 176)	-48.7 (-93, 633)
Week 52: OBS			
N	9	13	10
Missing	4	9	7
Mean (SD)	7.6 (7.98)	4.6 (5.04)	9.9 (9.96)
Median (Min, Max)	5.4 (1, 21)	3.3 (1, 19)	4.1 (1, 28)
Week 52: CHG			
N	9	13	10
Missing	4	9	7
Mean (SD)	-14.9 (15.91)	-21.5 (33.52)	-21.5 (18.97)

Abbreviations: N, number of randomized subjects; n, number of subjects within each category; SD, Standard Deviation; Min, Minimum; Max, Maximum.

Program Name: MUCUS-Shell11-V01
Output Location: .\Output\Shared\MUCUS-TFLS-Final-27MAR24-V01.pdf

Table 14.3.5.2: Summary of Clinical Laboratory Test Results

	Naive (N=13)	Biologic (N=22)	Multiple Biologic (N=17)
Median (Min, Max)	-6.1 (-42, -1)	-3.0 (-94, 2)	-22.7 (-50, 3)
Week 52: PCHG			
N	9	13	10
Missing	4	9	7
Mean (SD)	-61.8 (23.87)	-26.8 (82.84)	23.1 (286.36)
Median (Min, Max)	-63.0 (-94, -22)	-60.0 (-99, 170)	-70.7 (-93, 833)
Early Termination: OBS			
N	0	3	2
Missing	-	-	-
Mean (SD)	-	2.0 (0.30)	75.4 (101.54)
Median (Min, Max)	-	2.0 (2, 2)	75.4 (4, 147)
Early Termination: CHG			
N	0	3	2
Missing	-	-	-
Mean (SD)	-	-3.7 (2.75)	-30.2 (46.46)
Median (Min, Max)	-	-3.6 (-7, -1)	-30.2 (-63, 3)
Early Termination: PCHG			
N	0	3	2
Missing	-	-	-
Mean (SD)	-	-57.9 (23.13)	135.0 (233.33)
Median (Min, Max)	-	-61.0 (-79, -33)	135.0 (-30, 300)
Disease Flare: OBS			
N	0	0	1
Missing	-	-	-
Mean (SD)	-	-	19.3 (-)

Abbreviations: N, number of randomized subjects; n, number of subjects within each category; SD, Standard Deviation; Min, Minimum; Max, Maximum.

Table 14.3.5.2: Summary of Clinical Laboratory Test Results

	Naive (N=13)	Biologic (N=22)	Multiple Biologic (N=17)
Median (Min, Max)	-	-	19.3 (19, 19)
Disease Flare: CHG			
N	0	0	1
Missing	-	-	-
Mean (SD)	-	-	11.9 (-)
Median (Min, Max)	-	-	11.9 (12, 12)
Disease Flare: PCHG			
N	0	0	1
Missing	-	-	-
Mean (SD)	-	-	160.8 (-)
Median (Min, Max)	-	-	160.8 (161, 161)
Safety Follow-Up: OBS			
N	7	8	10
Missing	6	14	7
Mean (SD)	4.0 (2.81)	6.3 (7.91)	20.3 (31.20)
Median (Min, Max)	4.6 (1, 9)	3.4 (1, 25)	5.9 (2, 91)
Safety Follow-Up: CHG			
N	7	8	10
Missing	6	14	7
Mean (SD)	-19.6 (18.50)	-23.3 (37.65)	-27.7 (41.69)
Median (Min, Max)	-17.7 (-46, -1)	-1.5 (-88, 2)	-29.8 (-120, 40)
Safety Follow-Up: PCHG			
N	7	8	10
Missing	6	14	7
Mean (SD)	-71.5 (17.09)	-19.5 (70.31)	-43.1 (74.79)

Abbreviations: N, number of randomized subjects; n, number of subjects within each category; SD, Standard Deviation; Min, Minimum; Max, Maximum.

Table 14.3.5.2: Summary of Clinical Laboratory Test Results

	Naive (N=13)	Biologic (N=22)	Multiple Biologic (N=17)
Median (Min, Max)	-67.0 (-89, -45)	-30.0 (-95, 78)	-70.7 (-94, 154)
Fecal Calprotectin			
Baseline: OBS			
N	12	20	15
Missing	1	2	2
Mean (SD)	648.0 (791.44)	875.3 (954.21)	1170.5 (986.69)
Median (Min, Max)	247.0 (50, 2000)	584.5 (5, 4030)	800.0 (15, 3000)
Week 8: OBS			
N	10	13	12
Missing	3	9	5
Mean (SD)	226.8 (231.68)	382.9 (529.73)	773.4 (851.40)
Median (Min, Max)	177.5 (35, 804)	154.0 (50, 2000)	406.0 (28, 2100)
Week 8: CHG			
N	10	13	11
Missing	3	9	6
Mean (SD)	-343.1 (654.65)	-457.8 (1163.07)	-288.3 (493.39)
Median (Min, Max)	-75.3 (-1783, 305)	-127.0 (-3797, 562)	-22.0 (-1440, 286)
Week 8: PCHG			
N	10	13	11
Missing	3	9	6
Mean (SD)	28.2 (211.43)	829.9 (3128.23)	-20.7 (51.99)
Median (Min, Max)	-50.7 (-90, 610)	-45.2 (-94, 11240)	-22.7 (-94, 101)
Week 16: OBS			
N	7	13	10
Missing	6	9	7

Abbreviations: N, number of randomized subjects; n, number of subjects within each category; SD, Standard Deviation; Min, Minimum; Max, Maximum.

Program Name: MUCUS-Shell11-V01
Output Location: .\Output\Shared\MUCUS-TFLS-Final-27MAR24-V01.pdf

Table 14.3.5.2: Summary of Clinical Laboratory Test Results

	Naive (N=13)	Biologic (N=22)	Multiple Biologic (N=17)
Mean (SD)	235.3 (216.03)	345.1 (533.37)	858.7 (727.58)
Median (Min, Max)	162.0 (74, 650)	206.0 (50, 2000)	723.7 (50, 2000)
Week 16: CHG			
N	7	13	8
Missing	6	9	9
Mean (SD)	12.8 (177.21)	-540.0 (1273.02)	-403.1 (587.14)
Median (Min, Max)	-7.0 (-182, 355)	-225.0 (-3824, 1795)	-50.0 (-1264, 119)
Week 16: PCHG			
N	7	13	8
Missing	6	9	9
Mean (SD)	96.0 (275.38)	434.6 (1562.25)	-8.9 (45.61)
Median (Min, Max)	-7.4 (-66, 710)	-77.0 (-95, 5560)	-2.4 (-63, 61)
Week 26: OBS			
N	5	7	6
Missing	8	15	11
Mean (SD)	97.8 (69.53)	163.1 (90.43)	605.8 (667.57)
Median (Min, Max)	80.9 (20, 186)	185.0 (63, 298)	489.5 (50, 1800)
Week 26: CHG			
N	5	7	5
Missing	8	15	12
Mean (SD)	-116.0 (113.73)	-513.8 (585.30)	-164.2 (313.89)
Median (Min, Max)	-125.0 (-269, 50)	-459.0 (-1398, 293)	-44.0 (-724, 0)
Week 26: PCHG			
N	5	7	5
Missing	8	15	12

Abbreviations: N, number of randomized subjects; n, number of subjects within each category; SD, Standard Deviation; Min, Minimum; Max, Maximum.

Program Name: MUCUS-Shell11-V01
Output Location: .\Output\Shared\MUCUS-TFLS-Final-27MAR24-V01.pdf

Table 14.3.5.2: Summary of Clinical Laboratory Test Results

	Naive (N=13)	Biologic (N=22)	Multiple Biologic (N=17)
Mean (SD)	-47.7 (49.99)	777.7 (2241.34)	-23.6 (39.86)
Median (Min, Max)	-62.7 (-84, 37)	-82.5 (-94, 5860)	-5.5 (-94, 0)
Week 36: OBS			
N	6	8	6
Missing	7	14	11
Mean (SD)	153.0 (147.79)	347.4 (592.43)	451.9 (493.80)
Median (Min, Max)	67.0 (50, 367)	166.0 (50, 1800)	268.0 (50, 1292)
Week 36: CHG			
N	6	8	5
Missing	7	14	12
Mean (SD)	-321.0 (689.78)	-690.0 (1366.23)	-459.6 (617.16)
Median (Min, Max)	-156.0 (-1663, 317)	-412.0 (-3760, 810)	-507.8 (-1317, 251)
Week 36: PCHG			
N	6	8	5
Missing	7	14	12
Mean (SD)	53.7 (286.15)	460.5 (1471.76)	14.9 (140.83)
Median (Min, Max)	-72.2 (-84, 634)	-79.9 (-94, 4100)	-28.2 (-94, 259)
Week 52: OBS			
N	6	6	8
Missing	7	16	9
Mean (SD)	676.7 (773.45)	375.5 (598.72)	826.3 (789.84)
Median (Min, Max)	369.5 (50, 2000)	159.5 (5, 1584)	654.3 (14, 2000)
Week 52: CHG			
N	6	5	7
Missing	7	17	10

Abbreviations: N, number of randomized subjects; n, number of subjects within each category; SD, Standard Deviation; Min, Minimum; Max, Maximum.

Program Name: MUCUS-Shell11-V01
Output Location: .\Output\Shared\MUCUS-TFLS-Final-27MAR24-V01.pdf

Table 14.3.5.2: Summary of Clinical Laboratory Test Results

	Naive (N=13)	Biologic (N=22)	Multiple Biologic (N=17)
Mean (SD)	-194.3 (463.97)	-1278.2 (1510.93)	-308.1 (930.44)
Median (Min, Max)	-172.0 (-829, 521)	-674.0 (-3837, 77)	-100.0 (-1724, 917)
Week 52: PCHG			
N	6	5	7
Missing	7	17	10
Mean (SD)	130.8 (447.36)	235.6 (729.20)	34.1 (139.41)
Median (Min, Max)	-52.7 (-77, 1042)	-84.3 (-99, 1540)	-4.8 (-91, 324)
Early Termination: OBS			
N	0	3	1
Missing	-	-	-
Mean (SD)	-	155.8 (96.32)	348.0 (-)
Median (Min, Max)	-	140.0 (68, 259)	348.0 (348, 348)
Early Termination: CHG			
N	0	3	1
Missing	-	-	-
Mean (SD)	-	-87.9 (235.88)	251.0 (-)
Median (Min, Max)	-	-141.0 (-293, 170)	251.0 (251, 251)
Early Termination: PCHG			
N	0	3	1
Missing	-	-	-
Mean (SD)	-	19.9 (148.97)	258.8 (-)
Median (Min, Max)	-	-50.2 (-81, 191)	258.8 (259, 259)
Disease Flare: OBS			
N	0	0	0
Missing	-	-	-

Abbreviations: N, number of randomized subjects; n, number of subjects within each category; SD, Standard Deviation; Min, Minimum; Max, Maximum.

Program Name: MUCUS-Shell11-V01
Output Location: .\Output\Shared\MUCUS-TFLS-Final-27MAR24-V01.pdf

Table 14.3.5.2: Summary of Clinical Laboratory Test Results

	Naive (N=13)	Biologic (N=22)	Multiple Biologic (N=17)
Mean (SD)	-	-	-
Median (Min, Max)	-	-	-
Disease Flare: CHG			
N	0	0	0
Missing	-	-	-
Mean (SD)	-	-	-
Median (Min, Max)	-	-	-
Disease Flare: PCHG			
N	0	0	0
Missing	-	-	-
Mean (SD)	-	-	-
Median (Min, Max)	-	-	-
Safety Follow-Up: OBS			
N	3	5	6
Missing	10	17	11
Mean (SD)	1386.4 (543.05)	679.5 (814.40)	743.9 (974.99)
Median (Min, Max)	1191.7 (968, 2000)	335.0 (10, 2000)	174.0 (12, 2000)
Safety Follow-Up: CHG			
N	3	5	5
Missing	10	17	12
Mean (SD)	678.6 (1525.11)	-1148.9 (1509.42)	-451.8 (810.08)
Median (Min, Max)	1098.1 (-1012, 1950)	-655.0 (-3695, 310)	-100.0 (-1896, 0)
Safety Follow-Up: PCHG			
N	3	5	5
Missing	10	17	12

Abbreviations: N, number of randomized subjects; n, number of subjects within each category; SD, Standard Deviation; Min, Minimum; Max, Maximum.

Table 14.3.5.2: Summary of Clinical Laboratory Test Results

	Naive (N=13)	Biologic (N=22)	Multiple Biologic (N=17)
Mean (SD)	1674.0 (2022.62)	-60.3 (49.38)	-36.7 (39.53)
Median (Min, Max)	1173.2 (-51, 3900)	-88.1 (-98, 18)	-26.9 (-95, 0)
Hemoglobin			
Baseline: OBS			
N	13	22	17
Missing	0	0	0
Mean (SD)	10.3 (2.99)	11.4 (3.91)	9.8 (2.87)
Median (Min, Max)	11.0 (7, 14)	12.3 (3, 19)	9.2 (6, 15)
Week 8: OBS			
N	12	20	14
Missing	1	2	3
Mean (SD)	10.9 (3.39)	11.8 (3.53)	10.2 (2.69)
Median (Min, Max)	11.0 (7, 15)	13.0 (4, 16)	9.4 (6, 15)
Week 8: CHG			
N	12	20	14
Missing	1	2	3
Mean (SD)	0.7 (1.07)	0.4 (1.24)	0.4 (0.87)
Median (Min, Max)	0.5 (-1, 2)	0.4 (-3, 3)	0.5 (-2, 1)
Week 8: PCHG			
N	12	20	14
Missing	1	2	3
Mean (SD)	7.2 (13.43)	7.9 (25.45)	5.4 (9.22)
Median (Min, Max)	3.6 (-13, 37)	3.1 (-18, 111)	5.1 (-19, 21)
Week 16: OBS			
N	8	18	12

Abbreviations: N, number of randomized subjects; n, number of subjects within each category; SD, Standard Deviation; Min, Minimum; Max, Maximum.

Program Name: MUCUS-Shell11-V01
Output Location: .\Output\Shared\MUCUS-TFLS-Final-27MAR24-V01.pdf

Table 14.3.5.2: Summary of Clinical Laboratory Test Results

	Naive (N=13)	Biologic (N=22)	Multiple Biologic (N=17)
Missing	5	4	5
Mean (SD)	12.0 (3.35)	12.2 (3.02)	10.2 (2.96)
Median (Min, Max)	13.8 (8, 15)	12.7 (7, 17)	10.0 (6, 16)
Week 16: CHG			
N	8	18	12
Missing	5	4	5
Mean (SD)	0.8 (0.79)	0.5 (1.63)	0.7 (0.98)
Median (Min, Max)	0.4 (0, 2)	0.3 (-4, 5)	0.6 (-1, 2)
Week 16: PCHG			
N	8	18	12
Missing	5	4	5
Mean (SD)	6.6 (6.56)	12.4 (39.49)	7.1 (9.11)
Median (Min, Max)	4.2 (1, 20)	2.6 (-21, 167)	9.7 (-13, 21)
Week 26: OBS			
N	6	10	6
Missing	7	12	11
Mean (SD)	13.3 (2.94)	12.5 (2.65)	12.3 (3.08)
Median (Min, Max)	14.3 (8, 15)	13.7 (8, 15)	13.1 (7, 15)
Week 26: CHG			
N	6	10	6
Missing	7	12	11
Mean (SD)	1.1 (0.71)	0.0 (1.94)	0.8 (0.57)
Median (Min, Max)	0.8 (1, 2)	0.1 (-5, 3)	1.1 (0, 1)
Week 26: PCHG			
N	6	10	6

Abbreviations: N, number of randomized subjects; n, number of subjects within each category; SD, Standard Deviation; Min, Minimum; Max, Maximum.

Program Name: MUCUS-Shell11-V01
Output Location: .\Output\Shared\MUCUS-TFLS-Final-27MAR24-V01.pdf

Table 14.3.5.2: Summary of Clinical Laboratory Test Results

	Naive (N=13)	Biologic (N=22)	Multiple Biologic (N=17)
Missing	7	12	11
Mean (SD)	9.2 (6.15)	2.1 (13.11)	6.6 (4.73)
Median (Min, Max)	6.7 (3, 20)	0.7 (-25, 28)	7.7 (0, 13)
Week 36: OBS			
N	8	10	9
Missing	5	12	8
Mean (SD)	12.5 (2.61)	13.0 (2.94)	11.9 (2.94)
Median (Min, Max)	13.3 (8, 15)	13.8 (8, 16)	12.5 (7, 16)
Week 36: CHG			
N	8	10	9
Missing	5	12	8
Mean (SD)	1.1 (0.83)	0.2 (1.67)	0.8 (1.02)
Median (Min, Max)	1.1 (0, 3)	0.0 (-3, 3)	0.7 (-1, 2)
Week 36: PCHG			
N	8	10	9
Missing	5	12	8
Mean (SD)	11.4 (12.91)	3.0 (12.51)	7.0 (9.06)
Median (Min, Max)	7.9 (1, 42)	0.3 (-18, 31)	5.1 (-5, 21)
Week 52: OBS			
N	9	13	10
Missing	4	9	7
Mean (SD)	11.1 (2.90)	11.9 (3.05)	10.6 (2.15)
Median (Min, Max)	12.3 (7, 15)	12.7 (7, 17)	9.8 (9, 14)
Week 52: CHG			
N	9	13	10

Abbreviations: N, number of randomized subjects; n, number of subjects within each category; SD, Standard Deviation; Min, Minimum; Max, Maximum.

Program Name: MUCUS-Shell11-V01
Output Location: .\Output\Shared\MUCUS-TFLS-Final-27MAR24-V01.pdf

Table 14.3.5.2: Summary of Clinical Laboratory Test Results

	Naive (N=13)	Biologic (N=22)	Multiple Biologic (N=17)
Missing	4	9	7
Mean (SD)	1.0 (0.89)	0.8 (2.03)	1.0 (0.74)
Median (Min, Max)	1.1 (-0, 2)	0.5 (-4, 5)	0.8 (0, 2)
Week 52: PCHG			
N	9	13	10
Missing	4	9	7
Mean (SD)	11.8 (11.85)	19.3 (47.43)	12.9 (11.80)
Median (Min, Max)	9.0 (-2, 37)	5.5 (-19, 170)	9.4 (2, 37)
Early Termination: OBS			
N	0	3	2
Missing	-	-	-
Mean (SD)	-	14.6 (0.90)	10.6 (4.31)
Median (Min, Max)	-	14.1 (14, 16)	10.6 (8, 14)
Early Termination: CHG			
N	0	3	2
Missing	-	-	-
Mean (SD)	-	0.0 (0.40)	2.0 (0.21)
Median (Min, Max)	-	0.1 (-0, 0)	2.0 (2, 2)
Early Termination: PCHG			
N	0	3	2
Missing	-	-	-
Mean (SD)	-	0.4 (2.73)	24.9 (9.42)
Median (Min, Max)	-	0.7 (-3, 3)	24.9 (18, 32)
Disease Flare: OBS			
N	0	0	1

Abbreviations: N, number of randomized subjects; n, number of subjects within each category; SD, Standard Deviation; Min, Minimum; Max, Maximum.

Table 14.3.5.2: Summary of Clinical Laboratory Test Results

	Naive (N=13)	Biologic (N=22)	Multiple Biologic (N=17)
Missing	-	-	-
Mean (SD)	-	-	14.3 (-)
Median (Min, Max)	-	-	14.3 (14, 14)
Disease Flare: CHG			
N	0	0	1
Missing	-	-	-
Mean (SD)	-	-	0.4 (-)
Median (Min, Max)	-	-	0.4 (0, 0)
Disease Flare: PCHG			
N	0	0	1
Missing	-	-	-
Mean (SD)	-	-	2.9 (-)
Median (Min, Max)	-	-	2.9 (3, 3)
Safety Follow-Up: OBS			
N	7	8	10
Missing	6	14	7
Mean (SD)	10.4 (3.28)	10.3 (3.78)	10.1 (2.69)
Median (Min, Max)	9.2 (8, 16)	10.5 (5, 15)	9.4 (7, 15)
Safety Follow-Up: CHG			
N	7	8	10
Missing	6	14	7
Mean (SD)	0.7 (0.75)	0.5 (1.70)	1.0 (0.75)
Median (Min, Max)	0.6 (0, 2)	-0.1 (-1, 4)	1.0 (-1, 2)
Safety Follow-Up: PCHG			
N	7	8	10

Abbreviations: N, number of randomized subjects; n, number of subjects within each category; SD, Standard Deviation; Min, Minimum; Max, Maximum.

Table 14.3.5.2: Summary of Clinical Laboratory Test Results

	Naive (N=13)	Biologic (N=22)	Multiple Biologic (N=17)
Missing	6	14	7
Mean (SD)	7.1 (5.49)	19.7 (58.08)	11.7 (10.44)
Median (Min, Max)	7.0 (0, 17)	-2.3 (-6, 163)	10.2 (-6, 31)
Hematocrit			
Baseline: OBS			
N	13	22	17
Missing	0	0	0
Mean (SD)	23.7 (19.44)	30.9 (17.54)	22.6 (19.35)
Median (Min, Max)	35.0 (0, 44)	38.0 (0, 48)	35.5 (0, 45)
Week 8: OBS			
N	12	20	14
Missing	1	2	3
Mean (SD)	27.6 (20.20)	32.0 (17.36)	20.1 (20.83)
Median (Min, Max)	40.1 (0, 45)	39.5 (0, 48)	15.1 (0, 47)
Week 8: CHG			
N	12	20	14
Missing	1	2	3
Mean (SD)	4.8 (10.32)	2.1 (4.54)	-1.8 (8.89)
Median (Min, Max)	1.1 (-2, 37)	0.8 (-2, 20)	0.1 (-32, 4)
Week 8: PCHG			
N	12	20	14
Missing	1	2	3
Mean (SD)	809.4 (2779.95)	439.5 (1919.91)	-2.2 (29.38)
Median (Min, Max)	8.0 (-11, 9637)	5.3 (-4, 8596)	4.6 (-99, 29)
Week 16: OBS			

Abbreviations: N, number of randomized subjects; n, number of subjects within each category; SD, Standard Deviation; Min, Minimum; Max, Maximum.

Program Name: MUCUS-Shell11-V01
Output Location: .\Output\Shared\MUCUS-TFLS-Final-27MAR24-V01.pdf

Table 14.3.5.2: Summary of Clinical Laboratory Test Results

	Naive (N=13)	Biologic (N=22)	Multiple Biologic (N=17)
N	8	18	12
Missing	5	4	5
Mean (SD)	26.1 (21.36)	32.1 (17.82)	19.5 (20.29)
Median (Min, Max)	39.3 (0, 44)	40.0 (0, 49)	16.4 (0, 47)
Week 16: CHG			
N	8	18	12
Missing	5	4	5
Mean (SD)	-3.5 (22.99)	1.2 (13.89)	-2.6 (26.60)
Median (Min, Max)	-0.1 (-35, 38)	0.6 (-37, 44)	-0.1 (-45, 47)
Week 16: PCHG			
N	8	18	12
Missing	5	4	5
Mean (SD)	1214.1 (3509.95)	568.2 (2387.31)	1821.5 (4323.20)
Median (Min, Max)	-0.2 (-99, 9900)	5.4 (-99, 10133)	2.1 (-99, 11650)
Week 26: OBS			
N	6	10	6
Missing	7	12	11
Mean (SD)	34.7 (17.01)	32.5 (17.09)	34.4 (17.14)
Median (Min, Max)	41.0 (0, 45)	39.9 (0, 44)	39.9 (0, 46)
Week 26: CHG			
N	6	10	6
Missing	7	12	11
Mean (SD)	1.0 (2.55)	-2.9 (12.10)	14.2 (19.91)
Median (Min, Max)	0.0 (-1, 5)	0.0 (-37, 6)	3.0 (-0, 44)
Week 26: PCHG			

Abbreviations: N, number of randomized subjects; n, number of subjects within each category; SD, Standard Deviation; Min, Minimum; Max, Maximum.

Program Name: MUCUS-Shell11-V01
Output Location: .\Output\Shared\MUCUS-TFLS-Final-27MAR24-V01.pdf

Table 14.3.5.2: Summary of Clinical Laboratory Test Results

	Naive (N=13)	Biologic (N=22)	Multiple Biologic (N=17)
N	6	10	6
Missing	7	12	11
Mean (SD)	3.2 (6.37)	-7.0 (33.00)	3516.2 (5447.50)
Median (Min, Max)	1.4 (-3, 14)	1.4 (-99, 19)	7.2 (-3, 10900)
Week 36: OBS			
N	8	10	9
Missing	5	12	8
Mean (SD)	31.6 (19.36)	37.9 (13.72)	31.5 (17.91)
Median (Min, Max)	41.1 (0, 45)	40.8 (0, 48)	39.0 (0, 45)
Week 36: CHG			
N	8	10	9
Missing	5	12	8
Mean (SD)	2.0 (21.41)	2.0 (17.25)	10.1 (17.43)
Median (Min, Max)	-0.1 (-35, 45)	2.0 (-37, 36)	1.4 (-1, 43)
Week 36: PCHG			
N	8	10	9
Missing	5	12	8
Mean (SD)	1632.4 (4648.02)	985.6 (3132.39)	2413.1 (4782.23)
Median (Min, Max)	2.4 (-99, 13135)	5.3 (-99, 9900)	3.4 (-3, 11043)
Week 52: OBS			
N	9	13	10
Missing	4	9	7
Mean (SD)	26.1 (19.41)	32.4 (18.49)	20.7 (21.50)
Median (Min, Max)	37.1 (0, 43)	39.9 (0, 48)	18.7 (0, 47)
Week 52: CHG			

Abbreviations: N, number of randomized subjects; n, number of subjects within each category; SD, Standard Deviation; Min, Minimum; Max, Maximum.

Table 14.3.5.2: Summary of Clinical Laboratory Test Results

	Naive (N=13)	Biologic (N=22)	Multiple Biologic (N=17)
N	9	13	10
Missing	4	9	7
Mean (SD)	0.7 (2.10)	5.0 (10.52)	-2.3 (27.26)
Median (Min, Max)	0.1 (-3, 4)	2.0 (-1, 39)	0.3 (-37, 41)
Week 52: PCHG			
N	9	13	10
Missing	4	9	7
Mean (SD)	8.1 (10.52)	843.5 (2971.78)	2062.7 (4424.95)
Median (Min, Max)	9.3 (-7, 29)	6.4 (-2, 10733)	2.6 (-99, 10757)
Early Termination: OBS			
N	0	3	2
Missing	-	-	-
Mean (SD)	-	30.1 (25.88)	20.6 (28.66)
Median (Min, Max)	-	41.8 (0, 48)	20.6 (0, 41)
Early Termination: CHG			
N	0	3	2
Missing	-	-	-
Mean (SD)	-	-13.8 (23.59)	2.7 (3.75)
Median (Min, Max)	-	-0.3 (-41, 0)	2.7 (0, 5)
Early Termination: PCHG			
N	0	3	2
Missing	-	-	-
Mean (SD)	-	-33.2 (56.97)	23.7 (11.97)
Median (Min, Max)	-	-0.7 (-99, 0)	23.7 (15, 32)
Disease Flare: OBS			

Abbreviations: N, number of randomized subjects; n, number of subjects within each category; SD, Standard Deviation; Min, Minimum; Max, Maximum.

Program Name: MUCUS-Shell11-V01
Output Location: .\Output\Shared\MUCUS-TFLS-Final-27MAR24-V01.pdf

Table 14.3.5.2: Summary of Clinical Laboratory Test Results

	Naive (N=13)	Biologic (N=22)	Multiple Biologic (N=17)
N	0	0	1
Missing	-	-	-
Mean (SD)	-	-	42.5 (-)
Median (Min, Max)	-	-	42.5 (43, 43)
Disease Flare: CHG			
N	0	0	1
Missing	-	-	-
Mean (SD)	-	-	0.6 (-)
Median (Min, Max)	-	-	0.6 (1, 1)
Disease Flare: PCHG			
N	0	0	1
Missing	-	-	-
Mean (SD)	-	-	1.4 (-)
Median (Min, Max)	-	-	1.4 (1, 1)
Safety Follow-Up: OBS			
N	7	8	10
Missing	6	14	7
Mean (SD)	23.9 (22.34)	22.9 (19.81)	20.5 (21.31)
Median (Min, Max)	34.0 (0, 46)	29.0 (0, 46)	18.2 (0, 46)
Safety Follow-Up: CHG			
N	7	8	10
Missing	6	14	7
Mean (SD)	2.2 (23.08)	6.8 (14.04)	1.0 (32.56)
Median (Min, Max)	-0.0 (-35, 45)	0.1 (-2, 36)	-0.1 (-45, 46)
Safety Follow-Up: PCHG			

Abbreviations: N, number of randomized subjects; n, number of subjects within each category; SD, Standard Deviation; Min, Minimum; Max, Maximum.

Program Name: MUCUS-Shell11-V01
Output Location: .\Output\Shared\MUCUS-TFLS-Final-27MAR24-V01.pdf

Table 14.3.5.2: Summary of Clinical Laboratory Test Results

	Naive (N=13)	Biologic (N=22)	Multiple Biologic (N=17)
N	7	8	10
Missing	6	14	7
Mean (SD)	1504.6 (4017.19)	2436.0 (4474.45)	3199.4 (5219.47)
Median (Min, Max)	-1.4 (-99, 10614)	2.5 (-5, 9900)	0.8 (-99, 11120)
Mean Corpuscular Hemoglobin			
Baseline: OBS			
N	13	22	17
Missing	0	0	0
Mean (SD)	16.2 (13.97)	26.2 (31.94)	13.6 (13.28)
Median (Min, Max)	25.9 (2, 32)	28.2 (2, 157)	1.9 (1, 32)
Week 8: OBS			
N	12	20	13
Missing	1	2	4
Mean (SD)	15.1 (13.96)	20.9 (12.92)	12.8 (12.67)
Median (Min, Max)	14.5 (2, 30)	28.1 (1, 33)	1.9 (1, 31)
Week 8: CHG			
N	12	20	13
Missing	1	2	4
Mean (SD)	0.2 (0.47)	0.0 (0.46)	-0.3 (1.08)
Median (Min, Max)	0.1 (-0, 1)	0.0 (-1, 1)	-0.0 (-2, 2)
Week 8: PCHG			
N	12	20	13
Missing	1	2	4
Mean (SD)	2.3 (3.37)	-0.2 (3.35)	-1.1 (4.97)
Median (Min, Max)	2.6 (-3, 8)	0.2 (-12, 4)	-1.6 (-10, 10)

Abbreviations: N, number of randomized subjects; n, number of subjects within each category; SD, Standard Deviation; Min, Minimum; Max, Maximum.

Program Name: MUCUS-Shell11-V01
Output Location: .\Output\Shared\MUCUS-TFLS-Final-27MAR24-V01.pdf

Table 14.3.5.2: Summary of Clinical Laboratory Test Results

	Naive (N=13)	Biologic (N=22)	Multiple Biologic (N=17)
Week 16: OBS			
N	8	18	12
Missing	5	4	5
Mean (SD)	18.8 (14.06)	21.6 (12.76)	13.8 (12.99)
Median (Min, Max)	28.3 (2, 30)	28.7 (2, 32)	11.9 (1, 32)
Week 16: CHG			
N	8	18	12
Missing	5	4	5
Mean (SD)	0.3 (0.78)	0.1 (0.91)	-0.2 (1.07)
Median (Min, Max)	0.1 (-1, 2)	0.1 (-2, 2)	-0.0 (-3, 2)
Week 16: PCHG			
N	8	18	12
Missing	5	4	5
Mean (SD)	2.0 (3.56)	0.3 (3.45)	-1.1 (6.75)
Median (Min, Max)	1.9 (-3, 7)	0.6 (-6, 5)	-0.6 (-14, 9)
Week 26: OBS			
N	6	10	6
Missing	7	12	11
Mean (SD)	25.0 (11.45)	24.2 (11.94)	22.6 (11.01)
Median (Min, Max)	29.7 (2, 30)	29.5 (2, 33)	25.8 (2, 32)
Week 26: CHG			
N	6	10	6
Missing	7	12	11
Mean (SD)	1.4 (1.41)	0.2 (1.28)	-0.0 (1.60)
Median (Min, Max)	1.3 (-0, 3)	0.0 (-2, 2)	0.1 (-2, 3)

Abbreviations: N, number of randomized subjects; n, number of subjects within each category; SD, Standard Deviation; Min, Minimum; Max, Maximum.

Program Name: MUCUS-Shell11-V01
Output Location: .\Output\Shared\MUCUS-TFLS-Final-27MAR24-V01.pdf

Table 14.3.5.2: Summary of Clinical Laboratory Test Results

	Naive (N=13)	Biologic (N=22)	Multiple Biologic (N=17)
Week 26: PCHG			
N	6	10	6
Missing	7	12	11
Mean (SD)	5.1 (5.60)	0.8 (4.87)	0.6 (7.14)
Median (Min, Max)	4.3 (-1, 13)	0.3 (-8, 8)	1.1 (-9, 12)
Week 36: OBS			
N	8	10	9
Missing	5	12	8
Mean (SD)	22.6 (12.86)	23.7 (11.79)	21.2 (11.79)
Median (Min, Max)	29.5 (2, 30)	29.1 (2, 32)	23.9 (1, 32)
Week 36: CHG			
N	8	10	9
Missing	5	12	8
Mean (SD)	1.2 (1.19)	-0.5 (1.67)	0.0 (2.23)
Median (Min, Max)	0.8 (0, 4)	-0.1 (-4, 3)	0.2 (-5, 3)
Week 36: PCHG			
N	8	10	9
Missing	5	12	8
Mean (SD)	6.6 (4.63)	-1.8 (6.11)	0.6 (10.14)
Median (Min, Max)	5.9 (1, 14)	-0.5 (-12, 9)	1.4 (-20, 14)
Week 52: OBS			
N	9	13	10
Missing	4	9	7
Mean (SD)	17.2 (14.61)	18.4 (13.77)	11.8 (13.17)
Median (Min, Max)	29.0 (2, 30)	25.9 (2, 32)	1.9 (2, 31)

Abbreviations: N, number of randomized subjects; n, number of subjects within each category; SD, Standard Deviation; Min, Minimum; Max, Maximum.

Program Name: MUCUS-Shell11-V01
Output Location: .\Output\Shared\MUCUS-TFLS-Final-27MAR24-V01.pdf

Table 14.3.5.2: Summary of Clinical Laboratory Test Results

	Naive (N=13)	Biologic (N=22)	Multiple Biologic (N=17)
Week 52: CHG			
N	9	13	10
Missing	4	9	7
Mean (SD)	1.0 (1.23)	-0.5 (1.75)	0.2 (0.92)
Median (Min, Max)	0.6 (0, 4)	0.0 (-5, 3)	0.1 (-1, 2)
Week 52: PCHG			
N	9	13	10
Missing	4	9	7
Mean (SD)	6.4 (5.03)	-0.5 (6.85)	4.7 (8.11)
Median (Min, Max)	3.5 (2, 14)	0.0 (-16, 11)	3.2 (-5, 20)
Early Termination: OBS			
N	0	3	2
Missing	-	-	-
Mean (SD)	-	29.6 (1.11)	16.1 (20.23)
Median (Min, Max)	-	29.4 (29, 31)	16.1 (2, 30)
Early Termination: CHG			
N	0	3	2
Missing	-	-	-
Mean (SD)	-	0.4 (0.40)	1.2 (1.73)
Median (Min, Max)	-	0.5 (0, 1)	1.2 (-0, 2)
Early Termination: PCHG			
N	0	3	2
Missing	-	-	-
Mean (SD)	-	1.5 (1.35)	3.2 (7.61)
Median (Min, Max)	-	1.7 (0, 3)	3.2 (-2, 9)

Abbreviations: N, number of randomized subjects; n, number of subjects within each category; SD, Standard Deviation; Min, Minimum; Max, Maximum.

Program Name: MUCUS-Shell11-V01
Output Location: .\Output\Shared\MUCUS-TFLS-Final-27MAR24-V01.pdf

Table 14.3.5.2: Summary of Clinical Laboratory Test Results

	Naive (N=13)	Biologic (N=22)	Multiple Biologic (N=17)
Disease Flare: OBS			
N	0	0	1
Missing	-	-	-
Mean (SD)	-	-	28.9 (-)
Median (Min, Max)	-	-	28.9 (29, 29)
Disease Flare: CHG			
N	0	0	1
Missing	-	-	-
Mean (SD)	-	-	0.3 (-)
Median (Min, Max)	-	-	0.3 (0, 0)
Disease Flare: PCHG			
N	0	0	1
Missing	-	-	-
Mean (SD)	-	-	1.0 (-)
Median (Min, Max)	-	-	1.0 (1, 1)
Safety Follow-Up: OBS			
N	7	8	10
Missing	6	14	7
Mean (SD)	13.8 (14.98)	15.4 (14.60)	9.7 (12.81)
Median (Min, Max)	1.9 (2, 31)	13.8 (2, 31)	1.9 (2, 31)
Safety Follow-Up: CHG			
N	7	8	10
Missing	6	14	7
Mean (SD)	0.7 (1.10)	-0.7 (1.37)	0.3 (1.11)
Median (Min, Max)	0.3 (-0, 3)	-0.0 (-4, 0)	0.1 (-1, 3)

Abbreviations: N, number of randomized subjects; n, number of subjects within each category; SD, Standard Deviation; Min, Minimum; Max, Maximum.

Program Name: MUCUS-Shell11-V01
Output Location: .\Output\Shared\MUCUS-TFLS-Final-27MAR24-V01.pdf

Table 14.3.5.2: Summary of Clinical Laboratory Test Results

	Naive (N=13)	Biologic (N=22)	Multiple Biologic (N=17)
Safety Follow-Up: PCHG			
N	7	8	10
Missing	6	14	7
Mean (SD)	5.1 (5.97)	-1.4 (5.76)	5.8 (7.91)
Median (Min, Max)	2.1 (-1, 16)	-0.0 (-12, 7)	4.2 (-3, 22)
Mean Corpuscular Hemoglobin Concentration			
Baseline: OBS			
N	13	22	17
Missing	0	0	0
Mean (SD)	27.0 (6.65)	29.3 (6.13)	25.7 (6.13)
Median (Min, Max)	31.3 (19, 34)	32.6 (19, 35)	21.3 (19, 34)
Week 8: OBS			
N	12	20	14
Missing	1	2	3
Mean (SD)	26.7 (6.81)	29.4 (5.88)	26.0 (6.42)
Median (Min, Max)	25.9 (20, 35)	32.3 (18, 34)	25.9 (19, 34)
Week 8: CHG			
N	12	20	14
Missing	1	2	3
Mean (SD)	0.3 (0.72)	-0.1 (0.96)	0.1 (1.03)
Median (Min, Max)	0.2 (-1, 1)	0.0 (-3, 2)	-0.2 (-2, 2)
Week 8: PCHG			
N	12	20	14
Missing	1	2	3
Mean (SD)	0.9 (2.51)	-0.1 (3.03)	0.1 (3.84)
Median (Min, Max)	0.7 (-3, 4)	0.0 (-7, 5)	-0.7 (-6, 8)

Abbreviations: N, number of randomized subjects; n, number of subjects within each category; SD, Standard Deviation; Min, Minimum; Max, Maximum.

Program Name: MUCUS-Shell11-V01
Output Location: .\Output\Shared\MUCUS-TFLS-Final-27MAR24-V01.pdf

Table 14.3.5.2: Summary of Clinical Laboratory Test Results

	Naive (N=13)	Biologic (N=22)	Multiple Biologic (N=17)
Week 16: OBS			
N	8	18	12
Missing	5	4	5
Mean (SD)	28.7 (6.95)	29.8 (5.96)	26.0 (6.55)
Median (Min, Max)	32.4 (20, 36)	33.1 (20, 35)	25.4 (19, 34)
Week 16: CHG			
N	8	18	12
Missing	5	4	5
Mean (SD)	0.4 (1.32)	0.0 (1.75)	0.3 (1.01)
Median (Min, Max)	0.3 (-2, 2)	0.1 (-5, 3)	0.3 (-1, 2)
Week 16: PCHG			
N	8	18	12
Missing	5	4	5
Mean (SD)	1.4 (4.06)	0.3 (5.59)	1.0 (4.06)
Median (Min, Max)	1.5 (-6, 7)	0.4 (-16, 9)	1.3 (-6, 7)
Week 26: OBS			
N	6	10	6
Missing	7	12	11
Mean (SD)	32.4 (5.84)	31.0 (5.41)	30.4 (5.40)
Median (Min, Max)	34.5 (21, 36)	33.3 (21, 35)	32.5 (20, 34)
Week 26: CHG			
N	6	10	6
Missing	7	12	11
Mean (SD)	1.7 (1.43)	0.3 (1.33)	0.6 (1.03)
Median (Min, Max)	2.0 (-0, 3)	0.3 (-2, 3)	0.5 (-0, 2)

Abbreviations: N, number of randomized subjects; n, number of subjects within each category; SD, Standard Deviation; Min, Minimum; Max, Maximum.

Program Name: MUCUS-Shell11-V01
Output Location: .\Output\Shared\MUCUS-TFLS-Final-27MAR24-V01.pdf

Table 14.3.5.2: Summary of Clinical Laboratory Test Results

	Naive (N=13)	Biologic (N=22)	Multiple Biologic (N=17)
Week 26: PCHG			
N	6	10	6
Missing	7	12	11
Mean (SD)	5.6 (4.37)	1.0 (4.12)	2.1 (3.45)
Median (Min, Max)	6.0 (-1, 11)	1.3 (-6, 8)	1.4 (-1, 8)
Week 36: OBS			
N	8	10	9
Missing	5	12	8
Mean (SD)	30.7 (6.27)	31.0 (5.41)	29.6 (5.95)
Median (Min, Max)	33.8 (20, 36)	33.1 (21, 35)	32.1 (19, 35)
Week 36: CHG			
N	8	10	9
Missing	5	12	8
Mean (SD)	1.1 (1.02)	-0.2 (1.22)	0.6 (1.12)
Median (Min, Max)	1.0 (-0, 3)	-0.4 (-2, 3)	0.9 (-1, 3)
Week 36: PCHG			
N	8	10	9
Missing	5	12	8
Mean (SD)	3.9 (3.32)	-0.4 (3.76)	2.0 (3.95)
Median (Min, Max)	3.9 (-0, 9)	-1.7 (-5, 8)	2.7 (-5, 8)
Week 52: OBS			
N	9	13	10
Missing	4	9	7
Mean (SD)	28.1 (7.05)	28.7 (6.62)	25.5 (6.16)
Median (Min, Max)	33.0 (20, 36)	32.8 (20, 35)	21.3 (20, 34)

Abbreviations: N, number of randomized subjects; n, number of subjects within each category; SD, Standard Deviation; Min, Minimum; Max, Maximum.

Program Name: MUCUS-Shell11-V01
Output Location: .\Output\Shared\MUCUS-TFLS-Final-27MAR24-V01.pdf

Table 14.3.5.2: Summary of Clinical Laboratory Test Results

	Naive (N=13)	Biologic (N=22)	Multiple Biologic (N=17)
Week 52: CHG			
N	9	13	10
Missing	4	9	7
Mean (SD)	1.0 (1.01)	0.3 (1.13)	0.7 (0.68)
Median (Min, Max)	0.6 (-0, 3)	0.0 (-1, 3)	0.7 (-0, 2)
Week 52: PCHG			
N	9	13	10
Missing	4	9	7
Mean (SD)	3.6 (3.33)	1.1 (3.84)	3.0 (3.04)
Median (Min, Max)	2.9 (-1, 10)	0.0 (-4, 9)	2.6 (-1, 7)
Early Termination: OBS			
N	0	3	2
Missing	-	-	-
Mean (SD)	-	33.8 (1.15)	26.7 (9.33)
Median (Min, Max)	-	33.7 (33, 35)	26.7 (20, 33)
Early Termination: CHG			
N	0	3	2
Missing	-	-	-
Mean (SD)	-	0.7 (1.10)	0.3 (0.85)
Median (Min, Max)	-	1.2 (-1, 1)	0.3 (-0, 1)
Early Termination: PCHG			
N	0	3	2
Missing	-	-	-
Mean (SD)	-	2.0 (3.32)	0.7 (3.00)
Median (Min, Max)	-	3.7 (-2, 4)	0.7 (-1, 3)

Abbreviations: N, number of randomized subjects; n, number of subjects within each category; SD, Standard Deviation; Min, Minimum; Max, Maximum.

Program Name: MUCUS-Shell11-V01
Output Location: .\Output\Shared\MUCUS-TFLS-Final-27MAR24-V01.pdf

Table 14.3.5.2: Summary of Clinical Laboratory Test Results

	Naive (N=13)	Biologic (N=22)	Multiple Biologic (N=17)
Disease Flare: OBS			
N	0	0	1
Missing	-	-	-
Mean (SD)	-	-	33.6 (-)
Median (Min, Max)	-	-	33.6 (34, 34)
Disease Flare: CHG			
N	0	0	1
Missing	-	-	-
Mean (SD)	-	-	0.4 (-)
Median (Min, Max)	-	-	0.4 (0, 0)
Disease Flare: PCHG			
N	0	0	1
Missing	-	-	-
Mean (SD)	-	-	1.2 (-)
Median (Min, Max)	-	-	1.2 (1, 1)
Safety Follow-Up: OBS			
N	7	8	10
Missing	6	14	7
Mean (SD)	26.4 (6.86)	27.6 (6.71)	24.7 (6.26)
Median (Min, Max)	21.8 (21, 34)	26.9 (21, 36)	20.9 (20, 35)
Safety Follow-Up: CHG			
N	7	8	10
Missing	6	14	7
Mean (SD)	0.7 (0.83)	0.6 (1.16)	0.8 (1.01)
Median (Min, Max)	0.5 (-0, 2)	0.2 (-1, 2)	0.8 (-0, 3)

Abbreviations: N, number of randomized subjects; n, number of subjects within each category; SD, Standard Deviation; Min, Minimum; Max, Maximum.

Program Name: MUCUS-Shell11-V01
Output Location: .\Output\Shared\MUCUS-TFLS-Final-27MAR24-V01.pdf

Table 14.3.5.2: Summary of Clinical Laboratory Test Results

	Naive (N=13)	Biologic (N=22)	Multiple Biologic (N=17)
Safety Follow-Up: PCHG			
N	7	8	10
Missing	6	14	7
Mean (SD)	2.9 (2.85)	2.5 (5.06)	3.4 (4.20)
Median (Min, Max)	2.5 (-0, 8)	0.7 (-2, 13)	2.8 (-2, 9)
Mean Corpuscular Volume			
Baseline: OBS			
N	13	22	17
Missing	0	0	0
Mean (SD)	86.1 (3.97)	86.8 (4.37)	84.0 (7.39)
Median (Min, Max)	85.1 (80, 94)	87.0 (75, 95)	86.4 (70, 96)
Week 8: OBS			
N	12	20	14
Missing	1	2	3
Mean (SD)	86.6 (3.69)	87.4 (3.85)	83.1 (7.51)
Median (Min, Max)	86.8 (82, 94)	87.8 (79, 95)	84.7 (72, 95)
Week 8: CHG			
N	12	20	14
Missing	1	2	3
Mean (SD)	1.2 (3.40)	0.0 (2.97)	-0.9 (2.71)
Median (Min, Max)	1.0 (-5, 8)	0.0 (-8, 7)	-0.9 (-6, 3)
Week 8: PCHG			
N	12	20	14
Missing	1	2	3
Mean (SD)	1.5 (4.10)	0.1 (3.49)	-1.1 (3.39)

Abbreviations: N, number of randomized subjects; n, number of subjects within each category; SD, Standard Deviation; Min, Minimum; Max, Maximum.

Program Name: MUCUS-Shell11-V01
Output Location: .\Output\Shared\MUCUS-TFLS-Final-27MAR24-V01.pdf

Table 14.3.5.2: Summary of Clinical Laboratory Test Results

	Naive (N=13)	Biologic (N=22)	Multiple Biologic (N=17)
Median (Min, Max)	1.1 (-6, 10)	0.0 (-9, 9)	-1.0 (-8, 4)
Week 16: OBS			
N	8	18	12
Missing	5	4	5
Mean (SD)	87.4 (4.28)	84.4 (13.00)	81.9 (8.06)
Median (Min, Max)	87.4 (81, 95)	87.0 (34, 93)	83.0 (72, 94)
Week 16: CHG			
N	8	18	12
Missing	5	4	5
Mean (SD)	0.5 (2.72)	-3.5 (13.14)	-1.6 (4.12)
Median (Min, Max)	0.2 (-4, 4)	-1.0 (-55, 6)	-1.3 (-8, 4)
Week 16: PCHG			
N	8	18	12
Missing	5	4	5
Mean (SD)	0.5 (3.16)	-3.8 (14.84)	-1.9 (4.97)
Median (Min, Max)	0.2 (-5, 5)	-1.1 (-62, 7)	-1.4 (-9, 5)
Week 26: OBS			
N	6	10	6
Missing	7	12	11
Mean (SD)	84.6 (2.64)	88.7 (3.69)	81.3 (8.86)
Median (Min, Max)	84.4 (81, 89)	89.7 (84, 94)	81.0 (71, 94)
Week 26: CHG			
N	6	10	6
Missing	7	12	11
Mean (SD)	-0.5 (1.67)	-0.2 (3.03)	-1.3 (4.92)

Abbreviations: N, number of randomized subjects; n, number of subjects within each category; SD, Standard Deviation; Min, Minimum; Max, Maximum.

Table 14.3.5.2: Summary of Clinical Laboratory Test Results

	Naive (N=13)	Biologic (N=22)	Multiple Biologic (N=17)
Median (Min, Max)	0.0 (-3, 1)	-0.5 (-6, 5)	0.1 (-10, 3)
Week 26: PCHG			
N	6	10	6
Missing	7	12	11
Mean (SD)	-0.6 (1.98)	-0.1 (3.41)	-1.4 (6.17)
Median (Min, Max)	0.0 (-4, 1)	-0.6 (-7, 6)	0.2 (-12, 4)
Week 36: OBS			
N	8	10	9
Missing	5	12	8
Mean (SD)	86.5 (2.30)	87.4 (5.71)	82.6 (9.89)
Median (Min, Max)	86.5 (83, 89)	88.2 (77, 95)	84.0 (68, 95)
Week 36: CHG			
N	8	10	9
Missing	5	12	8
Mean (SD)	2.1 (2.30)	-1.3 (4.08)	-1.0 (5.71)
Median (Min, Max)	2.1 (-2, 5)	0.2 (-11, 2)	-1.3 (-13, 5)
Week 36: PCHG			
N	8	10	9
Missing	5	12	8
Mean (SD)	2.5 (2.81)	-1.5 (4.58)	-1.2 (7.08)
Median (Min, Max)	2.5 (-2, 6)	0.2 (-12, 2)	-1.7 (-16, 7)
Week 52: OBS			
N	9	13	10
Missing	4	9	7
Mean (SD)	87.2 (2.84)	87.0 (5.65)	84.3 (6.26)

Abbreviations: N, number of randomized subjects; n, number of subjects within each category; SD, Standard Deviation; Min, Minimum; Max, Maximum.

Table 14.3.5.2: Summary of Clinical Laboratory Test Results

	Naive (N=13)	Biologic (N=22)	Multiple Biologic (N=17)
Median (Min, Max)	86.0 (84, 93)	89.0 (78, 94)	85.5 (74, 94)
Week 52: CHG			
N	9	13	10
Missing	4	9	7
Mean (SD)	2.1 (2.10)	-1.3 (4.76)	1.0 (4.11)
Median (Min, Max)	2.0 (-1, 6)	-1.0 (-13, 7)	1.4 (-5, 9)
Week 52: PCHG			
N	9	13	10
Missing	4	9	7
Mean (SD)	2.6 (2.60)	-1.5 (5.34)	1.5 (5.17)
Median (Min, Max)	2.4 (-1, 8)	-1.1 (-14, 8)	1.6 (-5, 12)
Early Termination: OBS			
N	0	3	2
Missing	-	-	-
Mean (SD)	-	87.5 (0.45)	90.3 (1.77)
Median (Min, Max)	-	87.5 (87, 88)	90.3 (89, 92)
Early Termination: CHG			
N	0	3	2
Missing	-	-	-
Mean (SD)	-	-0.4 (1.74)	2.0 (4.31)
Median (Min, Max)	-	-1.0 (-2, 2)	2.0 (-1, 5)
Early Termination: PCHG			
N	0	3	2
Missing	-	-	-
Mean (SD)	-	-0.4 (1.99)	2.4 (4.96)

Abbreviations: N, number of randomized subjects; n, number of subjects within each category; SD, Standard Deviation; Min, Minimum; Max, Maximum.

Program Name: MUCUS-Shell11-V01
Output Location: .\Output\Shared\MUCUS-TFLS-Final-27MAR24-V01.pdf

Table 14.3.5.2: Summary of Clinical Laboratory Test Results

	Naive (N=13)	Biologic (N=22)	Multiple Biologic (N=17)
Median (Min, Max)	-	-1.1 (-2, 2)	2.4 (-1, 6)
Disease Flare: OBS			
N	0	0	1
Missing	-	-	-
Mean (SD)	-	-	86.0 (-)
Median (Min, Max)	-	-	86.0 (86, 86)
Disease Flare: CHG			
N	0	0	1
Missing	-	-	-
Mean (SD)	-	-	0.0 (-)
Median (Min, Max)	-	-	0.0 (0, 0)
Disease Flare: PCHG			
N	0	0	1
Missing	-	-	-
Mean (SD)	-	-	0.0 (-)
Median (Min, Max)	-	-	0.0 (0, 0)
Safety Follow-Up: OBS			
N	7	8	10
Missing	6	14	7
Mean (SD)	87.7 (2.59)	86.2 (3.47)	87.3 (5.55)
Median (Min, Max)	87.5 (83, 91)	86.7 (80, 91)	88.5 (75, 94)
Safety Follow-Up: CHG			
N	7	8	10
Missing	6	14	7
Mean (SD)	1.6 (3.39)	-3.5 (4.06)	1.6 (3.86)

Abbreviations: N, number of randomized subjects; n, number of subjects within each category; SD, Standard Deviation; Min, Minimum; Max, Maximum.

Program Name: MUCUS-Shell11-V01
Output Location: .\Output\Shared\MUCUS-TFLS-Final-27MAR24-V01.pdf

Table 14.3.5.2: Summary of Clinical Laboratory Test Results

	Naive (N=13)	Biologic (N=22)	Multiple Biologic (N=17)
Median (Min, Max)	0.3 (-2, 8)	-2.5 (-11, 0)	1.5 (-5, 9)
Safety Follow-Up: PCHG			
N	7	8	10
Missing	6	14	7
Mean (SD)	2.0 (4.14)	-3.8 (4.42)	2.2 (4.85)
Median (Min, Max)	0.3 (-2, 10)	-2.9 (-12, 0)	1.7 (-5, 12)
Mean Platelet Volume			
Baseline: OBS			
N	5	9	3
Missing	8	13	14
Mean (SD)	10.6 (1.41)	10.5 (1.51)	10.3 (0.49)
Median (Min, Max)	11.2 (8, 12)	10.0 (8, 13)	10.1 (10, 11)
Week 8: OBS			
N	4	6	5
Missing	9	16	12
Mean (SD)	10.4 (1.58)	11.2 (1.39)	10.0 (0.68)
Median (Min, Max)	10.5 (9, 12)	10.7 (10, 13)	10.2 (9, 11)
Week 8: CHG			
N	4	6	3
Missing	9	16	14
Mean (SD)	-0.1 (0.83)	0.1 (0.42)	-0.1 (0.46)
Median (Min, Max)	-0.0 (-1, 1)	0.1 (-0, 1)	-0.2 (-1, 0)
Week 8: PCHG			
N	4	6	3
Missing	9	16	14

Abbreviations: N, number of randomized subjects; n, number of subjects within each category; SD, Standard Deviation; Min, Minimum; Max, Maximum.

Program Name: MUCUS-Shell11-V01
Output Location: .\Output\Shared\MUCUS-TFLS-Final-27MAR24-V01.pdf

Table 14.3.5.2: Summary of Clinical Laboratory Test Results

	Naive (N=13)	Biologic (N=22)	Multiple Biologic (N=17)
Mean (SD)	-1.1 (7.79)	1.6 (3.98)	-0.9 (4.54)
Median (Min, Max)	0.1 (-11, 6)	1.2 (-3, 7)	-1.8 (-5, 4)
Week 16: OBS			
N	4	8	3
Missing	9	14	14
Mean (SD)	10.2 (1.30)	11.3 (0.91)	9.3 (0.51)
Median (Min, Max)	10.5 (8, 12)	11.1 (10, 12)	9.2 (9, 10)
Week 16: CHG			
N	3	6	2
Missing	10	16	15
Mean (SD)	-0.1 (0.29)	0.0 (0.83)	-0.5 (0.42)
Median (Min, Max)	-0.3 (-0, 0)	-0.1 (-1, 1)	-0.5 (-1, -0)
Week 16: PCHG			
N	3	6	2
Missing	10	16	15
Mean (SD)	-1.0 (2.95)	1.2 (7.88)	-5.0 (4.26)
Median (Min, Max)	-2.5 (-3, 2)	-0.8 (-7, 15)	-5.0 (-8, -2)
Week 26: OBS			
N	4	5	3
Missing	9	17	14
Mean (SD)	10.9 (0.76)	11.0 (1.19)	10.0 (0.58)
Median (Min, Max)	10.8 (10, 12)	10.4 (10, 13)	10.3 (9, 10)
Week 26: CHG			
N	3	5	1
Missing	10	17	16

Abbreviations: N, number of randomized subjects; n, number of subjects within each category; SD, Standard Deviation; Min, Minimum; Max, Maximum.

Table 14.3.5.2: Summary of Clinical Laboratory Test Results

	Naive (N=13)	Biologic (N=22)	Multiple Biologic (N=17)
Mean (SD)	-0.1 (0.36)	-0.2 (0.69)	-0.6 (-)
Median (Min, Max)	0.0 (-1, 0)	-0.3 (-1, 1)	-0.6 (-1, -1)
Week 26: PCHG			
N	3	5	1
Missing	10	17	16
Mean (SD)	-0.9 (3.18)	-1.6 (6.37)	-5.5 (-)
Median (Min, Max)	0.0 (-4, 2)	-2.3 (-8, 8)	-5.5 (-6, -6)
Week 36: OBS			
N	3	5	4
Missing	10	17	13
Mean (SD)	10.6 (0.55)	11.5 (1.12)	10.0 (0.14)
Median (Min, Max)	10.6 (10, 11)	11.0 (10, 13)	10.1 (10, 10)
Week 36: CHG			
N	2	5	3
Missing	11	17	14
Mean (SD)	-0.6 (0.00)	0.3 (0.59)	-0.3 (0.47)
Median (Min, Max)	-0.6 (-1, -1)	0.1 (-1, 1)	-0.1 (-1, 0)
Week 36: PCHG			
N	2	5	3
Missing	11	17	14
Mean (SD)	-5.2 (0.19)	2.8 (5.67)	-2.4 (4.36)
Median (Min, Max)	-5.2 (-5, -5)	0.8 (-4, 10)	-1.0 (-7, 1)
Week 52: OBS			
N	3	4	3
Missing	10	18	14

Abbreviations: N, number of randomized subjects; n, number of subjects within each category; SD, Standard Deviation; Min, Minimum; Max, Maximum.

Table 14.3.5.2: Summary of Clinical Laboratory Test Results

	Naive (N=13)	Biologic (N=22)	Multiple Biologic (N=17)
Mean (SD)	10.9 (1.00)	11.2 (1.18)	9.9 (0.66)
Median (Min, Max)	10.5 (10, 12)	10.8 (10, 13)	10.0 (9, 11)
Week 52: CHG			
N	2	3	1
Missing	11	19	16
Mean (SD)	-0.3 (0.64)	0.3 (0.47)	-0.1 (-)
Median (Min, Max)	-0.3 (-1, 0)	0.1 (-0, 1)	-0.1 (-0, -0)
Week 52: PCHG			
N	2	3	1
Missing	11	19	16
Mean (SD)	-2.3 (5.62)	2.8 (4.83)	-1.0 (-)
Median (Min, Max)	-2.3 (-6, 2)	1.0 (-1, 8)	-1.0 (-1, -1)
Early Termination: OBS			
N	0	1	1
Missing	-	-	-
Mean (SD)	-	12.1 (-)	10.1 (-)
Median (Min, Max)	-	12.1 (12, 12)	10.1 (10, 10)
Early Termination: CHG			
N	0	1	1
Missing	-	-	-
Mean (SD)	-	-0.8 (-)	0.1 (-)
Median (Min, Max)	-	-0.8 (-1, -1)	0.1 (0, 0)
Early Termination: PCHG			
N	0	1	1
Missing	-	-	-

Abbreviations: N, number of randomized subjects; n, number of subjects within each category; SD, Standard Deviation; Min, Minimum; Max, Maximum.

Program Name: MUCUS-Shell11-V01
Output Location: .\Output\Shared\MUCUS-TFLS-Final-27MAR24-V01.pdf

Table 14.3.5.2: Summary of Clinical Laboratory Test Results

	Naive (N=13)	Biologic (N=22)	Multiple Biologic (N=17)
Mean (SD)	-	-6.2 (-)	1.0 (-)
Median (Min, Max)	-	-6.2 (-6, -6)	1.0 (1, 1)
Disease Flare: OBS			
N	0	0	1
Missing	-	-	-
Mean (SD)	-	-	11.1 (-)
Median (Min, Max)	-	-	11.1 (11, 11)
Disease Flare: CHG			
N	0	0	1
Missing	-	-	-
Mean (SD)	-	-	0.2 (-)
Median (Min, Max)	-	-	0.2 (0, 0)
Disease Flare: PCHG			
N	0	0	1
Missing	-	-	-
Mean (SD)	-	-	1.8 (-)
Median (Min, Max)	-	-	1.8 (2, 2)
Safety Follow-Up: OBS			
N	0	0	0
Missing	13	22	17
Mean (SD)	-	-	-
Median (Min, Max)	-	-	-
Safety Follow-Up: CHG			
N	0	0	0
Missing	13	22	17

Abbreviations: N, number of randomized subjects; n, number of subjects within each category; SD, Standard Deviation; Min, Minimum; Max, Maximum.

Program Name: MUCUS-Shell11-V01
Output Location: .\Output\Shared\MUCUS-TFLS-Final-27MAR24-V01.pdf

Table 14.3.5.2: Summary of Clinical Laboratory Test Results

	Naive (N=13)	Biologic (N=22)	Multiple Biologic (N=17)
Mean (SD)	-	-	-
Median (Min, Max)	-	-	-
Safety Follow-Up: PCHG			
N	0	0	0
Missing	13	22	17
Mean (SD)	-	-	-
Median (Min, Max)	-	-	-
Platelet Count			
Baseline: OBS			
N	13	22	17
Missing	0	0	0
Mean (SD)	368.8 (147.52)	305.6 (96.57)	388.4 (124.51)
Median (Min, Max)	322.0 (218, 786)	286.5 (166, 497)	355.0 (240, 648)
Week 8: OBS			
N	12	20	14
Missing	1	2	3
Mean (SD)	351.4 (96.01)	290.9 (83.50)	357.2 (86.14)
Median (Min, Max)	335.5 (250, 578)	288.0 (166, 481)	341.5 (214, 542)
Week 8: CHG			
N	12	20	14
Missing	1	2	3
Mean (SD)	-21.3 (65.73)	-6.5 (54.74)	-35.9 (83.73)
Median (Min, Max)	-11.5 (-208, 38)	-3.0 (-130, 131)	-31.0 (-178, 146)
Week 8: PCHG			
N	12	20	14

Abbreviations: N, number of randomized subjects; n, number of subjects within each category; SD, Standard Deviation; Min, Minimum; Max, Maximum.

Program Name: MUCUS-Shell11-V01
Output Location: .\Output\Shared\MUCUS-TFLS-Final-27MAR24-V01.pdf

Table 14.3.5.2: Summary of Clinical Laboratory Test Results

	Naive (N=13)	Biologic (N=22)	Multiple Biologic (N=17)
Missing	1	2	3
Mean (SD)	-1.9 (11.96)	-0.4 (17.12)	-5.5 (23.89)
Median (Min, Max)	-4.1 (-26, 15)	-1.2 (-29, 51)	-9.0 (-45, 55)
Week 16: OBS			
N	8	18	12
Missing	5	4	5
Mean (SD)	344.4 (134.69)	276.2 (57.19)	374.1 (84.53)
Median (Min, Max)	329.0 (205, 641)	270.5 (143, 411)	404.5 (218, 483)
Week 16: CHG			
N	8	18	12
Missing	5	4	5
Mean (SD)	-22.0 (75.88)	-16.6 (70.60)	-36.1 (102.04)
Median (Min, Max)	-11.5 (-145, 92)	1.0 (-175, 89)	-34.5 (-219, 127)
Week 16: PCHG			
N	8	18	12
Missing	5	4	5
Mean (SD)	-1.5 (18.01)	-2.2 (19.63)	-4.8 (26.30)
Median (Min, Max)	-5.1 (-23, 33)	0.2 (-40, 28)	-8.8 (-44, 48)
Week 26: OBS			
N	6	10	6
Missing	7	12	11
Mean (SD)	291.3 (69.71)	281.4 (79.93)	377.8 (95.25)
Median (Min, Max)	293.5 (211, 368)	274.0 (186, 445)	380.0 (263, 522)
Week 26: CHG			
N	6	10	6

Abbreviations: N, number of randomized subjects; n, number of subjects within each category; SD, Standard Deviation; Min, Minimum; Max, Maximum.

Table 14.3.5.2: Summary of Clinical Laboratory Test Results

	Naive (N=13)	Biologic (N=22)	Multiple Biologic (N=17)
Missing	7	12	11
Mean (SD)	-38.5 (35.81)	-1.5 (90.73)	-49.7 (81.70)
Median (Min, Max)	-32.5 (-97, -1)	-9.0 (-154, 186)	-70.5 (-129, 64)
Week 26: PCHG			
N	6	10	6
Missing	7	12	11
Mean (SD)	-9.9 (7.48)	2.5 (29.95)	-10.9 (18.53)
Median (Min, Max)	-10.1 (-21, -0)	-4.5 (-35, 72)	-16.6 (-33, 14)
Week 36: OBS			
N	8	10	9
Missing	5	12	8
Mean (SD)	292.8 (72.33)	297.4 (127.39)	361.7 (89.47)
Median (Min, Max)	298.5 (212, 396)	255.0 (164, 523)	360.0 (246, 529)
Week 36: CHG			
N	8	10	9
Missing	5	12	8
Mean (SD)	-51.1 (41.45)	3.0 (106.05)	-43.8 (89.81)
Median (Min, Max)	-43.0 (-115, -6)	1.0 (-168, 236)	-83.0 (-146, 116)
Week 36: PCHG			
N	8	10	9
Missing	5	12	8
Mean (SD)	-13.6 (9.67)	3.7 (35.33)	-8.5 (24.85)
Median (Min, Max)	-10.6 (-28, -3)	0.4 (-38, 91)	-18.9 (-37, 43)
Week 52: OBS			
N	9	13	10

Abbreviations: N, number of randomized subjects; n, number of subjects within each category; SD, Standard Deviation; Min, Minimum; Max, Maximum.

Program Name: MUCUS-Shell11-V01
Output Location: .\Output\Shared\MUCUS-TFLS-Final-27MAR24-V01.pdf

Table 14.3.5.2: Summary of Clinical Laboratory Test Results

	Naive (N=13)	Biologic (N=22)	Multiple Biologic (N=17)
Missing	4	9	7
Mean (SD)	297.6 (76.04)	284.2 (89.36)	314.1 (71.67)
Median (Min, Max)	322.0 (183, 404)	272.0 (163, 490)	302.0 (211, 414)
Week 52: CHG			
N	9	13	10
Missing	4	9	7
Mean (SD)	-55.1 (42.71)	-23.8 (91.18)	-93.9 (149.33)
Median (Min, Max)	-41.0 (-143, 8)	-3.0 (-162, 168)	-78.0 (-379, 147)
Week 52: PCHG			
N	9	13	10
Missing	4	9	7
Mean (SD)	-15.5 (9.82)	-3.5 (28.11)	-16.1 (32.70)
Median (Min, Max)	-12.7 (-31, 2)	-1.8 (-37, 65)	-19.3 (-58, 55)
Early Termination: OBS			
N	0	3	2
Missing	-	-	-
Mean (SD)	-	253.7 (65.53)	272.0 (1.41)
Median (Min, Max)	-	256.0 (187, 318)	272.0 (271, 273)
Early Termination: CHG			
N	0	3	2
Missing	-	-	-
Mean (SD)	-	-7.7 (5.51)	-40.5 (60.10)
Median (Min, Max)	-	-8.0 (-13, -2)	-40.5 (-83, 2)
Early Termination: PCHG			
N	0	3	2

Abbreviations: N, number of randomized subjects; n, number of subjects within each category; SD, Standard Deviation; Min, Minimum; Max, Maximum.

Program Name: MUCUS-Shell11-V01
Output Location: .\Output\Shared\MUCUS-TFLS-Final-27MAR24-V01.pdf

Table 14.3.5.2: Summary of Clinical Laboratory Test Results

	Naive (N=13)	Biologic (N=22)	Multiple Biologic (N=17)
Missing	-	-	-
Mean (SD)	-	-3.2 (2.25)	-11.3 (17.01)
Median (Min, Max)	-	-4.1 (-5, -1)	-11.3 (-23, 1)
Disease Flare: OBS			
N	0	0	1
Missing	-	-	-
Mean (SD)	-	-	334.0 (-)
Median (Min, Max)	-	-	334.0 (334, 334)
Disease Flare: CHG			
N	0	0	1
Missing	-	-	-
Mean (SD)	-	-	-6.0 (-)
Median (Min, Max)	-	-	-6.0 (-6, -6)
Disease Flare: PCHG			
N	0	0	1
Missing	-	-	-
Mean (SD)	-	-	-1.8 (-)
Median (Min, Max)	-	-	-1.8 (-2, -2)
Safety Follow-Up: OBS			
N	7	8	10
Missing	6	14	7
Mean (SD)	314.3 (42.42)	276.3 (54.07)	294.6 (53.63)
Median (Min, Max)	322.0 (260, 374)	287.0 (194, 368)	297.5 (207, 374)
Safety Follow-Up: CHG			
N	7	8	10

Abbreviations: N, number of randomized subjects; n, number of subjects within each category; SD, Standard Deviation; Min, Minimum; Max, Maximum.

Table 14.3.5.2: Summary of Clinical Laboratory Test Results

	Naive (N=13)	Biologic (N=22)	Multiple Biologic (N=17)
Missing	6	14	7
Mean (SD)	-43.6 (65.88)	-20.8 (93.38)	-85.6 (123.56)
Median (Min, Max)	-42.0 (-158, 44)	-6.0 (-205, 109)	-46.0 (-388, 58)
Safety Follow-Up: PCHG			
N	7	8	10
Missing	6	14	7
Mean (SD)	-9.6 (16.40)	-2.3 (27.31)	-17.2 (22.30)
Median (Min, Max)	-10.6 (-36, 16)	-1.8 (-47, 42)	-14.2 (-60, 22)
Red Blood Cell Count			
Baseline: OBS			
N	13	22	17
Missing	0	0	0
Mean (SD)	4.4 (0.41)	4.3 (0.92)	4.6 (0.53)
Median (Min, Max)	4.4 (4, 5)	4.5 (2, 6)	4.6 (3, 5)
Week 8: OBS			
N	12	20	14
Missing	1	2	3
Mean (SD)	4.7 (0.53)	4.5 (0.72)	4.8 (0.48)
Median (Min, Max)	4.7 (4, 5)	4.6 (2, 6)	4.9 (4, 6)
Week 8: CHG			
N	12	20	14
Missing	1	2	3
Mean (SD)	0.2 (0.51)	0.3 (0.54)	0.3 (0.32)
Median (Min, Max)	0.3 (-1, 1)	0.1 (-0, 2)	0.3 (-1, 1)
Week 8: PCHG			

Abbreviations: N, number of randomized subjects; n, number of subjects within each category; SD, Standard Deviation; Min, Minimum; Max, Maximum.

Program Name: MUCUS-Shell11-V01
Output Location: .\Output\Shared\MUCUS-TFLS-Final-27MAR24-V01.pdf

Table 14.3.5.2: Summary of Clinical Laboratory Test Results

	Naive (N=13)	Biologic (N=22)	Multiple Biologic (N=17)
N	12	20	14
Missing	1	2	3
Mean (SD)	4.8 (11.78)	10.3 (30.07)	6.8 (8.78)
Median (Min, Max)	5.1 (-18, 26)	2.6 (-8, 135)	5.6 (-11, 29)
Week 16: OBS			
N	8	18	12
Missing	5	4	5
Mean (SD)	4.8 (0.44)	4.6 (0.44)	4.8 (0.61)
Median (Min, Max)	4.9 (4, 5)	4.5 (4, 6)	4.7 (4, 6)
Week 16: CHG			
N	8	18	12
Missing	5	4	5
Mean (SD)	0.2 (0.35)	0.3 (0.64)	0.4 (0.40)
Median (Min, Max)	0.1 (-0, 1)	0.2 (-0, 3)	0.2 (-0, 1)
Week 16: PCHG			
N	8	18	12
Missing	5	4	5
Mean (SD)	5.1 (8.43)	13.1 (35.61)	8.9 (10.44)
Median (Min, Max)	2.3 (-2, 23)	5.3 (-7, 153)	4.2 (-2, 29)
Week 26: OBS			
N	6	10	6
Missing	7	12	11
Mean (SD)	4.8 (0.35)	4.5 (0.27)	4.9 (0.27)
Median (Min, Max)	4.7 (5, 5)	4.4 (4, 5)	4.9 (5, 5)
Week 26: CHG			

Abbreviations: N, number of randomized subjects; n, number of subjects within each category; SD, Standard Deviation; Min, Minimum; Max, Maximum.

Table 14.3.5.2: Summary of Clinical Laboratory Test Results

	Naive (N=13)	Biologic (N=22)	Multiple Biologic (N=17)
N	6	10	6
Missing	7	12	11
Mean (SD)	0.2 (0.29)	0.1 (0.30)	0.3 (0.28)
Median (Min, Max)	0.1 (-0, 1)	0.0 (-0, 1)	0.3 (-0, 1)
Week 26: PCHG			
N	6	10	6
Missing	7	12	11
Mean (SD)	4.1 (6.36)	3.7 (7.52)	6.2 (6.06)
Median (Min, Max)	3.3 (-4, 13)	1.0 (-7, 19)	7.3 (-2, 13)
Week 36: OBS			
N	8	10	9
Missing	5	12	8
Mean (SD)	4.7 (0.39)	4.8 (0.51)	4.9 (0.37)
Median (Min, Max)	4.7 (4, 5)	4.5 (4, 5)	4.9 (4, 5)
Week 36: CHG			
N	8	10	9
Missing	5	12	8
Mean (SD)	0.2 (0.43)	0.3 (0.27)	0.3 (0.27)
Median (Min, Max)	0.0 (-0, 1)	0.3 (-0, 1)	0.3 (-0, 1)
Week 36: PCHG			
N	8	10	9
Missing	5	12	8
Mean (SD)	4.5 (10.11)	7.4 (6.51)	6.6 (6.15)
Median (Min, Max)	0.2 (-4, 26)	7.2 (-2, 20)	5.7 (-4, 19)
Week 52: OBS			

Abbreviations: N, number of randomized subjects; n, number of subjects within each category; SD, Standard Deviation; Min, Minimum; Max, Maximum.

Program Name: MUCUS-Shell11-V01
Output Location: .\Output\Shared\MUCUS-TFLS-Final-27MAR24-V01.pdf

Table 14.3.5.2: Summary of Clinical Laboratory Test Results

	Naive (N=13)	Biologic (N=22)	Multiple Biologic (N=17)
N	9	13	10
Missing	4	9	7
Mean (SD)	4.6 (0.39)	4.8 (0.55)	5.0 (0.39)
Median (Min, Max)	4.7 (4, 5)	4.9 (4, 6)	4.9 (5, 6)
Week 52: CHG			
N	9	13	10
Missing	4	9	7
Mean (SD)	0.2 (0.34)	0.6 (0.74)	0.4 (0.26)
Median (Min, Max)	0.2 (-0, 1)	0.4 (-0, 3)	0.3 (0, 1)
Week 52: PCHG			
N	9	13	10
Missing	4	9	7
Mean (SD)	5.2 (7.83)	21.2 (41.24)	7.9 (5.90)
Median (Min, Max)	4.7 (-4, 19)	9.2 (-4, 153)	6.5 (2, 17)
Early Termination: OBS			
N	0	3	2
Missing	-	-	-
Mean (SD)	-	4.9 (0.49)	4.3 (0.19)
Median (Min, Max)	-	4.8 (5, 5)	4.3 (4, 4)
Early Termination: CHG			
N	0	3	2
Missing	-	-	-
Mean (SD)	-	-0.1 (0.11)	0.7 (0.52)
Median (Min, Max)	-	-0.1 (-0, 0)	0.7 (0, 1)
Early Termination: PCHG			

Abbreviations: N, number of randomized subjects; n, number of subjects within each category; SD, Standard Deviation; Min, Minimum; Max, Maximum.

Program Name: MUCUS-Shell11-V01
Output Location: .\Output\Shared\MUCUS-TFLS-Final-27MAR24-V01.pdf

Table 14.3.5.2: Summary of Clinical Laboratory Test Results

	Naive (N=13)	Biologic (N=22)	Multiple Biologic (N=17)
N	0	3	2
Missing	-	-	-
Mean (SD)	-	-1.1 (2.09)	22.1 (18.90)
Median (Min, Max)	-	-2.2 (-3, 1)	22.1 (9, 35)
Disease Flare: OBS			
N	0	0	1
Missing	-	-	-
Mean (SD)	-	-	5.0 (-)
Median (Min, Max)	-	-	5.0 (5, 5)
Disease Flare: CHG			
N	0	0	1
Missing	-	-	-
Mean (SD)	-	-	0.1 (-)
Median (Min, Max)	-	-	0.1 (0, 0)
Disease Flare: PCHG			
N	0	0	1
Missing	-	-	-
Mean (SD)	-	-	2.0 (-)
Median (Min, Max)	-	-	2.0 (2, 2)
Safety Follow-Up: OBS			
N	7	8	10
Missing	6	14	7
Mean (SD)	4.5 (0.57)	4.3 (0.85)	4.7 (0.60)
Median (Min, Max)	4.5 (4, 5)	4.4 (3, 5)	4.7 (4, 6)
Safety Follow-Up: CHG			

Abbreviations: N, number of randomized subjects; n, number of subjects within each category; SD, Standard Deviation; Min, Minimum; Max, Maximum.

Program Name: MUCUS-Shell11-V01
Output Location: .\Output\Shared\MUCUS-TFLS-Final-27MAR24-V01.pdf

Table 14.3.5.2: Summary of Clinical Laboratory Test Results

	Naive (N=13)	Biologic (N=22)	Multiple Biologic (N=17)
N	7	8	10
Missing	6	14	7
Mean (SD)	0.1 (0.38)	0.4 (0.85)	0.2 (0.25)
Median (Min, Max)	0.2 (-1, 1)	0.1 (-0, 2)	0.3 (-0, 1)
Safety Follow-Up: PCHG			
N	7	8	10
Missing	6	14	7
Mean (SD)	2.6 (8.51)	19.9 (49.35)	5.7 (6.02)
Median (Min, Max)	5.3 (-11, 12)	2.7 (-5, 141)	5.8 (-7, 13)
Red Cell Distribution Width			
Baseline: OBS			
N	13	19	16
Missing	0	3	1
Mean (SD)	14.3 (1.52)	14.6 (3.31)	14.7 (2.55)
Median (Min, Max)	14.2 (13, 17)	13.2 (12, 23)	14.0 (11, 22)
Week 8: OBS			
N	12	18	14
Missing	1	4	3
Mean (SD)	13.9 (1.35)	14.2 (2.84)	15.5 (2.64)
Median (Min, Max)	13.4 (12, 16)	13.2 (11, 23)	14.7 (12, 20)
Week 8: CHG			
N	12	17	13
Missing	1	5	4
Mean (SD)	-0.6 (1.23)	-0.2 (1.17)	0.4 (2.11)
Median (Min, Max)	-0.5 (-3, 1)	0.0 (-3, 2)	0.2 (-2, 6)

Abbreviations: N, number of randomized subjects; n, number of subjects within each category; SD, Standard Deviation; Min, Minimum; Max, Maximum.

Program Name: MUCUS-Shell11-V01
Output Location: .\Output\Shared\MUCUS-TFLS-Final-27MAR24-V01.pdf

Table 14.3.5.2: Summary of Clinical Laboratory Test Results

	Naive (N=13)	Biologic (N=22)	Multiple Biologic (N=17)
Week 8: PCHG			
N	12	17	13
Missing	1	5	4
Mean (SD)	-3.6 (8.11)	-0.8 (6.61)	3.5 (14.27)
Median (Min, Max)	-3.1 (-19, 10)	0.0 (-14, 12)	1.5 (-14, 44)
Week 16: OBS			
N	7	16	11
Missing	6	6	6
Mean (SD)	13.0 (0.81)	13.1 (3.27)	15.8 (3.63)
Median (Min, Max)	13.2 (12, 14)	12.9 (5, 21)	14.9 (13, 24)
Week 16: CHG			
N	7	15	10
Missing	6	7	7
Mean (SD)	-1.0 (1.03)	-0.9 (2.24)	-0.2 (1.49)
Median (Min, Max)	-0.8 (-3, 0)	-0.2 (-8, 1)	0.1 (-3, 2)
Week 16: PCHG			
N	7	15	10
Missing	6	7	7
Mean (SD)	-6.9 (6.70)	-6.3 (17.15)	-0.5 (9.57)
Median (Min, Max)	-5.6 (-20, 2)	-1.7 (-64, 9)	0.8 (-20, 13)
Week 26: OBS			
N	6	10	6
Missing	7	12	11
Mean (SD)	13.5 (1.30)	13.4 (0.93)	15.2 (2.92)
Median (Min, Max)	13.1 (12, 16)	13.2 (12, 16)	14.8 (13, 20)

Abbreviations: N, number of randomized subjects; n, number of subjects within each category; SD, Standard Deviation; Min, Minimum; Max, Maximum.

Program Name: MUCUS-Shell11-V01
Output Location: .\Output\Shared\MUCUS-TFLS-Final-27MAR24-V01.pdf

Table 14.3.5.2: Summary of Clinical Laboratory Test Results

	Naive (N=13)	Biologic (N=22)	Multiple Biologic (N=17)
Week 26: CHG			
N	6	9	6
Missing	7	13	11
Mean (SD)	-0.5 (2.09)	-0.2 (1.24)	-0.4 (3.80)
Median (Min, Max)	-0.8 (-3, 3)	0.1 (-3, 1)	-0.5 (-5, 6)
Week 26: PCHG			
N	6	9	6
Missing	7	13	11
Mean (SD)	-2.8 (14.83)	-0.6 (7.55)	0.1 (24.01)
Median (Min, Max)	-5.4 (-18, 24)	0.8 (-17, 10)	-3.3 (-23, 42)
Week 36: OBS			
N	8	9	9
Missing	5	13	8
Mean (SD)	13.0 (0.66)	13.4 (1.04)	14.7 (2.12)
Median (Min, Max)	13.1 (12, 14)	13.3 (12, 15)	13.8 (12, 18)
Week 36: CHG			
N	8	8	8
Missing	5	14	9
Mean (SD)	-1.6 (1.42)	0.4 (0.79)	-1.0 (1.72)
Median (Min, Max)	-1.1 (-4, -0)	0.3 (-1, 2)	-0.8 (-4, 1)
Week 36: PCHG			
N	8	8	8
Missing	5	14	9
Mean (SD)	-10.1 (8.17)	3.1 (6.07)	-5.4 (9.67)
Median (Min, Max)	-8.2 (-24, -2)	1.8 (-5, 13)	-5.5 (-19, 9)

Abbreviations: N, number of randomized subjects; n, number of subjects within each category; SD, Standard Deviation; Min, Minimum; Max, Maximum.

Program Name: MUCUS-Shell11-V01
Output Location: .\Output\Shared\MUCUS-TFLS-Final-27MAR24-V01.pdf

Table 14.3.5.2: Summary of Clinical Laboratory Test Results

	Naive (N=13)	Biologic (N=22)	Multiple Biologic (N=17)
Week 52: OBS			
N	9	13	10
Missing	4	9	7
Mean (SD)	12.9 (0.55)	13.4 (1.15)	14.5 (2.16)
Median (Min, Max)	13.1 (12, 14)	13.4 (12, 15)	14.1 (13, 20)
Week 52: CHG			
N	9	11	9
Missing	4	11	8
Mean (SD)	-1.6 (1.34)	-0.6 (2.77)	-0.4 (1.38)
Median (Min, Max)	-1.7 (-4, 0)	-0.1 (-9, 2)	0.2 (-3, 1)
Week 52: PCHG			
N	9	11	9
Missing	4	11	8
Mean (SD)	-10.1 (7.91)	-2.3 (13.87)	-1.8 (9.03)
Median (Min, Max)	-11.6 (-22, 3)	-0.9 (-39, 17)	1.4 (-19, 8)
Early Termination: OBS			
N	0	2	2
Missing	-	-	-
Mean (SD)	-	13.2 (0.78)	17.5 (5.23)
Median (Min, Max)	-	13.2 (13, 14)	17.5 (14, 21)
Early Termination: CHG			
N	0	2	2
Missing	-	-	-
Mean (SD)	-	0.6 (0.85)	2.3 (4.31)
Median (Min, Max)	-	0.6 (0, 1)	2.3 (-1, 5)

Abbreviations: N, number of randomized subjects; n, number of subjects within each category; SD, Standard Deviation; Min, Minimum; Max, Maximum.

Program Name: MUCUS-Shell11-V01
Output Location: .\Output\Shared\MUCUS-TFLS-Final-27MAR24-V01.pdf

Table 14.3.5.2: Summary of Clinical Laboratory Test Results

	Naive (N=13)	Biologic (N=22)	Multiple Biologic (N=17)
Early Termination: PCHG			
N	0	2	2
Missing	-	-	-
Mean (SD)	-	4.8 (6.79)	13.9 (27.44)
Median (Min, Max)	-	4.8 (0, 10)	13.9 (-5, 33)
Disease Flare: OBS			
N	0	0	1
Missing	-	-	-
Mean (SD)	-	-	12.2 (-)
Median (Min, Max)	-	-	12.2 (12, 12)
Disease Flare: CHG			
N	0	0	1
Missing	-	-	-
Mean (SD)	-	-	-0.7 (-)
Median (Min, Max)	-	-	-0.7 (-1, -1)
Disease Flare: PCHG			
N	0	0	1
Missing	-	-	-
Mean (SD)	-	-	-5.4 (-)
Median (Min, Max)	-	-	-5.4 (-5, -5)
Safety Follow-Up: OBS			
N	7	8	10
Missing	6	14	7
Mean (SD)	13.0 (0.35)	14.8 (3.78)	13.4 (1.10)
Median (Min, Max)	13.2 (13, 13)	14.2 (11, 23)	13.7 (12, 15)

Abbreviations: N, number of randomized subjects; n, number of subjects within each category; SD, Standard Deviation; Min, Minimum; Max, Maximum.

Program Name: MUCUS-Shell11-V01
Output Location: .\Output\Shared\MUCUS-TFLS-Final-27MAR24-V01.pdf

Table 14.3.5.2: Summary of Clinical Laboratory Test Results

	Naive (N=13)	Biologic (N=22)	Multiple Biologic (N=17)
Safety Follow-Up: CHG			
N	7	6	10
Missing	6	16	7
Mean (SD)	-0.8 (1.04)	-1.0 (2.56)	-0.7 (1.31)
Median (Min, Max)	-0.6 (-2, 0)	-0.1 (-6, 1)	0.0 (-3, 1)
Safety Follow-Up: PCHG			
N	7	6	10
Missing	6	16	7
Mean (SD)	-5.6 (7.07)	-4.3 (12.31)	-4.3 (8.38)
Median (Min, Max)	-4.5 (-16, 3)	-1.1 (-27, 9)	0.0 (-21, 4)
White Blood Cell Count			
Baseline: OBS			
N	13	22	17
Missing	0	0	0
Mean (SD)	10.3 (5.31)	8.9 (3.47)	9.4 (3.47)
Median (Min, Max)	8.2 (6, 23)	7.5 (5, 19)	9.9 (5, 17)
Week 8: OBS			
N	12	20	14
Missing	1	2	3
Mean (SD)	8.3 (2.76)	8.2 (2.85)	8.5 (1.68)
Median (Min, Max)	7.1 (5, 14)	7.5 (5, 15)	8.4 (6, 12)
Week 8: CHG			
N	12	20	14
Missing	1	2	3
Mean (SD)	-2.2 (4.19)	-0.7 (2.56)	-1.5 (3.70)
Median (Min, Max)	-0.7 (-12, 2)	-0.2 (-9, 3)	-0.8 (-9, 4)

Abbreviations: N, number of randomized subjects; n, number of subjects within each category; SD, Standard Deviation; Min, Minimum; Max, Maximum.

Program Name: MUCUS-Shell11-V01
Output Location: .\Output\Shared\MUCUS-TFLS-Final-27MAR24-V01.pdf

Table 14.3.5.2: Summary of Clinical Laboratory Test Results

	Naive (N=13)	Biologic (N=22)	Multiple Biologic (N=17)
Week 8: PCHG			
N	12	20	14
Missing	1	2	3
Mean (SD)	-11.2 (29.80)	-3.8 (21.59)	-6.8 (31.02)
Median (Min, Max)	-10.0 (-52, 29)	-2.2 (-51, 37)	-8.7 (-54, 47)
Week 16: OBS			
N	8	18	12
Missing	5	4	5
Mean (SD)	7.1 (1.89)	8.8 (3.67)	8.0 (2.00)
Median (Min, Max)	6.9 (5, 10)	7.2 (5, 19)	7.4 (5, 13)
Week 16: CHG			
N	8	18	12
Missing	5	4	5
Mean (SD)	-1.2 (1.75)	-0.3 (3.89)	-2.1 (3.79)
Median (Min, Max)	-0.9 (-5, 1)	-0.9 (-8, 11)	-1.7 (-10, 2)
Week 16: PCHG			
N	8	18	12
Missing	5	4	5
Mean (SD)	-11.2 (14.82)	4.4 (46.46)	-13.0 (30.74)
Median (Min, Max)	-13.8 (-35, 15)	-9.3 (-42, 156)	-17.7 (-59, 41)
Week 26: OBS			
N	6	10	6
Missing	7	12	11
Mean (SD)	6.2 (1.14)	10.4 (4.09)	8.7 (1.83)
Median (Min, Max)	6.0 (5, 8)	9.6 (5, 16)	8.2 (6, 11)

Abbreviations: N, number of randomized subjects; n, number of subjects within each category; SD, Standard Deviation; Min, Minimum; Max, Maximum.

Program Name: MUCUS-Shell11-V01
Output Location: .\Output\Shared\MUCUS-TFLS-Final-27MAR24-V01.pdf

Table 14.3.5.2: Summary of Clinical Laboratory Test Results

	Naive (N=13)	Biologic (N=22)	Multiple Biologic (N=17)
Week 26: CHG			
N	6	10	6
Missing	7	12	11
Mean (SD)	-1.7 (2.30)	0.7 (3.46)	-1.1 (3.68)
Median (Min, Max)	-0.9 (-5, 0)	0.7 (-4, 9)	0.4 (-7, 3)
Week 26: PCHG			
N	6	10	6
Missing	7	12	11
Mean (SD)	-17.2 (22.64)	12.1 (47.43)	-0.7 (36.55)
Median (Min, Max)	-13.2 (-50, 6)	6.4 (-44, 134)	4.5 (-41, 57)
Week 36: OBS			
N	8	10	9
Missing	5	12	8
Mean (SD)	7.4 (2.66)	8.5 (3.39)	8.3 (2.23)
Median (Min, Max)	6.6 (5, 12)	7.5 (4, 14)	7.6 (5, 11)
Week 36: CHG			
N	8	10	9
Missing	5	12	8
Mean (SD)	-4.1 (5.04)	-0.6 (2.36)	-1.4 (3.45)
Median (Min, Max)	-1.9 (-15, -0)	-1.0 (-4, 3)	-0.3 (-7, 3)
Week 36: PCHG			
N	8	10	9
Missing	5	12	8
Mean (SD)	-27.0 (22.84)	-2.5 (32.46)	-8.3 (31.72)
Median (Min, Max)	-20.9 (-63, -5)	-9.1 (-45, 46)	-3.9 (-55, 35)

Abbreviations: N, number of randomized subjects; n, number of subjects within each category; SD, Standard Deviation; Min, Minimum; Max, Maximum.

Table 14.3.5.2: Summary of Clinical Laboratory Test Results

	Naive (N=13)	Biologic (N=22)	Multiple Biologic (N=17)
Week 52: OBS			
N	9	13	10
Missing	4	9	7
Mean (SD)	6.8 (1.77)	7.9 (3.19)	7.0 (1.41)
Median (Min, Max)	7.5 (4, 9)	7.1 (5, 15)	7.0 (5, 9)
Week 52: CHG			
N	9	13	10
Missing	4	9	7
Mean (SD)	-4.6 (5.11)	-0.6 (1.65)	-3.4 (4.11)
Median (Min, Max)	-2.9 (-15, 1)	-0.1 (-4, 2)	-2.6 (-11, 1)
Week 52: PCHG			
N	9	13	10
Missing	4	9	7
Mean (SD)	-31.3 (24.23)	-3.6 (19.82)	-26.2 (27.49)
Median (Min, Max)	-26.9 (-63, 10)	-1.7 (-43, 37)	-33.0 (-65, 21)
Early Termination: OBS			
N	0	3	2
Missing	-	-	-
Mean (SD)	-	9.4 (3.36)	6.0 (0.28)
Median (Min, Max)	-	8.1 (7, 13)	6.0 (6, 6)
Early Termination: CHG			
N	0	3	2
Missing	-	-	-
Mean (SD)	-	-0.6 (1.52)	-4.1 (0.28)
Median (Min, Max)	-	-0.3 (-2, 1)	-4.1 (-4, -4)

Abbreviations: N, number of randomized subjects; n, number of subjects within each category; SD, Standard Deviation; Min, Minimum; Max, Maximum.

Table 14.3.5.2: Summary of Clinical Laboratory Test Results

	Naive (N=13)	Biologic (N=22)	Multiple Biologic (N=17)
Early Termination: PCHG			
N	0	3	2
Missing	-	-	-
Mean (SD)	-	-5.2 (17.90)	-40.6 (2.80)
Median (Min, Max)	-	-2.1 (-24, 11)	-40.6 (-43, -39)
Disease Flare: OBS			
N	0	0	1
Missing	-	-	-
Mean (SD)	-	-	12.1 (-)
Median (Min, Max)	-	-	12.1 (12, 12)
Disease Flare: CHG			
N	0	0	1
Missing	-	-	-
Mean (SD)	-	-	2.0 (-)
Median (Min, Max)	-	-	2.0 (2, 2)
Disease Flare: PCHG			
N	0	0	1
Missing	-	-	-
Mean (SD)	-	-	20.1 (-)
Median (Min, Max)	-	-	20.1 (20, 20)
Safety Follow-Up: OBS			
N	7	8	10
Missing	6	14	7
Mean (SD)	6.5 (1.89)	7.0 (1.52)	7.7 (2.66)
Median (Min, Max)	6.5 (4, 10)	6.8 (5, 10)	7.1 (5, 14)

Abbreviations: N, number of randomized subjects; n, number of subjects within each category; SD, Standard Deviation; Min, Minimum; Max, Maximum.

Program Name: MUCUS-Shell11-V01
Output Location: .\Output\Shared\MUCUS-TFLS-Final-27MAR24-V01.pdf

Table 14.3.5.2: Summary of Clinical Laboratory Test Results

	Naive (N=13)	Biologic (N=22)	Multiple Biologic (N=17)
Safety Follow-Up: CHG			
N	7	8	10
Missing	6	14	7
Mean (SD)	-4.5 (5.78)	-1.7 (3.53)	-1.7 (3.66)
Median (Min, Max)	-3.6 (-16, 1)	-1.2 (-9, 3)	-0.5 (-8, 6)
Safety Follow-Up: PCHG			
N	7	8	10
Missing	6	14	7
Mean (SD)	-29.8 (30.25)	-10.7 (31.36)	-12.2 (35.47)
Median (Min, Max)	-33.3 (-72, 11)	-16.8 (-48, 48)	-5.8 (-50, 71)

Abbreviations: N, number of randomized subjects; n, number of subjects within each category; SD, Standard Deviation; Min, Minimum; Max, Maximum.

Program Name: MUCUS-Shell11-V01
Output Location: .\Output\Shared\MUCUS-TFLS-Final-27MAR24-V01.pdf

Table 14.4.1: Time to CD-Related Hospitalization

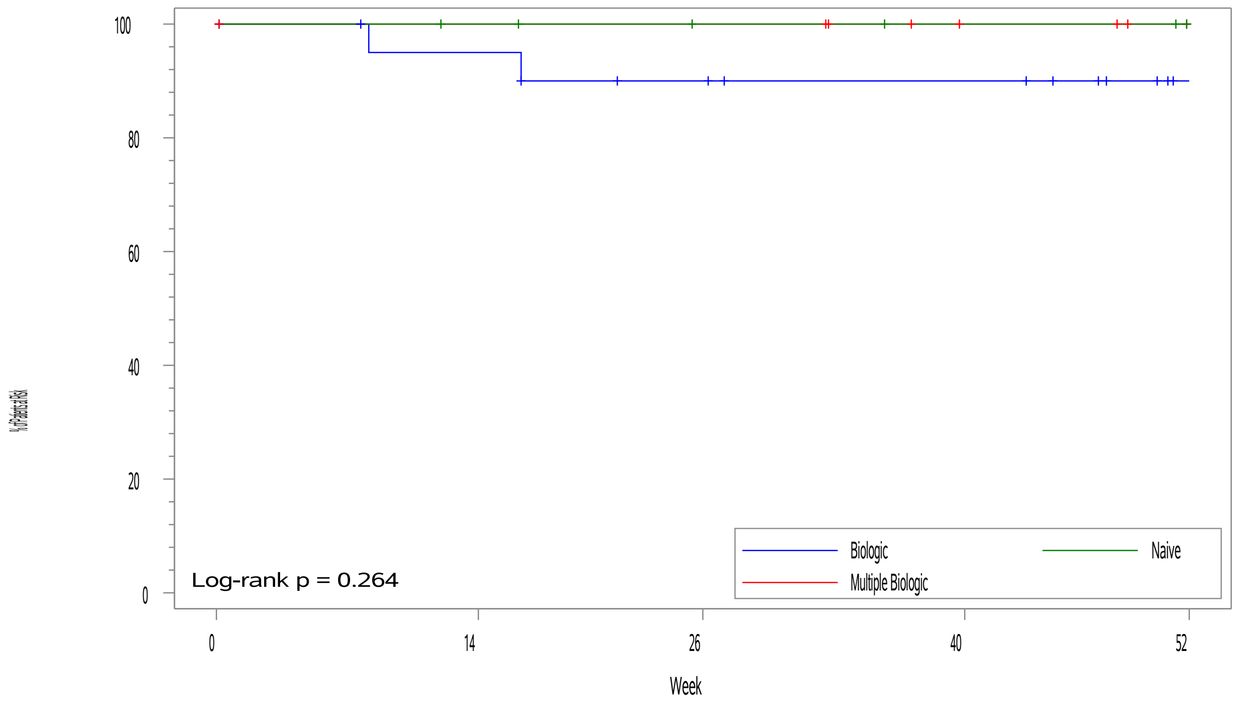

	0	14	26	40	52
**Subjects at Risk**					
Biologic	22	20	17	15	8
Multiple Biologic	17	17	17	11	8
Naive	13	13	11	10	8
**Number Surviving**					
Biologic	100.0%	95.0%	90.0%	90.0%	90.0%
Multiple Biologic	100.0%	100.0%	100.0%	100.0%	100.0%
Naive	100.0%	100.0%	100.0%	100.0%	100.0%

Table 14.4.2: Time to CD-Related Surgery

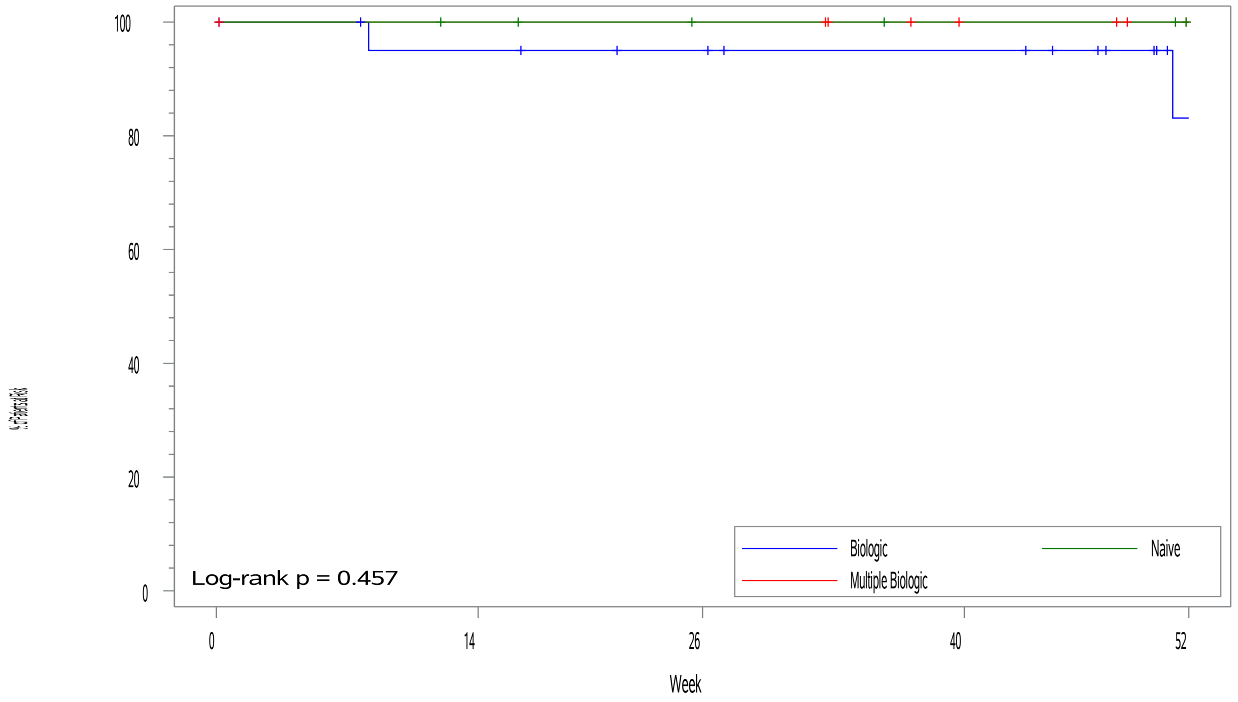

	0	14	26	40	52
**Subjects at Risk**					
Biologic	22	20	18	16	8
Multiple Biologic	17	17	17	11	8
Naive	13	13	11	10	8
**Number Surviving**					
Biologic	100.0%	95.0%	95.0%	95.0%	83.1%
Multiple Biologic	100.0%	100.0%	100.0%	100.0%	100.0%
Naive	100.0%	100.0%	100.0%	100.0%	100.0%

Table 14.4.3: Summary of Clinical Response and Endoscopic Response Exploratory Analysis at Week 52: ITT Population

	N	Odds Ratio (95% CI)	P-Value
Week 52			
Clinical Response + Endoscopic Response			
Segnificant Factor			
Age	52	0.933(0.873, 0.998)	0.043
CRP	52	1.014(0.996, 1.032)	0.124

Abbreviations: ITT, Intent-to-Treat Analysis set; N, number of randomized subjects; n, number of subjects within each category;

Outcome definition, Reduction from baseline in SES-CD score >= 50% and Harvey Bradshaw Index decrease >= 3 points.

Proc Logistic used to generate Odds Ratios and P-values.

Variables included in the full model: Prior Crohn's Treatment Strata, Age, Sex, Age at diagnosis, disease phenotype, extraintestinal manifestations, C-Reactive Protein, Fecal Calprotectin, Albumin, EuroQoL-5D Health Questionnaire Score.

Variables not significant at the 0.15 level were removed from the model by backwards elimination. Effect estimations for each variable kept in the model after backwards elimination are reported.

Program Name: MUCUS-Shell20-V01
Output Location: .\Output\Shared\MUCUS-TFLS-Final-27MAR24-V01.pdf
